# Asc-1 regulates white versus beige adipocyte fate in a subcutaneous stromal cell population

**DOI:** 10.1038/s41467-021-21826-9

**Published:** 2021-03-11

**Authors:** Lisa Suwandhi, Irem Altun, Ruth Karlina, Viktorian Miok, Tobias Wiedemann, David Fischer, Thomas Walzthoeni, Christina Lindner, Anika Böttcher, Silke S. Heinzmann, Andreas Israel, Ahmed Elagamy Mohamed Mahmoud Khalil, Alexander Braun, Ines Pramme-Steinwachs, Ingo Burtscher, Philippe Schmitt-Kopplin, Matthias Heinig, Martin Elsner, Heiko Lickert, Fabian J. Theis, Siegfried Ussar

**Affiliations:** 1 RG Adipocytes & Metabolism, Institute for Diabetes and Obesity, Helmholtz Center Munich, Neuherberg, Germany; 2 German Center for Diabetes Research (DZD), Neuherberg, Germany; 3 Institute for Diabetes and Obesity, Helmholtz Center Munich, Neuherberg, Germany; 4 Institute for Diabetes and Cancer, Helmholtz Center Munich, Neuherberg, Germany; 5 Institute for Computational Biology, Helmholtz Center Munich, Neuherberg, Germany; 6 Institute for Diabetes and Regeneration Research, Helmholtz Center Munich, Neuherberg, Germany; 7 Research Unit Analytical BioGeoChemistry, Department of Environmental Sciences, Helmholtz Center Munich, Neuherberg, Germany; 8 Analytical Chemistry and Water Chemistry, Technical University of Munich, Munich, Germany; 9 Department of Mathematics, Technische Universität München, Garching bei München, Germany; 10 Department of Medicine, Technische Universität München, Munich, Germany

**Keywords:** Mechanisms of disease, Differentiation, Obesity

## Abstract

Adipose tissue expansion, as seen in obesity, is often metabolically detrimental causing insulin resistance and the metabolic syndrome. However, white adipose tissue expansion at early ages is essential to establish a functional metabolism. To understand the differences between adolescent and adult adipose tissue expansion, we studied the cellular composition of the stromal vascular fraction of subcutaneous adipose tissue of two and eight weeks old mice using single cell RNA sequencing. We identified a subset of adolescent preadipocytes expressing the mature white adipocyte marker *Asc-1* that showed a low ability to differentiate into beige adipocytes compared to *Asc-1* negative cells in vitro. Loss of *Asc-1* in subcutaneous preadipocytes resulted in spontaneous differentiation of beige adipocytes in vitro and in vivo. Mechanistically, this was mediated by a function of the amino acid transporter ASC-1 specifically in proliferating preadipocytes involving the intracellular accumulation of the ASC-1 cargo D-serine.

## Abstract (web summary)

Adipose tissue is composed of a variety of cell types, including adipocyte precursor populations, that contribute to adipose tissue function upon differentiation. Here, using scRNA-sequencing of adolescent and adult mouse subcutaneous adipose tissue, the authors identify an Asc-1 positive preadipocyte population that is enriched in adolescent subcutaneous fat and demonstrate that loss of *Asc-1* triggers spontaneous beige adipocyte differentiation.

## Introduction

Increasing evidence in humans and rodents suggests that maintaining adipose tissue function dissociates body fat mass gain from the development of metabolic complications enabling a “fat-fit” phenotype^1–3^. A key element of healthy adipose tissue expansion is de novo differentiation of adipocytes rather than storage of fat in already existing adipocytes^4,5^. However, recent data indicate the existence of multiple white^6–9^ and brown^10,11^ adipocyte subtypes that originate from distinct precursor populations. Thus, the type of de novo differentiating adipocytes could strongly affect the metabolic response of the organism to weight gain, as lipid storage capacity, as well as adipokine production differs between white adipocyte subtypes^6–9^. Previous studies mainly focused on differences in precursor and adipocyte populations in adult mice in the transition from normal weight to obesity and insulin resistance. However, adipose tissues show a dramatic, but healthy expansion in the first postnatal weeks, which is essential for the establishment of a functional metabolism. Moreover, C57Bl/6 mice, among other strains, show a transient peak in the number of thermogenic beige adipocytes ~14 days postpartum, which decreases thereafter, when mice are housed at room temperature^12^. However, these dramatic changes in cellular composition and size of adipose tissues, especially subcutaneous adipose tissue, have not been studied in detail to elucidate novel mechanisms enabling healthy adipose tissue expansion in adults.

Here, we show that single-cell transcriptome profiling by single-cell RNA sequencing (scRNA-seq) of adolescent and adult stromal vascular fraction (SVF) from subcutaneous white adipose tissue of C57Bl/6 mice reveals major differences in adipocyte precursor populations. Among the various populations, we identify a subset of cells expressing the mature adipocyte marker alanine serine cysteine transporter-1 (*Asc-1*), which we show to be enriched in the adolescent SVF and to favor white over beige adipocyte differentiation. Mechanistically, we demonstrate that loss or inhibition of *Asc-1* specifically in proliferating preadipocytes results in spontaneous differentiation into thermogenic beige adipocytes, at least in part due to intracellular accumulation of the NMDA receptor co-agonist D-serine. Thus, the white adipocyte-specific amino acid transporter *Asc-1* plays an important role in regulating cell fate decisions in a subpopulation of proliferating precursor cells. Moreover, our data highlight that understanding the regulatory and cellular differences between adolescent and adult adipose tissues will foster the development of pharmacological approaches maintaining adipose tissue function upon weight gain in adult life.

## Results

### Identification of an *Asc-1*^*+*^ preadipocyte population

**Fig. 1:**
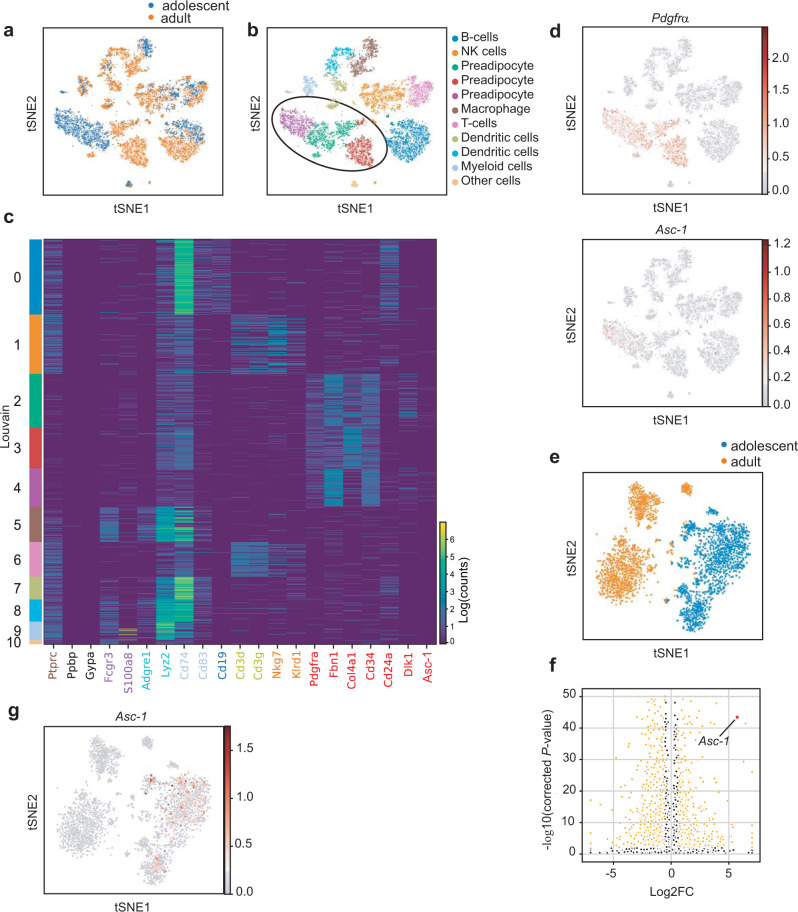
Single-cell sequencing reveals major differences between adolescent and adult preadipocytes. Single-cell sequencing of cells of the subcutaneous stromal vascular fraction from 2- to 8-week-old male C57Bl/6 mice. Cells were pooled from the SCF of seven (2 weeks old) and five (8 weeks old) mice. *T*-stochastic neighborhood embedding (*t*-SNE) of union of adolescent and adult cells of the SCF SVF (*n* = 9344) with mouse age (**a**) and Louvain group (**b**) superimposed. The circle indicates clusters containing preadipocytes. **c** Log1p expression [log1p = log(*X* + 1), where *X* is the expression of gene of interest in particular cell. Note that *X*, the mRNA counts from the alignment, are integer numbers] of different cell type markers in the 11 louvain clusters by cell (*n*_0_ = 1733, *n*_1_ = 1379, *n*_2_ = 1222, *n*_3_ = 948, *n*_4_ = 892, *n*_5_ = 816, *n*_6_ = 799, *n*_7_ = 518, *n*_8_ = 514, *n*_9_ = 421, *n*_10_ = 102). Leukocytes (*Ptprc*), T cells (*CD3d*, *CD3g*), NK cells (*Nkg7*, *Klrd1*), myeloid cells (*Fcgr3*, *S100a8*), macrophages (*Adgre1*, *Lyz2*), dendritic cells (*CD74*, *CD83*), B cells (*CD19*), megakaryocytes (*Ppbp*), erythrocytes (*Gypa*), and preadipocytes (*Pdgfra*, *Fbn1*, *Col4a1*, *CD34*, *CD24a*, *Dlk1*, *Asc-1*). **d** *t*-SNE of union of adolescent and adult cells of the SCF SVF (*n* = 9344) with log1p *Pdgfra* and *Asc-1* expression superimposed. **e** *t*-SNE of preadipocytes from adolescent and adult SCF (*n* = 3,062) colored by age. **f** Volcano plot of comparison between adolescent and adult preadipocytes with differential expression (false discovery rate-corrected *P* value <0.01, Welch’s *t* test) indicated by log1p expression. Each dot is a gene. Black: not differentially expressed, yellow: differentially expressed, red: Asc-1. **g** *t*-SNE of preadipocytes from adolescent and adult SCF (*n* = 3062) colored by *Asc-1* expression.

**Fig. 2:**
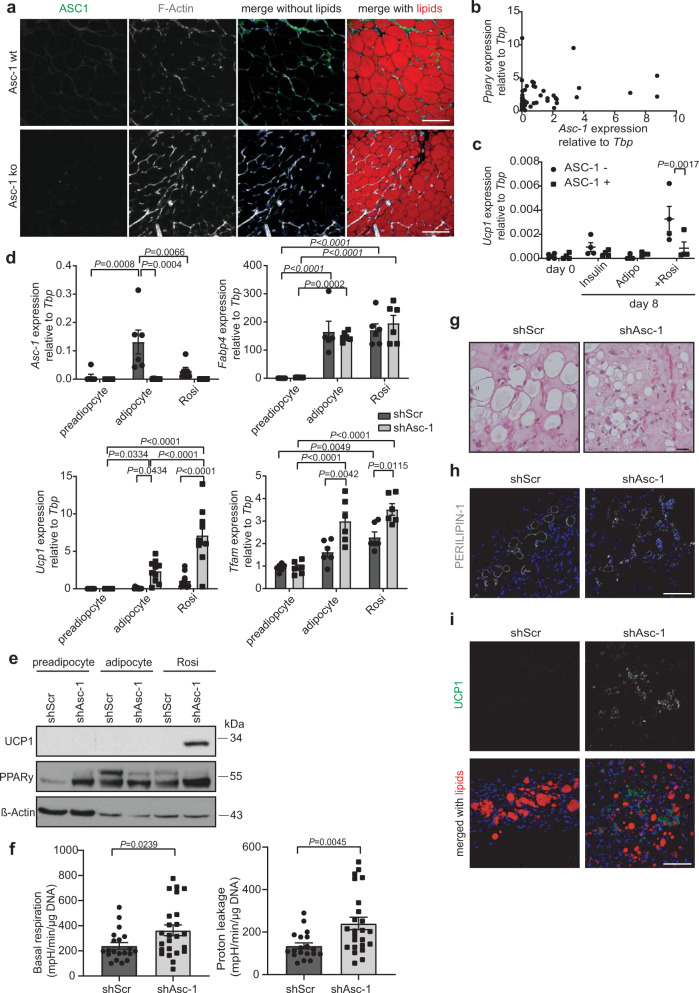
Loss of Asc-1 function induces beiging in white adipocytes. **a** Immunofluorescence staining of ASC-1 (green), F-Actin (gray), and lipids (red) in SCF of 2 week old Asc-1 wt and ko mice (*n* = 1). Size bar 100 µm. **b** Correlation between *Asc-1* and *Ppary* expression in 52 immortalized preadipocyte clones derived from adult subcutaneous SVF. **c** Relative *Ucp1* expression before (day 0) or after 8 days of differentiation of MACS-sorted ASC-1^+^ and ASC-1^−^ preadipocytes with either insulin alone, the normal differentiation protocol (Adipo) or the differentiation protocol plus 1 µM rosiglitazone (*n* = 4). *Asc-1* knockdown (shAsc-1) and control (shScr) immortalized subcutaneous preadipocytes were grown to 100% confluence, and RNA or protein was taken from the day of differentiation start (preadipocyte) and after 8 days of differentiation with (Rosi) or without rosiglitazone (adipocyte). **d** Relative gene expression of *Asc-1* (*n* = 6, only shAsc-1 preadipocyte *n* = 5), *Fabp4* (*n* = 6, only shScr adipocyte *n* = 5), *Ucp1* (*n* = 10, only shScr preadipocyte and shAsc-1 Rosi *n* = 9), and *Tfam* (*n* = 6). **e** Western blot of UCP1 (33 kDa), PPARy (54 kDa), and β-actin (42 kDa). **f** Basal respiration (*n* = 20 shScr and 24 shAsc-1 technical replicates) and proton leakage (*n* = 19 shScr and 24 shAsc-1 technical replicates) calculated from the OCR measured by a Seahorse flux analyzer after differentiation with rosiglitazone, normalized to DNA content (*n* = 3 biologicalreplicates). **g** Balb/c nude mice were injected with shAsc-1 and shScr preadipocytes over the sternum. Histology (*n* = 1; size bar 20 µm) and **h**–**i** immunofluorescence staining of engrafted tissue 6 weeks post transplantation. Green: UCP1 (*n* = 2), red: lipids, blue: DAPI, and gray: PERILIPIN-1 (*n* = 1 shScr-2 shAsc-1). Size bar 100 µm. Statistics were calculated using ordinary two-way ANOVA with Tukey’s multiple comparison post hoc test or two-tailed unpaired Students *t* test (**f**). Data are shown as mean ± SEM.

At 2 weeks, subcutaneous adipose tissue (SCF) of C57Bl/6 mice is rapidly expanding and shows a peak in beige adipocytes^12^, whereas at 8 weeks, SCF is mature. As adipose tissue expansion in adolescent mice is not associated with insulin resistance or inflammation, we aimed to identify cell populations or genes selectively expressed at this age to elucidate mechanisms promoting healthy tissue expansion in adults. To study compositional differences between adolescent (2 weeks old) and adult (8 weeks old) SVF of murine subcutaneous adipose tissue, we performed scRNA-seq of 3915 and 5429 cells, respectively (Fig. 1a). In contrast to previous studies^6–8^, we did not enrich for preadipocytes but sequenced cells from the entire SVF to obtain an unbiased view on the cellular composition of the SVFs at these different ages. A combined view of both datasets identified 11 major Louvain clusters (Fig. 1b), with overlaps between adolescent and adult cells in clusters representing diverse immune cell populations (Fig. 1c). In contrast, we identified three clusters with *Pdgfra*, *Fbn1*, *Col4a1*, *Cd34*, and *Dlk1*, positive preadipocytes^6^ (Fig. 1c, d), in which the cells were clearly separated by age (Fig. 1a). This was even more apparent in *t*-SNE plots focusing on preadipocytes (Fig. 1e). Differential gene expression analysis between adolescent and adult preadipocytes identified 3359 differentially regulated genes with a corrected *P* value <0.01 (Fig. 1f). Interestingly, among the most differentially expressed genes, enriched in the adolescent SVF, we identified the small uncharged amino acid transporter *Asc-1/ Slc7a10*, which we previously described as marker for mature white adipocytes^13^ (Figs. 1f–g and  2a). We identified two major clusters in the adolescent preadipocytes (Supplementary Fig. 1a), one of which is enriched in *Asc-1*^+^ cells (Supplementary Fig. 1b, c; *P* value of 1.41E−07, *t* test), which we used to compare gene expression between *Asc-1* enriched and *Asc-1* low cell populations. Differential gene expression analysis revealed 2442 genes and gene set enrichment analysis indicated that the differences between both clusters are mainly related to developmental processes (Supplementary Tables 1 and 2). Serine racemase (SRR), the enzyme generating the NMDAR co-agonist D-serine that is transported by ASC-1 was not differentially expressed between these two clusters (Supplementary Fig. 1b, c). Thus, we identified a unique subpopulation of precursor cells high for the stem cell marker *Cd34* and the marker for fibro-inflammatory stromal cells *Dpp4*, but low for the classical preadipocyte marker *Dlk1* and the mature adipocyte marker *Fabp4* that express *Asc-1*, a marker for mature adipocytes (Supplementary Fig. 1b), which was recently identified as interstitial progenitors by Merrick and colleagues^14^.

### *Asc-1* marks cells favoring white adipocyte differentiation

Expression analysis in clones derived from the immortalized SVF of subcutaneous fat of adult mice showed that *Asc-1*^*+*^ cells can also be detected in adults, albeit in lower number compared to adolescent SVF (Fig. 2b). Antibody-based sorting and culturing of ASC-1^+^ and ASC-1^−^ cells confirmed initial stable expression or absence of *Asc-1* in these cells, respectively (Supplementary Fig. 1d). However, further passaging of these cells resulted in re-expression of *Asc-1* in the initially ASC-1^−^ cells (Supplementary Fig. 1e). Differentiation using either the standard differentiation protocol, insulin alone, or differentiation in the presence of rosiglitazone did not reveal differences in *Ppary* expression, as marker for adipogenesis between the two cell populations. In contrast, differentiation in the presence of the PPARy agonist rosiglitazone increased expression of *Ucp1* in initially ASC-1^−^, but not ASC-1^+^ cells (Fig. 2c). Thus, Asc-1 appears to mark a transient precursor state favoring white over beige adipocyte differentiation.

### Knockout of *Asc-1* results in a complex metabolic phenotype

To address the role of *Asc-1* in adipose tissue in vivo, we studied whole body knockout mice for *Asc-1* (Fig. 2a and Supplementary Fig. 2a). ASC-1 transports small uncharged L-amino acids, as well as the NMDA receptor co-agonist D-serine^15–18^. Loss of *Asc-1* results in a severe neurological phenotype and early postnatal lethality within 3 weeks after birth due to reduced glycine levels^19^ and synaptic accumulation of D-serine^18–20^. Thus, we analyzed a potential role of *Asc-1* in adipose tissue within the first 2 weeks postpartum. In addition to the neurological phenotype, *Asc-1* knockout mice show a complex metabolic phenotype with reduced body size and weight, hepatosteatosis, hypoglycemia, and reduced body temperature (Supplementary Fig. 2b–f), the latter most likely due to a strong decrease in *Ucp1* expression in brown fat (Supplementary Fig. 2h). However, these phenotypes are most likely secondary as *Asc-1* is not expressed in BAT, liver, or skeletal muscle^13^. We did not observe differences in gene expression of other thermogenic genes in BAT, despite a reduction in the mitochondrial transcription factor *Tfam*, suggesting reduced mitochondrial mass (Supplementary Fig. 2h). SCF mass was slightly reduced (Supplementary Fig. 2g) and histology revealed reduced adipocyte sizes in SCF and BAT (Supplementary Fig. 2i). We observed an increase in UCP1-positive beige adipocytes in SCF by staining, whereas gene expression and protein levels of UCP1, or other adipogenic and thermogenic genes were not altered (Supplementary Fig. 2i–k). The discrepancy between the stainings and mRNA expression of *Ucp1* is puzzling, and could suggest that similar to BAT the overall pathology of the *Asc-1* knockout reduces thermogenic gene expression in beige adipocytes, while the cells retain some of their UCP1 protein. Thus, the severe neurological defects caused by the loss of Asc-1 in the central nervous system restrict the analysis of its metabolic role in adipose tissue. To this end, we tested if heterozygous loss of *Asc-1* results in a metabolic phenotype, as previously described for various other genes^21^. We analyzed male and female *Asc-1* heterozygous mice fed either a chow or high-fat diet (HFD) for 13 weeks. We did not observe genotype specific differences in body weight gain, body temperature, hepatic lipid accumulation, adipose tissue mass, glucose or insulin tolerance, and thermogenic gene expression in either BAT or SCF (Supplementary Fig. 2l–t), indicating that haploinsufficiency of *Asc-1* does not cause metabolic impairment or changes in white adipose tissue.

### Loss of *Asc-1* induces beige adipocyte differentiation

To elucidate the function of *Asc-1*, we generated cell lines with a stable knockdown of Asc-1, together with cells expressing a scrambled shRNA as control from adult subcutaneous preadipocytes (Fig. 2d and Supplementary Fig. 3a). As previously described^13^, *Asc-1* expression was increased upon adipocyte differentiation. However, the treatment with rosiglitazone significantly reduced *Asc-1* expression in control cells, while increasing *Ucp1* expression (Fig. 2d). Knockdown of *Asc-1* almost completely abolished *Asc-1* mRNA and protein levels (Fig. 2d and Supplementary Fig. 3a). Loss of *Asc-1* did not impair adipocyte differentiation (Fig. 2d and Supplementary Fig. 3b). Conversely, shAsc-1 adipocytes expressed significantly higher *Ucp1* levels compared to shScr cells, especially when differentiated with rosiglitazone (Fig. 2d), which was also confirmed on protein levels (Fig. 2e). Similar expression changes were observed for other brown/beige adipocyte markers, such as *P2rx5* and *Pgc1α*, while expression of *Prdm16* and *Pat2* were slightly but not significantly increased in the shAsc-1 compared to control cells (Supplementary Fig. 3b). In line with the increased expression of thermogenic genes in shAsc-1 cells, *Tfam* expression (Fig. 2d), as well as protein content of complexes 1 and 4 of the mitochondrial respiratory chain were increased, especially in rosiglitazone-treated shAsc-1 cells (Supplementary Fig. 3c). Seahorse extracellular flux measurements confirmed increased mitochondrial basal respiration and proton leakage (Fig. 2f) in shAsc-1 cells compared to controls, providing evidence that loss of *Asc-1* results in the formation of functional beige adipocytes. To assess the ability of *Asc-1*-deficient cells to form beige adipocytes in vivo, we injected shAsc-1 and shScr cells subcutaneously on top of the sternum of Balb/c nude mice. However, due to specifications from our animal protocol, we had to house the mice at thermoneutrality, which usually reduces the formation of beige adipocytes. Histology and PERILIPIN-1 staining of engrafted shAsc-1 cells showed smaller and multilocular lipid droplet formation compared to shScr, which showed bigger and predominantly unilocular lipids (Fig. 2g–h). Furthermore, in line with in vitro findings, we observed cells expressing UCP1 in engrafted tissue of shAsc-1, which we did not observe in shScr transplants, further demonstrating that Asc-1 regulates white versus beige adipocyte identity (Fig. 2i).

### *Asc-1* overexpression blunts brown adipocyte *Ucp1* expression

**Fig. 3:**
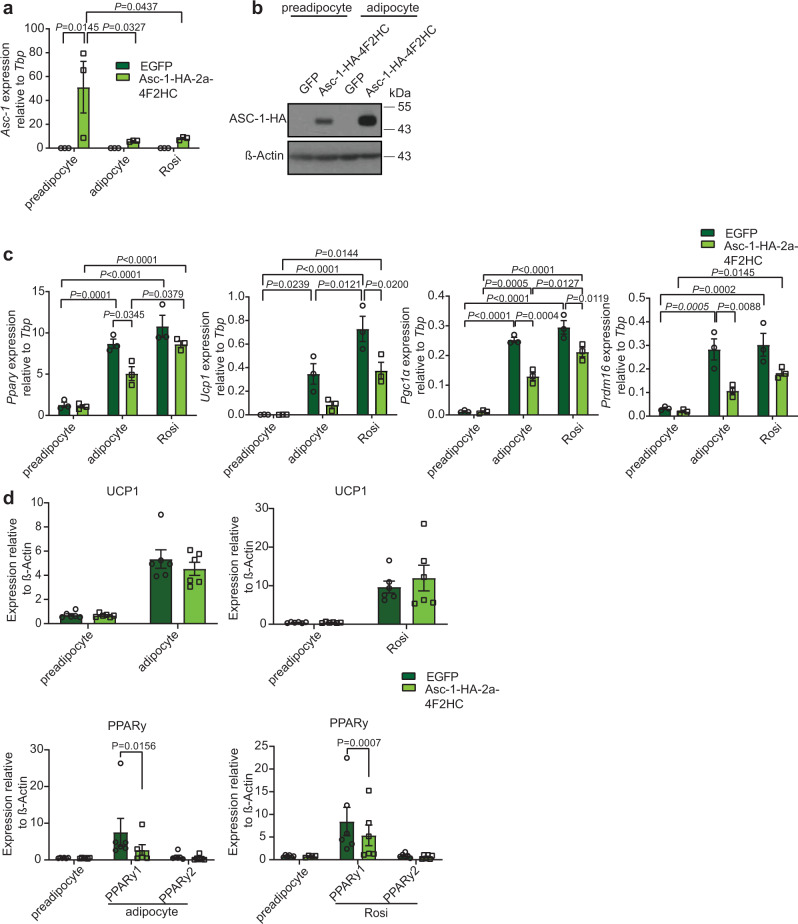
Overexpression of Asc-1 in brown (pre-)adipocytes suppresses thermogenic gene expression. **a** Asc-1-HA-2a-4F2HC or EGFP overexpressing brown preadipocytes were grown to 100% confluence, and RNA or protein was collected from the day of differentiation start (preadipocyte), and after 8 days of differentiation with (Rosi) or without rosiglitazone (adipocyte) and relative gene expression of *Asc-1* measured (*n* = 3). **b** Western blot of ASC-1-HA (45 kDa) and β-actin (42 kDa; *n* = 2). **c** Relative gene expression of *Ppary* (*n* = 3), *Ucp1* (*n* = 3), *Pgc1α* (*n* = 3), and *Prdm16* (*n* = 3). **d** Quantification of PPARy1 and 2 and UCP1 (33 kDa) protein levels relative to β-actin (42 kDa; *n* = 6, only preadipocyte in Ppary blot of Rosi *n* = 4). The same preadipocyte samples were used as controls for the different differentiation conditions. Statistics were calculated using two-way ANOVA with Tukey’s or Sidak’s (for **a**, Pgc1α in **c**, and Ucp1 and Ppary1–2 for adipocyte in **d**) or two-way ANOVA mixed-effects analysis (Ppary1–2 for Rosi in **d**) multiple comparison post hoc test. Data are shown as mean ± SEM.

Next, we tested if *Asc-1* overexpression could suppress thermogenic gene expression in brown adipocytes. We established a cell line stably overexpressing HA-tagged Asc-1 together with its co-receptor 4F2hc^22^ in immortalized brown preadipocytes (Fig. 3a, b). Immunostainings of brown preadipocytes and adipocytes showed, that ASC-1 localized to the plasma membrane in mature adipocytes, and to non-actin containing structures in preadipocytes (Supplementary Fig. 3d). Overexpression of *Asc-1* had no effect on brown adipocyte differentiation, when differentiated in the presence of rosiglitazone (Fig. 3c), albeit *Ppary*, *Fabp4*, and *Adiponectin* mRNA levels were reduced upon differentiation without rosiglitazone and PPARy1, but not PPARy 2, protein levels in general. Importantly, overexpression of Asc-1 resulted in decreased expression of *Ucp1*, *Pgc1α*, *Prdm16*, *Pat2*, and a minor nonsignificant reduction in *Tfam* expression compared to GFP expressing control cells (Fig. 3c, d and Supplementary Fig. 3e) in brown adipocytes differentiated with or without rosiglitazone. Hence, *Asc-1* actively suppresses brown/beige adipocyte-specific gene expression and loss of *Asc-1* in white preadipocytes promotes differentiation of beige adipocytes.

### Loss of Asc-1 primes preadipocytes for beiging

**Fig. 4:**
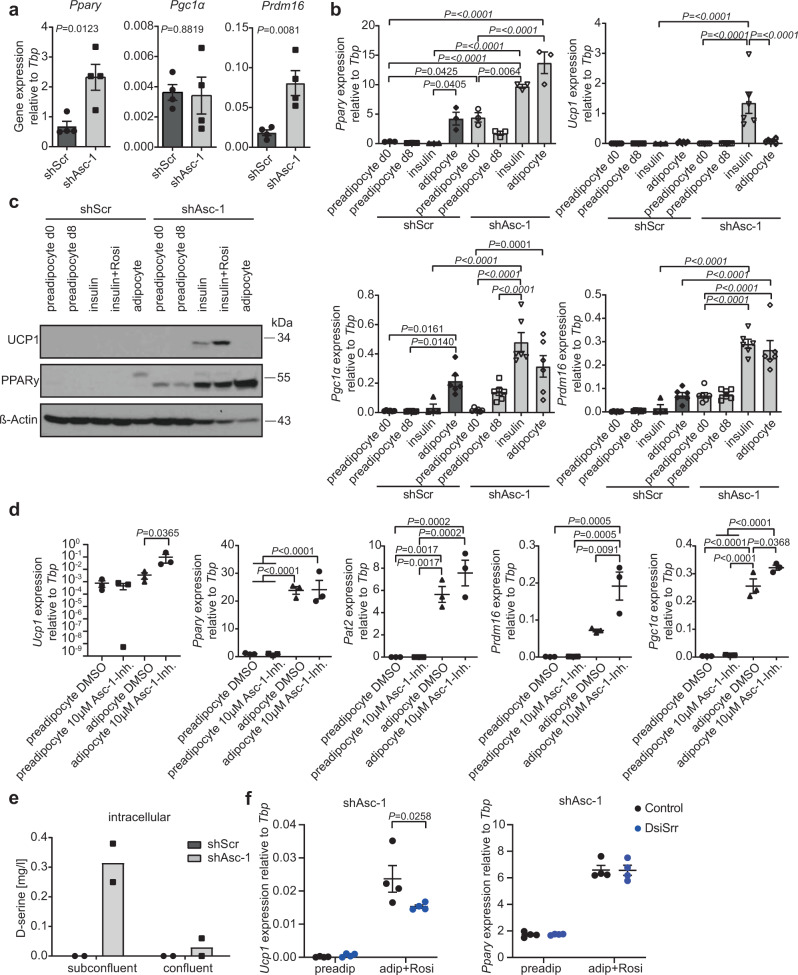
Asc-1 regulates white versus beige adipocyte lineage commitment in a subset of proliferating precursors. **a** *Ppary*, *Pgc1α*, and *Prdm16* gene expression of subconfluent proliferating shAsc-1 and shScr preadipocytes (*n* = 4). **b** Preadipocytes were grown to 100% confluence and differentiation was induced. RNA was taken from the day of differentiation start (preadipocyte d0) and after 8 days (preadipocyte d8), as well as after 8 days of insulin treatment (insulin), insulin and rosiglitazone treatment (insulin + rosi), or differentiation (adipocyte). Relative gene expression of *Ppary* (*n* = 3), *Ucp1* (*n* = 6; for shScr insulin *n* = 3), *Pgc1α* (*n* = 6; for shScr insulin *n* = 4), and *Prdm16* (*n* = 6; for shScr insulin *n* = 4). **c** Western blot of UCP1 (33 kDa), PPARy (54 kDa), and β-actin (42 kDa; *n* = 1). **d** shScr preadipoctyes were plated in presence of 10 µM Asc-1 inhibitor (BMS-466442) or DMSO as control. After 2 days, when cells reached 100% confluency, differentiation was induced with Asc-1 inhibitor or DMSO. Relative gene expression of *Ucp1* (*n* = 3; one-way ANOVA performed with log transformed data), *Ppary* (*n* = 3), *Pat2* (*n* = 3), *Prdm16* (*n* = 3), and *Pgc1α* (*n* = 3). **e** Intracellular D-serine levels of subconfluent and confluent preadipocytes (*n* = 2). **f** Srr was knocked down using DsiSrr in proliferating shAsc-1 preadipocytes and differentiated upon confluency. *Ucp1* and *Ppary* mRNA levels of shAsc-1 cells at day of induction (preadip) or cells differentiated following the regular differentiation protocol, including rosiglitazone (adip + Rosi). A scrambled DsiRNA was used as negative Control (Control) for knockdown (*n* = 4). Statistics were calculated using two-tailed *t* test (**a**) or ordinary one-way or two-way (**f**) ANOVA with Tukey’s or Sidak’s multiple comparison post hoc test. Data are shown as mean ± SEM.

We then tested if loss of *Asc-1* in preadipocytes primes these cells to a beige adipocyte fate prior to differentiation. We observed significantly increased expression of *Ppary* and *Prdm16*, but not *Pgc1α* in subconfluent proliferating shAsc-1 preadipocytes compared to control cells (Fig. 4a). As *Ppary* is the key regulator of adipocyte differentiation and together with Prdm16 induces beiging^23^, we tested if shAsc-1 preadipocytes can spontaneously differentiate into beige adipocytes. Preadipocytes were grown to confluency and incubated for eight additional days with either normal growth medium, growth medium with 100 nM insulin, or differentiated following our regular differentiation protocol (Fig. 4b). shAsc-1 preadipocytes already expressed *Ppary* to the same extend as fully differentiated control adipocytes, and its expression was further increased upon insulin supplementation reaching the same level as following the differentiation protocol (Fig. 4b). This strongly suggested spontaneous differentiation of shAsc-1 cells, which we confirmed by live cell imaging (Supplementary Movie 1). Moreover, we observed proliferating lipid laden shAsc-1 adipocytes (Supplementary Movie 1), which is extremely surprising as differentiating adipocytes are thought to be post mitotic^24^. *Ucp1* mRNA and protein levels were high in shAsc-1 adipocytes differentiated with insulin and further increased in insulin plus rosiglitazone-treated cells (Fig. 4b, c). Similarly, insulin was sufficient to increase *Pgc1α* and *Prdm16* expression (Fig. 4b) in shAsc-1 adipocytes. Together, these data show that loss of *Asc-1* results in spontaneous differentiation of a subset of preadipocytes into beige adipocytes, supporting the notion that the presence of *Asc-1* suppresses beige adipocyte lineage commitment. Moreover, these data explain why insulin alone results in a more pronounced expression of *Ucp1* and other thermogenic genes, as *Asc-1* negative cells do not differentiate spontaneously, while cells that had expressed *Asc-1* but were depleted for it by RNAi, spontaneously differentiate into beige adipocytes and proliferate to take over the culture.

### Intracellular D-serine regulates beiging

Based on these data, we tested if the inhibition of Asc-1 using BMS-466442 (ref. ^25^) in proliferating preadipocytes could induce beiging. Preadipocytes were treated with the Asc-1 inhibitor 2 days prior to reaching confluency and throughout differentiation (Fig. 4d). Inhibition of ASC-1 did not result in immediate changes in gene expression in preadipocytes. However, when ASC-1 activity was inhibited in proliferating preadipocytes, these cells differentiated into beige adipocytes, as shown by increased expression of *Ucp1*, *Prdm16*, and *Pgc1α*. Inhibition of ASC-1 had no effect on the differentiation per se as evidenced by equal expression of *Ppary* (Fig. 4d). Importantly, ASC-1 inhibition from the day of induction onward did not alter thermogenic gene expression (Supplementary Fig. 4a). Thus, these data demonstrate that ASC-1 transport activity in proliferating preadipocytes is necessary to suppress the differentiation of a subset of cells into beige adipocytes.

ASC-1 bidirectionally transports small, neutral L-amino acids^15,22^, as well as D-serine and loss of *Asc-1* altered intracellular L-amino acid levels of both preadipocytes and adipocytes, with little changes in the medium (Supplementary Fig. 4b). However, depletion of the ASC-1 cargos alanine, serine, glycine and glutamine from the cell culture medium did not alter expression of *Ppary, Ucp1* or *Pgc1α* in either control or shAsc-1 cells (Supplementary Fig. 4c). In contrast to the overall reduction in intracellular L-amino acid levels, we observed an intracellular accumulation of D-serine in both; subconfluent, proliferating (Fig. 4e), as well as confluent shAsc-1 cells, which resulted in reduced extracellular D-serine concentrations (Supplementary Fig. 4d). Cell culture medium can contain up to 0.4 mg/l D-serine, thus the intracellular and extracellular levels could result from de novo synthesis by SRR or uptake form the medium. However, the accumulation of intracellular D-serine suggests an inhibition of the export of de novo synthesized D-serine upon loss of *Asc-1*. To test if increasing extracellular D-serine levels could alter brown/beige adipocyte gene expression, we differentiated brown control and *Asc-1* overexpressing brown preadipocytes in the presence of different D-serine concentrations. However, this had no effect on the expression of either *Ppary* or *Ucp1* (Supplementary Fig. 4e). To fully explore the role of elevated extracellular D-serine levels on beige adipocyte differentiation in vivo, we analyzed adipose samples from mice with chronically elevated systemic D-serine levels using supplementation of the drinking water^26^. Chronic supplementation with D-serine raised D-serine tissue levels in both SCF and BAT (Supplementary Fig. 4f). We did not observe any changes in *Ucp1* expression in white adipose tissues on chow or HFD (Supplementary Fig. 4g), suggesting also in vivo that the observed effects on white adipocyte beiging are most likely mediated through an intracellular accumulation of D-serine. However, we observed a reduction in *Ucp1* expression in BAT of CD-fed D-serine supplemented mice (Supplementary Fig. 4g). NMR-based metabolomics of confluent control and shAsc-1 preadipocytes confirmed the depletion of intracellular L-amino acid levels and revealed accumulation of lipids, myo-inositol, choline, and phosphocholine indicative of adipogenesis^27^; however, it did not identify metabolites that could explain how D-serine mediates the reprogramming of the preadipocytes toward beige adipogenesis (Supplementary Fig. 4h and Supplementary Table 3). Nevertheless, knockdown of serine racemase (*Srr*), converting L-serine to D-serine, significantly reduced *Ucp1* expression in shAsc-1 adipocytes when the knockdown was performed in proliferating preadipocytes (2 days prior to the induction of differentiation). The knockdown of *Srr* had no effect on adipocyte differentiation (Fig. 4f and Supplementary Fig. 4i). In line with the overall very low levels of D-serine in control cells (Fig. 4e), knockdown of *Srr* did not alter *Ucp1* mRNA levels in shScr cells compared to controls (Supplementary Fig. 4i). These data suggest that the accumulation of intracellular D-serine due to knockdown of *Asc-1* contributes to the beiging of a subset of white subcutaneous preadipocytes. However, we cannot exclude the possibility that other ASC-1 cargos that remain to be identified, can also contribute to the observed beiging.

## Discussion

Utilizing an unbiased view on the differences between adolescent and adult stromal vascular cells, we observe major differences in gene expression of preadipocyte populations. This allowed us to identify a previously undescribed transient *Asc-1*^*+*^ precursor population, differentiating into white and not beige adipocytes. Additional studies are required to identify and describe additional differences between these two states, especially with respect to adipose tissue expansion and triacylglyceride storage capacity that could help understanding differences in the developmental versus pathological adipose tissue expansion. Focusing on the role of *Asc-1*, we demonstrate that *Asc-1* regulates beige versus white adipocyte lineage decision. One possible mode of action of ASC-1 is the export of D-serine, intracellularly produced by SRR. Consequently, we show that the loss of *Asc-1* results in intracellular accumulation of D-serine and knockdown of *Srr*, in *Asc-1* knockdown cells, reduced the expression of *Ucp1*. However, ASC-1 also transports several other amino acids, of which reduced glycine levels have been shown to be responsible for the severe neurological defects in *Asc-1* knockout animals^19^. Thus, we cannot exclude or verify that other amino acids or metabolites could also play a role in the role of *Asc-1* in determining beige versus white adipocyte differentiation. In addition to the observed phenotypes, the presence of the machinery to produce and transport D-serine, and its only known function as co-agonist of the NMDA receptors, raises the question on the function of NMDARs in adipose tissues.

Taken together, our data highlight the complexity of adipose lineage decisions and the plasticity of adipose tissue to respond to various environmental cues to shape tissue architecture, cellular composition, and ultimately function. While most work to date focuses on understanding the transition from lean metabolically intact to obese and metabolically impaired individuals, we propose to utilize non-pathological developmental programs, such as initial adipose tissue expansion to elucidate previously unrecognized pathways enabling healthy tissue remodeling in the adult state.

## Methods

### Mouse models

Heterozygous Asc-1 knockout mice were purchased from Deltagen (B6;129P2-Slc7a10^tm1Dgen^/H), and bred and housed under 45–65% humidity and a 12 h light:12 h dark cycle at ambient temperature 22 ± 2 °C. Whole body knockout mice and littermate controls were sacrificed 1 or 2 weeks after birth. For the comparison of older mice, Asc-1 heterozygous mice and wild-type littermates were fed a standard laboratory chow diet (Altromin 1314) or a 58% HFD (Research Diets D12331) ad libitum from an age of 8 weeks on.

For D-serine supplementation, male C57BL/6N-Rj mice were purchased from Janvier at an age of 3 weeks. Mice received either normal water or water supplemented with 1% D-serine ad libitum.

For preadipocyte transplantation, shAsc-1 and shScr preadipocytes were collected in CellStripper (Corning) buffer, washed one time with DPBS and pelleted at 300 × *g* for 5 min. A total of 1 × 10^6^ cells were resuspended in 100 µl of 50% pure DMEM (Dulbecco’s Modified Eagle Medium) and 50% Matrigel (Biosciences). Male Balb/c nude mice were subcutaneously injected on top of the sternum with a 25 G syringe. Transplanted mice were kept at thermoneutrality and single housed for 5–6 weeks. Engrafted tissues were removed and fixed in 4% PFA. Animal experiments were conducted in accordance with the German animal welfare law and performed with permission, and in accordance with all relevant guidelines and regulations of the district government of Upper Bavaria (Bavaria, Germany), ROB-55.2Vet-2532.Vet_02-14-33, ROB-55.2-2532.Vet_02-16-52, ROB-55.2-2532.Vet_02-18-188, and ROB-55.2-2532.Vet_02-17-125.

### Single-cell sequencing

Single-cell libraries were generated using the ChromiumTM Single-cell 3′ library and gel bead kit v2 (PN #120237) from 10× Genomics. Briefly, live cells from the SVF of subcutaneous adipose tissue of 2- and 8-week-old mice were obtained by flow cytometry following dead cell exclusion using 7-AAD. Afterward, cells were loaded onto a channel of the 10× chip to produce Gel Bead-in-Emulsions. This underwent reverse transcription to barcode RNA before cleanup and cDNA amplification followed by enzymatic fragmentation and 5′ adaptor and sample index attachment. Libraries were sequenced on the HiSeq4000 (Illumina) with 150 bp paired-end sequencing of read2 and 50,000 reads per cell. The count matrices are shown as Supplementary Data 1 and 2, and the analysis pipeline as Supplementary Data 3.

### Computational analysis of the single-cell RNA-seq data

For the analysis, an indexed mm10 reference genome was build based on the GRCm38 assembly and genome annotation release 94 from Ensembl. For the alignment, QC, the estimation of valid barcodes, and creating the count matrices, the Cell Ranger pipeline (version 2.1.1, from 10× Genomics, https://support.10xgenomics.com) was run with the command “cellranger count” with standard parameters, except that the number of expected cells was set to 8000 (adolescent sample) and 14,000 (adult sample), respectively, the chemistry was set to “SC3Pv2”. An anndata object was created using the python package Scanpy^28^ (version 1.4.4).

#### Cell type assignment

The workflow was performed in scanpy version 1.4.4 (ref. ^28^). Cells were filtered based on a minimum UMI count of 500. The remaining cell vectors were normalized to sum a total count of 1e4 by linear scaling. We performed a PCA, took the top 50 PCs to compute a *t*-SNE and a *k*-nearest neighbor (*k*NN) graph (*k* = 100, method = umap). We computed a louvain clustering (resolution = 1, flavor = vtraag) based on the *k*NN graph, we call this clustering louvain_1 in the following. We assigned cell types to clusters based on marker gene expression by cluster.

#### Heterogeneity analysis of preadipocytes

The workflow was performed in scanpy version 1.4.4 (ref. ^28^). We selected the louvain clusters that correspond to preadipocytes from the overall clustering (louvain 1) and separately processed these cells by first filtering for a minimum UMI count of 500, then normalizing to 1e4 counts, then log(*X* + 1) transforming the data followed by PCA with 50 PCs. Again, we computed *t*-SNE and a *k*NN graph (*k* = 100, method = umap) based on the PC space. We computed a louvain clustering (resolution = 1, flavor = vtraag) based on the *k*NN graph, we call this clustering louvain_2 in the following. Similarly, we also computed *t*-SNE and louvain clusterings for preadipocytes from adolescent mice only (louvain_3 with resolution = 1 and a coarser clustering louvain_4 with resolution = 0.5) and from old mice only louvain_5 with resolution = 1).

#### Differential expression analysis

We performed differential expression analysis between two groups with Welch’s *t* tests and performed multiple testing correction with the Benjamini–Hochberg correction.

#### Gene set enrichment analysis

Gene set enrichment analysis was performed with the GSEA^29^ based on genes with a false discovery-corrected *P* value for differential expression lower than 0.01.

### Glucose and insulin tolerance tests

Glucose tolerance tests and insulin tolerance tests were carried out in mice fasted for 4 h. A total of 2 g/kg glucose (20% glucose solution; Braun) or 0.75 U insulin/kg BW (Actrapaid® PenFill® Novo Nordisk) were injected intraperitoneally, and glucose concentrations measured in blood collected from the tail before and 15, 30, 60, 90, and 120 min after the injection using a FreeStyle Freedom Lite glucometer (Abbott).

### Serum and liver metabolites

Serum insulin levels were determined using the Mouse ultrasensitive insulin ELISA kit (Alpco). Liver triglyceride content was measured with the Triglyceride Quantification Colorimetric/Fluorometric Kit (BioVision).

### Histology and imaging

Tissues were fixed in 4% PFA/PBS overnight, paraffin embedded, and sectioned at 2 µm. Sections were stained with hematoxylin and eosin. Immunohistochemistry for UCP1 (anti-UCP1; (Abcam, ab10983; 1:500)) was performed using a VECTASTAIN ABC HRP Kit (Vector Laboratories), following the manufacturer’s instructions with a Mayer’s Hemalum counterstain. For immunofluorescence, a secondary antibody (anti-rabbit Alexa 488 (Life Technologies; 1:500)) was used. Immunofluorescence stainings of UCP1 (Abcam; 1:250) and PERILIPIN-1 (Cell Signalling; 1:200) for transplants were performed on 30 µm cryo-sections after blocking in 3% BSA, and permeabilizing with 1% Triton-X100 and incubated with secondary antibody (anti-rabbit Alexa 594 (Jackson ImmunoResearch; 1:400)). For the determination of ASC-1, tissues were embedded in agarose after fixation and sectioned using a vibratome at 70–100 µm. Sections were stained with an anti-Asc-1 monoclonal antibody (clone 10A11 produced in rat against the peptide MRRDSDMASHIQQPGGHGNPGPAPS of murine Asc-1) and secondary antibody (anti-rat 488 (Life Technologies; 1:400)) was incubated together with Phalloidin Alexa 647 (Life Technologies, 1:100) to stain F-actin and Bodipy 493/503 (1:1000; Life Technologies D3922) to visualize lipids.

To investigate the localization of Asc-1-HA in vitro, cells were fixed 10 min in 10% formalin, washed three times with PBS, and blocked for 1 h in 3% BSA/PBS. Primary antibody was incubated overnight at 4 °C (anti-HA-tag; Roche, 1:100) and secondary antibody (anti-rat Alexa 488 (Life Technologies; 1:500)) mixed with Alexa Fluor® 546 Phalloidin (Life Technologies; 1:100) to stain the actin cytoskeleton and DAPI (1 µg/ml; Merck Millipore) was added for 1 h. Cells were mounted in fluorescent mounting medium (DAKO, S3023) and images were acquired, using a Zeiss scope A1 and a Leica SP5 confocal microscope.

#### Live cell imaging

Subconfluent shAsc-1 cells were cultured in regular growth medium supplemented with 170 nM insulin. Imaging was initiated 4 h post seeding and continued for 3 days. The live cell imaging was performed with the Zeiss Cell Observer microscope using the Zen 2.6 program. Pictures were taken in 10 min intervals.

### Cell models and cell culture

Murine subcutaneous or brown adipose tissue was dissected and digested for 30 min at 37 °C in DMEM (DMEM high glucose + GlutaMAX, Gibco) containing 1 mg/ml collagenase type IV (Gibco) and 1% BSA (Albumin fraction V, Roth). The digestion was stopped by washing with PBS (Gibco) containing 1% BSA. The cells were centrifuged for 5 min at 800 × *g* and washed with growth medium (DMEM, DMEM + GlutaMAX + high glucose, Gibco) containing 10% fetal bovine serum (Gibco) and 1% penicillin/streptomycin (Gibco). After a second centrifugation step, cells were resuspended in growth medium and seeded on six-well plates. The cells were immortalized using an ecotropic SV40 large T retrovirus produced in HEK 293 T cells. Immortalized cells were plated on six-well plates, infected with a lentivirus containing a pLKO.1 vector with a scrambled (scr), or an anti-Asc-1 shRNA (TRCN0000079470: CCGGGCCTGTCTTTCCCAACTGTATCTCGAGATACAGTTGGGAAAGACAGGCTTTTTG) or a pCDH-CMV-MCS-EF1-puro vector with a GFP or Asc-1-HA-2a-4F2hc overexpression. After 24 h, the cells were selected with growth medium containing 2.5 µg/ml puromycin (Biomol). This medium also was used for all cell culture experiments. The preadipocytes were grown in cell culture plates up to 100% confluence and the differentiation of subcutaneous preadipocytes was induced with 0.5 mM 3-isobuthyl-1-methylxanthin (IBMX, Sigma), 1 µM dexamethasone (Merck Millipore), and 170 nM insulin (Sigma) in the growth medium. After 2 days, the medium was changed to continuum medium containing only 170 nM insulin. Differentiation of brown preadipocytes was induced with 0.5 mM IBMX, 5 µM dexamethasone, 0.125 mM indomethacine (Santa Cruz Biotechnology), 1 nM triiodothyronine (T3, Merck Millipore), and 100 nM insulin. After 2 days, the medium was changed to medium containing only 100 nM insulin and 1 nM T3. The medium was changed every 2 days until day 8. In addition, some cells were supplemented with 1 µM rosiglitazone (Santa Cruz Biotechnology) during the first 2 days of differentiation, with different concentrations of the Asc-1-inhibitor BMS-466442 (Aobious), diluted in DMSO, or D-serine from the day of seeding to end of the differentiation phase. For the differentiation in amino acid-depleted medium, amino acid free medium (Genaxxon) was supplemented with 10% FBS, 1% penicillin/streptomycin, and amino acids in the same concentration then the DMEM used for the other cell culture experiments.

Srr knockdown was performed using a reverse transfection method^30^. Briefly, cells were seeded in 12-well plates together with the transfection mix containing 50 nM DsiSrr (mm.Ri.Srr.13.3, IDT) or negative control (IDT; Control) and Lipofectamine RNAiMAX (Invitrogen) in OptiMEM (Gibco). After 24 h, transfected shScr and shAsc-1 cells were reseeded, grown to confluency for 48 h and differentiated with 0.5 mM IBMX, 5 µM dexamethasone, and 100 nM insulin supplemented with 1 µM rosiglitazone. After 2 days, the medium was changed to culture medium containing only 100 nM insulin. The medium was changed every 2 days until day 8.

### MACS sorting of ASC-1^+^ preadipocytes

Primary or immortalized preadipoctyes were collected in CellStripper (Corning) buffer, pelleted at 500 × *g* at 4 °C for 5 min and blocked in MACS Running Buffer (Milteny Biotec) for 10 min on ice. Cells were then incubated with the ASC-1 antibody 10A11 cell culture supernatant at a dilution of 1:2 for 30 min. Cells were incubated with 25 µl protein G microbeads (Milteny Biotec) for 30 min. MS columns (Milteny Biotech) were equilibrated with MACS Running Buffer. The flow through fraction was collected as negative control. The column was washed three times with 200 µl ml MACS running buffer, removed from the magnetic field and retained cells were eluted with 500 µl ml MACS running buffer. Both flow through and eluted cells were plated on 24-well plates in growth medium and cultured for further experiments.

### pCDH-CMV-MCS-EF1-puro-Asc-1-HA-4F2hc cloning

Murine Asc-1 was PCR amplified by PCR introducing a 5′ EcoRI and a 3′ NotI omitting the stop codon and cloned into a pCDH-CMV-MCS-EF1-puro lentiviral vector deficient for XbaI and NheI in the MCS. A HA-tag was inserted at the 3′ end using the lightning site mutagenesis kit (Agilent) and correctness of the construct was confirmed by sequencing. The 4F2hc (Slc3a2) cDNA was PCR amplified introducing a 5′ NheI and a 3′ NotI and EcoRI site. The product was cloned into a pULTRA plasmid 3′ of the T2A element. The T2A-4F2hc fragment was PCR amplified introducing a 5′ BamHI site and cloned into pCDH-CMV-MCS-EF1-puro–Asc-1-HA generating a pCDH-CMV-MCS-EF1-puro-NheI-EcoRI-Asc-1-HA-BamHI-2a-NheI.4F2hc-Stop-NotI plasmid.

### RNA isolation, reverse transcription, and real-time PCR

For the determination of gene expression, RNA was extracted from tissue or cell culture samples using the RNeasy mini kit (Quiagen) and cDNA synthesis (0.5–1 µg total RNA, High Capacity cDNA Reverse Transcription Kit, Applied Biosystems) was performed according to the manufacturers’ instructions. qPCR was performed in a CFX384 Touch (BioRad), using 300 nM forward and reverse primers and iTaq Universal SYBR Green supermix (BioRad) and analyzed using the BioRad CFX Manager 3.1 Software. The target gene expression was normalized on TATA box-binding protein (TBP) expression. Samples without Ct value were set to 50, given that TBP was expressed. The primer sequences are shown in Supplementary Table 4.

### Protein extraction and western blot

Protein was extracted with IP lysis buffer (#87787; Thermo Fisher Scientific) or RIPA buffer (50 mM Tris, 150 mM sodium chloride, 1 mM ethylenediaminetetraacetic acid, 1% Triton-X100, pH 7.4) containing 0.1% SDS, 1% protease-inhibitor (Sigma-Aldrich), 1% phosphatase-inhibitor cocktail II (Sigma-Aldrich), and 1% phosphatase-inhibitor cocktail III (Sigma-Aldrich). Cells were lyzed, incubated on ice for 10 min and centrifuged for 10 min at 14,000 × *g* at 4 °C. The supernatant was collected and protein concentration was determined using the Pierce BCA protein assay kit (Thermo Fischer Scientific). Samples were mixed with 4× sample buffer (Life technologies) containing 2.5% β-mercaptoethanol (Carl Roth), boiled for 10 min at 70 °C, and loaded on an SDS gel. After transfer on a PVDF membrane (0.22 µm or 0.45 µm, Roth), the membrane was blocked in 5% BSA or skim milk (Carl Roth)/TBST for 1 h and incubated in primary antibody (UCP1 (for brown (pre-) adipocytes: 1:15,000 described in ref. ^31^, and for subcutaneous (pre-) adipocytes: cell signaling; 1:1000), PPARy (Cell Signaling Technology; 1:2000), Total OxPhos Complex Kit (Invitrogen; 1:2000)), ASC-1 (10A11; 1:10), and GAPDH (Calbiochem; 1:1000) at 4 °C overnight. After washing, secondary antibodies (anti-rabbit HRP (Cell Signaling Technology or Invitrogen; 1:10,000), anti-mouse HRP (Cell Signaling Technology or Santa Cruz; 1:10,000), and anti-rat IgG1 HRP (monoclonal core facility Helmholtz Munich; 1:1000)) were incubated for 1 h. The HRP-linked ß-actin (Santa Cruz Biotechnology; 1:10.000) and HA-tag antibodies (Cell Signaling; 1:1000) were incubated 1 h at RT. After washing, ECL (Merck Millipore) was added to the membranes and the signal was detected either with films (ECL high performance chemiluminescence film (GE Healthcare Life Sciences),CEA® RP New Medical X-Ray Screen Film Blue Sensitive (CEA Group), or Chemidoc MP (BioRad)). Quantifications were performed using the BioRad ImageLab Software.

### Seahorse analysis

Cells were seeded in a Seahorse 96-well plate, induced for differentiation with rosiglitazone according to the protocol above and the oxygen consumption rate (OCR) was measured at day 8 of differentiation with a XF96 Extracellular Flux analyzer (Seahorse). Cells were equilibrated at 37 °C in XF Assay Medium Modified DMEM (Seahorse Bioscience) supplemented with 5 mM sodium pyruvate (Gibco) 1 h prior to the measurement. All compounds were diluted in assay medium and loaded in the equilibrated cartridge ports: (A) 20 µg/ml oligomycin (Merck), (B) 20 µM FCCP (R&D systems), (C) 50 µM rotenone and 20 µM antimycin A (Sigma), and (D) 1 M 2-deoxy-D-glucose (Alfa Aesar). Each cycle comprised 3 min mixing, 1 min waiting, and 2 min measuring. For the analysis, the mean of cycles 4 and 5 presents basal respiration (OCR), and the mean of cycles 7 and 8 refers to proton leakage (OCR). Data points with a ROUT *Q* value equal to or >5% were excluded.

### NMR-based metabolomics

Confluent cells were scratched down, centrifuged to remove remaining medium, and directly frozen at −80 °C. Cells were suspended in 1 ml ice-cold methanol and homogenized in a TissueLyser (Qiagen). Methanol was evaporated in a SpeedVac (Christ), metabolites resuspended in NMR buffer (200 µl 25% D_2_O, 0.375 M PO_4_, pH 7.4, 0.025% trimethylsilyltetradeuteropropionic acid) and transferred to 3 mm outer diameter NMR tubes and analyzed, using a cryo-800 MHz NMR spectrometer. NMR spectroscopy was performed as previously described^26^. Normalization of NMR spectra was carried out using the probabilistic quotient normalization method^32^, quantification of selected metabolites was done by integration of their area under the curve (AUC).

### D-serine measurement

D-serine, L-serine, sodium tetraborate, Marfey’s reagent (N_α_-(2,4-dinitro-5-fluorophenyl)-l-alaninamide), hydrochloric acid, and acetone were purchased from Sigma-Aldrich. Acetonitrile was purchased from Thermo Scientific. LC-MS grade water with 0.1% acetic acid was purchased from Fluka.

Cells were homogenized in 1 ml 100% methanol using a Polytron. Cells and medium were dried at 60 °C under a stream of N_2_, and the dried residues were reconstituted in 50 µl MilliQ water. Separation of the serine enantiomers was performed with chemical derivatization using Marfey’s reagent, generating dinitrophenyl-5-l-alanine-d/l-serine diastereomers (DNPA-D/L-serine), which can be separated by reverse-phase chromatography^33^. To this end, 100 µl methanol, 50 µl 0.125 M sodium tetraborate buffer, and 50 µl 1% Marfey’s reagent in acetone were added to the reconstituted sample and heated to 50 °C for 60 min. Derivatization was then stopped by addition of 20 µl 2 N HCl. For standard curve calibration, D- and L-serine standards were prepared at 166, 55, 18, 6, 2, 0.7, 0.2, 0.07, and 0.02 mg/l by serial dilution of a 500 mg/l stock solution and derivatized, as described above.

Chromatographic separation of DNPA-D- and DNPA-L-serine was accomplished on an Agilent HP 1200 HPLC system using a Synergi Hydro-RP (C18) 150 mm × 2.1 mm I.D., 4 µm 80° A particles column (Phenomenex) at 25 °C. Mobile phase A was LC-MS grade water with 0.1% acetic acid and mobile phase B was acetonitrile, which was delivered according to the following gradient profile (min/% mobile phase B): 0.0/5, 1.9/5, 12/20, 20/20, 24/90, and 30/90. The flow rate was 0.2 ml/min and the injection volume was 10 µl.

The HPLC system was coupled to a Q-Trap MS/MS system (Applied Biosystems). Ionization was accomplished by electrospray ionization in the negative ion mode. Mass spectrometry was carried out in the multiple reaction monitoring mode. To determine the MS/MS fragment pattern with the highest intensity, a single analyte standard containing DNPA-D/L-serine was dissolved in a mixture of water and methanol 50:50 (v/v) at a concentration of 1 mg/l. The standard was infused at a flow rate of 200 µl/min for tuning the compound-dependent MS parameters. The infusion experiment was performed using a syringe pump directly connected to the interface. Optimal detection was provided by scanning for the mass pair 356/356. Declustering potential, collision energy, collision cell exit potential, and entrance potential were optimized to −45, −12, −3, and −6 V. The optimized values for the parameters ion spray voltage, nebulizer gas, auxiliary gas, curtain gas, collision gas, and auxiliary gas temperatures were −4500, 40, 35, 20, 3, and 400 °C, respectively. The curtain, collision, turbo, and nebulizer gas was nitrogen generated from pressurized air in a nitrogen generator. To obtain adequate selectivity and sensitivity, the mass spectrometer was set to unit resolution, and the dwell time was 150 ms.

Calibration curves were obtained by plotting the peak area of D/L-serine against their concentration. Peak integration was automatically accomplished via Analyst software (version 1.4.2, AB/MDS Sciex). A least squares regression analysis was used to obtain a linear equation over the range of the calibration. The assay finally produced a standard curve that was linear over a range of 0.02–166 mg/l. Samples were measured in triplicates.

### Amino acid measurement

Cells were homogenized in 1 ml 100% methanol using a Polytron and placed on a column of Dowex 50Wx8 (H^+^ form, 200–400 mesh, 5 × 10 mm). The column was washed twice with 750 ml of water and was developed with 1 ml of 2 M ammonium hydroxide. An aliquot of the eluate was dried under a stream of nitrogen, and the residue was dissolved in 50 ml of dry acetonitrile. A total of 50 ml of N-(tert-butyldimethylsilyl)-N-methyl-trifluoroacetamide containing 1% tert-butyldimethylsilylchlorid (Sigma-Aldrich) was added. The mixture was kept at 70 °C for 30 min. The resulting mixture of tert-butyl-dimethylsilyl derivatives (TBDMS) of amino acids was used for GC/MS analysis without further workup. Gas chromatography/mass spectrometry analysis was performed on a GC-17A Gas Chromatograph (Shimadzu, Duisburg, Germany) equipped with a fused silica capillary column (Equity TM-5; 30 m × 0.25 mm, 0.25 mm film thickness; SUPELCO, Bellafonte, PA) and a QP-5000 mass selective detector (Shimadzu, Duisburg, Germany) working with electron impact ionization at 70 eV. An aliquot (1 µl) of a solution containing TBDMS amino acids was injected in 1:5 split mode at an interface temperature of 260 °C and a helium inlet pressure of 70 kPa. The column was developed at 150 °C for 3 min and then with a temperature gradient of 10 °C/min to a final temperature of 260 °C that was held for 3 min. Data were collected using the Class 5000 software (Shimadzu, Duisburg, Germany). Samples were analyzed three times.

### Statistics

Data are presented as mean ± standard error of the mean (SEM) unless stated differently in the figure legend. Statistical significance was determined by unpaired Student’s *t* test or, for multiple comparisons, using one- or two-way ANOVA, followed by Tukey’s multiple comparison’s test, or as stated in the respective figure legend. Differences reached statistical significance with *P* < 0.05.

Supplementary Fig. 2 (a) n = 11 wt-5 het-7 ko. (h) *n* = 10 wt-5 het-7 ko only Ucp1 9 wt. (j) *n* = 4 wt-5 ko. (k) *n* = 11 wt-5 het-7 ko. (l) *n* = 4 wt male-7 het male-3 wt female-3 het female for CD and *n* = 5 wt male-8 het male-8 wt female-8 het female for HFD feeding. (m) *n* = 2 wt male-4 het male- 4 wt female-4 het female. (n) *n* = 4 CD wt male-7 CD het male-5 HFD wt male-8 HFD het male (o) *n* = 3 CD wt female-3 CD het female-7 HFD wt female-8 HFD het female, (p), (q) and (t) *n* = 4 CD wt male-7 CD het male-5 HFD wt male-8 HFD het male and *n* = 3 CD wt female-3 CD het female-7 HFD wt female-8 HFD het female. (r) *n* = 4 CD wt male-7 CD het male-3 HFD wt male-4 HFD het male and *n* = 3 CD wt female-3 CD het female-4 HFD wt female-4 HFD het female. (s) *n* = 4 CD wt male-7 CD het male-5 HFD wt male- 8 HFD het male and *n* = 3 CD wt female-2 CD het female-7 HFD wt female- 8 HFD het female. Supplementary Fig. 4 (g) SCF (*n* = 8 only for HFD + D-ser *n* = 7), PGF (*n* = 8 only for CD and HFD + D-ser *n* = 7), PRF (*n* = 8 only for HFD + D-ser *n* = 7) and BAT (*n* = 8 only for HFD and HFD + D-ser *n* = 7).

### Reporting summary

Further information on research design is available in the Nature Research Reporting Summary linked to this article.

## Supplementary information

Supplementary Information

Description of Additional Supplementary Files

Supplementary Data 1

Supplementary Data 2

Supplementary Data 3

Supplementary Movie 1

Reporting Summary

## Data Availability

All sequencing data that support the findings of this study have been deposited in the National Center for Biotechnology Information Gene Expression Omnibus (GEO) and are accessible through the GEO Series accession number GSE164350. All other relevant data are available from the corresponding author on request. Source data are provided with this paper.

## Source data

Source Data
