# Supplementary material for: Asc-1 regulates white versus beige adipocyte fate in a subcutaneous stromal cell population
Source: Nat Commun. 2021 Mar 11;12:1588. doi: 10.1038/s41467-021-21826-9 (PMC7952576; doi:10.1038/s41467-021-21826-9)
Supplement: Supplementary file 1 — Supplementary Information [file 41467_2021_21826_MOESM1_ESM.pdf]

Supplementary Figure 1

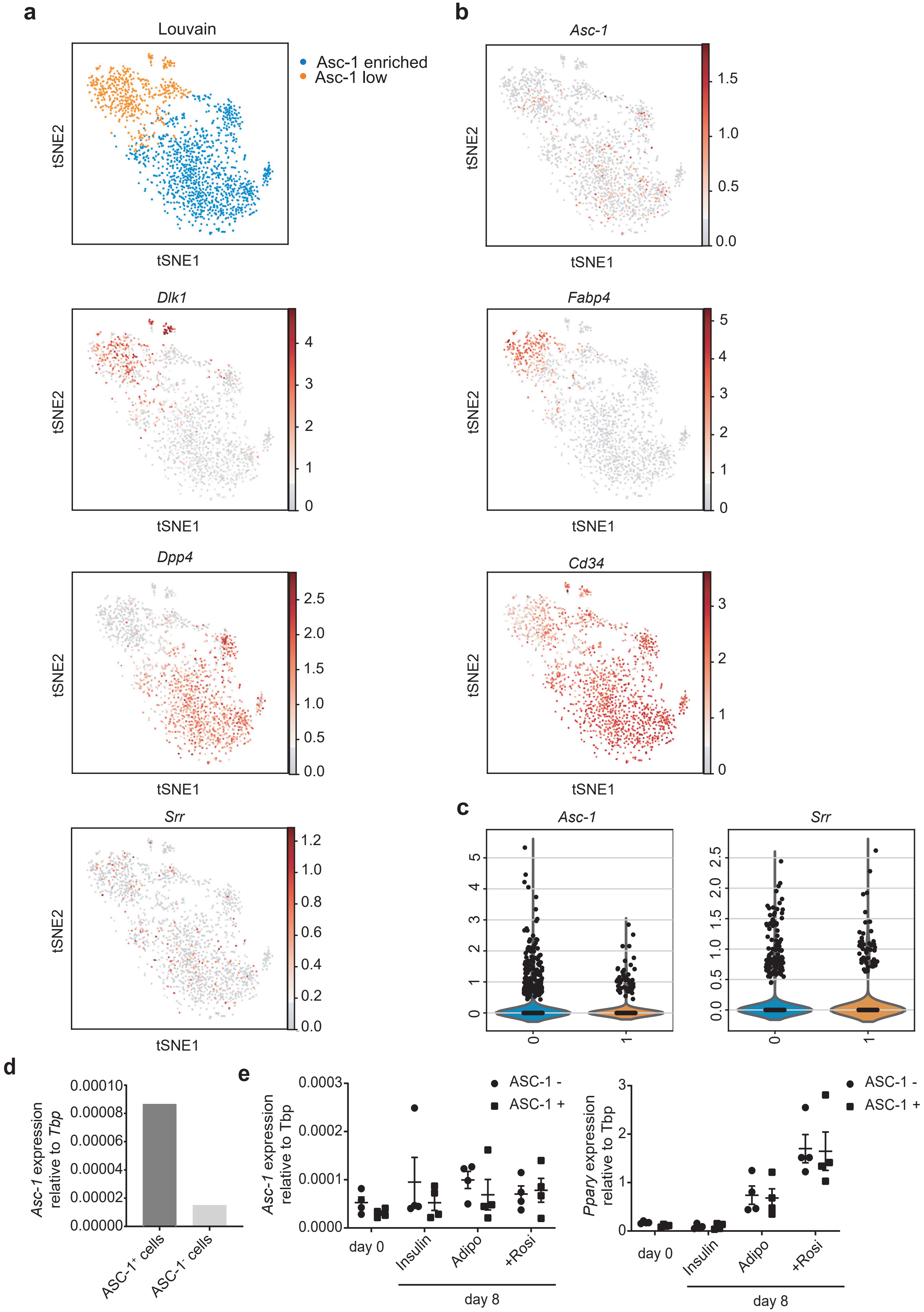

Supplementary Figure 1: Asc-1 expression is enriched in a subpopulation of adolescent preadipocytes

t-SNE of preadipocytes from adolescent SCF SVF (*n*=1,636) with (**a**) louvain cluster and (**b**) superimposed log1p, *Asc-1*, *Dlk1*, *Fabp4*, *Dpp4*, *Cd34* and, *Srr* expression. (**c**) Violin plot of *Asc-1* and *Srr*. Cluster 0 is *Asc-1* enriched and cluster 1 is *Asc-1* low. **(d)** qPCR for *Asc-1* from immortalized subcutaneous preadipocytes MACS sorted for ASC-1 (*n*=1). (**e**) Relative *Asc-1* and *Pparγ* expression in ASC-1^+ and ASC-1^- cells before (day 0) or after 8 days of differentiation with either insulin alone, the normal differentiation protocol (Adipo) or the differentiation protocol plus 1 µM rosiglitazone (*n*=4). Statistics were calculated using ordinary two-way ANOVA with Sidak's multiple comparison test. Data are shown as mean ± SEM.

Supplementary Figure 2

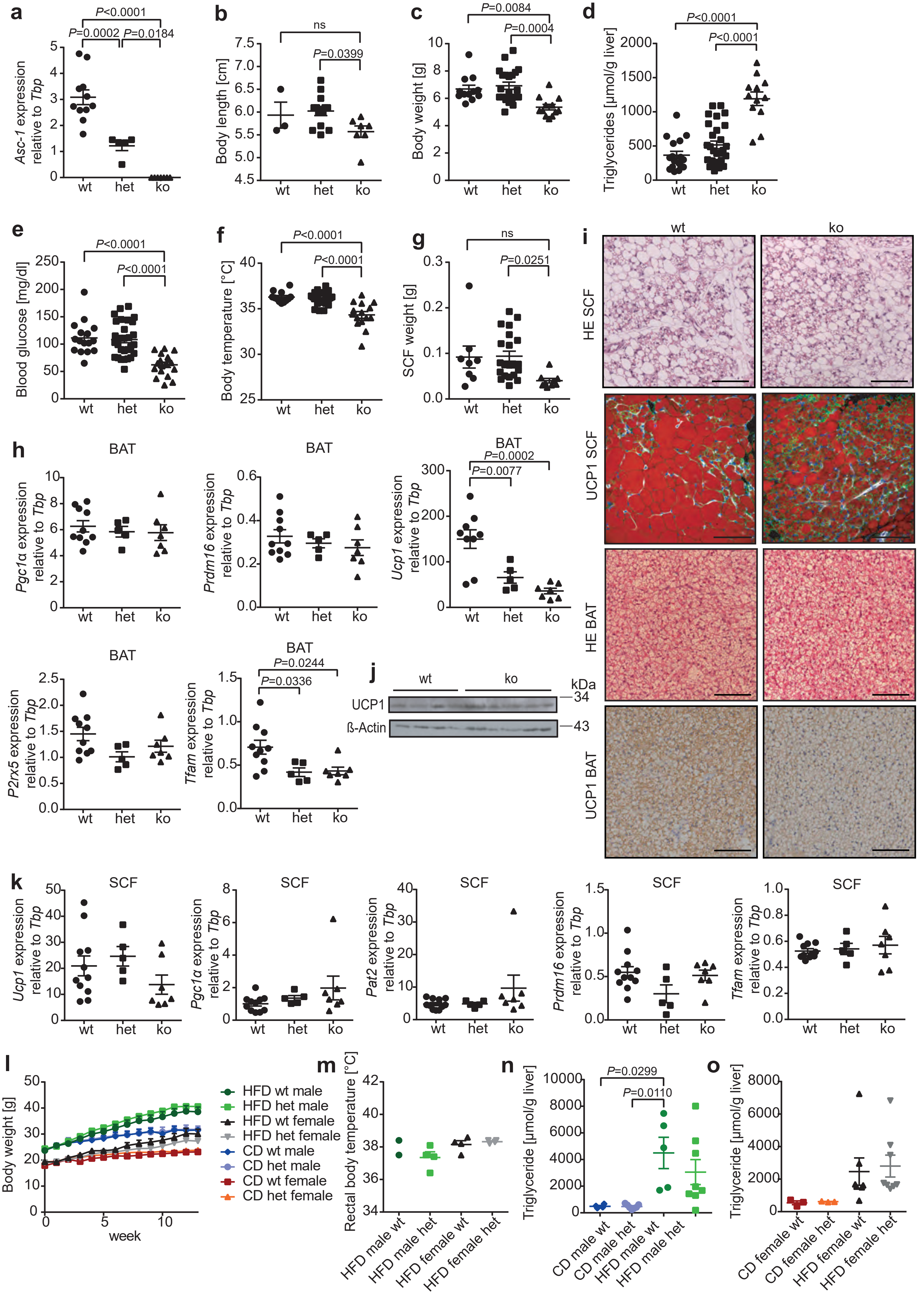

Supplementary Figure 2

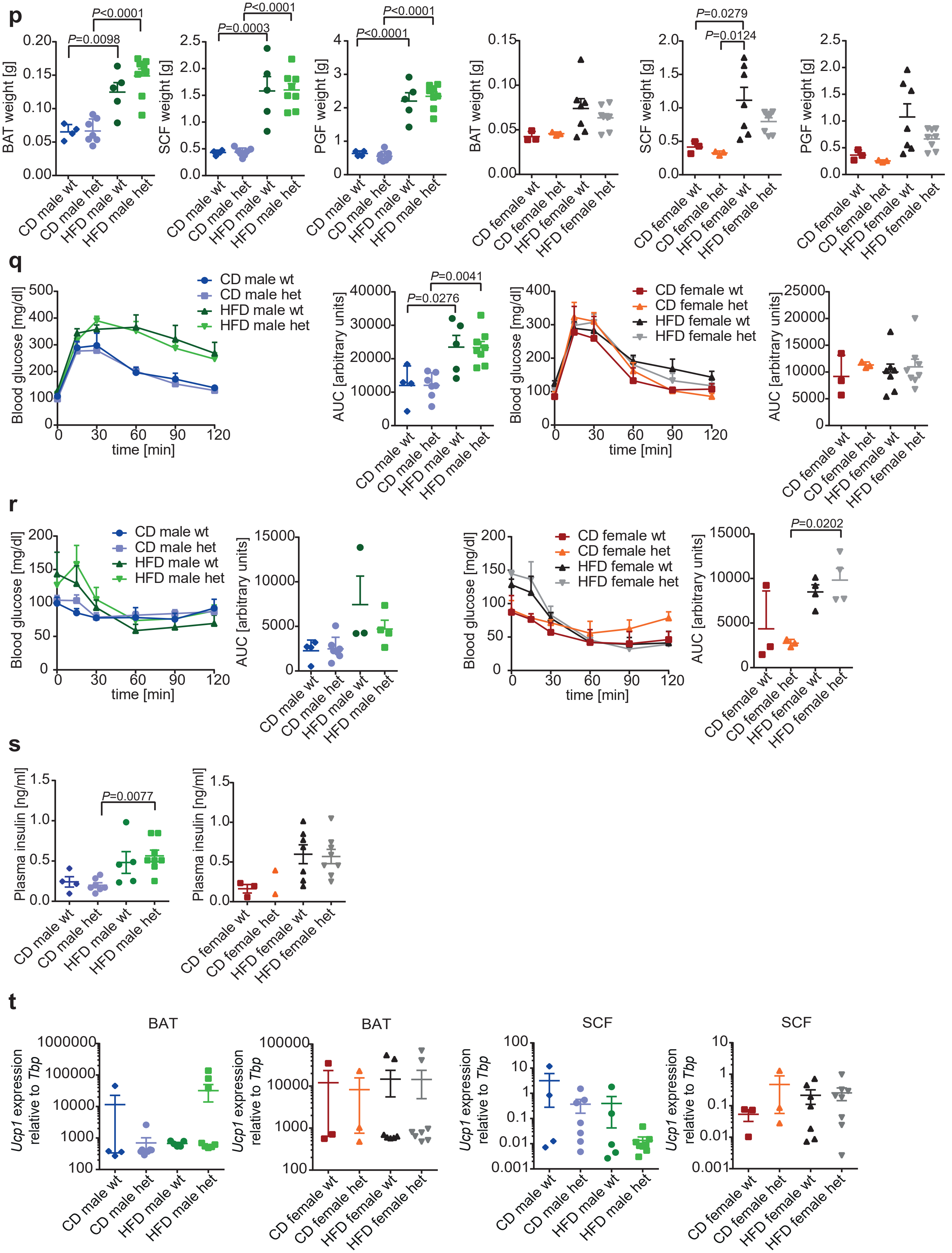

Supplementary Figure 2: Metabolic characterization of Asc-1 knockout mice

(**a**) Relative *Asc-1* expression in SCF of 2 week old Asc-1 wt, het and ko mice. (**b**) Body length and (**c**) weight of 2-week old Asc-1 wt, het and ko mice. (**d**) Liver triglyceride content of 2 week old Asc-1 wt, het and ko mice. (**e**) Blood glucose of 2-week old Asc-1 wt, het and ko mice. (**f**) Rectal body temperature of 2 week old Asc-1 wt, het and ko mice. (**g**) SCF weight of 2 week old Asc-1 wt, het and ko mice. (**h**) Relative expression of *Pgc1α*, *Prdm16*, *Ucp1*, *P2rx5* and *Tfam* in BAT of 2-week old mice. (**i**) Histology (*n*=4 wt- 3 ko) of SCF and BAT and immunofluorescence staining of UCP1 (green), F-Actin (grey), Lipids (red) and DAPI (blue) (*n*=1) of SCF and DAB staining of UCP1 (*n*=1) in BAT of 2-week old mice. Size bar 100 µm. (**j**) Western blot of UCP1 (33 kDa) and β-Actin (42 kDa) of 1-week old Asc-1 wt and ko mice. (**k**) Relative expression of *Ucp1*, *Pgc1α*, *Prdm16*, *Pat2* and *Tfam* in SCF of 2-week old Asc-1 wt, het and ko mice. (**l**) Body weight of 21-week old Asc-1 wt and het mice during 13 weeks of CD or HFD feeding (**m**) Rectal body temperature of 9-week old Asc-1 wt and het mice. Liver triglyceride content of 21-week old (**n**) male and (**o**) female Asc-1 wt and het mice after 13 weeks of CD or HFD feeding. (**p**) Tissue weights of male and female Asc-1 wt and het mice after 13 weeks of CD or HFD feeding. (**q**) GTT of Asc-1 wt and het mice after 12 weeks of CD or HFD feeding. (**r**) ITT after 12 weeks of CD or HFD feeding. (**s**) Plasma insulin levels after 12 weeks of CD or HFD feeding. (**t**) Relative *Ucp1* expression in SCF and BAT of Asc-1 wt and het mice after 12 weeks of CD or HFD feeding. Statistics were calculated using ordinary one-way ANOVA with Tukey's multiple comparison post-hoc test. n values are shown in the statistics section. Data are shown as mean ± SEM.

Supplementary Figure 3

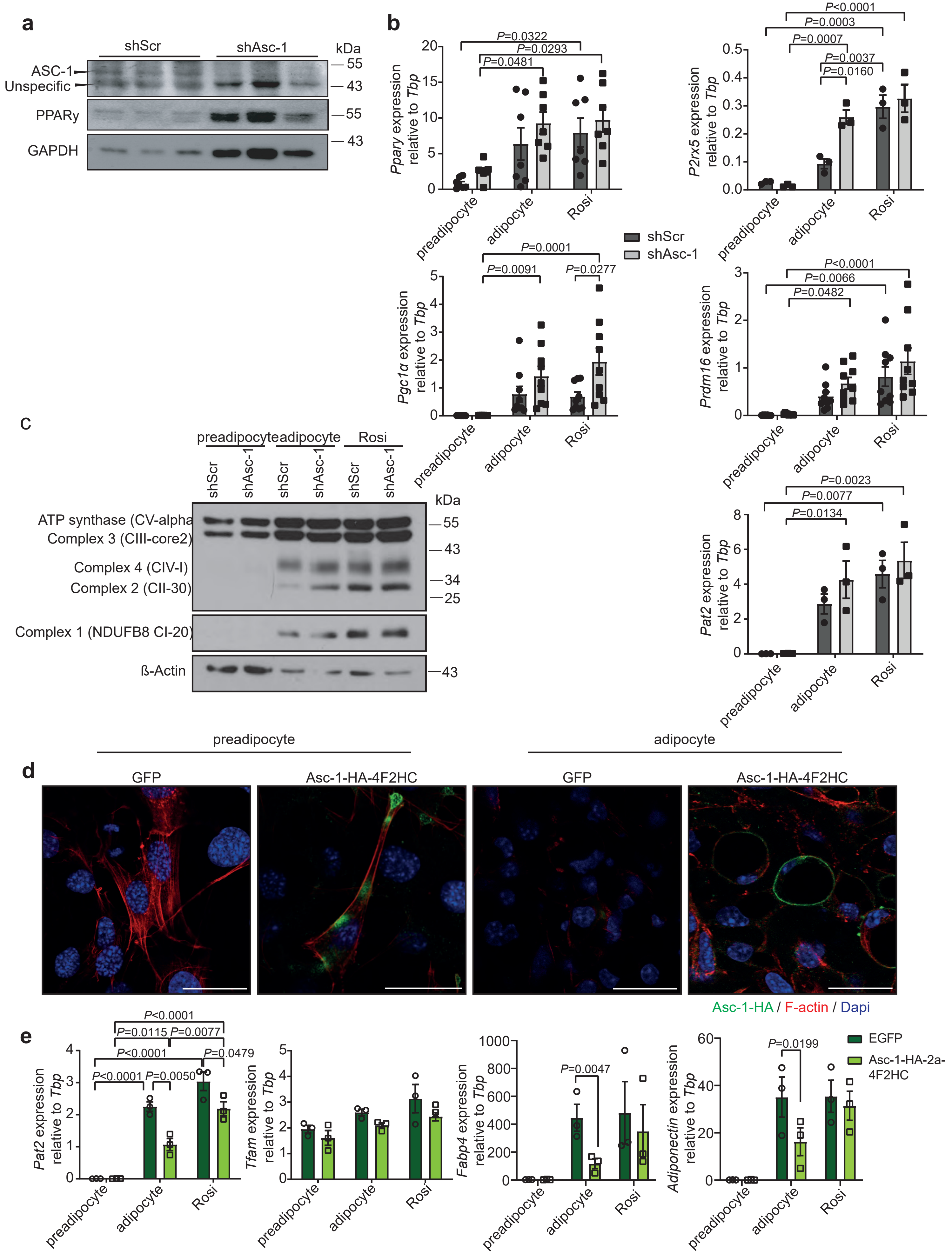

Supplementary Figure 3: Asc-1 regulates thermogenic gene expression in brown and white adipocytes

(**a**) Western blot of ASC-1 (~45 kDa), PPARγ (54 kDa) and GAPDH (37 kDa) of differentiated shScr and shAsc-1 (*n*=3). shAsc-1 and shScr subcutaneous preadipocytes were grown to 100 % confluence and RNA or protein was taken from the day of differentiation start (preadipocyte) and after 8 days of differentiation with (Rosi) or without rosiglitazone (adipocyte). (**b**) Relative expression of *Ppary* (*n*=7), *P2rx5* (*n*=3), *Pgc1α* (*n*=9), *Prdm16* (*n*=9) and *Pat2* (*n*=3). (**c**) Western blot of mitochondrial respiratory chain complex subunits (CV alpha (53 kDa), CIII-core 2 (47 kDa), CIV-I (39 kDa), CII-30 (30 kDa), NDUFB8 (20 kDa)), UCP1 (33 kDa), β -actin (42 kDa) (*n*=1). (**d**) Immunofluorescence staining of Asc-1-HA-2a-4F2HC or EGFP overexpressing brown preadipocytes before and after differentiation. Green: ASC-1-HA. Red: F-Actin. Blue: DAPI (*n*=1). Size bar 30 µm. (**e**) Relative gene expression of *Pat2*, *Tfam*, *Fabp4* and *Adiponectin* (*n*=3). Statistics were calculated using ordinary two-way ANOVA with Tukey's or Sidak's multiple comparison post-hoc test. Data are shown as mean ± SEM.

Supplementary Figure 4

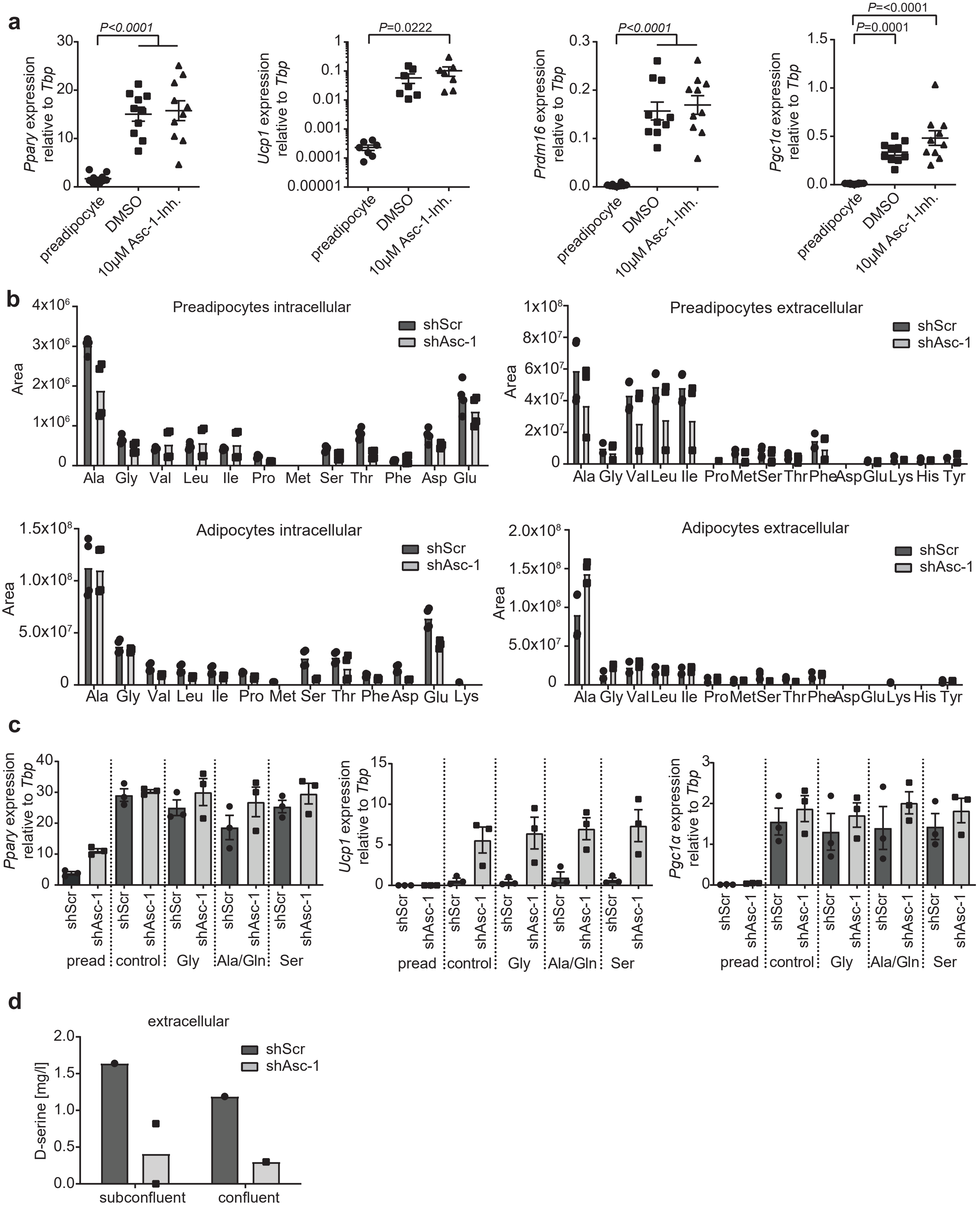

Supplementary Figure 4

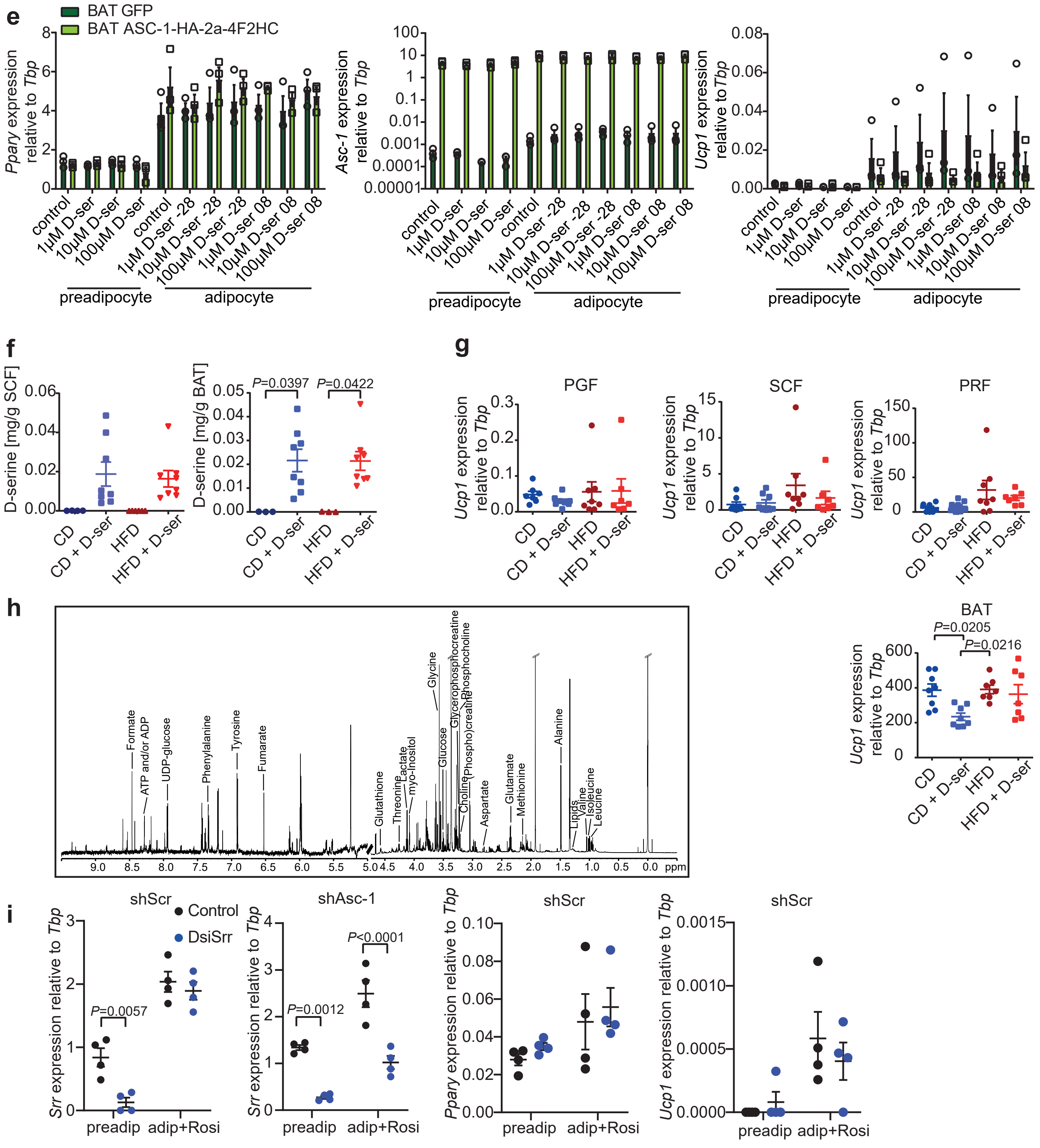

Supplementary Figure 4: Loss of Asc-1 alters intracellular amino acid levels

(**a**) Immortalized subcutaneous preadipocytes differentiated with 10 µM Asc-1 inhibitor (BMS-466442) or DMSO as control. Relative expression of of *Pparγ* (*n*=10), *Ucp1* (*n*=7), *Prdm16* (*n*=10) and *Pgc1α* (*n*=10) before (preadipocyte) or after 8 days of differentiation. (**b**) shAsc-1 and shScr preadipocytes were grown to 100 % confluence and differentiated for 8 days. Intra- and extracellular free amino acid levels of confluent preadipocytes and differentiated adipocytes (technical *n*=2, biological *n*=2). (**c**) Cells were grown to 100 % confluence and differentiation was induced without the indicated amino acids. Gene expression of *Pparγ, Ucp1* and *Pgc1α* (*n*=3). (**d**) Extracellular D-serine levels of subconfluent (*n*=1 shScr-2 shAsc-1) and confluent (*n*=1) preadipocytes (**e**) Asc-1-HA-2a-4F2HC or GFP overexpressing brown preadipocytes were treated with no (control), 1, 10 or 100 µM D-serine from 2 days before (-28) or from the day of differentiation on (08). Relative gene expression of *Pparγ*, *Asc-1* and *Ucp1* (*n*=3, only *Ucp1* of Asc-1-HA-4F2hc preadipocyte with 100 µM D-ser *n=2*). (**f**) 4 week old C57Bl/6 mice were fed a CD or HFD and supplemented with or without 10 g/l D-serine in drinking water for 8 weeks. D-serine concentrations in SCF (*n*=4 CD- 8 CD+Ser- 6 HFD- 8 HFD+D-Ser) and BAT (*n*=3 without D-Ser- 8 with D-Ser) after chronic D-serine supplementation. (**g**) Relative Ucp1 expression in SCF, PGF, PRF and BAT after 8 weeks of D-serine supplementation and CD or HFD feeding. n values are shown in the statistics section. (**h**) Sample NMR spectrum of cell extract with all metabolite assignments from Supplementary Table 3. The ppm area 9.5-5.0 ppm is enlarged x8. (**i**) Srr was knocked down using DsiSrr in proliferating shScr and shAsc-1 preadipocytes and differentiated upon confluency. *Srr* mRNA levels of shAsc-1 and shScr cells and *Ucp1* and *Pparγ* expression of shScr at day of induction (preadip) or cells differentiated following the regular differentiation protocol including rosiglitazone (adip+Rosi). A scrambled DsiRNA was used as negative Control (Control) for knockdown (*n*=4). Statistics were calculated using ordinary one- or two-way (i) ANOVA with Tukey's or Sidak´s multiple comparison post-hoc test. Data are shown as mean ± SEM.

Supplementary Table 1

Overlap Results

Collection(s):	BP	
# overlaps shown:	100	
# genesets in collections:	4436	
# genes in comparison (n):	2169	
# genes in universe (N):	45956	

Gene Set Name	# Genes in Gene Set (K)	Description
GO_CELLULAR_RESPONSE_TO_ORGANIC_SUBSTANCE	1848	Any process that results in a change in state or activity of a cell (in terms of movement, secretion, enzyme production, gene expression, etc.) as a result of an organic substance stimulus.
GO_RESPONSE_TO_ENDOGENOUS_STIMULUS	1450	Any process that results in a change in state or activity of a cell or an organism (in terms of movement, secretion, enzyme production, gene expression, etc.) as a result of a stimulus arising within the organism.
GO_REGULATION_OF_CELL_PROLIFERATION	1496	Any process that modulates the frequency, rate or extent of cell proliferation.
GO_REGULATION_OF_MULTICELLULAR_ORGANISMAL_DEVELOPMENT	1672	Any process that modulates the frequency, rate or extent of multicellular organismal development.
GO_ORGANONITROGEN_COMPOUND_METABOLIC_PROCESS	1796	The chemical reactions and pathways involving organonitrogen compound.
GO_TISSUE_DEVELOPMENT	1518	The process whose specific outcome is the progression of a tissue over time, from its formation to the mature structure.
GO_RESPONSE_TO_OXYGEN_CONTAINING_COMPOUND	1381	Any process that results in a change in state or activity of a cell or an organism (in terms of movement, secretion, enzyme production, gene expression, etc.) as a result of an oxygen-containing compound stimulus.
GO_POSITIVE_REGULATION_OF_RESPONSE_TO_STIMULUS	1929	Any process that activates, maintains or increases the rate of a response to a stimulus. Response to stimulus is a change in state or activity of a cell or an organism (in terms of movement, secretion, enzyme production, gene expression, etc.) as a res
GO_REGULATION_OF_CELL_DIFFERENTIATION	1492	Any process that modulates the frequency, rate or extent of cell differentiation, the process in which relatively unspecialized cells acquire specialized structural and functional features.
GO_RESPONSE_TO_EXTERNAL_STIMULUS	1821	Any process that results in a change in state or activity of a cell or an organism (in terms of movement, secretion, enzyme production, gene expression, etc.) as a result of an external stimulus.
GO_POSITIVE_REGULATION_OF_MULTICELLULAR_ORGANISMAL_PROCESS	1395	Any process that activates or increases the frequency, rate or extent of an organismal process, any of the processes pertinent to the function of an organism above the cellular level; includes the integrated processes of tissues and organs.
GO_NEGATIVE_REGULATION_OF_RESPONSE_TO_STIMULUS	1360	Any process that stops, prevents, or reduces the frequency, rate or extent of a response to a stimulus. Response to stimulus is a change in state or activity of a cell or an organism (in terms of movement, secretion, enzyme production, gene expressi
GO_REGULATION_OF_ANATOMICAL_STRUCTURE_MORPHOGENESIS	1021	Any process that modulates the frequency, rate or extent of anatomical structure morphogenesis.
GO_REGULATION_OF_CELL_DEATH	1472	Any process that modulates the rate or frequency of cell death. Cell death is the specific activation or halting of processes within a cell so that its vital functions markedly cease, rather than simply deteriorating gradually over time, which culminates
GO_POSITIVE_REGULATION_OF_DEVELOPMENTAL_PROCESS	1142	Any process that activates or increases the rate or extent of development, the biological process whose specific outcome is the progression of an organism over time from an initial condition (e.g. a zygote, or a young adult) to a later condition (e.g. a
GO_PROTEIN_LOCALIZATION	1805	Any process in which a protein is transported to, or maintained in, a specific location.
GO_CELL_DEVELOPMENT	1426	The process whose specific outcome is the progression of the cell over time, from its formation to the mature structure. Cell development does not include the steps involved in committing a cell to a specific fate.
GO_POSITIVE_REGULATION_OF_CELL_COMMUNICATION	1532	Any process that increases the frequency, rate or extent of cell communication. Cell communication is the process that mediates interactions between a cell and its surroundings. Encompasses interactions such as signaling or attachment between o
GO_CIRCULATORY_SYSTEM_DEVELOPMENT	788	The process whose specific outcome is the progression of the circulatory system over time, from its formation to the mature structure. The circulatory system is the organ system that passes nutrients (such as amino acids and electrolytes), gases, ho
GO_SMALL_MOLECULE_METABOLIC_PROCESS	1767	The chemical reactions and pathways involving small molecules, any low molecular weight, monomeric, non-encoded molecule.
GO_CATABOLIC_PROCESS	1773	The chemical reactions and pathways resulting in the breakdown of substances, including the breakdown of carbon compounds with the liberation of energy for use by the cell or organism.
GO_REGULATION_OF_PHOSPHORUS_METABOLIC_PROCESS	1618	Any process that modulates the frequency, rate or extent of the chemical reactions and pathways involving phosphorus or compounds containing phosphorus.
GO_REGULATION_OF_CELLULAR_COMPONENT_MOVEMENT	771	Any process that modulates the frequency, rate or extent of the movement of a cellular component.
GO_NEGATIVE_REGULATION_OF_MULTICELLULAR_ORGANISMAL_PROCESS	983	Any process that stops, prevents, or reduces the frequency, rate or extent of an organismal process, the processes pertinent to the function of an organism above the cellular level; includes the integrated processes of tissues and organs.
GO_CELLULAR_RESPONSE_TO_ENDOGENOUS_STIMULUS	1008	Any process that results in a change in state or activity of a cell (in terms of movement, secretion, enzyme production, gene expression, etc.) as a result of a stimulus arising within the organism.
GO_REGULATION_OF_PROTEIN_MODIFICATION_PROCESS	1710	Any process that modulates the frequency, rate or extent of the covalent alteration of one or more amino acid residues within a protein.
GO_REGULATION_OF_TRANSPORT	1804	Any process that modulates the frequency, rate or extent of the directed movement of substances (such as macromolecules, small molecules, ions) into, out of or within a cell, or between cells, by means of some agent such as a transporter or pore.
GO_NEUROGENESIS	1402	Generation of cells within the nervous system.
GO_REGULATION_OF_INTRACELLULAR_SIGNAL_TRANSDUCTION	1656	Any process that modulates the frequency, rate or extent of intracellular signal transduction.
GO_REGULATION_OF_RESPONSE_TO_STRESS	1468	Any process that modulates the frequency, rate or extent of a response to stress. Response to stress is a change in state or activity of a cell or an organism (in terms of movement, secretion, enzyme production, gene expression, etc.) as a result of a
GO_POSITIVE_REGULATION_OF_MOLECULAR_FUNCTION	1791	Any process that activates or increases the rate or extent of a molecular function, an elemental biological activity occurring at the molecular level, such as catalysis or binding.
GO_LOCOMOTION	1114	Self-propelled movement of a cell or organism from one location to another.
GO_NEGATIVE_REGULATION_OF_CELL_COMMUNICATION	1192	Any process that decreases the frequency, rate or extent of cell communication. Cell communication is the process that mediates interactions between a cell and its surroundings. Encompasses interactions such as signaling or attachment between c
GO_ESTABLISHMENT_OF_PROTEIN_LOCALIZATION	1423	The directed movement of a protein to a specific location.
GO_MOVEMENT_OF_CELL_OR_SUBCELLULAR_COMPONENT	1275	The directed, self-propelled movement of a cell or subcellular component without the involvement of an external agent such as a transporter or a pore.
GO_IMMUNE_SYSTEM_PROCESS	1984	Any process involved in the development or functioning of the immune system, an organismal system for calibrated responses to potential internal or invasive threats.
GO_POSITIVE_REGULATION_OF_PROTEIN_METABOLIC_PROCESS	1492	Any process that activates or increases the frequency, rate or extent of the chemical reactions and pathways involving a protein.
GO_ESTABLISHMENT_OF_LOCALIZATION_IN_CELL	1676	The directed movement of a substance or cellular entity, such as a protein complex or organelle, to a specific location within, or in the membrane of, a cell.
GO_CELLULAR_MACROMOLECULE_LOCALIZATION	1234	Any process in which a macromolecule is transported to, and/or maintained in, a specific location at the level of a cell. Localization at the cellular level encompasses movement within the cell, from within the cell to the cell surface, or from one locat
GO_REGULATION_OF_TRANSCRIPTION_FROM_RNA_POLYMERASE_II_PROMOTER	1784	Any process that modulates the frequency, rate or extent of transcription from an RNA polymerase II promoter.
GO_RESPONSE_TO_HORMONE	893	Any process that results in a change in state or activity of a cell or an organism (in terms of movement, secretion, enzyme production, gene expression, etc.) as a result of a hormone stimulus.
GO_RESPONSE_TO_NITROGEN_COMPOUND	859	Any process that results in a change in state or activity of a cell or an organism (in terms of movement, secretion, enzyme production, gene expression, etc.) as a result of a nitrogen compound stimulus.
GO_RESPONSE_TO_ABIOTIC_STIMULUS	1024	Any process that results in a change in state or activity of a cell or an organism (in terms of movement, secretion, enzyme production, gene expression, etc.) as a result of an abiotic (non-living) stimulus.
GO_RESPONSE_TO_LIPID	888	Any process that results in a change in state or activity of a cell or an organism (in terms of movement, secretion, enzyme production, gene expression, etc.) as a result of a lipid stimulus.
GO_POSITIVE_REGULATION_OF_BIOSYNTHETIC_PROCESS	1805	Any process that activates or increases the frequency, rate or extent of the chemical reactions and pathways resulting in the formation of substances.
GO_POSITIVE_REGULATION_OF_GENE_EXPRESSION	1733	Any process that increases the frequency, rate or extent of gene expression. Gene expression is the process in which a gene's coding sequence is converted into a mature gene product or products (proteins or RNA). This includes the production of a
GO_ORGANONITROGEN_COMPOUND_BIOSYNTHETIC_PROCESS	1024	The chemical reactions and pathways resulting in the formation of organonitrogen compound.
GO_INTRACELLULAR_SIGNAL_TRANSDUCTION	1572	The process in which a signal is passed on to downstream components within the cell, which become activated themselves to further propagate the signal and finally trigger a change in the function or state of the cell.
GO_POSITIVE_REGULATION_OF_CELL_DIFFERENTIATION	823	Any process that activates or increases the frequency, rate or extent of cell differentiation.
GO_NEGATIVE_REGULATION_OF_MOLECULAR_FUNCTION	1079	Any process that stops or reduces the rate or extent of a molecular function, an elemental biological activity occurring at the molecular level, such as catalysis or binding.
GO_BIOLOGICAL_ADHESION	1032	The attachment of a cell or organism to a substrate, another cell, or other organism. Biological adhesion includes intracellular attachment between membrane regions.
GO_VASCULATURE_DEVELOPMENT	469	The process whose specific outcome is the progression of the vasculature over time, from its formation to the mature structure. The vasculature is an interconnected tubular multi-tissue structure that contains fluid that is actively transported aroun
GO_RESPONSE_TO_ORGANIC_CYCLIC_COMPOUND	917	Any process that results in a change in state or activity of a cell or an organism (in terms of movement, secretion, enzyme production, gene expression, etc.) as a result of an organic cyclic compound stimulus.
GO_EXTRACELLULAR_STRUCTURE_ORGANIZATION	304	A process that is carried out at the cellular level which results in the assembly, arrangement of constituent parts, or disassembly of structures in the space external to the outermost structure of a cell. For cells without external protective or external
GO_ORGAN_MORPHOGENESIS	841	Morphogenesis of an organ. An organ is defined as a tissue or set of tissues that work together to perform a specific function or functions. Morphogenesis is the process in which anatomical structures are generated and organized. Organs are comm
GO_POSITIVE_REGULATION_OF_CELLULAR_COMPONENT_ORGANIZATION	1152	Any process that activates or increases the frequency, rate or extent of a process involved in the formation, arrangement of constituent parts, or disassembly of cell structures, including the plasma membrane and any external encapsulating structu
GO_POSITIVE_REGULATION_OF_CELL_PROLIFERATION	814	Any process that activates or increases the rate or extent of cell proliferation.
GO_INTERSPECIES_INTERACTION_BETWEEN_ORGANISMS	662	Any process in which an organism has an effect on an organism of a different species.
GO_CELLULAR_CATABOLIC_PROCESS	1322	The chemical reactions and pathways resulting in the breakdown of substances, carried out by individual cells.
GO_CELLULAR_RESPONSE_TO_OXYGEN_CONTAINING_COMPOUND	799	Any process that results in a change in state or activity of a cell (in terms of movement, secretion, enzyme production, gene expression, etc.) as a result of an oxygen-containing compound stimulus.
GO_BLOOD_VESSEL_MORPHOGENESIS	364	The process in which the anatomical structures of blood vessels are generated and organized. The blood vessel is the vasculature carrying blood.
GO_PROTEIN_LOCALIZATION_TO_MEMBRANE	376	A process in which a protein is transported to, or maintained in, a specific location in a membrane.
GO_PROTEIN_TARGETING_TO_MEMBRANE	157	The process of directing proteins towards a membrane, usually using signals contained within the protein.
GO_REGULATION_OF_IMMUNE_SYSTEM_PROCESS	1403	Any process that modulates the frequency, rate, or extent of an immune system process.
GO_EPITHELIUM_DEVELOPMENT	945	The process whose specific outcome is the progression of an epithelium over time, from its formation to the mature structure. An epithelium is a tissue that covers the internal or external surfaces of an anatomical structure.
GO_ENZYME_LINKED_RECEPTOR_PROTEIN_SIGNALING_PATHWAY	689	Any series of molecular signals initiated by the binding of an extracellular ligand to a receptor on the surface of the target cell, where the receptor possesses catalytic activity or is closely associated with an enzyme such as a protein kinase, and endir
GO_NEGATIVE_REGULATION_OF_CELL_DEATH	872	Any process that decreases the rate or frequency of cell death. Cell death is the specific activation or halting of processes within a cell so that its vital functions markedly cease, rather than simply deteriorating gradually over time, which culminates i
GO_POSITIVE_REGULATION_OF_CATALYTIC_ACTIVITY	1518	Any process that activates or increases the activity of an enzyme.

GO_REGULATION_OF_HYDROLASE_ACTIVITY	1327	Any process that modulates the frequency, rate or extent of hydrolase activity, the catalysis of the hydrolysis of various bonds, e.g. C-O, C-N, C-C, phosphoric anhydride bonds, etc. Hydrolase is the systematic name for any enzyme of EC class 3.
GO_REGULATION_OF_RESPONSE_TO_EXTERNAL_STIMULUS	926	Any process that modulates the frequency, rate or extent of a response to an external stimulus.
GO_MEMBRANE_ORGANIZATION	899	A process which results in the assembly, arrangement of constituent parts, or disassembly of a membrane. A membrane is a double layer of lipid molecules that encloses all cells, and, in eukaryotes, many organelles; may be a single or double lipid b
GO_ANATOMICAL_STRUCTURE_FORMATION_INVOLVED_IN_MORPHOGENESIS	957	The developmental process pertaining to the initial formation of an anatomical structure from unspecified parts. This process begins with the specific processes that contribute to the appearance of the discrete structure and ends when the structur
GO_POSITIVE_REGULATION_OF_PHOSPHORUS_METABOLIC_PROCESS	1036	Any process that increases the frequency, rate or extent of the chemical reactions and pathways involving phosphorus or compounds containing phosphorus.
GO_NEGATIVE_REGULATION_OF_DEVELOPMENTAL_PROCESS	801	Any process that stops, prevents or reduces the rate or extent of development, the biological process whose specific outcome is the progression of an organism over time from an initial condition (e.g. a zygote, or a young adult) to a later condition (
GO_ESTABLISHMENT_OF_PROTEIN_LOCALIZATION_TO_MEMBRANE	264	The directed movement of a protein to a specific location in a membrane.
GO_NEGATIVE_REGULATION_OF_PROTEIN_METABOLIC_PROCESS	1087	Any process that stops, prevents, or reduces the frequency, rate or extent of chemical reactions and pathways involving a protein.
GO_REGULATION_OF_CELLULAR_LOCALIZATION	1277	Any process that modulates the frequency, rate or extent of a process in which a cell, a substance, or a cellular entity is transported to, or maintained in a specific location within or in the membrane of a cell.
GO_PHOSPHATE_CONTAINING_COMPOUND_METABOLIC_PROCESS	1977	The chemical reactions and pathways involving the phosphate group, the anion or salt of any phosphoric acid.
GO_POSITIVE_REGULATION_OF_PROTEIN_MODIFICATION_PROCESS	1135	Any process that activates or increases the frequency, rate or extent of the covalent alteration of one or more amino acid residues within a protein.
GO_INTRACELLULAR_PROTEIN_TRANSPORT	781	The directed movement of proteins in a cell, including the movement of proteins between specific compartments or structures within a cell, such as organelles of a eukaryotic cell.
GO_NEGATIVE_REGULATION_OF_NITROGEN_COMPOUND_METABOLIC_PROCESS	1517	Any process that stops, prevents, or reduces the frequency, rate or extent of the chemical reactions and pathways involving nitrogen or nitrogenous compounds.
GO_HOMEOSTATIC_PROCESS	1337	Any biological process involved in the maintenance of an internal steady state.
GO_POSITIVE_REGULATION_OF_INTRACELLULAR_SIGNAL_TRANSDUCTION	876	Any process that activates or increases the frequency, rate or extent of intracellular signal transduction.
GO_ESTABLISHMENT_OF_PROTEIN_LOCALIZATION_TO_ENDOPLASMIC_RETICULUM	104	The directed movement of a protein to a specific location in the endoplasmic reticulum.
GO_TRANSLATIONAL_INITIATION	146	The process preceding formation of the peptide bond between the first two amino acids of a protein. This includes the formation of a complex of the ribosome, mRNA, and an initiation complex that contains the first aminoacyl-tRNA.
GO_NEURON_DIFFERENTIATION	874	The process in which a relatively unspecialized cell acquires specialized features of a neuron.
GO_NEGATIVE_REGULATION_OF_CELL_PROLIFERATION	643	Any process that stops, prevents or reduces the rate or extent of cell proliferation.
GO_CELL_PROLIFERATION	672	The multiplication or reproduction of cells, resulting in the expansion of a cell population.
GO_SKELETAL_SYSTEM_DEVELOPMENT	455	The process whose specific outcome is the progression of the skeleton over time, from its formation to the mature structure. The skeleton is the bony framework of the body in vertebrates (endoskeleton) or the hard outer envelope of insects (exos
GO_PROTEIN_LOCALIZATION_TO_ENDOPLASMIC_RETICULUM	123	A process in which a protein is transported to, or maintained in, a location within the endoplasmic reticulum.
GO_NEGATIVE_REGULATION_OF_GENE_EXPRESSION	1493	Any process that decreases the frequency, rate or extent of gene expression. Gene expression is the process in which a gene's coding sequence is converted into a mature gene product or products (proteins or RNA). This includes the production of a
GO_CELLULAR_COMPONENT_MORPHOGENESIS	900	The process in which cellular structures, including whole cells or cell parts, are generated and organized.
GO_CELL_MOTILITY	835	Any process involved in the controlled self-propelled movement of a cell that results in translocation of the cell from one place to another.
GO_RESPONSE_TO_GROWTH_FACTOR	475	Any process that results in a change in state or activity of a cell or an organism (in terms of movement, secretion, enzyme production, gene expression, etc.) as a result of a growth factor stimulus.
GO_SINGLE_ORGANISM_CELLULAR_LOCALIZATION	898	A cellular localization which involves only one organism.
GO_PROTEIN_TARGETING	406	The process of targeting specific proteins to particular regions of the cell, typically membrane-bounded subcellular organelles. Usually requires an organelle specific protein sequence motif.
GO_RESPONSE_TO_WOUNDING	563	Any process that results in a change in state or activity of a cell or an organism (in terms of movement, secretion, enzyme production, gene expression, etc.) as a result of a stimulus indicating damage to the organism.
GO_CELL_MORPHOGENESIS_INVOLVED_IN_DIFFERENTIATION	513	The change in form (cell shape and size) that occurs when relatively unspecialized cells, e.g. embryonic or regenerative cells, acquire specialized structural and/or functional features that characterize the cells, tissues, or organs of the mature organis
GO_REGULATION_OF_CELL_ADHESION	629	Any process that modulates the frequency, rate or extent of attachment of a cell to another cell or to the extracellular matrix.
GO_CELLULAR_AMIDE_METABOLIC_PROCESS	727	The chemical reactions and pathways involving an amide, any derivative of an oxoacid in which an acidic hydroxy group has been replaced by an amino or substituted amino group, as carried out by individual cells.

Supplementary Table 1: Gene set enrichment analysis (GSEA) of *Asc-1* enriched cells of adolescent subcutaneous preadipocytes.

GSEA was performed using 2,442 genes listed in Supplementary Table 2. A false-discovery corrected *P*-value for differential expression lower than 0.01.

Supplementary Table 2

Gene Name
Sntg1
Adhfe1
Sulf1
Tram1
Gm9947
Msc
Rpl7
Eloc
Pi15
Crispld1
Gm28153
Ogfrl1
Smap1
Adgrb3
Rab23
Dst
Arid5a
Cnnm4
Lipt1
Map4k4
Il1r1
Col3a1
Col5a2
Slc40a1
Nabp1
Nab1
Inpp1
1700019D03Rik
Ankrd44
Sf3b1
Hspd1
Spats2l
Clk1
Tmem237
Fzd7
Gm973
Nop58
Raph1
Nrp2
Eef1b2
Gpr1
Myl1
Fn1
Xrcc5
Igfbp5
Tns1

Arpc2
Slc11a1
Cyp27a1
Dnpep
Speg
Chpf
Slc4a3
Epha4
Kcne4
Agfg1
Pid1
Sp100
Ptma
Eif4e2
Efhd1
Sh3bp4
Ackr3
Col6a3
Lrrfip1
Ramp1
Scly
Twist2
Hdac4
Gpc1
Sned1
Hdlbp
Bok
Thap4
Ppip5k2
Pam
Phlpp1
Bcl2
Dsel
Gli2
Dbi
Ccnt2
Gm15675
Cd55
Rab7b
Prelp
Btg2
Ppp1r12b
Nav1
Csrp1
Phlda3
Gm19705
Pla2g4a
Ptgs2
Ptgs2os2
Fam129a

1700025G04Rik
Lamc2
Lamc1
Rgs16
Ier5
Tor3a
Angptl1
Fmo1
Fmo2
Prrx1
Kifap3
Atp1b1
Dpt
Mpzl1
Ildr2
Aldh9a1
Mgst3
Rxrg
Hsd17b7
Ddr2
Uap1
Olfml2b
Apoa2
Adamts4
Pex19
Igsf8
Atp1a2
Tagln2
Ackr1
Cadm3
Ifi207
Ifi204
Mndal
Ifi203
Ifi205
Cep170
Hnrnpu
H3f3a
Sde2
Lefty1
Tmem63a
Ephx1
Degs1
Capn2
Disp1
Aida
Hlx
Tgfb2
Kcnk2
Nenf

Lpgat1
Gm15867
Lamb3
Hsd11b1
G0s2
Cd34
Frmd4a
Camk1d
Cdc123
Atp5c1
Itih5
Fbxo18
Vim
Plxdc2
Apbb1ip
Mrpl41
Tubb4b
Tmem203
Uap1l1
Entpd2
Npdc1
Paxx
Notch1
Agpat2
Surf4
Col5a1
Ralgds
Rapgef1
2600006K01Rik
Cercam
Ier5l
Prrx2
Ptges
Ncs1
Hmcn2
Ass1
Abl1
Aif1l
Dnm1
Ak1
Rpl12
Garnl3
Angptl2
Hspa5
Phf19
Gsn
Stom
Ptgs1
Nek6
Nr6a1

Olfml2a
Rpl35
Zeb2
Acvr2a
Lypd6
Cacnb4
Cd302
Rbms1
Dpp4
Fap
Scn7a
Stk39
Cers6
Cybrd1
Map3k20
Hoxd3os1
Hoxd8
Nfe2l2
Pde11a
Osbpl6
Sestd1
Pde1a
Nckap1
Zc3h15
Itgav
Fam171b
Calcrl
Serping1
Timm10
Slc43a3
Tnks1bp1
Ptprj
Psmc3
Mdk
Creb3l1
Chst1
Tspan18
Prr5l
Pamr1
Cd44
Cat
Rcn1
Mpped2
Lgr4
Ccdc34
Fibin
Ano3
Nop10
Ryr3
Thbs1

Chp1
Stard9
Lcmt2
Serf2
Casc4
B2m
Duoxa1
Duox1
Slc30a4
Sqor
Fbn1
Fgf7
Sppl2a
Gm14005
Fbln7
Sirpa
Stk35
Nop56
Cpxm1
Ptpra
4930402H24Rik
Adam33
Hspa12b
Cenpb
Rassf2
Tmem230
Crls1
Bmp2
Plcb4
Mkks
Jag1
Ism1
Macrod2
Rrbp1
Slc24a3
Rin2
Naa20
Thbd
Cst3
Fkbp1a
Trib3
Defb25
Rem1
Id1
Pofut1
Necab3
1700003F12Rik
E2f1
Dynlrb1
Map1lc3a

Edem2
Procr
Rbm39
Scand1
2900097C17Rik
Dlgap4
Tgif2
Rab5if
Samhd1
Rpn2
Lbp
Mafb
Top1
Fitm2
Pkig
Matn4
Pigt
Snx21
Ctsa
Pltp
Eya2
Sulf2
Ptgis
Snai1
Cebpb
Dpm1
Tshz2
Bmp7
Zbp1
Pmepa1
Npepl1
Gnas
Ctsz
Atp5e
Rps21
Helz2
Clcn5
Praf2
Rbm3
Srpx
Tspan7
Mid1ip1
Ddx3x
Cask
Chst7
Cdk16
Agtr2
Dock11
Il13ra1
Pgrmc1

Slc25a5
Rpl39
Lamp2
Dcaf12l1
Elf4
Igsf1
Gpc3
Mospd1
Fhl1
Map7d3
Mamld1
Bgn
Idh3g
Rpl10
Fundc2
Pls3
Prrg1
Tmem47
Amer1
Heph
Ar
Tmem28
Rps4x
Cited1
Slc16a2
Rlim
Rtl3
P2ry10b
Itm2a
Pcdh11x
Pcdh19
Srpx2
Rpl36a
Armcx3
Arxes2
Arxes1
Tceal8
Tceal9
Bex3
Tceal1
Nrk
Tsc22d3
Hsd17b10
Prdx4
Eif1ax
Adgrg2
Reps2
Rbbp7
Ap1s2
Vegfd

Mospd2
Gpm6b
Rab9
Tmsb4x
Gm15261
Gm8797
Mrps28
Fabp5
Fabp4
Car13
Car3
Cyp7b1
Cp
Tnik
Rpl22l1
Sec62
Prkci
Skil
Gnb4
Ndufb5
Fgf2
Spry1
Fat4
Jade1
Pcdh10
Setd7
Mgst2
Ufm1
Postn
Rnf13
Siah2
P2ry14
Igsf10
P2ry1
Mme
Ssr3
Veph1
Ptx3
B3galnt1
Golim4
Fam198b
Ctso
Gucy1b1
Gucy1a1
Dchs2
Sfrp2
Gm26771
Mnd1
Fhdc1
Rps3a1

Pear1
Pmf1
Lmna
Ssr2
Thbs3
Krtcap2
Adam15
Gm15417
Cks1b
Shc1
Kcnn3
Atp8b2
Ubap2l
Rps27
S100a13
S100a16
S100a6
S100a11
S100a10
Selenbp1
Ctsk
Mcl1
Adamtsl4
Ecm1
Plekho1
Mtmr11
Txnip
Prkab2
Notch2
Hmgcs2
Phgdh
Ptgfrn
Atp1a1
Vangl1
A230001M10Rik
Ngf
Olfml3
Slc16a1
Rhoc
Wnt2b
Rap1a
Gm5547
Alx3
Gstm2
Psma5
Tmem167b
Prpf38b
Slc25a24
Amy1
Vcam1

Snx7
Slc44a3
A530020G20Rik
F3
Arhgap29
Abca4
Bcar3
Prss12
Tram1l1
Ank2
Enpep
Pla2g12a
Mcub
Ostc
Rpl34
Hadh
Papss1
Dkk2
Cxxc4
Ppp3ca
Adh7
Adh1
Adh5
Tspan5
Pdlim5
Gbp2
Gtf2b
Lmo4
Sh3glb1
Cyr61
Ddah1
Gng5
Ptgfr
Acadm
Slc44a5
Lhx8
Negr1
Ptger3
Wls
Rps20
Gm11808
Penk
Fam110b
Tox
8430436N08Rik
Car8
Gem
Runx1t1
Ggh
Manea

Pnrc1
Aco1
Tmem215
Dnaja1
Bag1
Ube2r2
Ubap1
AI464131
Enho
Cntfr
Il11ra1
Gm13305
Gm2564
Ccl19
Ccdc107
Creb3
Hrct1
Spaar
Reck
Grhpr
Shb
Col15a1
Sec61b
Tmeff1
Cavin4
AI427809
Slc44a1
Tmem38b
Zfp462
Klf4
Tmem245
Svep1
Gng10
Ugcg
Slc31a2
Rgs3
Col27a1
Atp6v1g1
Pappa
Astn2
Megf9
Lurap1l
Mpdz
Nfib
Gm11266
Frem1
Adamtsl1
Plin2
Rps6
Hacd4

Mtap
Tek
Jun
Cyp2j6
Nfia
Pgm2
Cachd1
Jak1
Leprot
Fyb2
Plpp3
Usp24
Hspb11
Podn
Scp2
Echdc2
Gpx7
Nrd1
Cdkn2c
Trabd2b
Efcab14
Uqcrh
Nasp
Akr1a1
Prdx1
Ptch2
Rps8
Gm12840
B4galt2
Ybx1
Kcnq4
Heyl
Pabpc4
Bmp8a
Ndufs5
Rspo1
Eva1b
Tekt2
Sfpq
Azin2
Ak2
Rbbp4
Marcksl1
Khdrbs1
Ptp4a2
Col16a1
Snhg12
Atpif1
Slc9a1
Trnp1

Hmgn2
Cep85
Maco1
Rsrp1
Syf2
Clic4
Pnrc2
Rpl11
Id3
Asap3
Camk2n1
Nbl1
Capzb
Mrto4
Igsf21
Mfap2
Efhd2
Kazn
Pdpn
Dhrs3
Tnfrsf1b
Plod1
Draxin
Pgd
Kif1b
Spsb1
H6pd
Rere
Errfi1
Gpr153
Smim1
Ski
Faap20
Gnb1
Mmp23
Vwa1
C1qtnf12
Isg15
Fzd1
Gm8773
Steap4
Adam22
Sema3d
Sema3a
Sema3e
Sema3c
Cd36
Gnai1
Lrrc17
Fam126a

Chpf2
Wdr86
Il6
Ost4
Emilin1
Khk
Cgref1
Mrpl33
Spon2
Ctbp1
Mxd4
Lrpap1
Cpz
Htra3
Ablim2
Afap1
Msx1
Cd38
Ldb2
Qdpr
Lgi2
Pi4k2b
Sel1l3
Smim20
Rbpj
Stim2
Klf3
Fam114a1
Rpl9
Ugdh
Smim14
Apbb2
Uchl1
Limch1
Gabra2
Slain2
Sgcb
Pdgfra
Igfbp7
Slc4a4
Cxcl1
Btc
Parm1
Naaa
44085
Ccng2
Fras1
Anxa3
Bmp2k
Antxr2

Prdm8
Bmp3
Prkg2
Hnrnpdl
Plac8
Hpse
Arhgap24
Slc10a6
Hsd17b13
Sparcl1
Pkd2
Gbp6
Lrrc8b
Lrrc8c
Lrrc8d
Tgfbr3
Ephx4
Evi5
Fam69a
Mfsd7a
Fgfrl1
Miat
Tpst2
Asphd2
2900026A02Rik
Wscd2
Cmklr1
Iscu
Tmem119
Coro1c
Trpv4
Gltp
Acads
Pxn
Rplp0
Ccdc60
Hspb8
Pebp1
Tesc
Med13l
Tbx5
Rpl6
Trafd1
Aldh2
Ppp1cc
Hvcn1
Atp2a2
Ift81
P2rx4
Orai1

Setd1b
Clip1
Hcar1
Kmt5a
Ncor2
Ubc
Bri3bp
Tmem132b
5930412G12Rik
Adgrd1
Chchd2
Vkorc1l1
Gusb
Tpst1
Auts2
Limk1
Gm10369
Cldn3
Mlxipl
Por
Dtx2
Cldn15
Serpine1
Trim56
Pcolce
Irs3
Mepce
Cyp3a13
Prkar1b
Adap1
Chst12
Ttyh3
Radil
Fbxl18
Actb
Fscn1
Kdelr2
Daglb
Rac1
Usp42
Bri3
Nptx2
Arpc1b
Wasf3
Rpl21
Rasl11a
Slc7a1
Hmgb1
Medag
N4bp2l1

Col1a2
Pon3
Dync1i1
Asns
C1galt1
Ndufa4
Thsd7a
Cav2
Cav1
Met
Wnt2
Gm20186
Cped1
Wnt16
Fam3c
Prrt4
Calu
Flnc
Kcp
Ube2h
Mest
Gm43154
Gm13848
Akr1b3
Akr1b8
Tmem140
1810058I24Rik
Chrm2
Ptn
Fmc1
Klrg2
Mkrn1
Fam131b
Zyx
Epha1
Atp6v0e2
Rarres2
Tmem176b
Tmem176a
Gpnmb
Mpp6
Gsdme
Osbpl3
Cycs
Hnrnpa2b1
Hoxa5
Hoxa9
Hibadh
Tax1bp1
Creb5

Tril
Scrn1
Mturn
Ggct
Inmt
Adcyap1r1
Lsm5
Pyurf
Fam13a
Krcc1
Chmp3
Tmem150a
Vamp5
Vamp8
Sh2d6
Capg
Retsat
Tgoln1
Tmsb10
Hk2
Htra2
Rtkn
Nagk
Rab11fip5
Nat8f3
Nat8f1
Snrpg
C87436
Anxa4
Antxr1
Cnbp
Mgll
Plxna1
Chchd6
Txnrd3
Klf15
Iqsec1
Fbln2
Tmem43
Ccdc174
Frmd4b
Mitf
Gm765
Foxp1
Eif4e3
Chl1
Gm44040
Bhlhe40
Oxtr
Gt(ROSA)26Sor

Hrh1
Vgll4
Pparg
Raf1
Rpl32
Plxnd1
Cxcl12
Hnrnpf
Csgalnact2
Wnt5b
Atp6v1e1
Usp18
Mfap5
Apobec1
C1rl
C1ra
C1s1
C1rb
Chd4
Gapdh
Cd27
Tnfrsf1a
Cd9
Ntf3
Tspan11
Tspan9
Clec2d
Gabarapl1
Ybx3
Lrp6
Mansc1
Gprc5a
H2afj
Mgp
Eps8
Mgst1
Plekha5
Aebp2
Pde3a
Ldhb
Kcnj8
Bcat1
Kras
Gm15706
Sspn
Caprin2
Etfbkmt
Resf1
Bicd1
Myadm

Tmc4
Rps9
Cdc42ep5
Cox6b2
Tmem238
Rpl28
Ube2s
Isoc2a
Ssc5d
Zim1
Peg3
Rps5
Ube2m
Nop53
Bbc3
Slc1a5
Dact3
Calm3
Ppm1n
Rtn2
Fosb
Apoc1
Apoe
Tomm40
Pvr
Plaur
Zfp575
Rps19
Arhgef1
Pafah1b3
Megf8
B3gnt8
Bckdha
Tgfb1
Axl
Ltbp4
Sertad1
Rps16
Plekhg2
Zfp36
Samd4b
Hnrnpl
Ech1
Eif3k
Kcnk6
Yif1b
Ppp1r14a
Capns1
Tbcb
Sbsn

Dmkn
Krtdap
Cebpg
Cebpa
Slc7a10
Rhpn2
Tshz3
Plekhf1
Pop4
Vstm2b
Josd2
Med25
Rras
Prrg2
Rcn3
Fcgrt
Rpl13a
Ftl1
Bax
Dhdh
Ppp1r15a
Plekha4
Mamstr
Rpl18
Sult2b1
Grin2d
Emp3
Saa1
Ldha
Spty2d1
Htatip2
Cyfip1
Atp10a
Pcsk6
Aldh1a3
Adamts17
Arrdc4
Nr2f2
Rgma
Slco3a1
Anpep
Idh2
Nmb
Sec11a
Pde8a
Rps17
Whamm
Adamtsl3
Stard5
Abhd17c

Arnt2
Fah
Rab38
Tmem135
Fzd4
Prss23
Sytl2
Ccdc90b
Prcp
Ndufc2
Capn5
Acer3
Tsku
Wnt11
Gm45187
Serpinh1
Gdpd5
Rps3
Tpbgl
Gm10605
Slco2b1
P4ha3
Arhgef17
P2ry2
Cavin3
Apbb1
Ilk
Taf10
Eif3f
Lmo1
Rpl27a
Adm
Eif4g2
Galnt18
Parva
Spon1
Rras2
Rps13
Nucb2
Rps15a
Arl6ip1
Iqck
Tmem159
Rbbp6
Il4ra
D430042O09Rik
Eif3c
Sult1a1
Mapk3
Ypel3

Aldoa
Tmem219
Sephs2
Stx4a
Vkorc1
Fus
Pycard
Tgfb1i1
Bag3
Tacc2
Htra1
Oat
Fam53b
Ctbp2
Adam12
Glrx3
Echs1
Cox8b
Ifitm2
Ifitm1
Ifitm3
Rnh1
Rplp2
Tspan4
Ap2a2
Lsp1
Mrpl23
H19
Igf2
Igf2os
Kcnq1ot1
Cdkn1c
Tnfrsf23
Mrgprg
Mrgpre
Ccnd1
Akap12
Syne1
Stxbp5
Rab32
Utrn
Plagl1
Aig1
Adgrg6
Abracl
Gm10827
Nhsl1
Hebp2
Ifngr1
Sgk1

Slc18b1
Vnn1
Epb41l2
Rspo3
Calhm6
Marcks
Gm31378
Lama4
Fyn
Mettl24
Fig4
Mical1
Cd164
Sesn1
Prep
Man1a
Hsf2
Smpdl3a
44084
Sowahc
Mcu
Micu1
Spock2
Psap
Sgpl1
Ppa1
Sar1a
Hk1
Egr2
Arid5b
Rhobtb1
Ank3
Ccdc6
Fam13c
Bicc1
Gnaz
Snrpd3
Ggt5
Ddt
Gstt1
Gstt2
Mif
Smarcb1
Mmp11
Chchd10
S100b
Col6a2
Col6a1
Slc19a1
Col18a1

Adarb1
Dnmt3l
Icosl
Cstb
Prss57
Palm
Plppr3
Prtn3
Cnn2
Cirbp
Efna2
Dazap1
Reep6
Adamtsl5
Lsm7
Gadd45b
Atcayos
Nfic
Aes
Tle2
Glt8d2
Chst11
Aldh1l2
1500009L16Rik
Ckap4
Timp3
Igf1
Nr1h4
Lta4h
Snrpf
Ntn4
Nudt4
Btg1
Dcn
Lum
Atp2b1
Kitl
Ppfia2
Nav3
E2f7
Csrp2
Phlda1
Rap1b
Srgap1
Tmem5
Avpr1a
Slc16a7
Ctdsp2
Ddit3
Gli1

Ndufa4l2
Nab2
Naca
Myl6
Suox
Mmp19
Cd63
Rdh5
Bloc1s1
Itga7
Fcor
Evi5l
Efnb2
Tnfsf13b
Col4a1
Col4a2
Mcf2l
Lamp1
Gas6
Rasa3
Cln8
Arhgef10
Angpt2
Thsd1
Vdac3
Polb
Plat
Sfrp1
Adam9
Htra4
Fgfr1
Zfp703
Rnf122
Rbpms
Saraf
Mfhas1
Fat1
Slc25a4
Casp3
Stox2
Hpgd
Palld
Ddx60
Spock3
Cpe
Psd3
Sh2d4a
Csgalnact1
Lpl
Cope

Uba52
Ell
Gdf15
Lsm4
Jund
Gm3336
Kcnn1
Rpl18a
Plvap
Bst2
Slc27a1
Pgls
Gm10282
Klf2
Hmox1
Ednra
Hhip
Tbc1d9
Dnajb1
Gipc1
Adgre5
Gm26532
Ier2
Nfix
Gcdh
Prdx2
Junb
Dnaja2
Zfp423
Adcy7
Tox3
Rpgrip1l
Irx3
Irx5
Mmp2
Mt2
Mt1
Arl2bp
Ndrg4
Cdh11
Cmtm3
Dync1li2
Pdp2
Rrad
Ces2g
Tppp3
Hsd11b2
Psmb10
Smpd3
Sntb2

Nfat5
Il34
St3gal2
Gcsh
Pkd1l2
Gan
Cmip
Cdh13
Mbtps1
Cotl1
Crispld2
1190005I06Rik
Cox4i1
Map1lc3b
Banp
Zfp469
Aprt
Cbfa2t3
Sult5a1
Dpep1
Fanca
Rhou
Rab4a
Agt
2810004N23Rik
Tomm20
Nrp1
Itgb1
Flnb
Rarb
Rpl15
Nid2
Kcnk5
Anxa7
Camk2g
Plau
Adk
Rps24
Anxa11
Gm9780
Plac9b
Selenok
Mustn1
Spcs1
Nt5dc2
Nisch
Dph3
Vstm4
Arhgap22
Gdf10

Glud1
Fam213a
Gnpnat1
Ddhd1
Bmp4
Cnih1
Samd4
Fbxo34
Ktn1
Klhl33
Pnp
Ndrg2
Slc7a7
Efs
Il17d
Fam124a
Ints6
Ctsb
Fdft1
Fam167a
Scara5
Scara3
Ephx2
Stmn4
Dpysl2
Ebf2
Kctd9
Loxl2
Egr3
Bmp1
Lgi3
Lpar6
Itm2b
Htr2a
Tpt1
Tsc22d1
Dnajc15
Tdrd3
Pcdh20
Pcdh9
Rpl36a-ps1
Kctd12
Spry2
Gpc6
Cldn10
Mbnl2
Dock9
Casp4
Pdgfd
Mmp3

Yap1
Vstm5
Smco4
Olfm2
Col5a3
A230050P20Rik
Dnmt1
Mrpl4
Icam1
Slc44a2
Smarca4
Ldlr
Kank2
Dock6
Tmem205
Epor
Acp5
Bmper
44081
Eepd1
Opcml
Aplp2
Arhgap32
St3gal4
Fez1
Pknox2
Tbrg1
AW551984
Hspa8
Sc5d
Tmem136
D630033O11Rik
Thy1
H2afx
Hmbs
Rps25
Bcl9l
Atp5l
Fxyd6
Sidt2
Sik3
Rexo2
Nnmt
Rpl10-ps3
Pts
Bco2
Il18
Layn
Arhgap20
Kdelc2

Etfa
Peak1
Snx33
1700017B05Rik
Rpp25
Islr
Islr2
Loxl1
Insyn1
Cd276
Nptn
Rec114
Neo1
Hexa
Thsd4
Larp6
Glce
Anp32a
Coro2b
Calml4
Rpl4
Igdcc4
Cilp
Rasl12
Plekho2
Oaz2
Ppib
Rab8b
Tln2
Ice2
Anxa2
Gtf2a2
Myo1e
Adam10
BC065403
Prtg
Myo5a
Lysmd2
Scg3
Cox7a2
Hmgn3
Bckdhb
Ibtk
Pgm3
Snap91
Tbx18
Nt5e
Tmed3
Plscr1
Pcolce2

Spsb4
Rbp1
Ackr4
Ppm1m
Alas1
Rpl29
Abhd14b
Pcbp4
Parp3
Sema3b
Mst1r
Gmppb
Tcta
Rhoa
Gpx1
Lamb2
Impdh2
Wdr6
Pfkfb4
Ccdc12
Pth1r
Rps27rt
Stt3b
Tgfbr2
Itga9
Acaa1b
Myd88
Eif1b
Rpl14
Sec22c
Zfp651
Higd1a
Ackr2
Snrk
Zdhhc3
Clec3b
Tmem158
Lars2
Limd1
Selenom
Tcn2
Castor1
Rasl10a
Kremen1
Znrf3
Aebp1
Ppia
H2afv
Myo1g
Ccm2

Igfbp3
Tns3
Ddc
Grb10
Actr2
Slc1a4
Lgalsl
Peli1
Ugp2
Ehbp1
Efemp1
Rps27a
Sptbn1
Stc2
Rhbdf1
Fbxw11
Fgf18
Slit3
Ccng1
Gabrb2
Pttg1
Adra1b
Rnf145
Ebf1
Clint1
Adam19
Irgm1
Ifi47
Cnot6
Gfpt2
Sqstm1
Clk4
Skp1a
Irf1
Gm12216
Slc22a4
P4ha2
Anxa6
Slc36a2
Sparc
Atox1
G3bp1
Glra1
Sap30l
Igtp
Sh3bp5l
Hist3h2a
Guk1
Alkbh5
Flii

Shmt1
Map2k3
Mfap4
Ulk2
Adora2b
Ncor1
Cenpv
Ubb
Trim16
Hs3st3b1
Gas7
Ntn1
Ccdc42os
Ccdc42
Myh10
Ndel1
Rpl26
Rangrf
Tmem107
Trp53
Mpdu1
Cd68
Eif4a1
Fgf11
Plscr3
Kctd11
Gabarap
Dlg4
Cxcl16
Rnf167
Rabep1
C1qbp
Txndc17
Med31
Smtnl2
Camkk1
Shpk
Rtn4rl1
Serpinf1
Tlcd2
Ywhae
Tusc5
Ssh2
Git1
Traf4
Rpl23a
Rab34
Proca1
Vtn
Ift20

Lyrm9
Lgals9
Ksr1
Ccl11
Ccl8
Slfn5
Rasl10b
Gm11444
44078
Scpep1
Trim25
Tmem100
Nme2
Spag9
Tob1
Cacna1g
Acsf2
Col1a1
Samd14
Ngfr
Phb
Abi3
Gngt2
Cbx1
Copz2
Cisd3
Rpl23
Rpl19
Stac2
Igfbp4
Krt10
Krt20
Krt23
Krt19
Eif1
P3h4
Stat3
Cavin1
Ramp2
Wnk4
Aoc3
Vat1
Tmem106a
Arl4d
Meox1
Grn
Itga2b
Hexim1
1700023F06Rik
Nsf

Arf2
Itgb3
Tanc2
Cyb561
Limd2
Ddx5
Cep95
Smurf2
Gm11713
Prkca
Fam20a
Abca8a
Abca5
Ttyh2
Slc9a3r1
Fdxr
Fads6
Cdr2l
Jpt1
Sumo2
Mif4gd
H3f3b
Trim47
Acox1
Ubald2
Cygb
Prcd
Jmjd6
Srsf2
Sec14l1
44083
Tnrc6c
Socs3
Timp2
Cep295nl
Lgals3bp
C1qtnf1
Gm11747
Engase
Gaa
Baiap2
Slc38a10
Actg1
Pcyt2
Pycr1
Rac3
Metrnl
Gdi2
Akr1c14
Akr1c18

Klf6
Chrm3
Nid1
Rala
Epdr1
Sfrp4
Hist1h2bc
Hfe
Cmah
Acot13
Tdp2
Gm11361
Dusp22
Irf4
Gmds
Serpinb1a
Serpinb6a
1110046J04Rik
Tubb2b
Psmg4
Pxdc1
Bmp6
Txndc5
Edn1
Nol7
Dtnbp1
Atxn1
Dek
Id4
Ecm2
Aspn
Omd
Ogn
S1pr3
Sema4d
Sfxn1
4833439L19Rik
Cltb
Tspan17
Prelid1
Dbn1
Pdlim7
Pitx1
Tgfbi
Hnrnpa0
Idnk
Gkap1
Hnrnpk
Ntrk2
Gas1

Ctsl
Adcy2
Srd5a1
Adamts16
Mrpl36
Clptm1l
Ftl1-ps1
Cast
Ell2
A830082K12Rik
Mef2c
Cox7c
Vcan
Rps23
Ssbp2
Serinc5
Thbs4
Lhfpl2
Arhgef28
Btf3
Map1b
Serf1
Pik3r1
Rnf180
Kif2a
Elovl7
Plpp1
Fst
Itga2
Fgf10
Klhl29
2810032G03Rik
Sdc1
Gm46323
Smc6
Vsnl1
Fam49a
Lpin1
Rock2
Adam17
Id2
Rps7
Sntg2
Alkal2
Prkar2b
Gdap10
Twist1
Hdac9
Meox2
Agmo

Etv1
Arl4a
Gm17056
Dock4
Dnajb9
Stxbp6
Nova1
Npas3
Nfkbia
Trappc6b
Fkbp3
Rps29
Mgat2
Sav1
Nin
Gm32219
Frmd6
Dact1
4930404H11Rik
Pcnx4
Ppm1a
Tmem30b
Hif1a
Wdr89
Rab15
Max
Gphn
Zfp36l1
Actn1
Galnt16
Srsf5
Smoc1
Rbm25
Aldh6a1
Npc2
Pgf
Tmed10
Fos
Snw1
Ston2
Flrt2
Zc3h14
Calm1
Tc2n
Fbln5
Rin3
Lgmn
Ifi27
Serpina3c
Serpina3n

Gsc
Gm28875
Evl
Wars
Dlk1
Meg3
B830012L14Rik
Dync1h1
Hsp90aa1
Gm266
Ckb
Inf2
Siva1
Ahnak2
Pacs2
Mta1
Crip2
Crip1
Esyt2
Selenop
Rpl37
Osmr
Lifr
Egflam
Slc1a3
Prlr
Dnajc21
Adamts12
Tars
Basp1
Trio
Dap
Cmbl
Snhg18
Cpq
Rpl30
Nipal2
Osr2
Cox6c
Rnf19a
Ncald
Gm49313
Klf10
Cthrc1
Zfpm2
Angpt1
Emc2
Eif3h
Ext1
Tnfrsf11b

Nov
Enpp2
Deptor
Col14a1
Has2
Tmem65
Ndufb9
Mtss1
Asap1
Slc45a4
Ly6e
Ly6a
Ly6c1
Ly6h
Eef1d
Grina
Cyc1
Zfp251
Rpl8
Tst
Kctd17
C1qtnf6
Card10
Cdc42ep1
Triobp
H1f0
Tmem184b
Csnk1e
Apobec3
Rpl3
Atf4
Tob2
Pmm1
Snu13
Srebf2
Smdt1
Ndufa6
Cyp2d22
Cyb5r3
Tspo
Prr5
Fbln1
Pim3
Ttll8
Mapk12
Slc2a13
Prickle1
Slc38a2
Slc38a4
Rapgef3

Hdac7
Tmem106c
Ccnt1
Fkbp11
Arf3
Wnt10b
Wnt1
Tuba1a
Cox14
Mettl7a1
Grasp
Krt80
Igfbp6
Csad
Rarg
Prr13
Hoxc10
Hoxc8
Hoxc6
Hoxc4
Hnrnpa1
Zfp385a
Ppp1r1a
Vasn
Rogdi
Ubn1
Ppl
Carhsp1
Rpl39l
Emp2
Litaf
Snai2
Fgd4
Scarf2
Zdhhc8
Comt
Eif4g1
Chrd
Senp2
Tra2b
Eif4a2
St6gal1
Rtp4
Ccdc50
Fam43a
Ppp1r2
Apod
Pigz
Rpl35a
Heg1

Itgb5
Umps
Pdia5
Dirc2
Parp14
Fstl1
Pla1a
Adprh
Lsamp
Gap43
Zbtb20
Naa50
Boc
Ccdc80
Cd200
Phldb2
Nectin3
Cd47
Alcam
Nfkbiz
Rpl24
Abi3bp
Tmem45a
Tomm70a
Filip1l
Dcbld2
Pros1
Htr1f
Vgll3
Robo2
Nrip1
Btg3
App
Adamts5
Tiam1
Ifnar2
Il10rb
Ifngr2
Son
Mrps6
Slc5a3
Rcan1
Bace2
Tiam2
Gtf2h5
Dynlt1f
Rnaset2b
Fndc1
Rnaset2a
Pde10a

Qk
Agpat4
Airn
Dact2
Smoc2
Thbs2
Lnpep
Has1
Hcfc1r1
Flywch2
Abca3
Eci1
Rab26os
Slc9a3r2
Noxo1
Gnptg
Dusp1
Ergic1
Itpr3
Uqcc2
Rps10
Anks1
AC132460.3
Def6
Rpl10a
Stk38
Srsf3
BC004004
Pi16
Mtch1
Pim1
Mdga1
Zfand3
Rsph1
Notch3
Akap8l
Angptl4
Rps28
BC051226
Zbtb22
Gm19412
Pfdn6
Rps18
H2-K1
Brd2
Psmb9
Psmb8
Tap2
Tnxb
C4b

Cfb
C2
Clic1
Ddah2
H2-D1
H2-Q4
H2-Q6
H2-Q7
H2-Q10
Ppp1r10
H2-T23
H2-T22
H2-M3
Gm42418
9130008F23Rik
Tnfrsf21
Rcan2
Enpp5
Enpp4
Tmem63b
Vegfa
Mad2l1bp
Gm26785
Cul7
Guca1a
AI661453
Ccnd3
Tomm6
Mdfi
Mocs1
Rftn1
Plcl2
Plin4
Ptprs
Rpl36
Tubb4a
Tnfsf9
C3
Efna5
Fer
Pja2
Ralbp1
Twsg1
Mtcl1
Epb41l3
Emilin2
Clip4
Ypel5
Ltbp1
Crim1

Vit
Qpct
Cyp1b1
Srsf7
Zfp36l2
Epas1
Rhoq
Svil
Zeb1
Fzd8
Colec12
Zfp521
Dtna
Ino80c
Galnt1
Slc39a6
Celf4
Lims2
Nrep
Srp19
Reep5
4933408B17Rik
Egr1
Hspa9
Ecscr
Cxxc5
Sra1
Taf7
BC037039
Diaph1
Spry4
Ppp2r2b
Dpysl3
Eif3j2
Mcc
Tmed7
Eif1a
Prr16
Sncaip
Ppic
Aldh7a1
Lmnb1
Isoc1
Adamts19
Gm4951
F830016B08Rik
Iigp1
Rps14
Arsi
Pdgfrb

Ppargc1b
Csnk1a1
Bvht
Ablim3
Txnl1
Tubb6
Tcf4
Ccdc68
Mbd2
Mex3c
Me2
Mbd1
Rpl17
Rnf165
Atp5a1
Tshz1
Cndp2
Cyb5a
Cbln2
Lrp5
1810055G02Rik
Cdk2ap2
Rhod
Rbm4b
Cd248
Yif1a
Efemp2
Malat1
Cdc42ep2
Capn1
Fau
Atg2a
Ehd1
Rasgrp2
Ppp1r14b
Fkbp2
Cox8a
Rtn3
Pla2g16
Nxf1
Lrrn4cl
Ubxn1
Eef1g
Ahnak
Fth1
Rab3il1
AW112010
Ms4a4d
Dtx4
Glyat

Ostf1
Rorb
Anxa1
Aldh1a1
Zfand5
Gda
Abhd17b
Klf9
Kank1
Vldlr
Ak3
Plgrkt
Il33
Rpl9-ps6
Pten
Fas
Ifit2
Ifit3
Ppp1r3c
43895
Myof
Rbp4
Slc35g1
Plce1
Tbc1d12
Pdlim1
Ubtd1
Pyroxd2
Scd2
Scd1
Sfxn3
Npm3
Kcnip2
Nolc1
Sfxn2
As3mt
Sfr1
Gsto1
Add3
Bbip1
Tcf7l2
Ablim1
Atrnl1
Emx2os
Emx2
E330013P04Rik

Supplementary Table 2: List of differentially expressed genes used for GSEA analysis of *Asc-1* enriched cells of adolescent subcutaneous preadipocytes.

The list contains the names of the 2,442 genes used for GSEA analysis shown in Supplementary Table 1.

Supplementary Table 3: Quantification of metabolites in shScr control and shAsc-1 cell extracts via NMR based metabolomics analysis.

metabolites	shScr	shAsc-1	*P-value*
Lipids	125.3±14.0	527.6±41.3	<0.001
Myo-inositol	66.3±2.5	145.3±8.3	<0.001
ATP and/or ADP	1.5±0.1	2.4±0.0	<0.01
Leucine	51.3±1.0	32.3±0.9	<0.001
Valine	28.9±0.6	16.4±0.3	<0.0001
Isoleucine	26.6±0.5	16.1±0.3	<0.0001
Aspartate	10.9±0.7	16.2±0.9	<0.01
Threonine	36.5±0.3	23.2±0.5	<0.0001
Alanine	130.3±13.8	75.8±1.9	<0.05
Glutamate	113.4±11.7	77.7±2.5	<0.05
Methionine	13.9±0.7	9.8±0.1	<0.01
Tyrosine	11.5±0.4	4.5±0.3	<0.001
Phenylalanine	12.1±0.5	5.0±0.1	<0.001
Glycine	114.1±15.3	50.9±2.4	<0.05
(Phospho)creatine	37.5±1.7	28.9±0.3	<0.01
Choline	24.5±3.0	15.4±0.4	<0.05
Phosphocholine	124.4±13.4	36.2±1.3	<0.01
Glycerophosphocholine	53.7±4.6	55.8±1.1	n.s.
Glutathione	23.5±1.4	11.4±1.7	<0.01
UDP-glucose	11.6±0.2	9.2±0.3	<0.01
Glucose	31.2±7.2	29.9±1.1	n.s.
Lactate	74.8±6.7	30.7±2.3	<0.01
Formate	3.3±0.1	2.7±0.1	<0.05
Fumarate	2.1±0.0	1.4±0.2	<0.05

Supplementary Table 3: Quantification of metabolites in shScr control and shAsc-1 cell extracts via NMR based metabolomics analysis.

Metabolites were relatively quantified by AUC quantification of representative NMR peaks. Data are shown as mean ± SEM, statistics calculated using a two-tailed students t-test.

Supplementary Table 4: Primer Sequences

Gene Name	Fwd sequence 5’-3’	Rev sequence 5’-3’
Fabp4	gatgcctttgtgggaacct	ctgtcgtctgcggtgattt
Asc-1	agtgttccaggacacccttg	gggtggcactcaagaaagag
P2rx5	ctgcagctcaccatcctgt	cactctgcagggaagtgtca
Pat2	gtgccaagaagctgcagag	tgttgcctttgaccagatga
Pgc1α	agccgtgaccactgacaacgag	gctgcatggttctgagtgctaag
Ppary	ccctggcaaagcatttgtat	gaaactggcacccttgaaaa
Prdm16	ccgctgtgatgagtgtgatg	ggacgatcatgtgttgctcc
Tbp	acccttcaccaatgactcctatg	tgactgcagcaaatcgcttgg
Tfam	caggaggcaaaggatgattc	ccaagacttcatttcattgtcg
Ucp1	ctgccaggacagtacccaag	tcagctgttcaaagcacaca
Srr	ggcgcaatcttttcttcaaa	aactacggctttgggcttct
Adiponectin	gatggcactcctggagagaa	tctccaggctctcctttcct
