# Supplementary material for: Asc-1 regulates white versus beige adipocyte fate in a subcutaneous stromal cell population
Source: Nat Commun. 2021 Mar 11;12:1588. doi: 10.1038/s41467-021-21826-9 (PMC7952576; doi:10.1038/s41467-021-21826-9)
Supplement: Supplementary file 2 — Description of Additional Supplementary Files [file 41467_2021_21826_MOESM2_ESM.pdf]

Description of Additional Supplementary Files

Supplementary Movie 1

Sub-confluent shAsc-1 cells were supplemented with 170 nM insulin in regular growth medium. Imaging was initiated 4 hours post seeding and continued for three days. The live cell imaging was performed with the Zeiss Cell Observer microscope using the Zen 2.6 program. Pictures were taken in 10 minute intervals.

Supplementary Data 1

Adolescent gene matrices

Supplementary Data 2

Adult gene matrices

Supplementary Data 3

scRNAseq analysis pipeline
