# Supplementary material for: Asc-1 regulates white versus beige adipocyte fate in a subcutaneous stromal cell population
Source: Nat Commun. 2021 Mar 11;12:1588. doi: 10.1038/s41467-021-21826-9 (PMC7952576; doi:10.1038/s41467-021-21826-9)

asc1_preadipocytes_full_analysis_v2_

July 3, 2020

1 Summary of notebook

In this notebook, we perform the following analysis: (1) Clustering of the cells of young and old mice, annotation of the clusters using marker genes of immune cells and adipocytes. (2) Separate the preadipocytes from the immune cells and identify the mature subpopulation.

2 Load packages and set global variables

[1]:
```
import numpy as np
import scanpy.api as sc
import scipy as sci
import scipy.sparse
import re
import pandas
import matplotlib as mpl
import matplotlib.pyplot as plt
import seaborn as sns
import sys

from matplotlib import colors

import batchglm
import diffxpy.api as de

from beakerx import *

%load_ext autoreload
%autoreload 2

sc.settings.verbosity = 3 # amount of output
dir_in = '/Users/viktorian.miok/Documents/consultation/Altun-Ussar/David/data/'
dir_out = '/Users/viktorian.miok/Documents/consultation/Altun-Ussar/David/
↪results/'
dir_tables = dir_out+'tables/'
sc_settings_figdir = dir_out+'panels/'
sc_settings_writedir = dir_out+'anndata/'
sc.logging.print_versions()
```

```
sc.settings.set_figure_params(dpi=80, scanpy=True)
print (sys.version)
```

```
/Users/viktorian.miok/anaconda3/lib/python3.7/site-
packages/scanpy/api/__init__.py:6: FutureWarning:

In a future version of Scanpy, `scanpy.api` will be removed.
Simply use `import scanpy as sc` and `import scanpy.external as sce` instead.

  FutureWarning
```

```
scanpy==1.4.4.post1 anndata==0.6.22.post1 umap==0.3.10 numpy==1.18.4
scipy==1.4.1 pandas==0.25.3 scikit-learn==0.22.1 statsmodels==0.10.2 python-
igraph==0.7.1 louvain==0.6.1
3.7.6 | packaged by conda-forge | (default, Jan  7 2020, 22:05:27)
[Clang 9.0.1 ]
```

[2]:
```
print(de.__version__)
```

```
v0.7.4
```

2.1 Global variables

All embeddings and clusterings can be saved and loaded into this script. Be careful with overwriting cluster caches as soon as cell type annotation has started as cluster labels may be shuffled.

Set whether anndata objects are recomputed or loaded from cache.

[3]:
```
bool_recomp = False
```

Set whether clustering is recomputed or loaded from saved .obs file. Loading makes sense if the clustering changes due to a change in scanpy or one of its dependencies and the number of clusters or the cluster labels change accordingly.

[4]:
```
bool_recluster = False
```

Set whether cluster cache is overwritten. Note that the cache exists for reproducibility of clustering, see above.

[5]:
```
bool_write_cluster_cache = False
```

Set whether to produce plots, set to False for test runs.

[6]:
```
bool_plot = True
```

3 Load data

[7]:
```
if bool_recomp:
    # Count matrix:
    fn_cnts_young = dir_in+'Data S1 adolescent_filtered_gene_bc_matrices_h5.h5'
    adata_young = sc.read_10x_h5(fn_cnts_young)
    fn_cnts_old = dir_in+'Data S2 adult_filtered_gene_bc_matrices_h5.h5'
    adata_old = sc.read_10x_h5(fn_cnts_old)
    adata_raw = adata_young.concatenate([adata_old], batch_key='age',␣
 ↪batch_categories=['young', 'old'])
    sc.write(sc_settings_writedir+'adata_raw.h5ad', adata_raw)
else:
    adata_raw = sc.read(sc_settings_writedir+'adata_raw.h5ad')
```

4 Process data

4.1 Embeddings and clustering

Summary of steps performed here: Only cells with at least 500 UMIs are kept. Counts per cell are cell library depth normalized. The gene (feature) space is reduced with PCA to 50 PCs. A nearest neighbour graph and t-SNE are computed based on the PC space. Cell are clustered with louvain clustering based on the nearest neighbour graph. Graph abstraction is computed based on the louvain clustering.

[8]:
```
if bool_recomp:
    adata_proc = adata_raw.copy()
    sc.pp.filter_cells(adata_proc, min_counts=500)
    sc.pp.normalize_per_cell(adata_proc)
    adata_proc.raw = sc.pp.log1p(adata_proc, copy=True)
    sc.pp.pca(adata_proc, n_comps=50, random_state=0, svd_solver='arpack')
    sc.pp.neighbors(adata_proc, n_neighbors=100, knn=True, method='umap',␣
 ↪n_pcs=50, random_state=0)
    sc.tl.tsne(adata_proc, n_jobs=3)
    sc.tl.umap(adata_proc)
    if bool_recluster==True:
        sc.tl.louvain(adata_proc, resolution=1, flavor='vtraag', random_state=0)
        pandas.DataFrame(adata_proc.obs).to_csv(
            path_or_buf =sc_settings_writedir+"obs_adata_proc.csv")
    else:
        obs = pandas.read_csv(sc_settings_writedir+'obs_adata_proc.csv')
        adata_proc.obs['louvain'] = pandas.Series(obs['louvain'].values,␣
 ↪dtype='category')
    sc.write(sc_settings_writedir+'adata_proc.h5ad', adata_proc)
else:
    adata_proc = sc.read(sc_settings_writedir+'adata_proc.h5ad')
sc.tl.paga(adata_proc)
```

```
running PAGA
    finished: added
    'paga/connectivities', connectivities adjacency (adata.uns)
    'paga/connectivities_tree', connectivities subtree (adata.uns) (0:00:00)
```

Produce some summarizing plots that show the global characteristics of the data.

[9]:
```
#Define a nice colour map for gene expression
colors2 = plt.cm.Reds(np.linspace(0, 1, 128))
colors3 = plt.cm.Greys_r(np.linspace(0.7,0.8,20))
colorsComb = np.vstack([colors3, colors2])
mymap = colors.LinearSegmentedColormap.from_list('my_colormap', colorsComb)
```

[10]:
```
if bool_plot==True:
    sc.pl.tsne(adata_proc, color=['age'], size=5, save="_all_age.pdf")
    sc.pl.tsne(adata_proc, color=['louvain'], size=5, save="_all_louvain.pdf")
    sc.pl.tsne(adata_proc, color=['n_counts'], size=5, save="_all_n_counts.pdf")
    sc.pl.tsne(adata_proc, color=['Pdgfra'], size=5, save="_all_Pdgfra.pdf",␣
 ↪color_map=mymap)
    sc.pl.tsne(adata_proc, color=['Slc7a10'], size=5, save="_all_Slc7a10.pdf",␣
 ↪color_map=mymap)
```

```
WARNING: saving figure to file figures/tsne_all_age.pdf
```

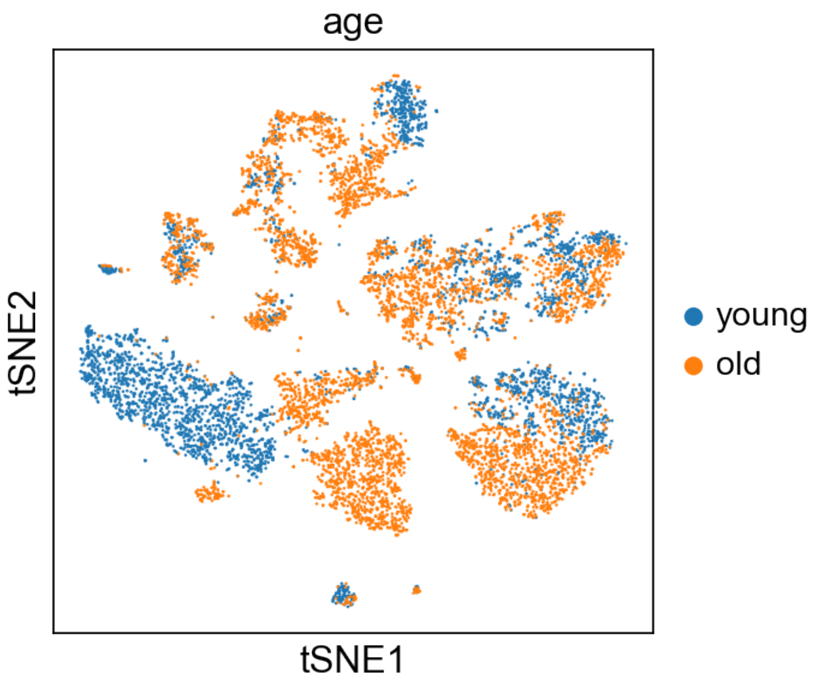


```
WARNING: saving figure to file figures/tsne_all_louvain.pdf
```

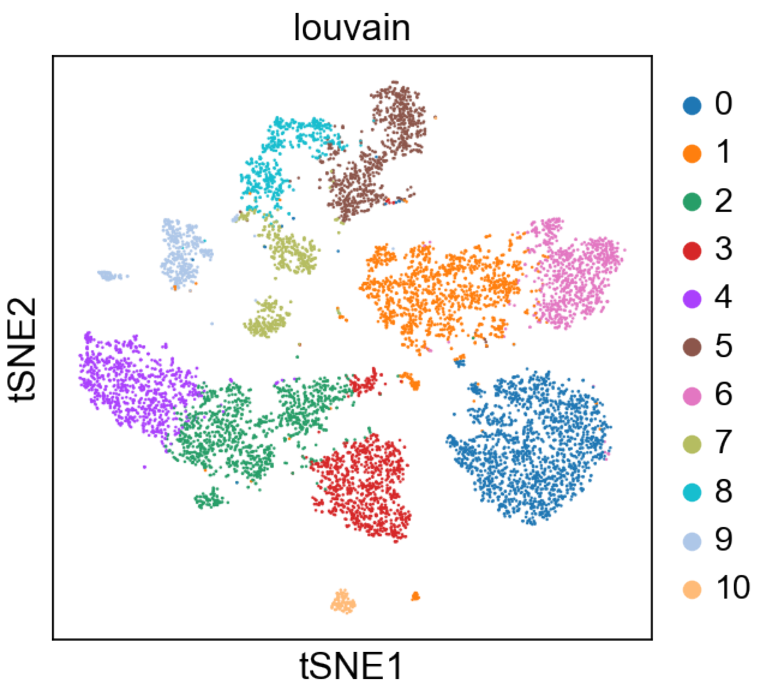


```
WARNING: saving figure to file figures/tsne_all_n_counts.pdf
```

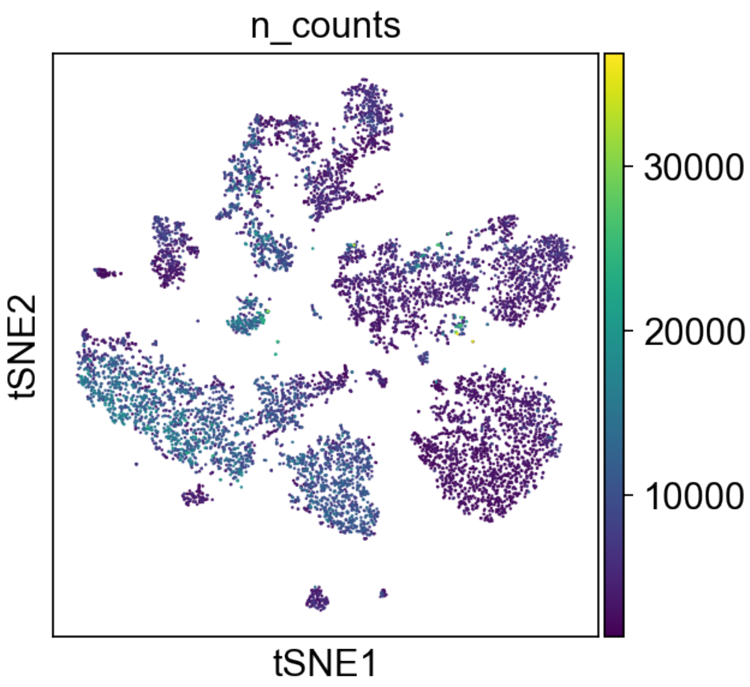

```
WARNING: saving figure to file figures/tsne_all_Pdgfra.pdf
```

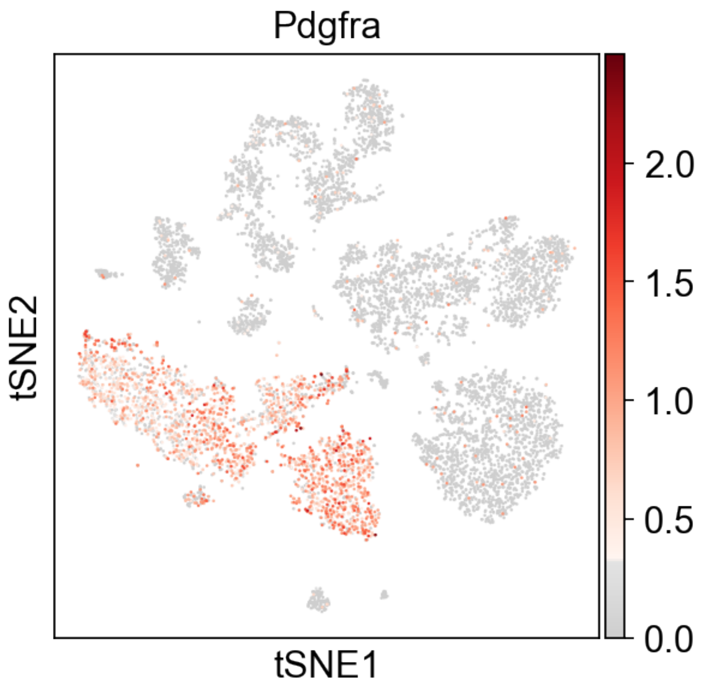


```
WARNING: saving figure to file figures/tsne_all_Slc7a10.pdf
```

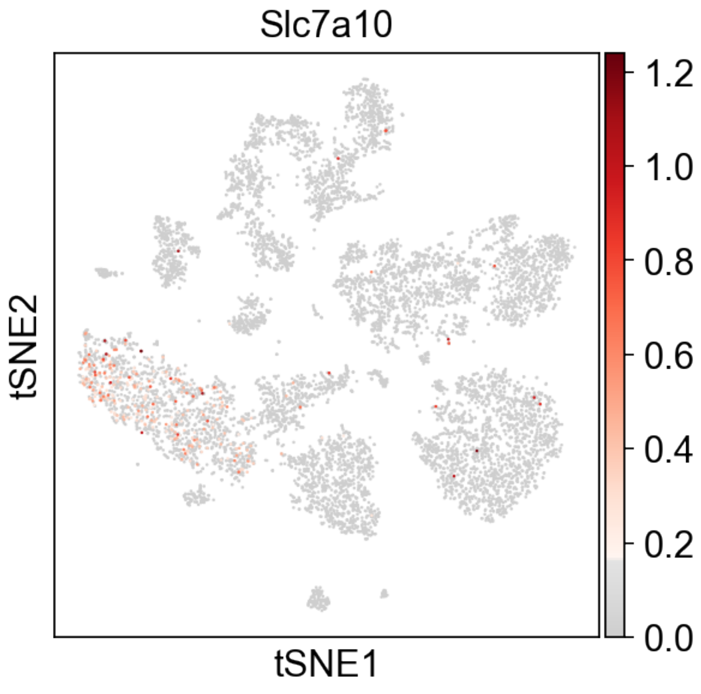

[11]:
```
if bool_plot==True:
    sc.pl.paga(adata_proc, save="_all.pdf")
```

```
--> added 'pos', the PAGA positions (adata.uns['paga'])
WARNING: saving figure to file figures/paga_all.pdf
```

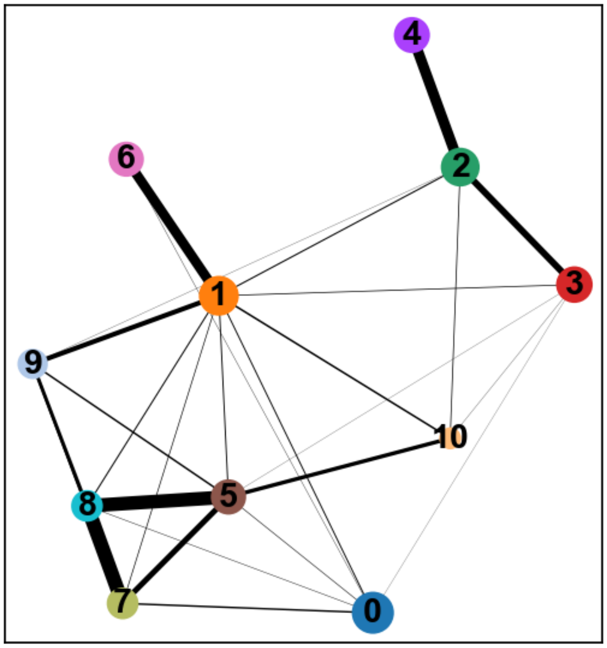

Number of cells in each sample:

[12]:
```
print(np.sum(adata_proc.obs["age"].values == "young"))
```

```
3915
```

[13]:
```
print(np.sum(adata_proc.obs["age"].values == "old"))
```

```
5429
```

[14]:
```
adata_proc.obs['louvain'].value_counts()
```

[14]:
```
0    1733
1    1379
2    1222
3     948
4     892
5     816
```

```
6       799
7       518
8       514
9       421
10      102
Name: louvain, dtype: int64
```

5 Define cell types

5.1 Marker genes

5.1.1 Define marker sets

Define surface marker sets for some of the expected cell types.

[15]:
```
# Leukocyte markers:
leukocyte_markers = ['Ptprc']
tc_markers = ['Cd3d','Cd3e','Cd3g','Cd4']
nk_markers = ['Nkg7','Il2rb','Ncr1','Klrd1','Klrb1b','Klrb1f']
myeloid_markers = ['Cd79a','Itgax','Itgam','Fcgr3','S100a8', 'S100a9']
mp_markers = ['Adgre1','Lyz2']
dc_markers = ['Cd74','Anpep' , 'Cd33', 'Cd80', 'Cd83', 'Cd86']
bc_markers = ['Cd19']
adipocyte_markers =␣
 ↪['Pdgfra','Slc7a10','Pparg','Fermt2','Fbn1','Col4a1','Itgb1','Cd34','Cd24a','Dlk1','Slc7a10
megakaryocyte_markers = ['Ppbp']
erythrocyte_markers = ['Gypa']
go_adip_dev =␣
 ↪['Aacs','Acat1','Arid5b','Arrdc3','Atf2','Bbs4','Bdh1','Csf1','Dgat2','Dyrk1b','Ebf2','Amer
                ␣
 ↪'Id2','Lep','Lrp5','Nampt','Oxct1','Paxip1','Pik3ca','Ppard','Ppargc1a','Rorc','Sh3pxd2b','
                 'Sox8','Spg20','Tbl1xr1','Xbp1']
```

5.1.2 Plotting routines for marker gene sets:

[16]:
```
def plot_violin_marker(adata, markers, save=None, use_raw=True):
    for i in range(len(markers) // 2 + len(markers) % 2):
        if save is not None:
            sc.pl.violin(
                adata,
                groupby='louvain',
                keys=markers[(2*i):np.min([2*(i+1), len(markers)])],
                use_raw=use_raw,
                rotation=90, size=5,
                save=save+"_"+str(i)+".pdf"
            )
        else:
```

```
            sc.pl.violin(
                adata,
                groupby='louvain',
                keys=markers[(2*i):np.min([2*(i+1), len(markers)])],
                use_raw=use_raw,
                rotation=90, size=5,
                save="dasdad"
            )

def plot_tsne_marker(adata, markers, size=5, save=None, use_raw=True):
    for i in range(len(markers) // 2 + len(markers) % 2):
        if save is not None:
            sc.pl.tsne(
                adata,
                color=markers[(2*i):np.min([2*(i+1), len(markers)])],
                size=size,
                use_raw=use_raw,
                color_map=mymap,
                save=save+"_"+str(i)+".pdf"
            )
        else:
            sc.pl.tsne(
                adata,
                color=markers[(2*i):np.min([2*(i+1), len(markers)])],
                size=size,
                use_raw=use_raw
            )
```

5.1.3 Leukocyte markers:

[17]:
```
if bool_plot==True:
    plot_violin_marker(adata_proc, leukocyte_markers,␣
 ↪save="_all_markers_leukocyte")
```

```
WARNING: saving figure to file figures/violin_all_markers_leukocyte_0.pdf
```

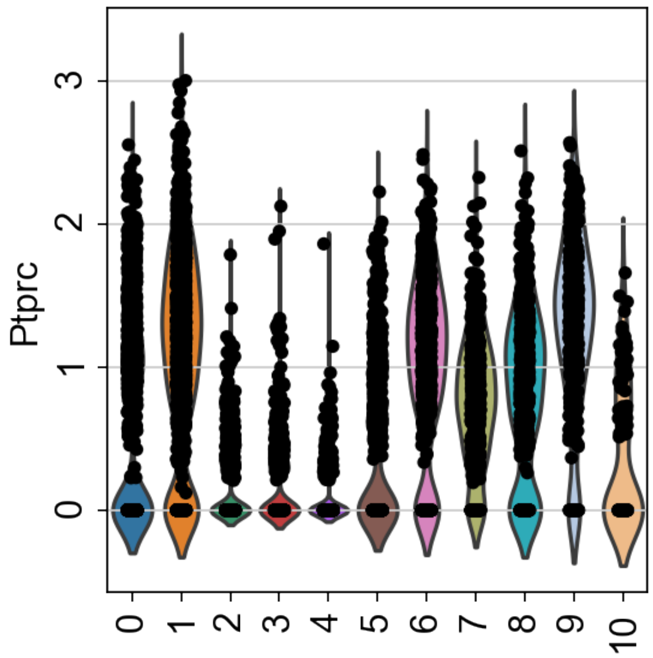

Cluster 1,6,7,8,9 and potentially 0,10 express leukocyte marker Ptprc. These clusters are further validated by leukocyte specific markers below. The remaining clusters 2,3,4 are investigated with non-leukocyte marker sets.

5.1.4 Megakaryocyte markers:

[18]:
```
if bool_plot==True:
    plot_violin_marker(adata_proc, megakaryocyte_markers,␣
↪save="_all_markers_megakaryocytes")
```

```
WARNING: saving figure to file figures/violin_all_markers_megakaryocytes_0.pdf
```

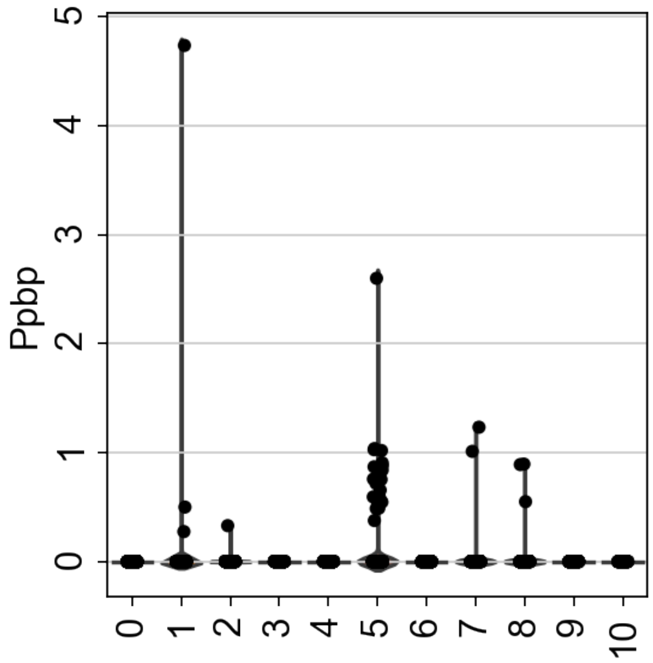

There do not seem to be many megakaryocytes in this data set.

5.1.5 Erythrocyte markers:

[19]:
```
if bool_plot==True:
    plot_violin_marker(adata_proc, erythrocyte_markers,␣
 ↪save="_all_markers_erythrcytes")
```

```
WARNING: saving figure to file figures/violin_all_markers_erythrcytes_0.pdf
```

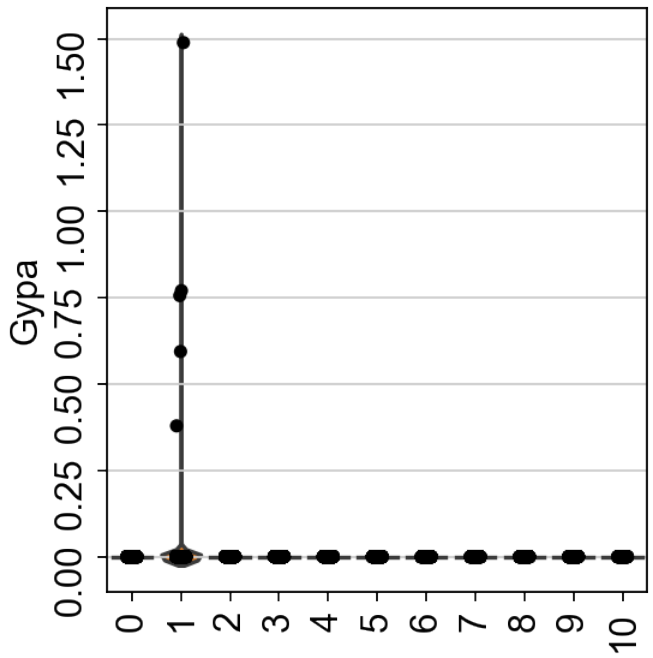

There do not seem to be many erythrocytes in this data set.

5.1.6 Preadipocyte markers:

[20]:
```
if bool_plot==True:
    plot_violin_marker(adata_proc, adipocyte_markers,␣
↪save="_all_markers_preadipocytes")
```

```
WARNING: saving figure to file figures/violin_all_markers_preadipocytes_0.pdf
```

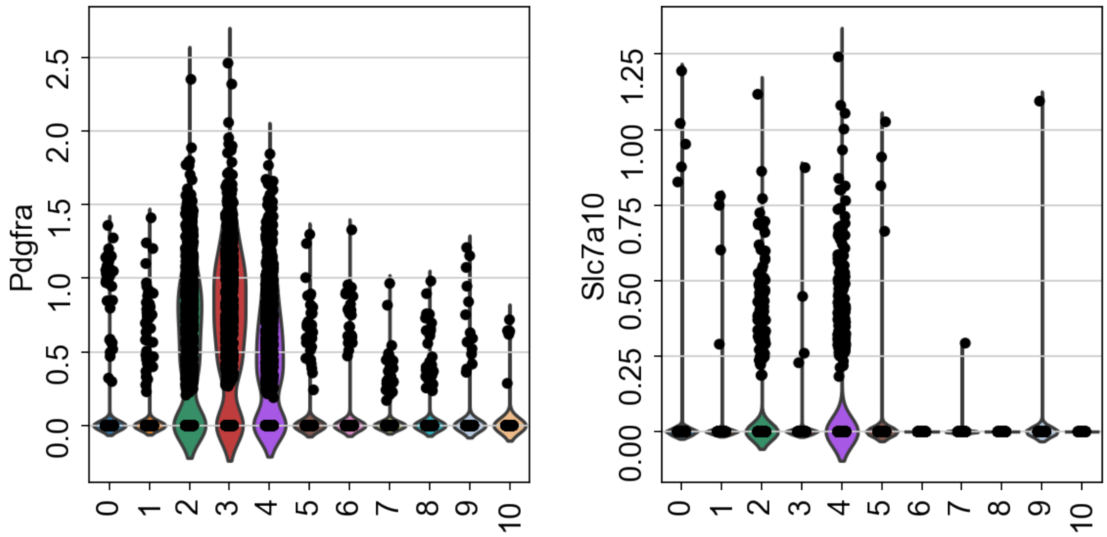

WARNING: saving figure to file figures/violin_all_markers_preadipocytes_1.pdf

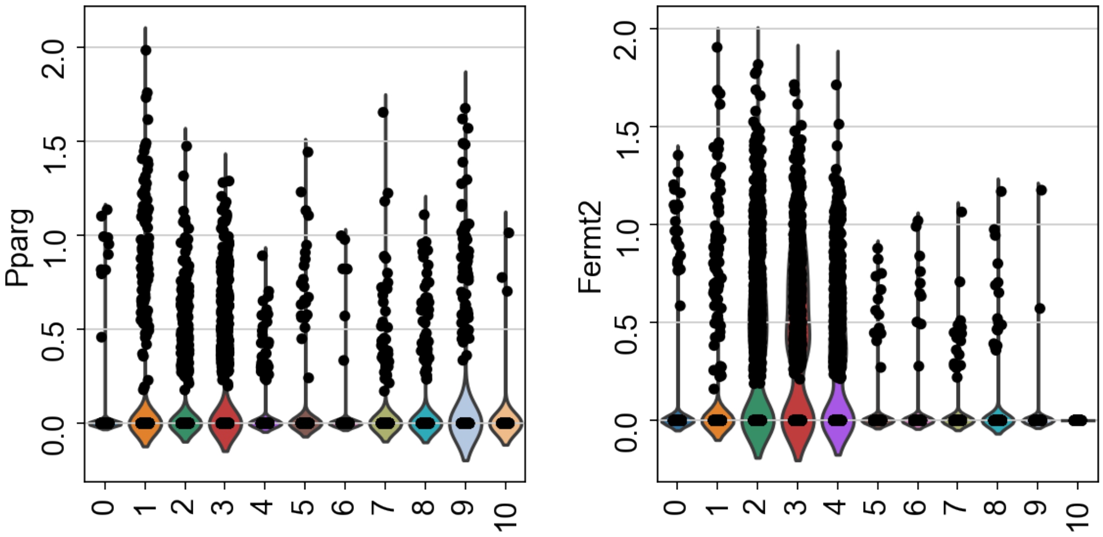

WARNING: saving figure to file figures/violin_all_markers_preadipocytes_2.pdf

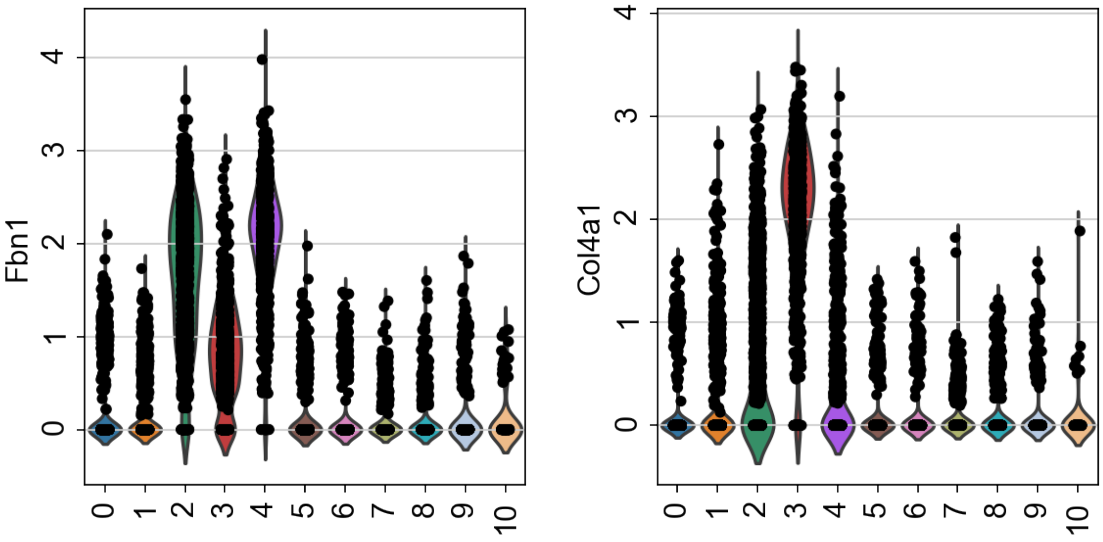

WARNING: saving figure to file figures/violin_all_markers_preadipocytes_3.pdf

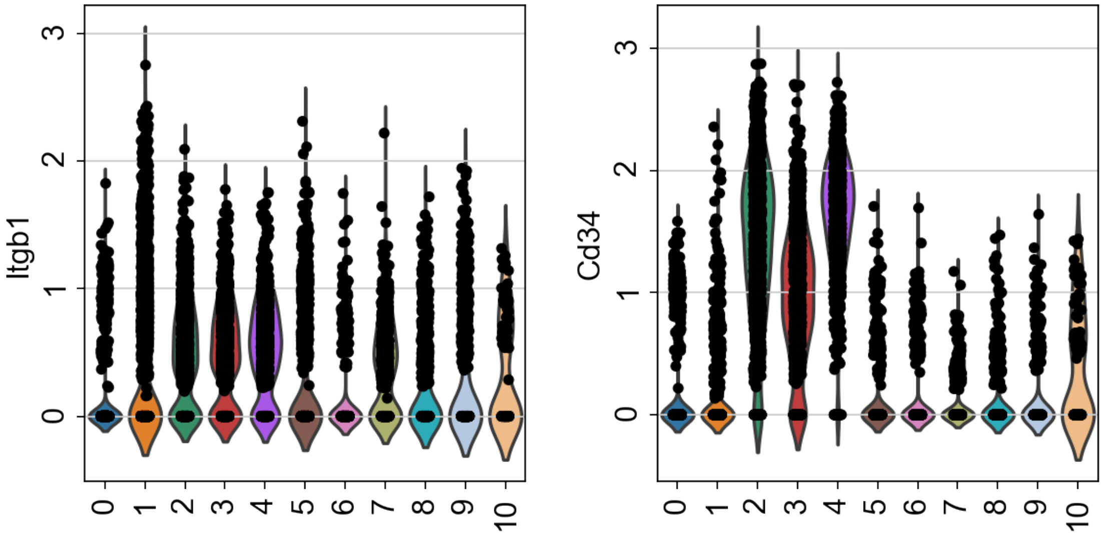

WARNING: saving figure to file figures/violin_all_markers_preadipocytes_4.pdf

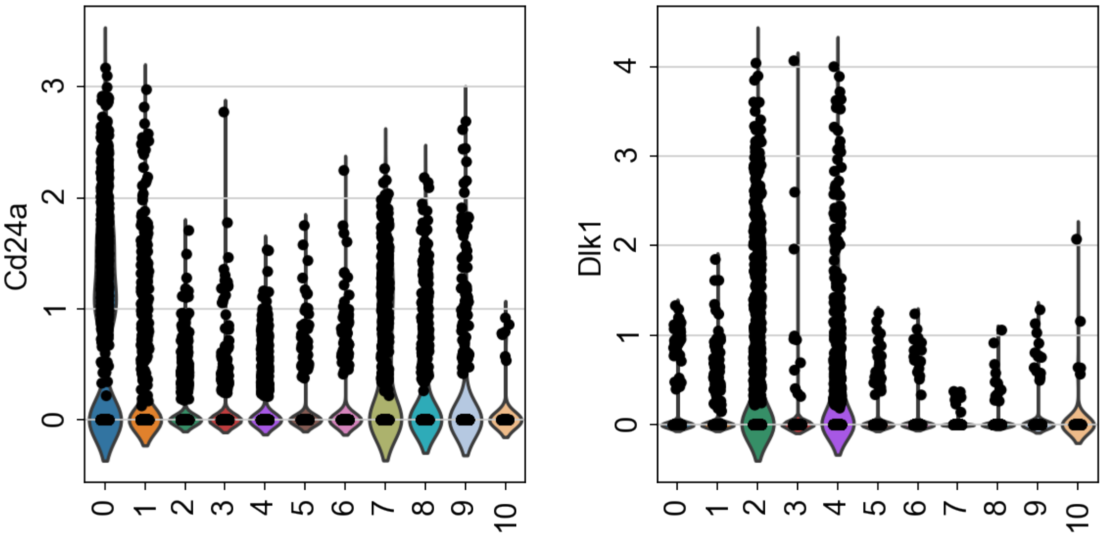

WARNING: saving figure to file figures/violin_all_markers_preadipocytes_5.pdf

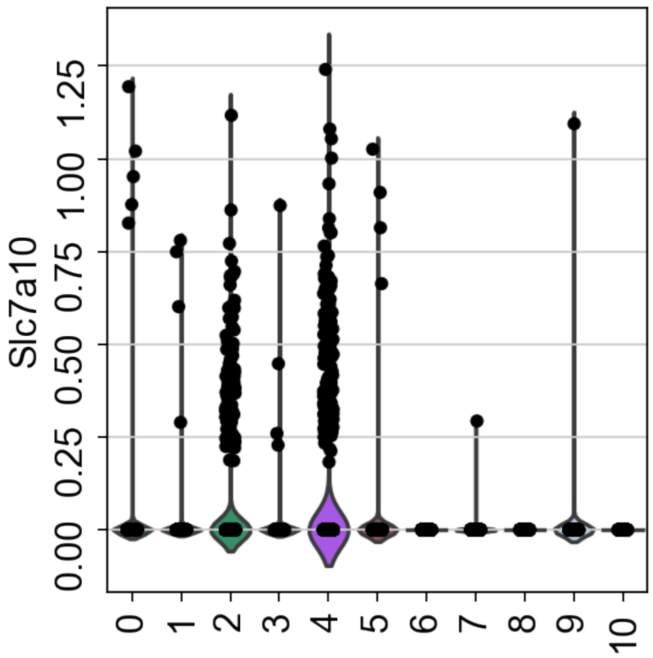

Cluster 2,3,4 express adipocyte markers.

5.1.7 T-cell markers:

[21]:
```
if bool_plot==True:
    plot_violin_marker(adata_proc, tc_markers, save="_all_markers_tcells")
```

WARNING: saving figure to file figures/violin_all_markers_tcells_0.pdf

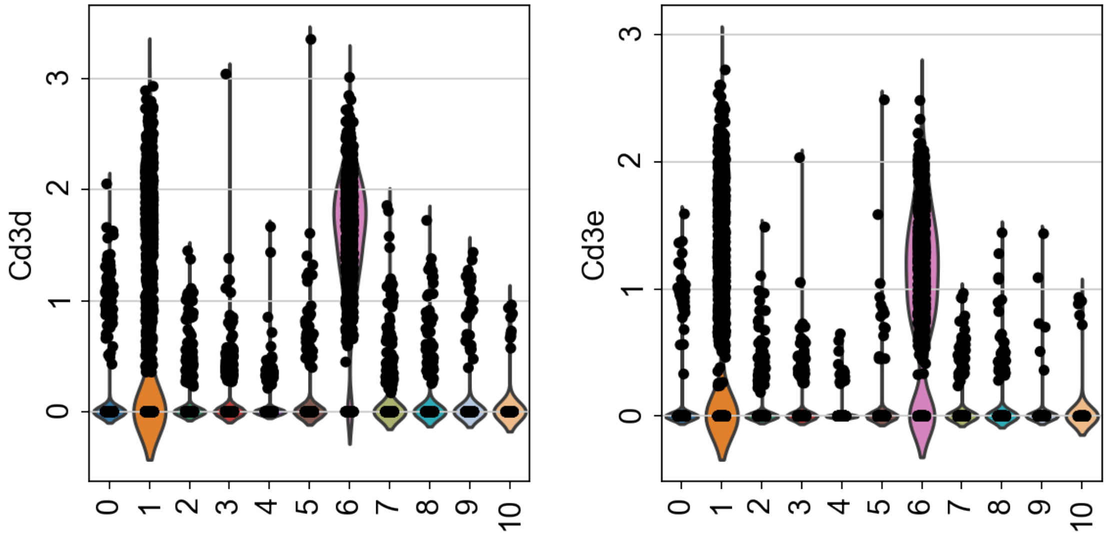

```
WARNING: saving figure to file figures/violin_all_markers_tcells_1.pdf
```

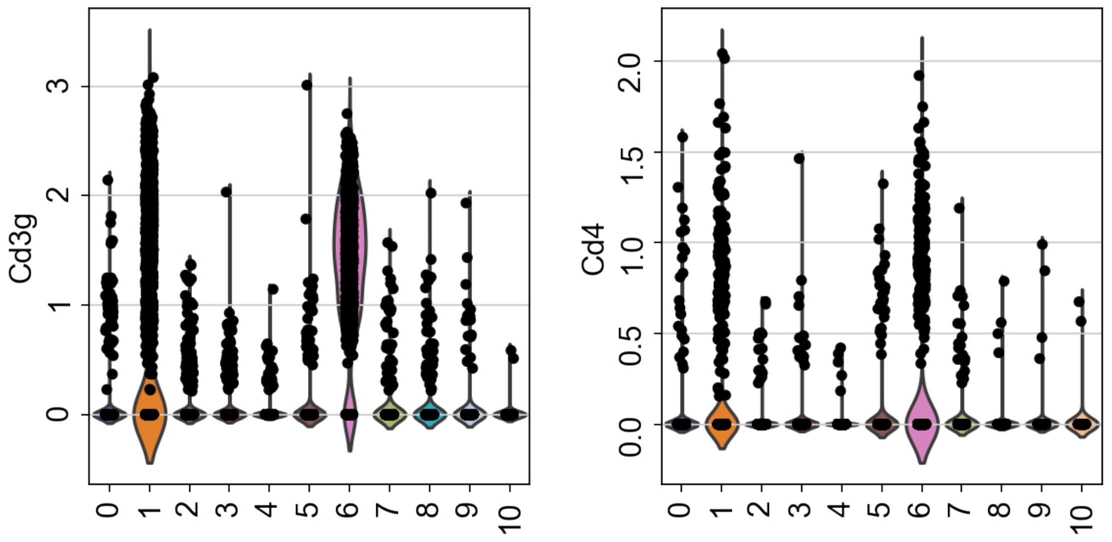

Marker gene expression suggests that cluster 1 and 6 are T-cells, interestingly not Cd4+Cd8+ T-cells it seems as Cd4 expression is low.

5.1.8 Natural killer cell markers:

[22]:
```python
if bool_plot==True:
    plot_violin_marker(adata_proc, nk_markers, save="_all_markers_nk")
```

```
WARNING: saving figure to file figures/violin_all_markers_nk_0.pdf
```

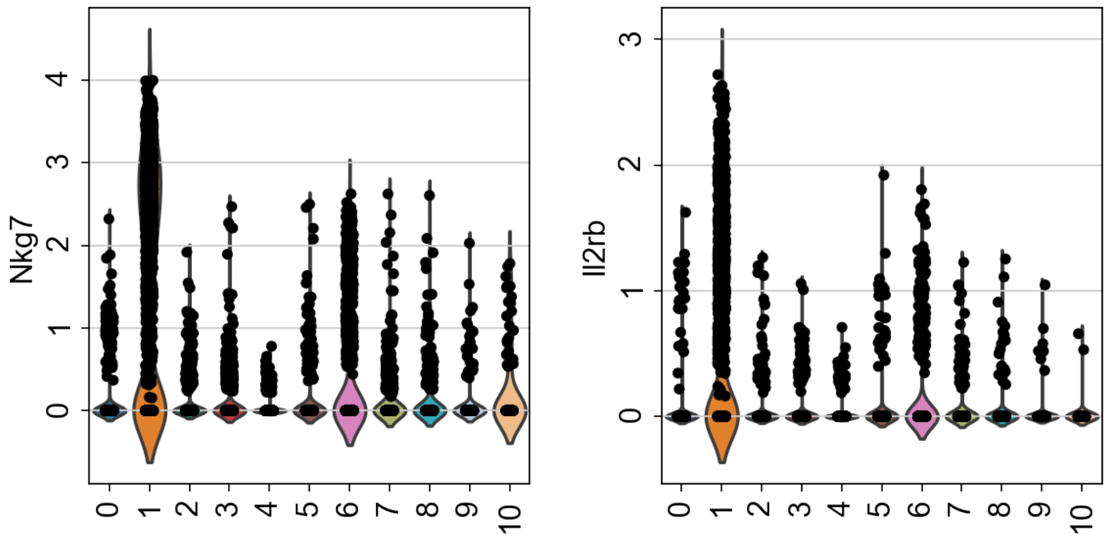

```
WARNING: saving figure to file figures/violin_all_markers_nk_1.pdf
```

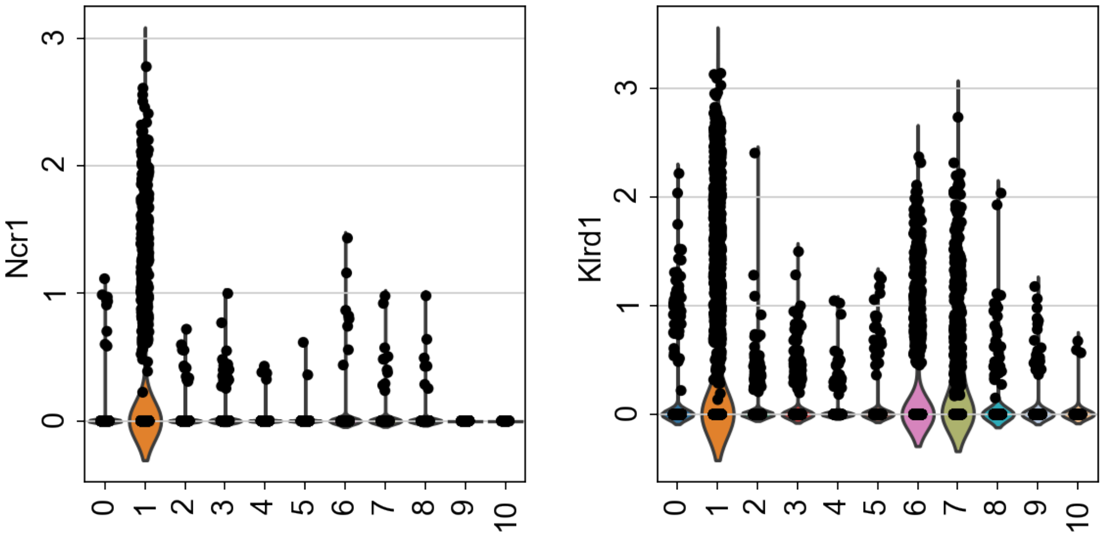


```
WARNING: saving figure to file figures/violin_all_markers_nk_2.pdf
```

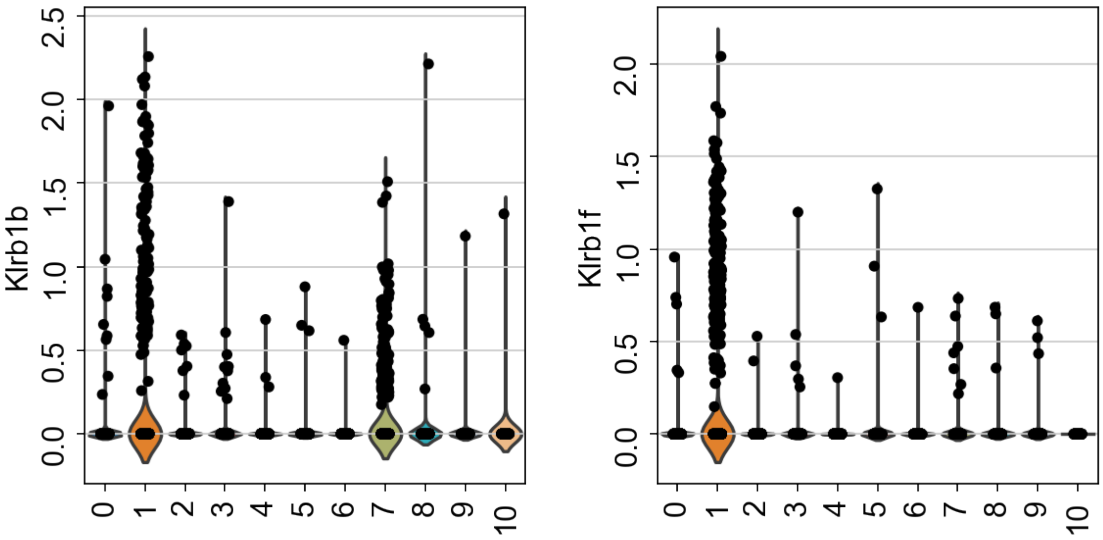

Cluster 1,6,7, have natural killer cell marker gene expression, cluster 1 also expresses Cd3 so it may contain yd-T-cells-?

5.1.9 Myeloid cell markers:

[23]:
```python
if bool_plot==True:
    plot_violin_marker(adata_proc, myeloid_markers, save="_all_markers_myeloid")
```

```
WARNING: saving figure to file figures/violin_all_markers_myeloid_0.pdf
```

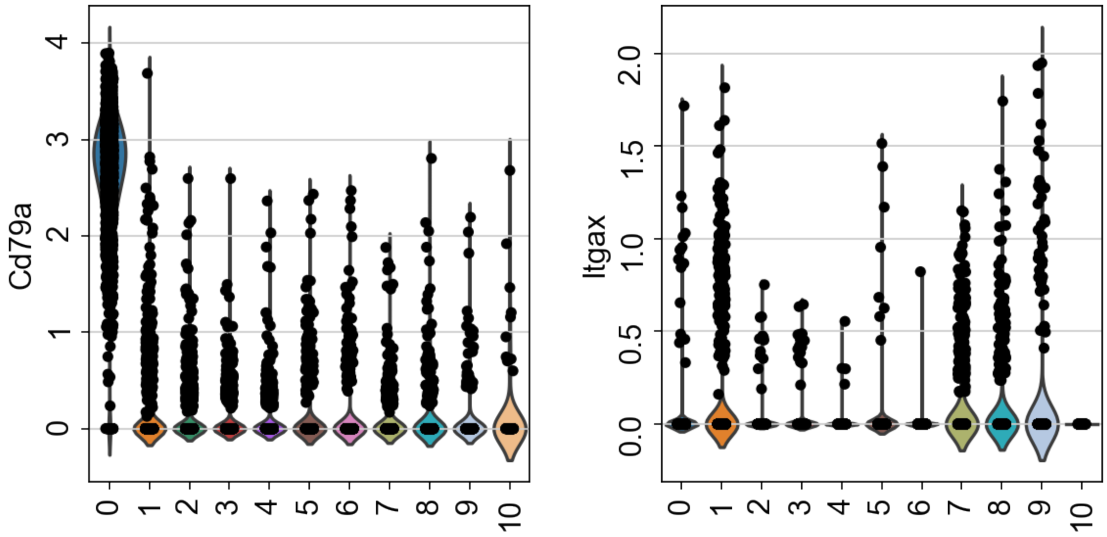


```
WARNING: saving figure to file figures/violin_all_markers_myeloid_1.pdf
```

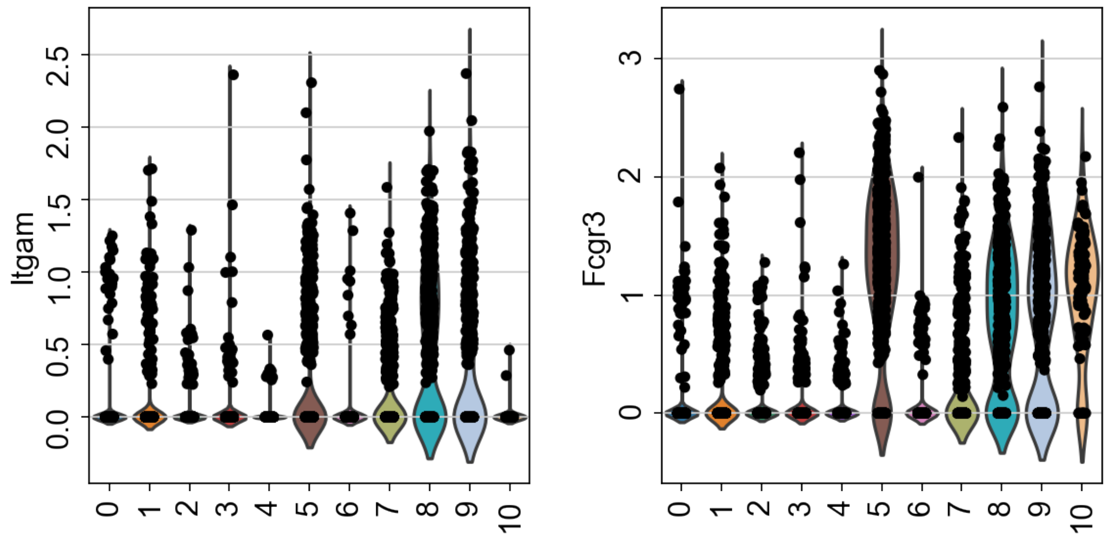


```
WARNING: saving figure to file figures/violin_all_markers_myeloid_2.pdf
```

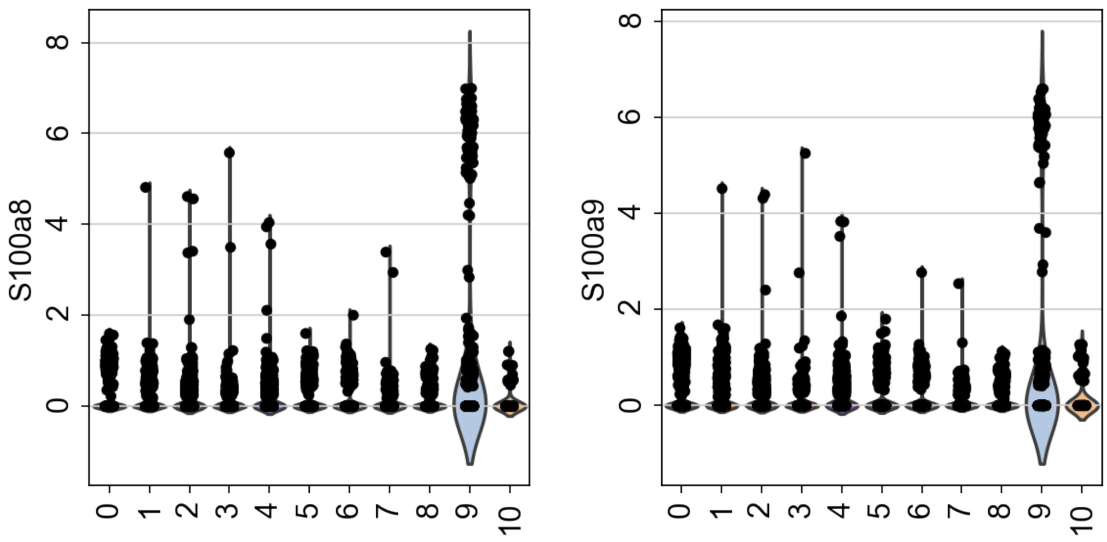

Cluster 5,8,9,10 express myeloid cell marker genes. Cluster 9 seems to have bimodal expression in S100a8 and S100a9 so it may need subclustering to subdivide cell types here.

5.1.10 Macrophage markers:

[24]:
```
if bool_plot==True:
    plot_violin_marker(adata_proc, mp_markers, save="_all_markers_macrophages")
```

```
WARNING: saving figure to file figures/violin_all_markers_macrophages_0.pdf
```

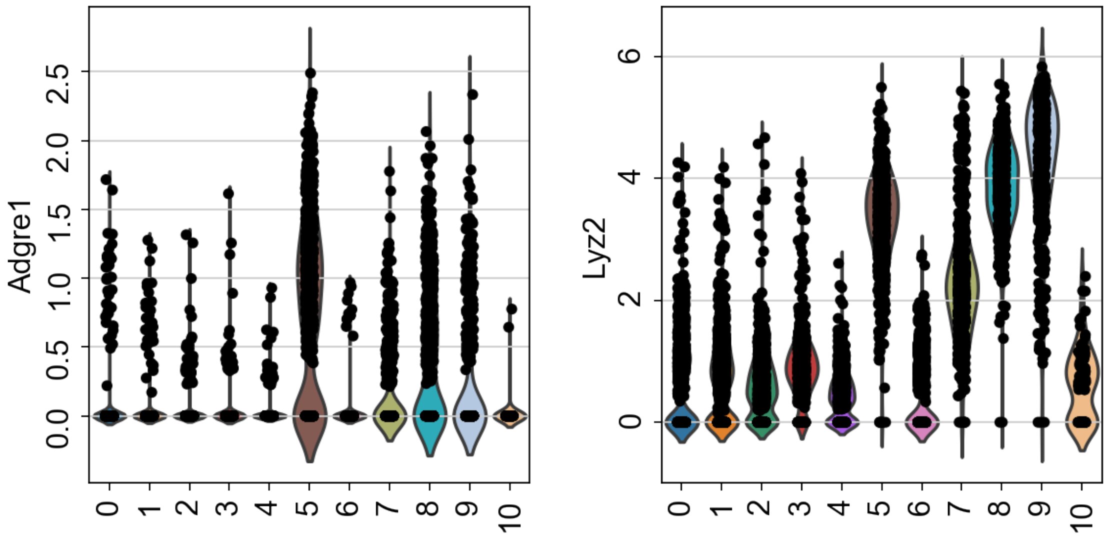

Cluster 5,7,8,9 express macrophage markers, in line with the myeloid cell marker gene expression.

5.1.11 Dendritic cell markers:

[25]:
```
if bool_plot==True:
    plot_violin_marker(adata_proc, dc_markers, save="_all_markers_dendritic")
```

WARNING: saving figure to file figures/violin_all_markers_dendritic_0.pdf

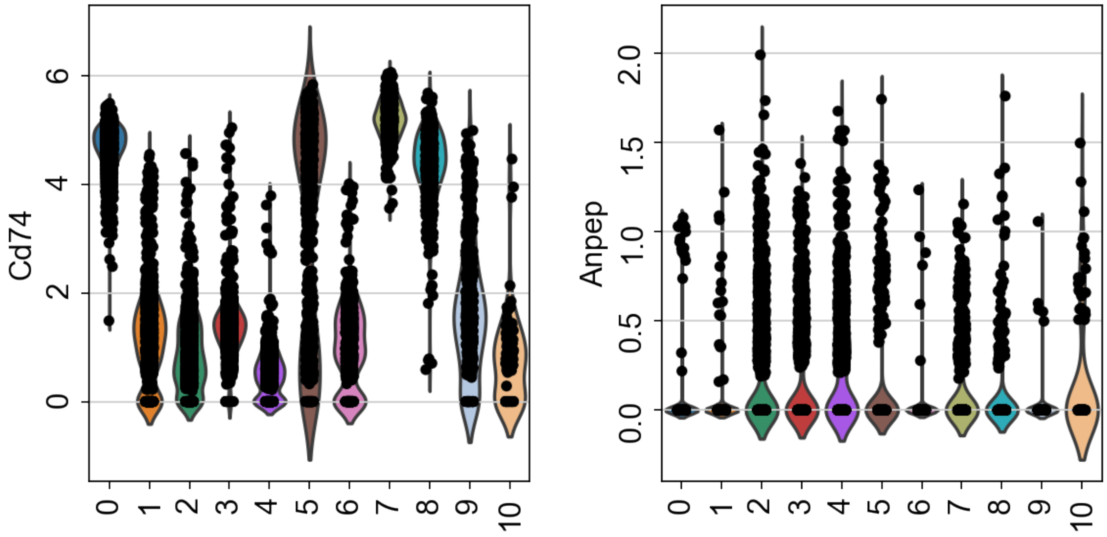

WARNING: saving figure to file figures/violin_all_markers_dendritic_1.pdf

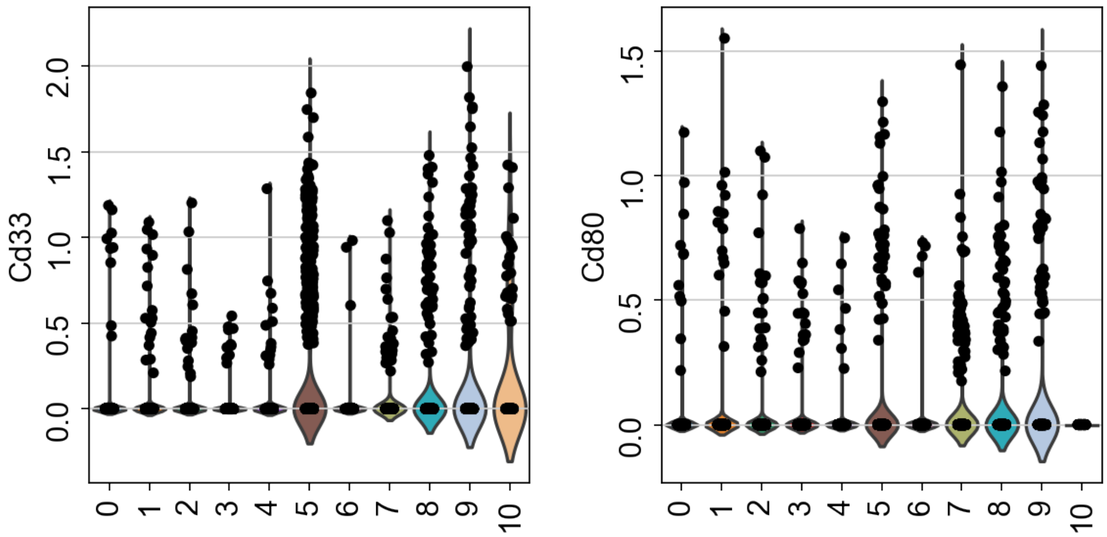

WARNING: saving figure to file figures/violin_all_markers_dendritic_2.pdf

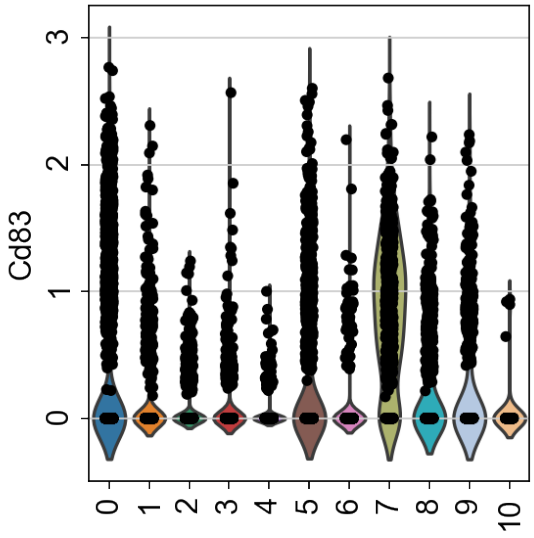

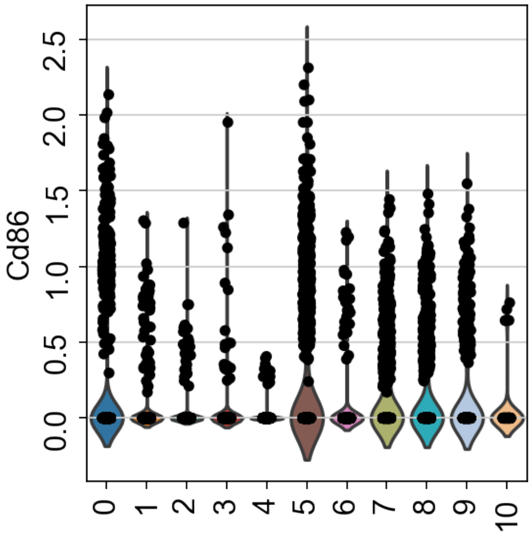

Clusters 0,5,7,8,9 express dendritic cell markers, in line with myeloid marker gene expression, cluster 0,7 could be a non-myeloid dendritic cell.

5.1.12 B-cell markers:

[26]:
```
if bool_plot==True:
    plot_violin_marker(adata_proc, bc_markers, save="_all_markers_bcells")
```

```
WARNING: saving figure to file figures/violin_all_markers_bcells_0.pdf
```

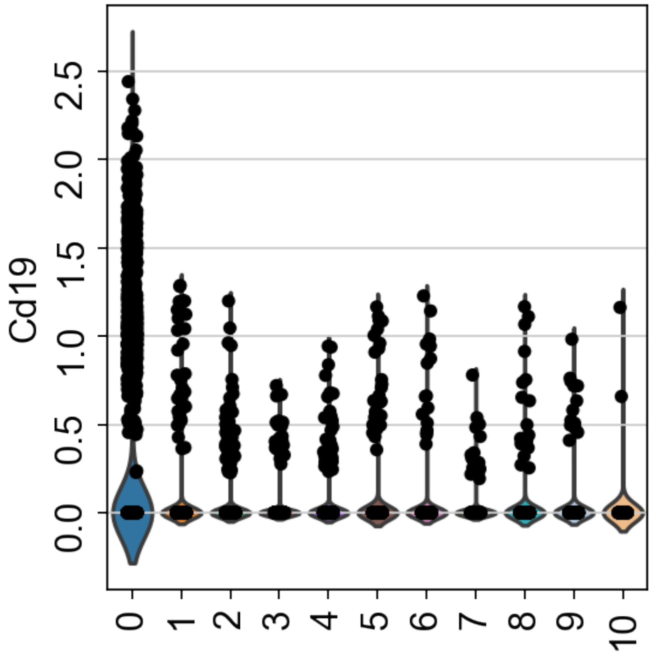

Cluster 0 may contain B-cells.

5.2 Summary heatmap to characterize cell types

Select a few genes to summarize cell type assignments:

[27]:
```
selected_leukocyte_markers = ['Ptprc']
selected_tc_markers = ['Cd3d','Cd3g']
selected_nk_markers = ['Nkg7','Klrd1']
selected_myeloid_markers = ['Fcgr3','S100a8']
selected_mp_markers = ['Adgre1','Lyz2']
selected_dc_markers = ['Cd74','Cd83']
selected_bc_markers = ['Cd19']
selected_adipocyte_markers =␣
↪['Pdgfra','Fbn1','Col4a1','Cd34','Cd24a','Dlk1','Slc7a10']
selected_megakaryocyte_markers = ['Ppbp']
selected_erythrocyte_markers = ['Gypa']
```

[28]:
```
selected_cell_markers = selected_leukocyte_markers + \
selected_megakaryocyte_markers + \
selected_erythrocyte_markers + \
selected_myeloid_markers + \
selected_mp_markers + \
selected_dc_markers + \
selected_bc_markers + \
selected_tc_markers + \
selected_nk_markers + \
selected_adipocyte_markers
```

Only keep markers that occur in data set.

[29]:
```
if bool_plot==True:
    sc.pl.heatmap(
        adata=adata_proc,
        var_names=selected_cell_markers,
        groupby="louvain",
        use_raw=True,
        log=False,
        dendrogram=False,
        var_group_rotation=90,
        show_gene_labels=True,
        show=True,
        save="_all_markers_celltypes.pdf"
    )
```

```
WARNING: saving figure to file figures/heatmap_all_markers_celltypes.pdf
```

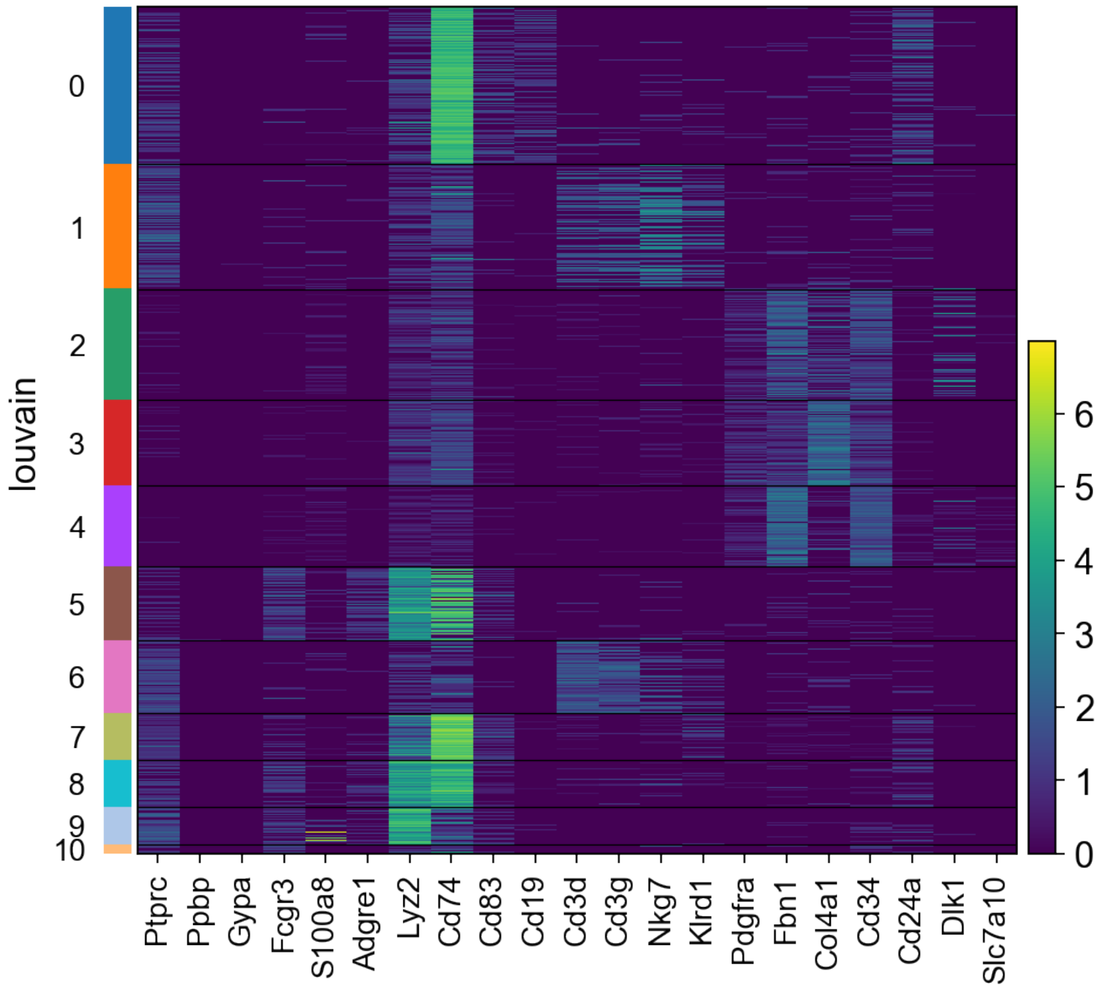

6 Preadipocytes only

6.1 Embedding and clustering

[30]:
```
if bool_recomp==True:
    cell_ids_adip = np.asarray(adata_proc.obs_names)[[x in ['2','3','4']
                                                      for x in np.
 ↪asarray(adata_proc.obs['louvain'].values)]]
    adata_adip = adata_raw[cell_ids_adip,:].copy()
    sc.pp.filter_cells(adata_adip, min_counts=500)
    sc.pp.normalize_per_cell(adata_adip)
    adata_adip.raw = adata_adip.copy()
    sc.pp.log1p(adata_adip)
    sc.pp.pca(adata_adip, n_comps=50, random_state=0, svd_solver='arpack')
```

```
    sc.pp.neighbors(adata_adip, n_neighbors=100, knn=True, method='umap',␣
↪n_pcs=50, random_state=0)
    sc.tl.tsne(adata_adip, n_jobs=3)
    if bool_recluster==True:
        sc.tl.louvain(adata_adip, resolution=1, flavor='vtraag', random_state=0)
        pandas.DataFrame(adata_adip.obs).to_csv(
            path_or_buf=sc_settings_writedir+"obs_adata_adip.csv")
    else:
        obs = pandas.read_csv(sc_settings_writedir+'obs_adata_adip.csv')
        adata_adip.obs['louvain'] = pandas.Series(obs['louvain'].values,␣
↪dtype='category')
    sc.write(sc_settings_writedir+'adata_adip.h5ad', adata_adip)
else:
    adata_adip = sc.read(sc_settings_writedir+'adata_adip.h5ad')
sc.tl.paga(adata_adip)
```

```
running PAGA
    finished: added
    'paga/connectivities', connectivities adjacency (adata.uns)
    'paga/connectivities_tree', connectivities subtree (adata.uns) (0:00:00)
```

[31]:

```
if bool_plot==True:
    sc.pl.tsne(adata_adip, color=['age'], size=20, save="_preadip_age.pdf")
    sc.pl.tsne(adata_adip, color=['louvain'], size=20, save="_preadip_louvain.
↪pdf")
    sc.pl.tsne(adata_adip, color=['n_counts'], size=20, save="_preadip_n_counts.
↪pdf")
```

```
WARNING: saving figure to file figures/tsne_preadip_age.pdf
```

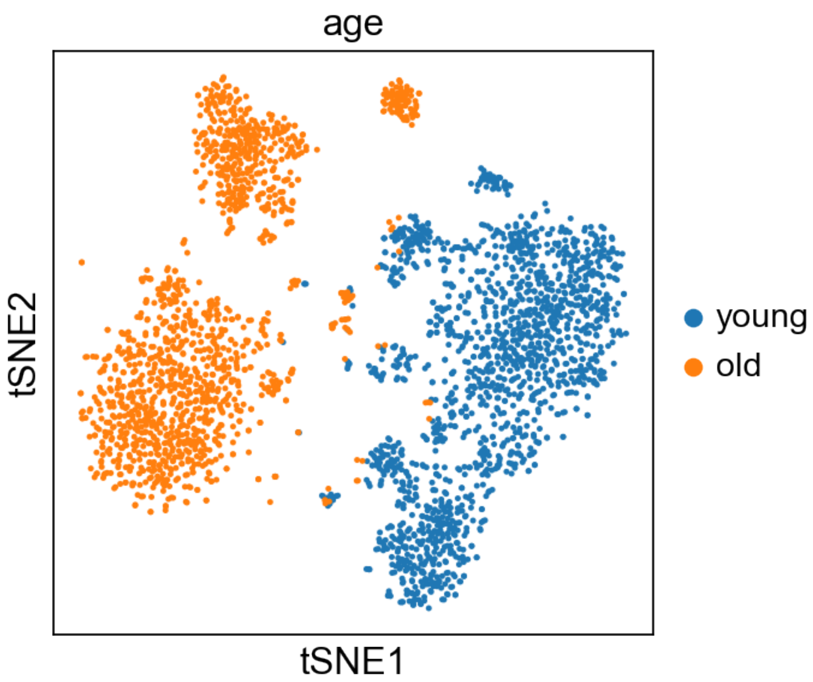


```
WARNING: saving figure to file figures/tsne_preadip_louvain.pdf
```

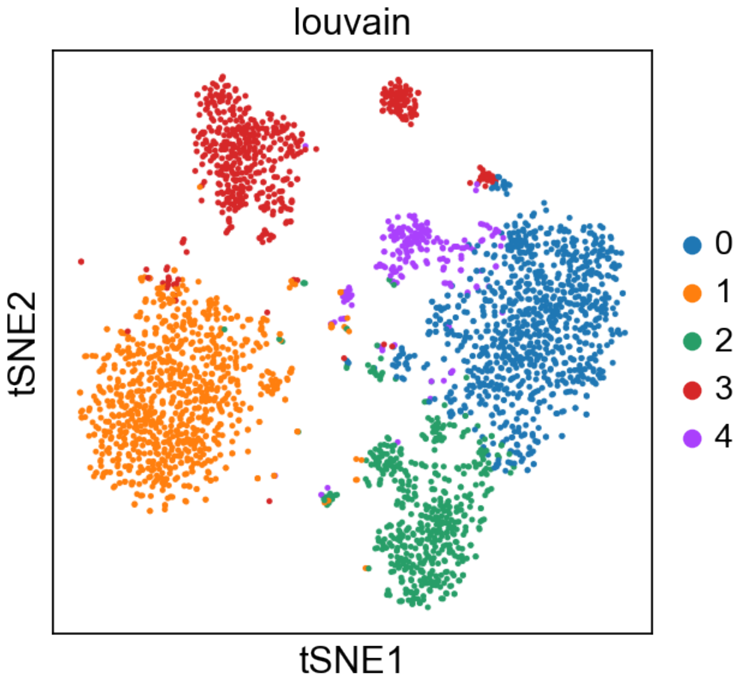

```
WARNING: saving figure to file figures/tsne_preadip_n_counts.pdf
```

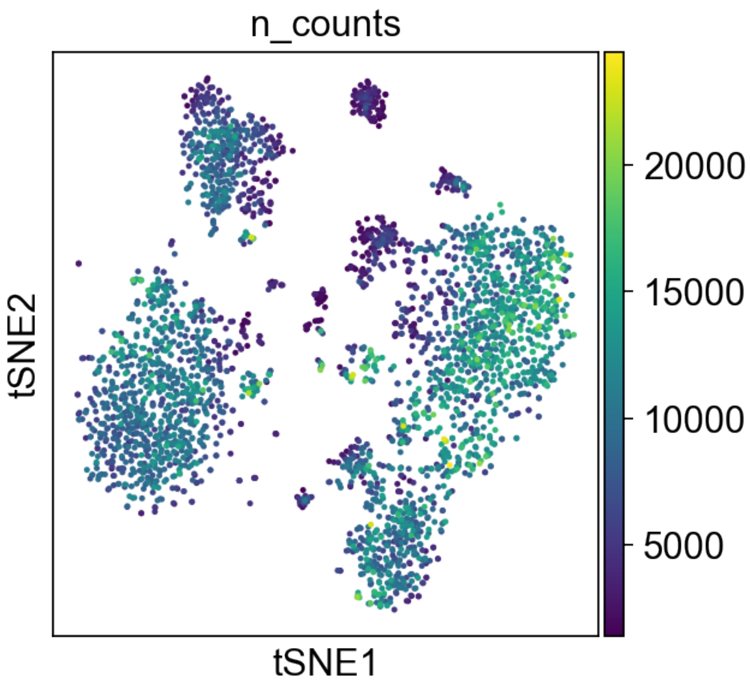

Fairly equal count depth across preadipocytes. Potential batch effect or age effect, indistinguishable in this scenario.

[32]:
```python
if bool_plot==True:
    sc.pl.paga(adata_adip, save="_preadip.pdf")
```

```
--> added 'pos', the PAGA positions (adata.uns['paga'])
WARNING: saving figure to file figures/paga_preadip.pdf
```

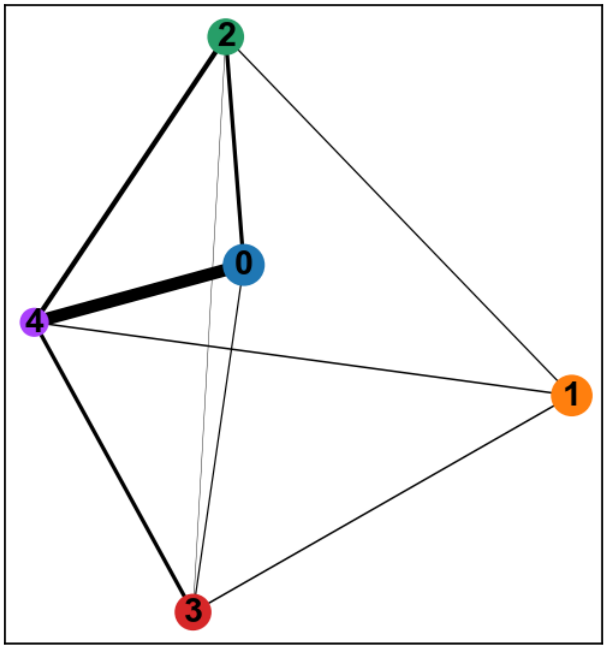

6.2 Marker gene sets

[33]:
```
if bool_plot==True:
    plot_violin_marker(adata_adip, adipocyte_markers,␣
↪save="_preadip_markers_preadipcytes", use_raw=False)
```

```
WARNING: saving figure to file figures/violin_preadip_markers_preadipcytes_0.pdf
```

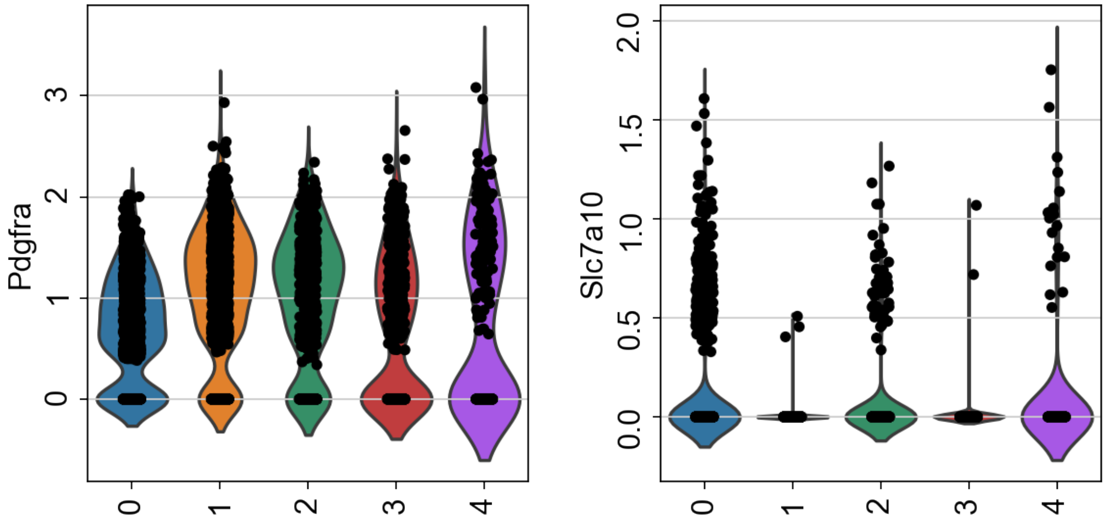

```
WARNING: saving figure to file figures/violin_preadip_markers_preadipcytes_1.pdf
```

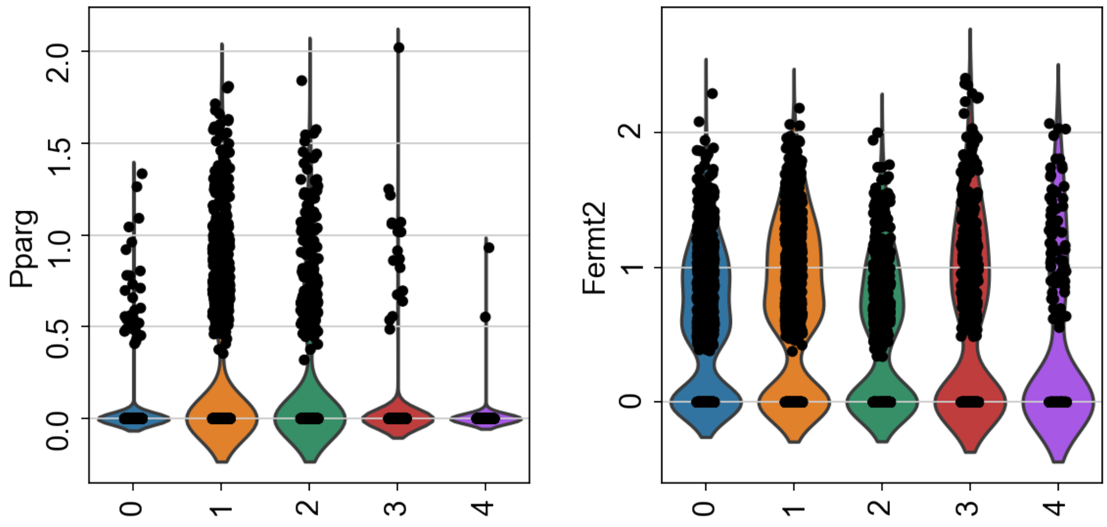


```
WARNING: saving figure to file figures/violin_preadip_markers_preadipcytes_2.pdf
```

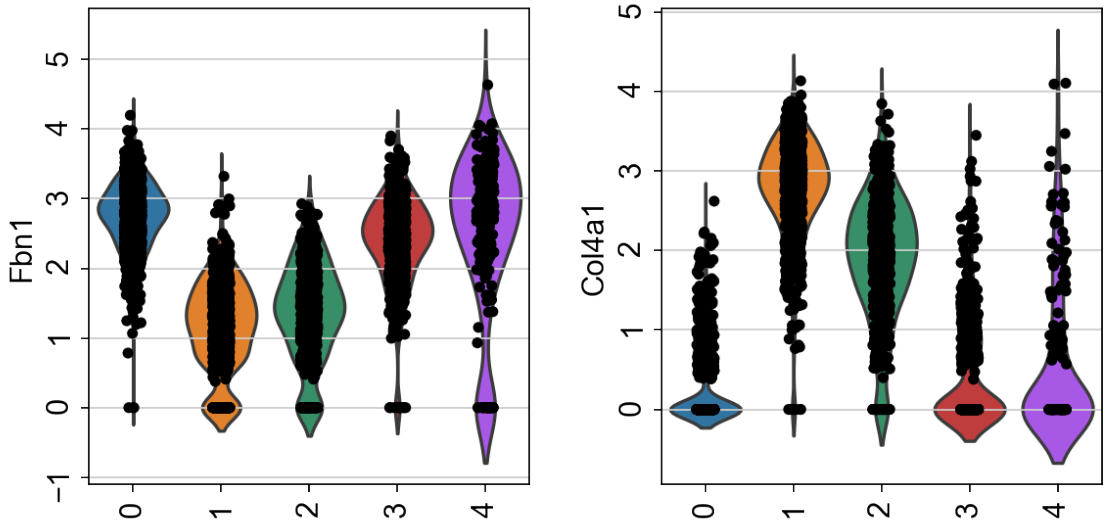


```
WARNING: saving figure to file figures/violin_preadip_markers_preadipcytes_3.pdf
```

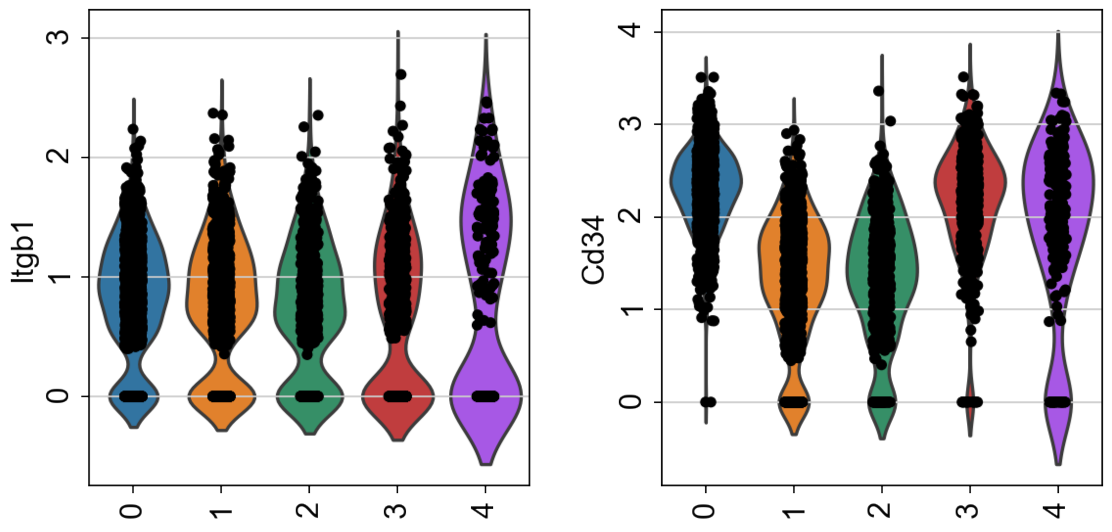

WARNING: saving figure to file figures/violin_preadip_markers_preadipcytes_4.pdf

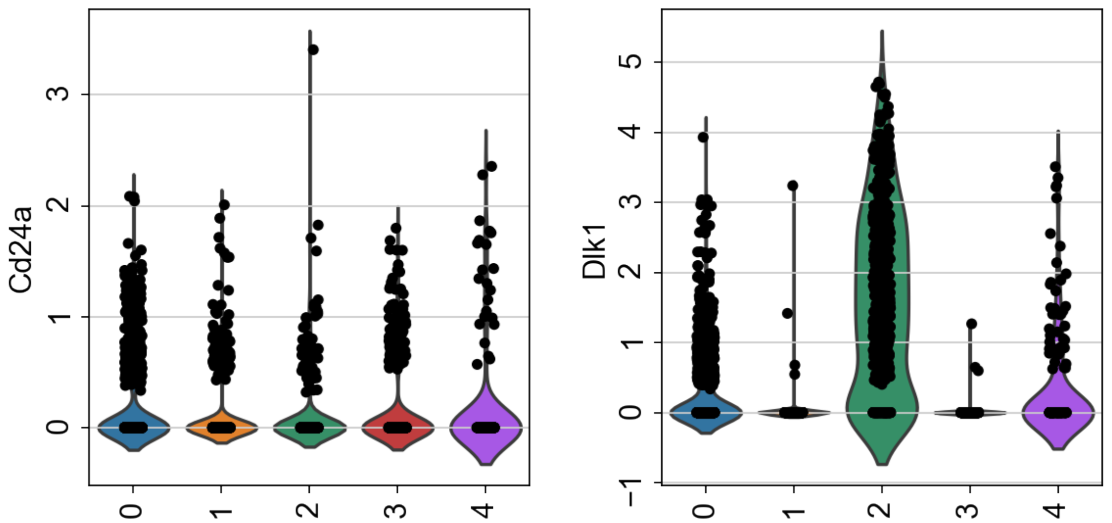

WARNING: saving figure to file figures/violin_preadip_markers_preadipcytes_5.pdf

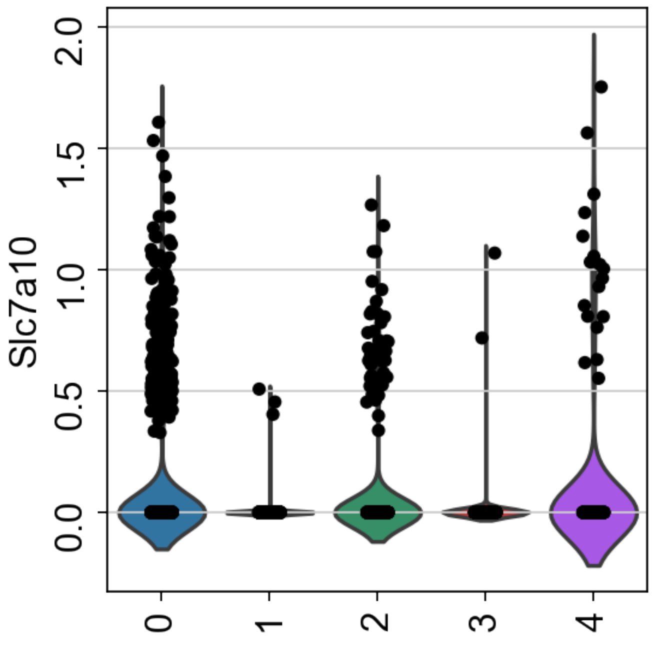

[34]:
```
if bool_plot==True:
    plot_tsne_marker(adata_adip, adipocyte_markers, size=20,␣
 ↪save="_preadip_markers_preadipcytes", use_raw=False)
```

WARNING: saving figure to file figures/tsne_preadip_markers_preadipcytes_0.pdf

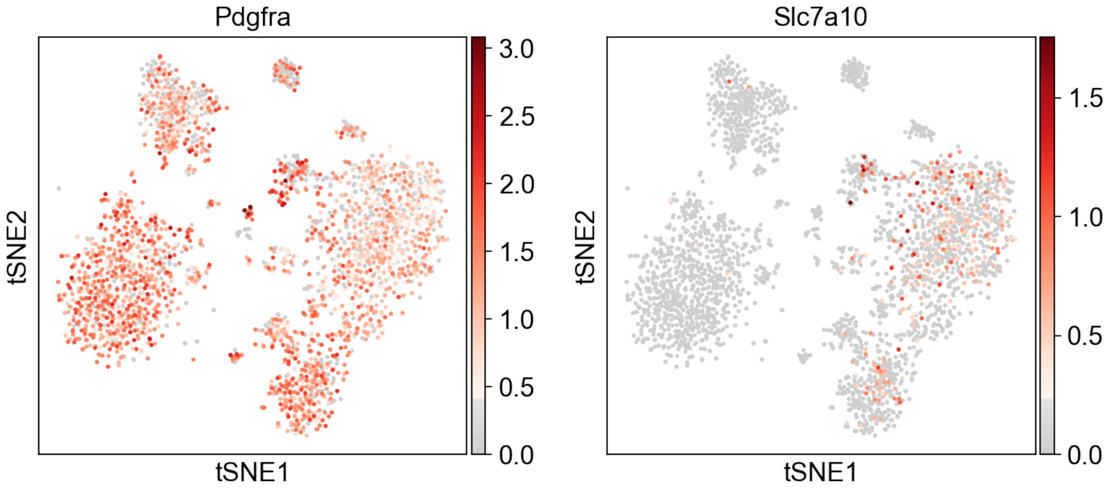

WARNING: saving figure to file figures/tsne_preadip_markers_preadipcytes_1.pdf

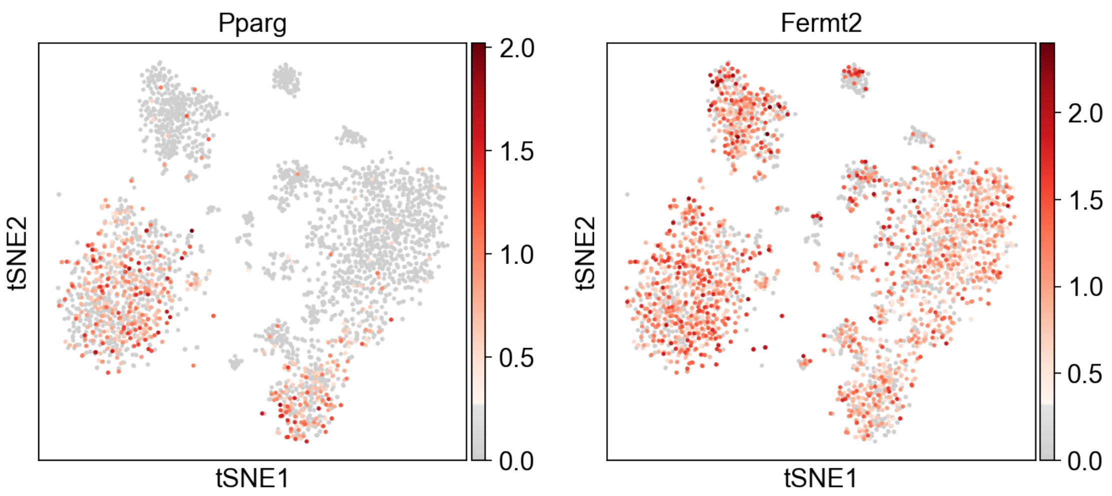

WARNING: saving figure to file figures/tsne_preadip_markers_preadipcytes_2.pdf

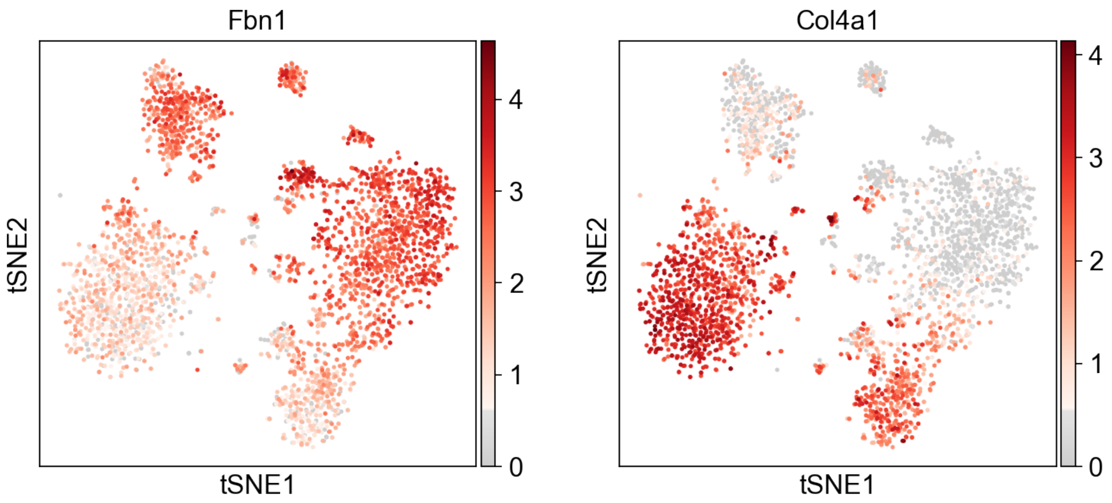

WARNING: saving figure to file figures/tsne_preadip_markers_preadipcytes_3.pdf

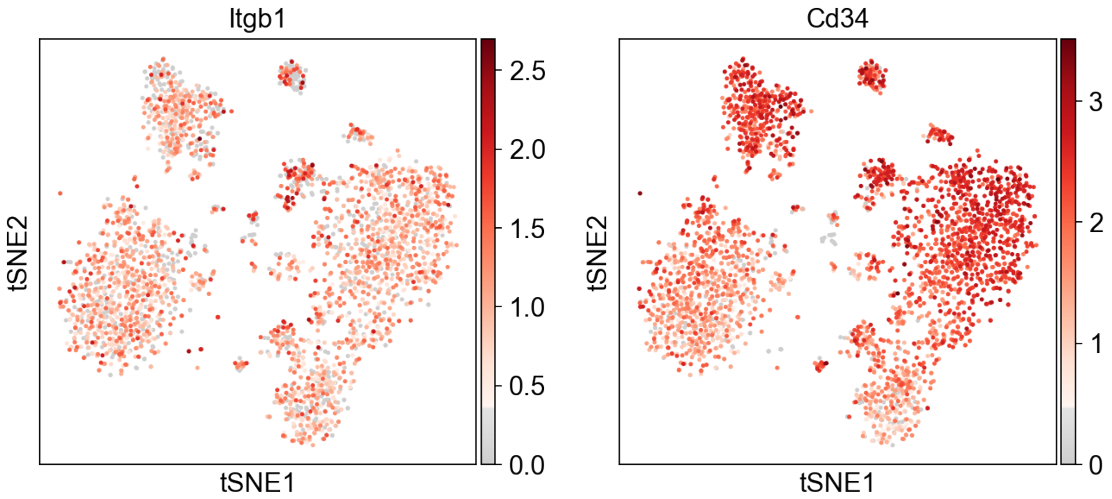


```
WARNING: saving figure to file figures/tsne_preadip_markers_preadipcytes_4.pdf
```

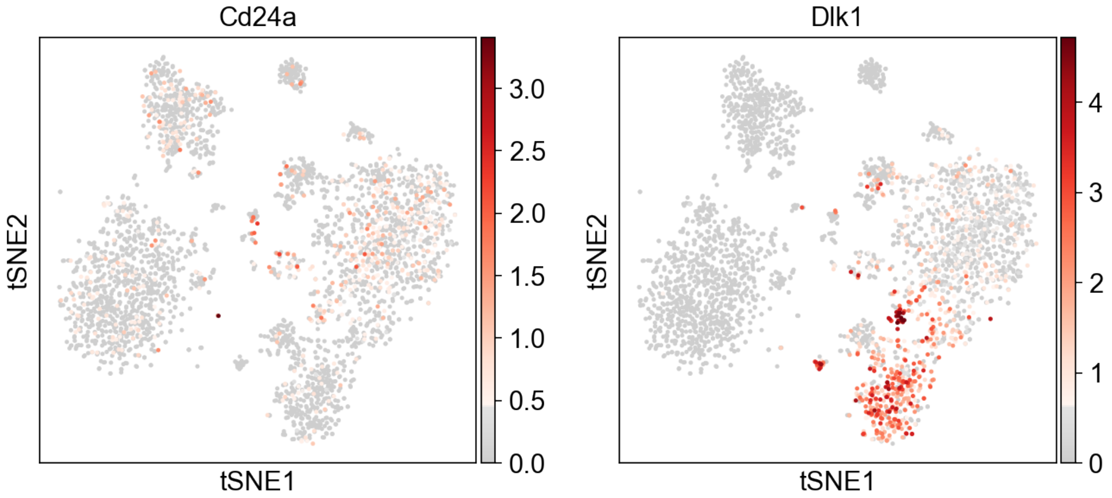


```
WARNING: saving figure to file figures/tsne_preadip_markers_preadipcytes_5.pdf
```

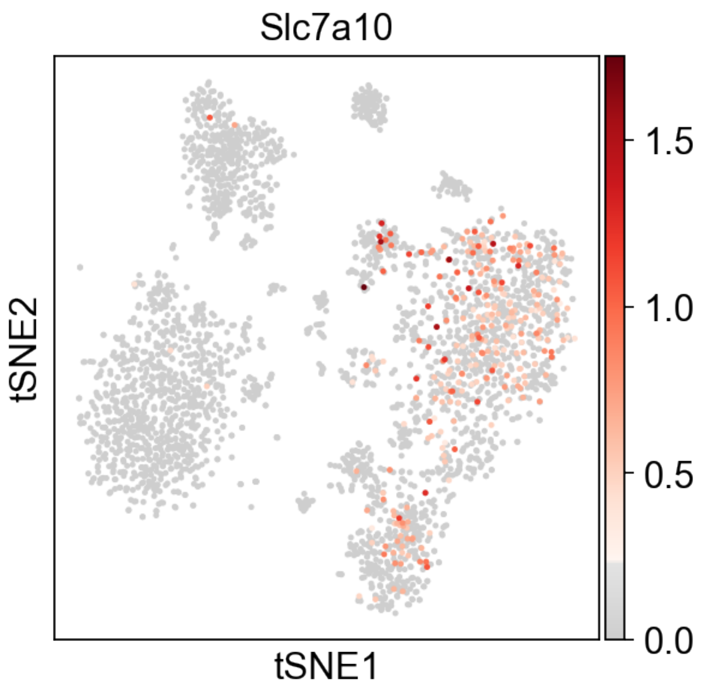

It seems that Slc7a10+ cells are in clusters 0,2,4, which directly correspond to the samples of the young mice with a few exceptions.

7 Preadipocytes only by age

7.1 Embedding and clustering

7.1.1 Young mouse

[35]:
```
if bool_recomp==True:
    cell_ids_adip_young = np.asarray(adata_proc.obs_names)[
        np.logical_and(np.asarray([x in ['2','3','4']
                                   for x in np.asarray(adata_proc.
↪obs['louvain'].values)]),
                       np.asarray([x=='young'
                                   for x in np.asarray(adata_proc.obs['age'].
↪values)]))]
    adata_adip_young = adata_raw[cell_ids_adip_young,:].copy()
    sc.pp.filter_cells(adata_adip_young, min_counts=500)
    sc.pp.normalize_per_cell(adata_adip_young)
    adata_adip_young.raw = adata_adip_young.copy()
    sc.pp.log1p(adata_adip_young)
    sc.pp.pca(adata_adip_young, n_comps=50, random_state=0, svd_solver='arpack')
    sc.pp.neighbors(adata_adip_young, n_neighbors=100, knn=True, method='umap',␣
↪n_pcs=50, random_state=0)
```

```
    sc.tl.tsne(adata_adip_young, n_jobs=3)
    if bool_recluster==True:
        sc.tl.louvain(adata_adip_young, resolution=1, flavor='vtraag',␣
↪random_state=0)
        pandas.DataFrame(adata_adip_young.obs).to_csv(
            path_or_buf =sc_settings_writedir+"obs_adata_adip_young.csv")
    else:
        obs = pandas.read_csv(sc_settings_writedir+'obs_adata_adip_young.csv')
        adata_adip_young.obs['louvain'] = pandas.Series(obs['louvain'].values,␣
↪dtype='category')
    sc.write(sc_settings_writedir+'adata_adip_young.h5ad', adata_adip_young)
else:
    adata_adip_young = sc.read(sc_settings_writedir+'adata_adip_young.h5ad')
sc.tl.paga(adata_adip_young)
```

```
running PAGA
    finished: added
    'paga/connectivities', connectivities adjacency (adata.uns)
    'paga/connectivities_tree', connectivities subtree (adata.uns) (0:00:00)
```

[36]:
```
if bool_plot==True:
    sc.pl.tsne(adata_adip_young, color=['louvain'], size=20,␣
↪save="_preadipYoung_louvain.pdf")
    sc.pl.tsne(adata_adip_young, color=['n_counts'], size=20,␣
↪save="_preadipYoung_n_counts.pdf")
    sc.pl.paga(adata_adip_young, save="_preadipYoung.pdf")
```

```
WARNING: saving figure to file figures/tsne_preadipYoung_louvain.pdf
```

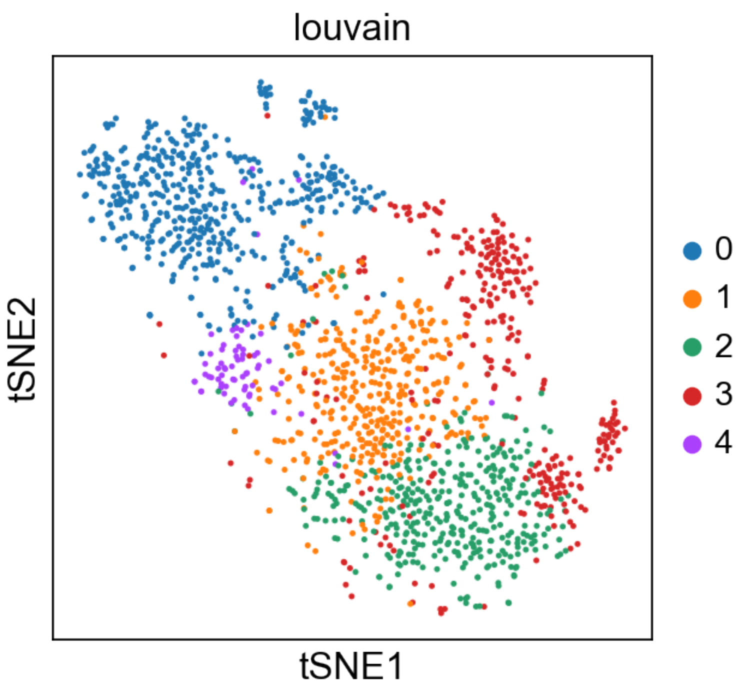

WARNING: saving figure to file figures/tsne_preadipYoung_n_counts.pdf

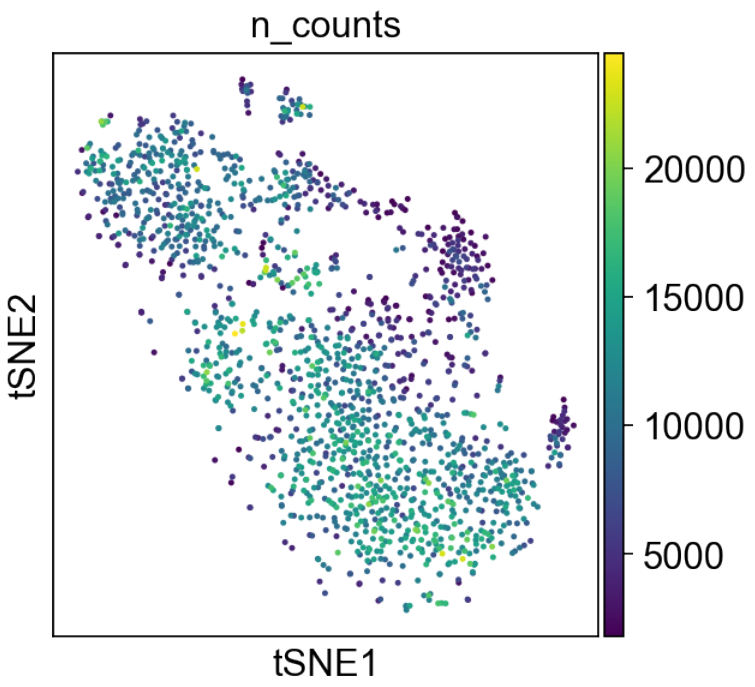

```
--> added 'pos', the PAGA positions (adata.uns['paga'])
WARNING: saving figure to file figures/paga_preadipYoung.pdf
```

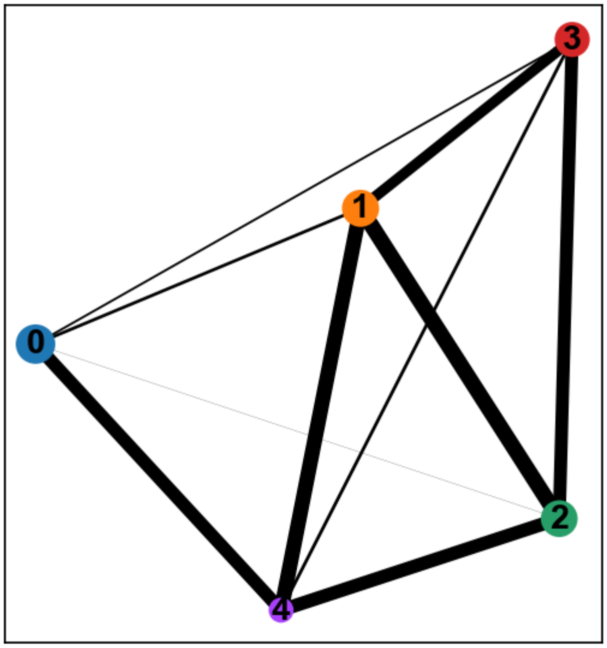

Number of preadipocytes observed in young mouse with scRNAseq:

[37]:
```
print(adata_adip_young.X.shape[0])
```

```
1636
```

7.1.2 Young mouse - coarse clustering

[38]:
```
adata_adip_young_lowres = adata_adip_young.copy()
if True:
    if True:
        sc.tl.louvain(adata_adip_young_lowres, resolution=0.5, flavor='vtraag',␣
 ↪random_state=0)
        pandas.DataFrame(adata_adip_young_lowres.obs).to_csv(
            path_or_buf =sc_settings_writedir+"obs_adata_adip_young_lowres.csv")
    else:
        obs = pandas.read_csv(sc_settings_writedir+'obs_adata_adip_young_lowres.
 ↪csv')
        adata_adip_young_lowres.obs['louvain'] = pandas.Series(obs['louvain'].
 ↪values, dtype='category')
```

```
running Louvain clustering
    using the "louvain" package of Traag (2017)
```

```
finished: found 2 clusters and added
'louvain', the cluster labels (adata.obs, categorical) (0:00:00)
```

[39]:
```
if bool_plot==True:
    sc.pl.tsne(adata_adip_young_lowres, color=['louvain'], size=20,␣
↪save="_preadipYoung_louvain_lowres.pdf")
```

```
WARNING: saving figure to file figures/tsne_preadipYoung_louvain_lowres.pdf
```

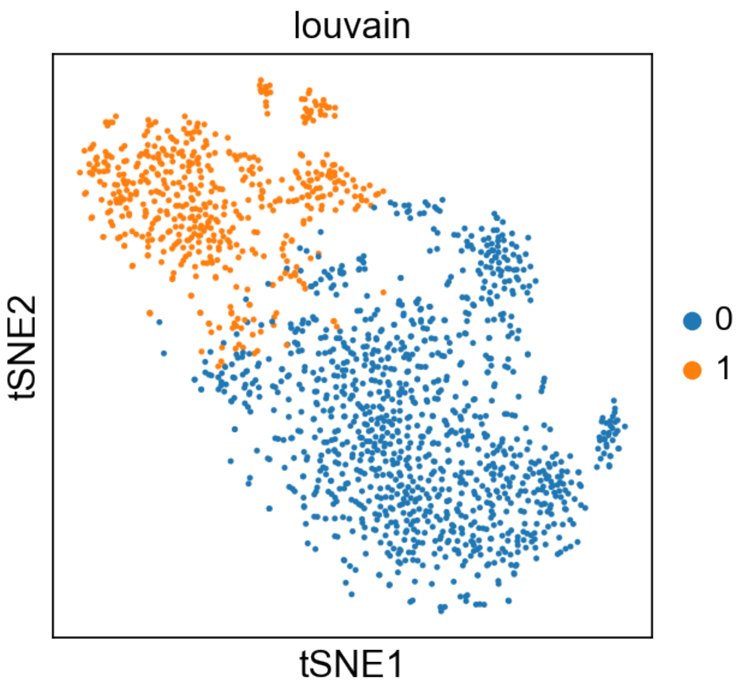

[40]:
```
adata_adip_young_lowres.obs['louvain'].value_counts()
```

[40]:
```
0    1115
1     521
Name: louvain, dtype: int64
```

7.1.3 Old mouse

[41]:
```
if bool_recomp==True:
    cell_ids_adip_old = np.asarray(adata_proc.obs_names)[
        np.logical_and(np.asarray([x in ['2','3','4']
                                   for x in np.asarray(adata_proc.
↪obs['louvain'].values)]),
                       np.asarray([x=='old'
                                   for x in np.asarray(adata_proc.obs['age'].
↪values)]))]
```

```
    adata_adip_old = adata_raw[cell_ids_adip_old,:].copy()
    sc.pp.filter_cells(adata_adip_old, min_counts=500)
    sc.pp.normalize_per_cell(adata_adip_old)
    adata_adip_old.raw = adata_adip_old.copy()
    sc.pp.log1p(adata_adip_old)
    sc.pp.pca(adata_adip_old, n_comps=50, random_state=0, svd_solver='arpack')
    sc.pp.neighbors(adata_adip_old, n_neighbors=50, knn=True, method='umap',␣
↪n_pcs=50, random_state=0)
    sc.tl.tsne(adata_adip_old, n_jobs=3)
    if bool_recluster==True:
        sc.tl.louvain(adata_adip_old, resolution=1, flavor='vtraag',␣
↪random_state=0)
        pandas.DataFrame(adata_adip_old.obs).to_csv(
            path_or_buf =sc_settings_writedir+"obs_adata_adip_old.csv")
    else:
        obs = pandas.read_csv(sc_settings_writedir+'obs_adata_adip_old.csv')
        adata_adip_old.obs['louvain'] = pandas.Series(obs['louvain'].values,␣
↪dtype='category')
    sc.write(sc_settings_writedir+'adata_adip_old.h5ad', adata_adip_old)
else:
    adata_adip_old = sc.read(sc_settings_writedir+'adata_adip_old.h5ad')
sc.tl.paga(adata_adip_old)
```

```
running PAGA
    finished: added
    'paga/connectivities', connectivities adjacency (adata.uns)
    'paga/connectivities_tree', connectivities subtree (adata.uns) (0:00:00)
```

[42]:
```
if bool_plot==True:
    sc.pl.tsne(adata_adip_old, color=['louvain'], size=20,␣
↪save="_preadipOld_louvain.pdf")
    sc.pl.tsne(adata_adip_old, color=['n_counts'], size=20,␣
↪save="_preadipOld_n_counts.pdf")
    sc.pl.paga(adata_adip_old, save="_preadipOld.pdf")
```

```
WARNING: saving figure to file figures/tsne_preadipOld_louvain.pdf
```

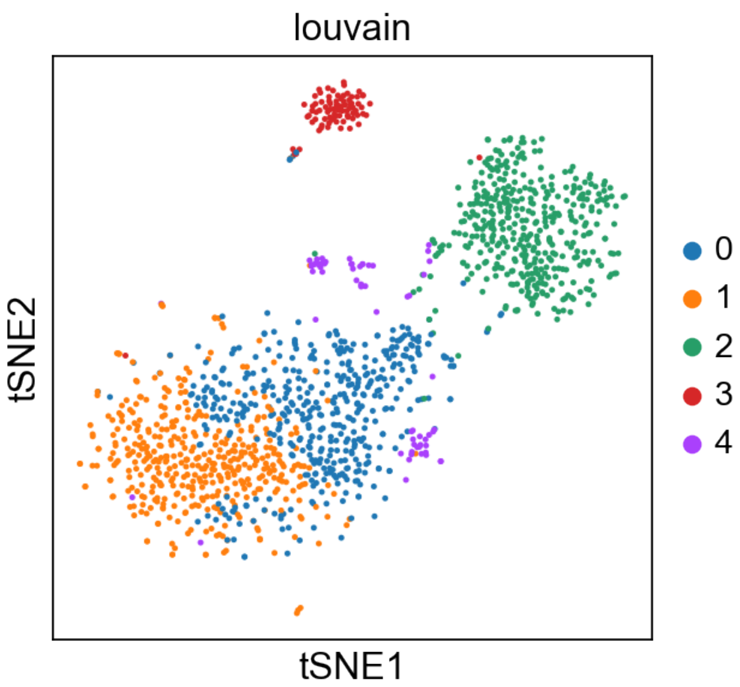

WARNING: saving figure to file figures/tsne_preadipOld_n_counts.pdf

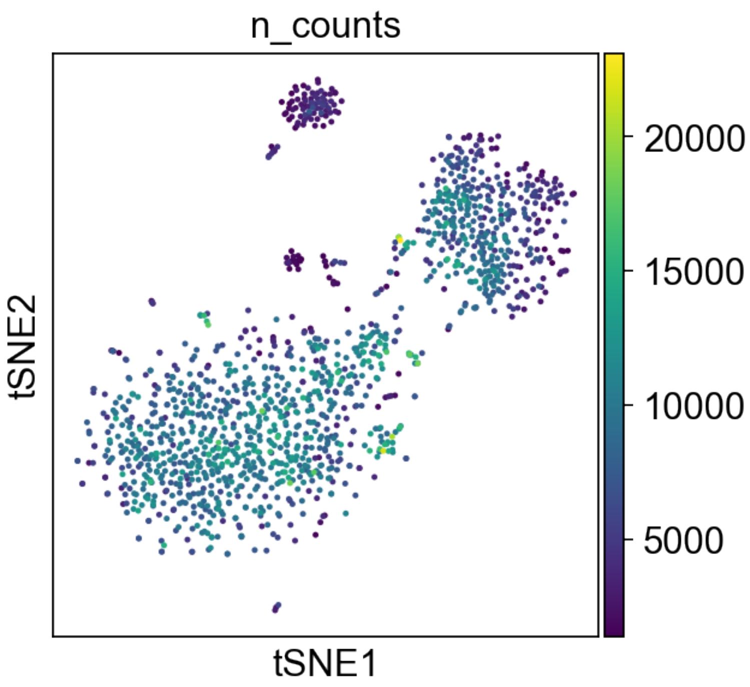

```
--> added 'pos', the PAGA positions (adata.uns['paga'])
WARNING: saving figure to file figures/paga_preadipOld.pdf
```

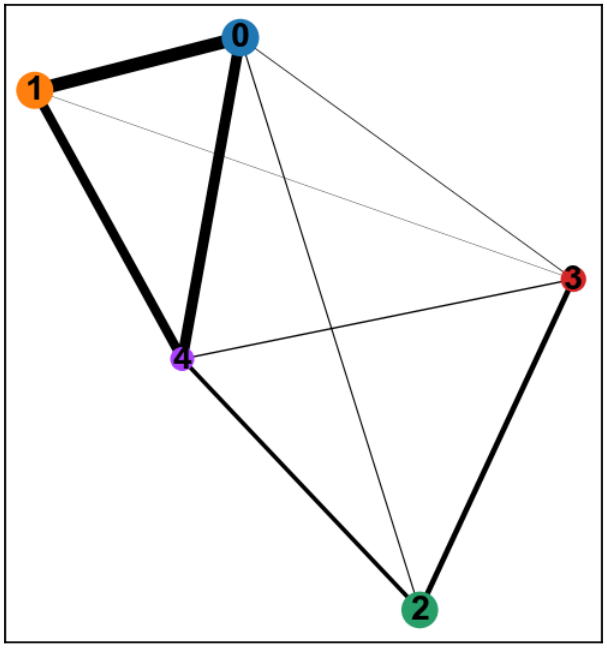

Number of preadipocytes observed in old mouse with scRNAseq:

[43]:
```python
print(adata_adip_old.X.shape[0])
```

```
1426
```

7.2 Marker gene sets

7.2.1 Young mouse

[44]:
```python
adipocyte_markers=['Pdgfra','Slc7a10','Pparg','Fermt2','Fbn1','Col4a1','Itgb1','Cd34','Cd24a',
```

[45]:
```python
if bool_plot==True:
    plot_violin_marker(adata_adip_young, adipocyte_markers,␣
↪save="_preadipYoung_markers_preadipocytes", use_raw=False)
```

```
WARNING: saving figure to file
figures/violin_preadipYoung_markers_preadipocytes_0.pdf
```

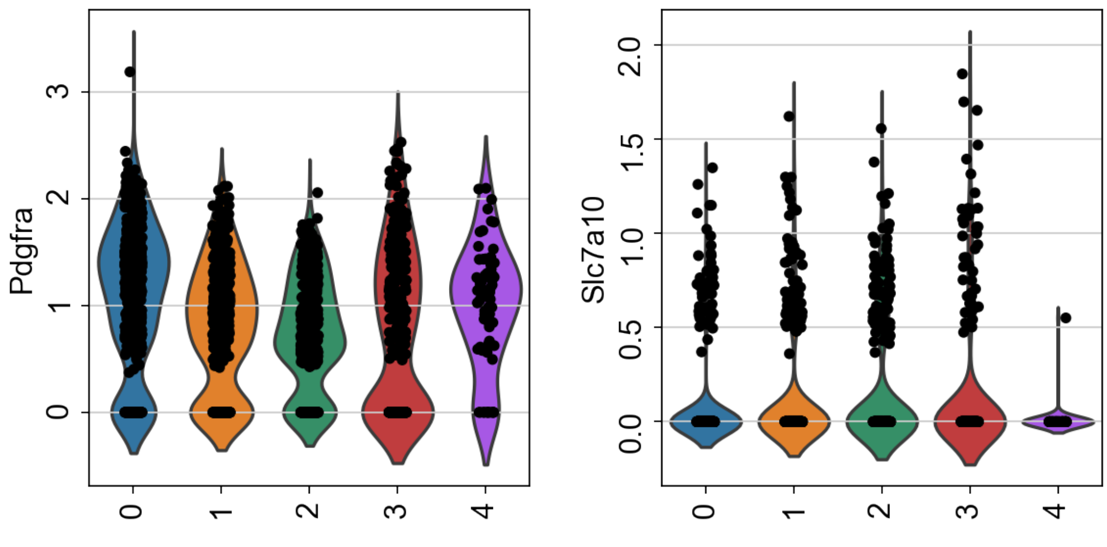

WARNING: saving figure to file
figures/violin_preadipYoung_markers_preadipocytes_1.pdf

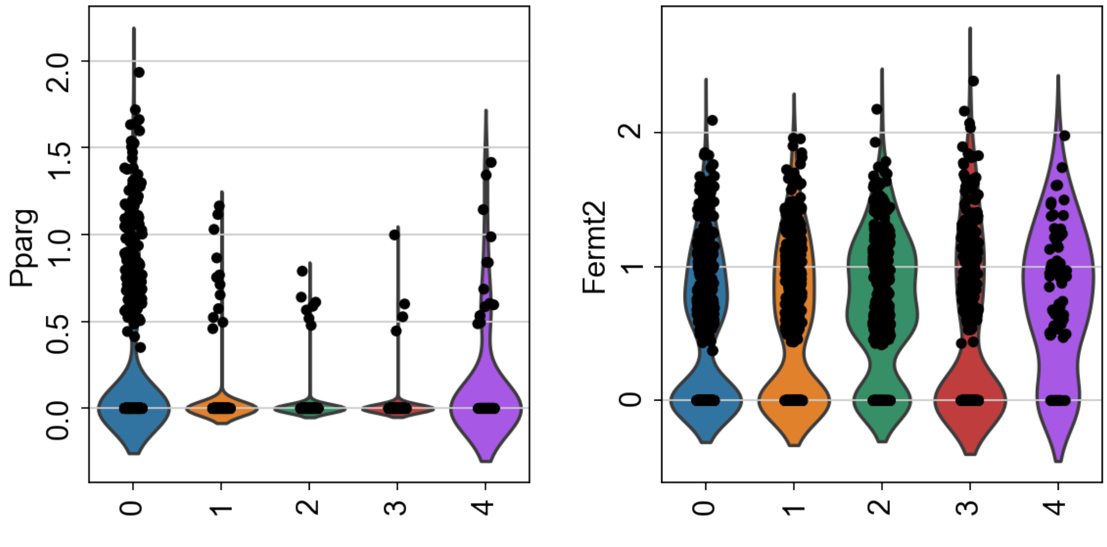

WARNING: saving figure to file
figures/violin_preadipYoung_markers_preadipocytes_2.pdf

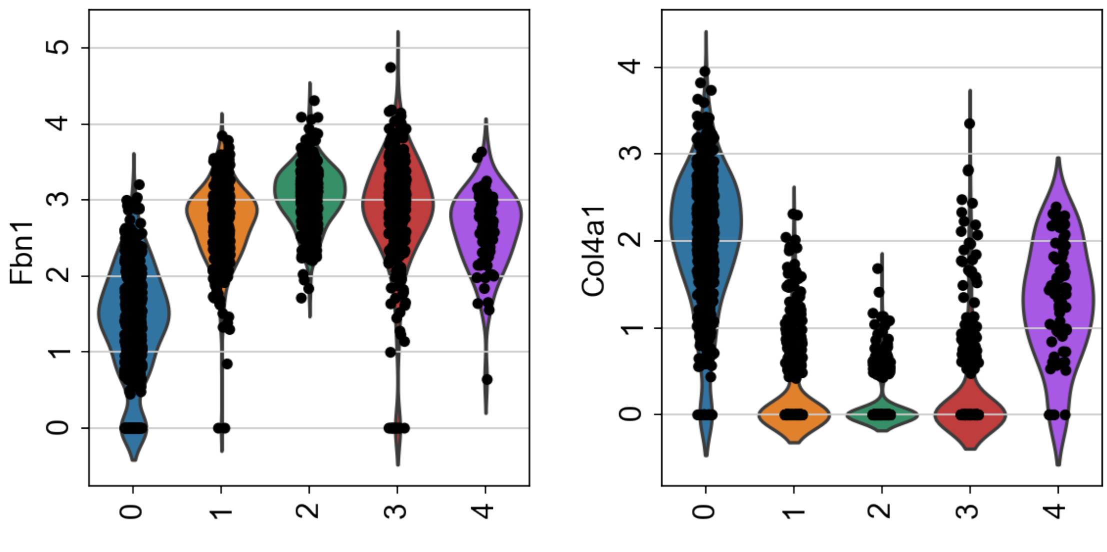

WARNING: saving figure to file
figures/violin_preadipYoung_markers_preadipocytes_3.pdf

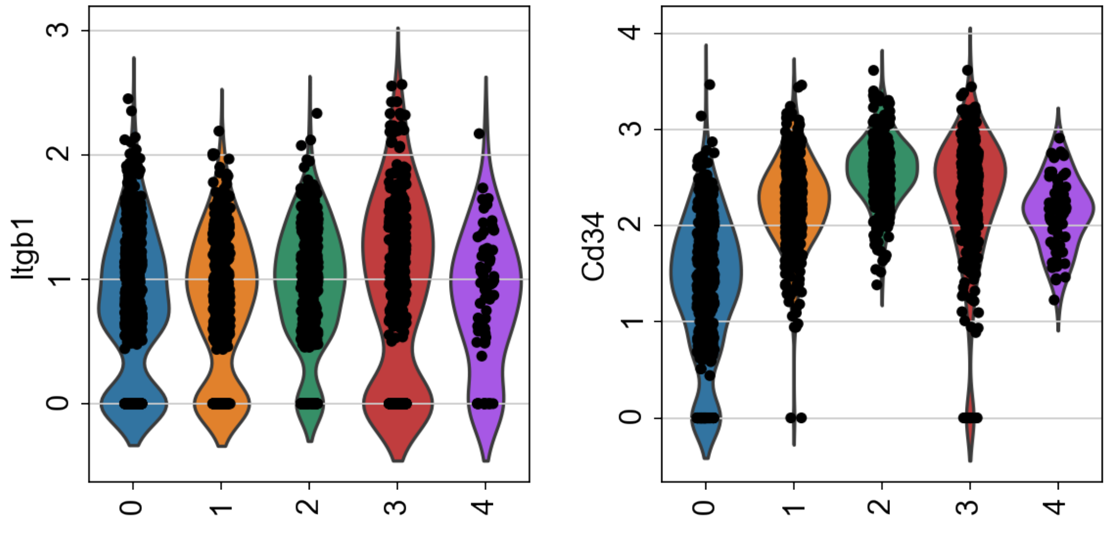

WARNING: saving figure to file
figures/violin_preadipYoung_markers_preadipocytes_4.pdf

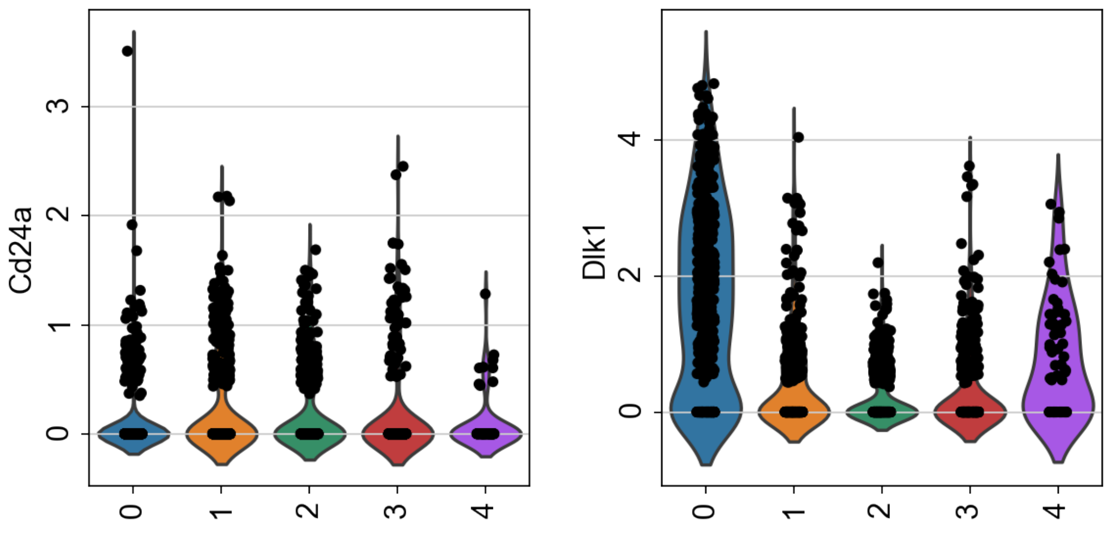

WARNING: saving figure to file
figures/violin_preadipYoung_markers_preadipocytes_5.pdf

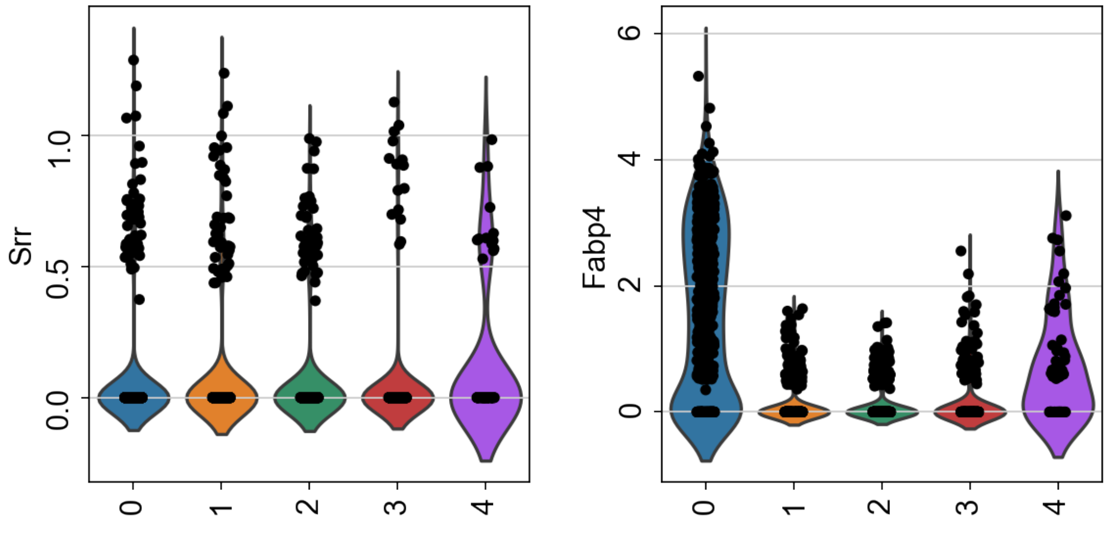

WARNING: saving figure to file
figures/violin_preadipYoung_markers_preadipocytes_6.pdf

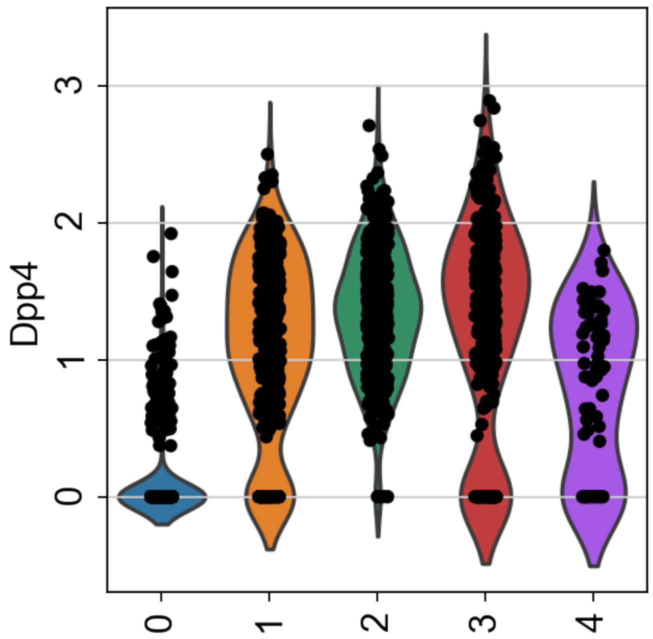

[46]:
```
if bool_plot==True:
    plot_violin_marker(adata_adip_young, go_adip_dev,␣
↪save="_preadipYoung_markers_GO_adipocyte_dev", use_raw=False)
```

```
WARNING: saving figure to file
figures/violin_preadipYoung_markers_GO_adipocyte_dev_0.pdf
```

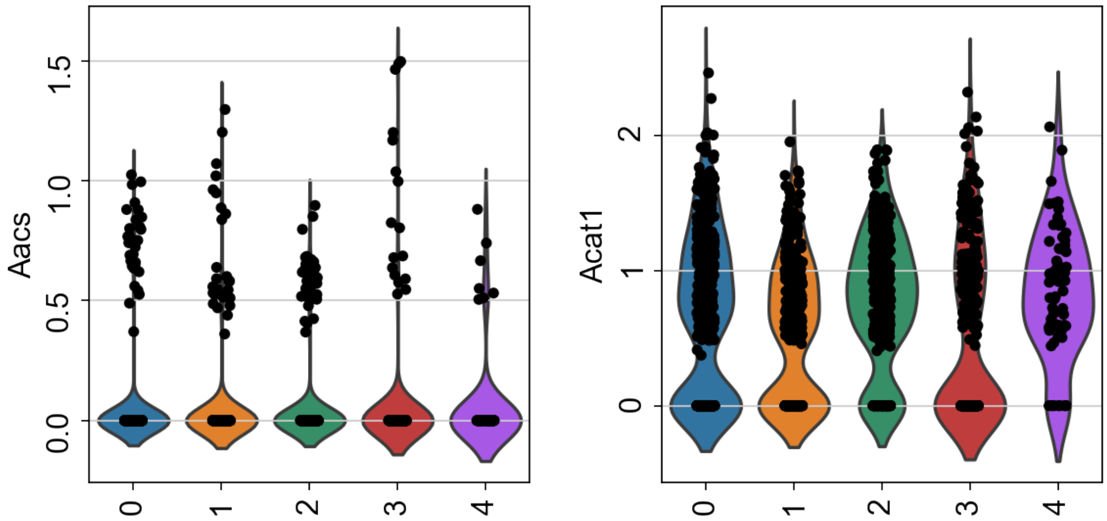


```
WARNING: saving figure to file
```

```
figures/violin_preadipYoung_markers_GO_adipocyte_dev_1.pdf
```

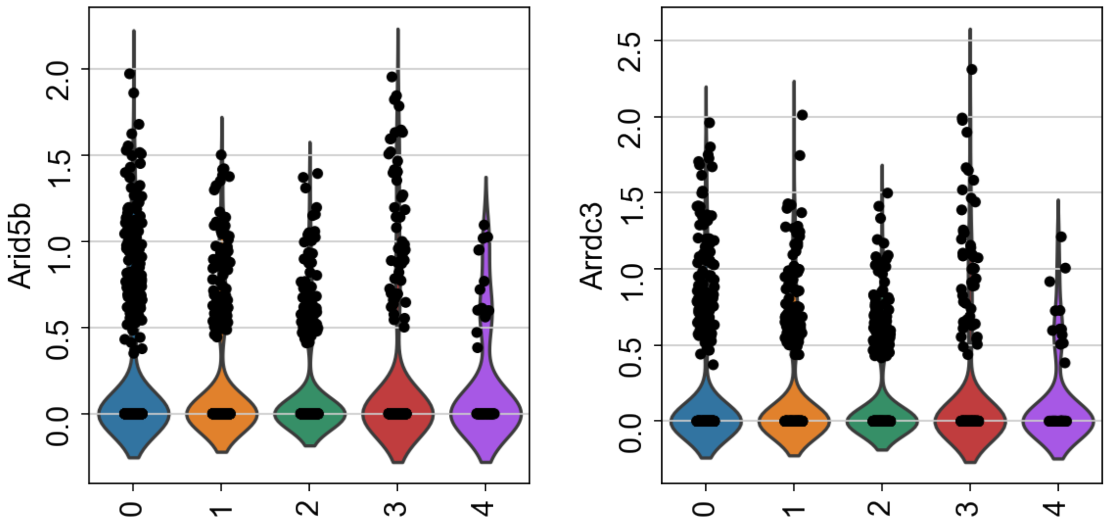


```
WARNING: saving figure to file
figures/violin_preadipYoung_markers_GO_adipocyte_dev_2.pdf
```

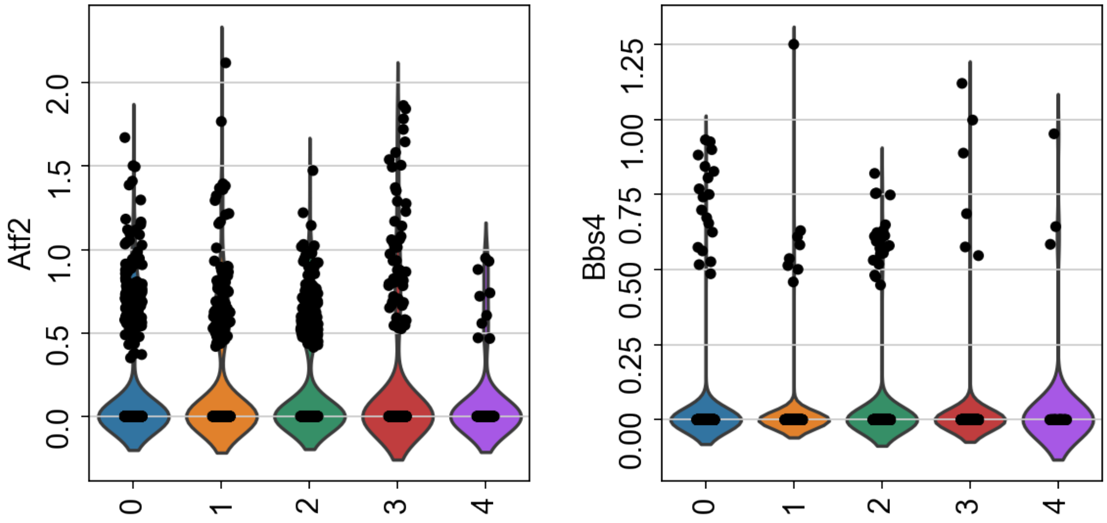


```
WARNING: saving figure to file
figures/violin_preadipYoung_markers_GO_adipocyte_dev_3.pdf
```

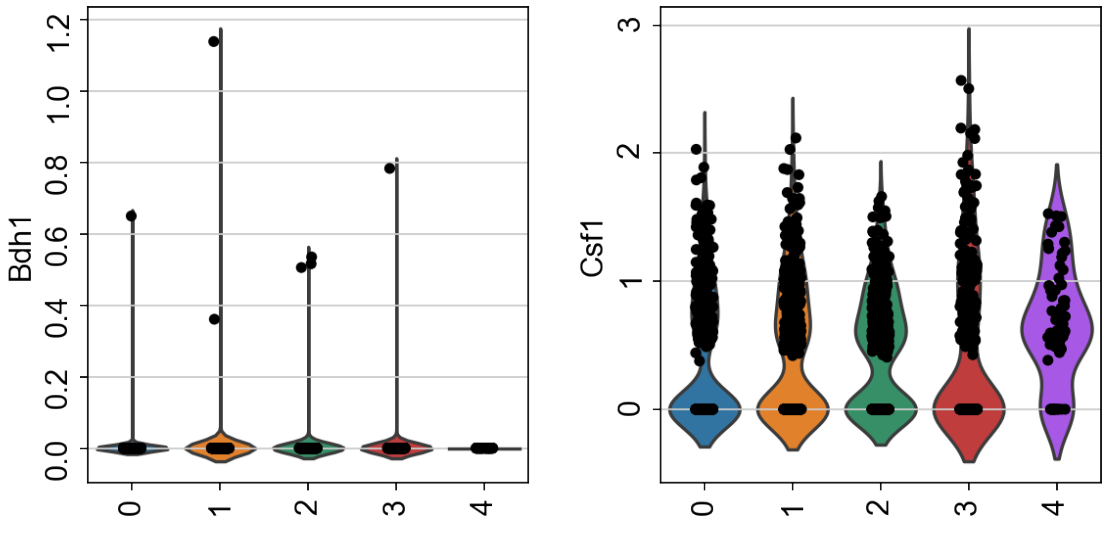

WARNING: saving figure to file
figures/violin_preadipYoung_markers_GO_adipocyte_dev_4.pdf

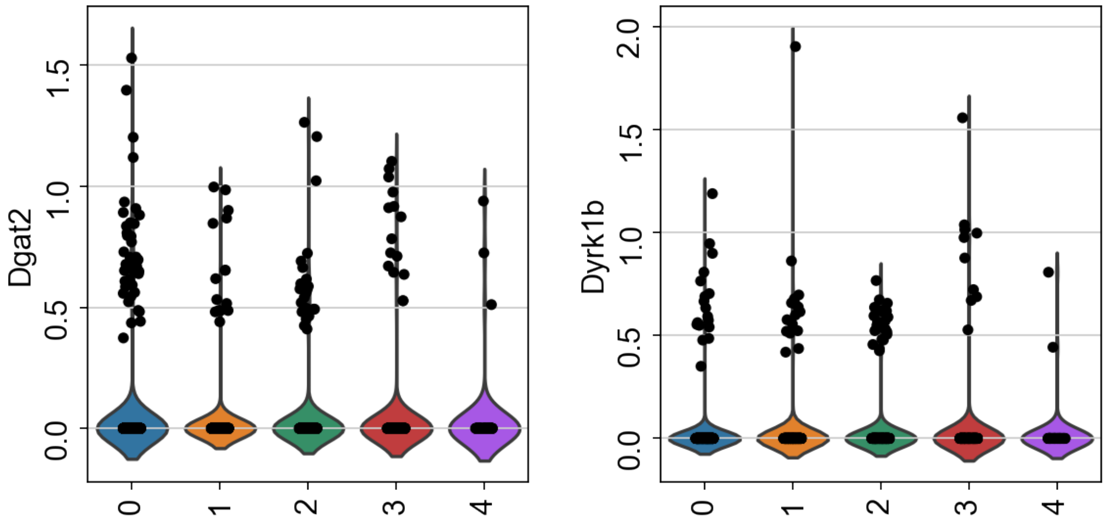

WARNING: saving figure to file
figures/violin_preadipYoung_markers_GO_adipocyte_dev_5.pdf

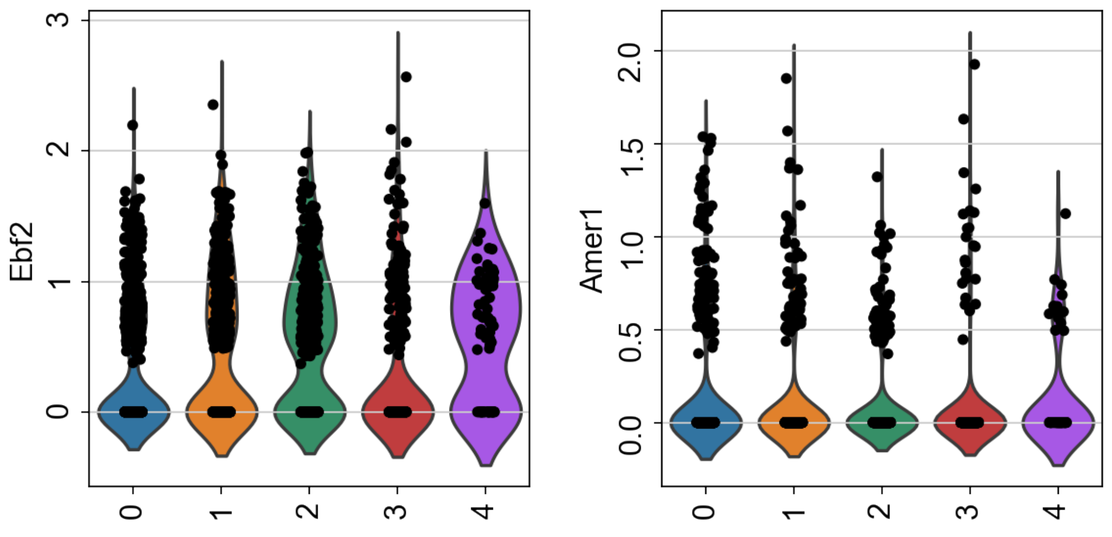

WARNING: saving figure to file
figures/violin_preadipYoung_markers_GO_adipocyte_dev_6.pdf

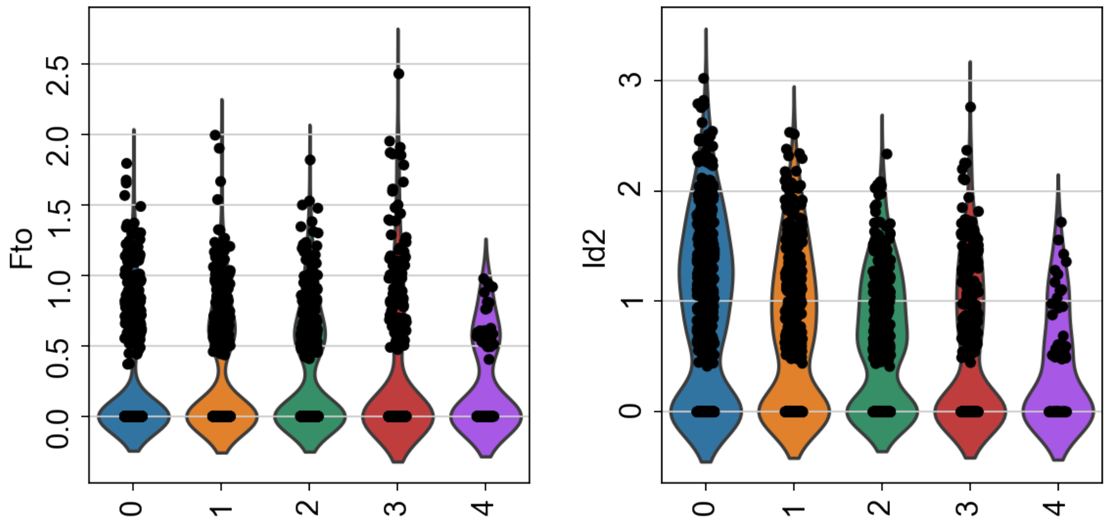

WARNING: saving figure to file
figures/violin_preadipYoung_markers_GO_adipocyte_dev_7.pdf

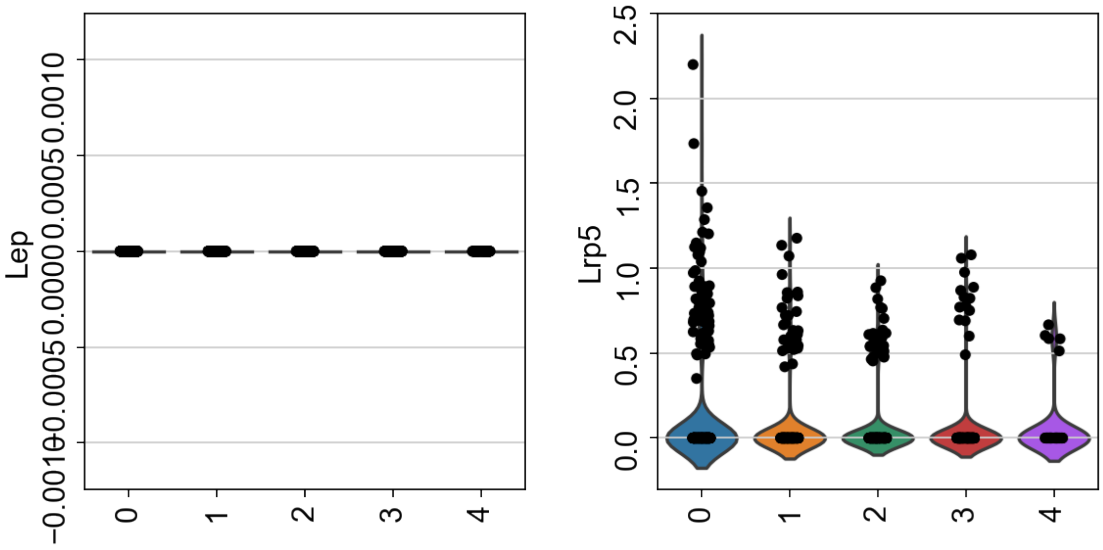

WARNING: saving figure to file
figures/violin_preadipYoung_markers_GO_adipocyte_dev_8.pdf

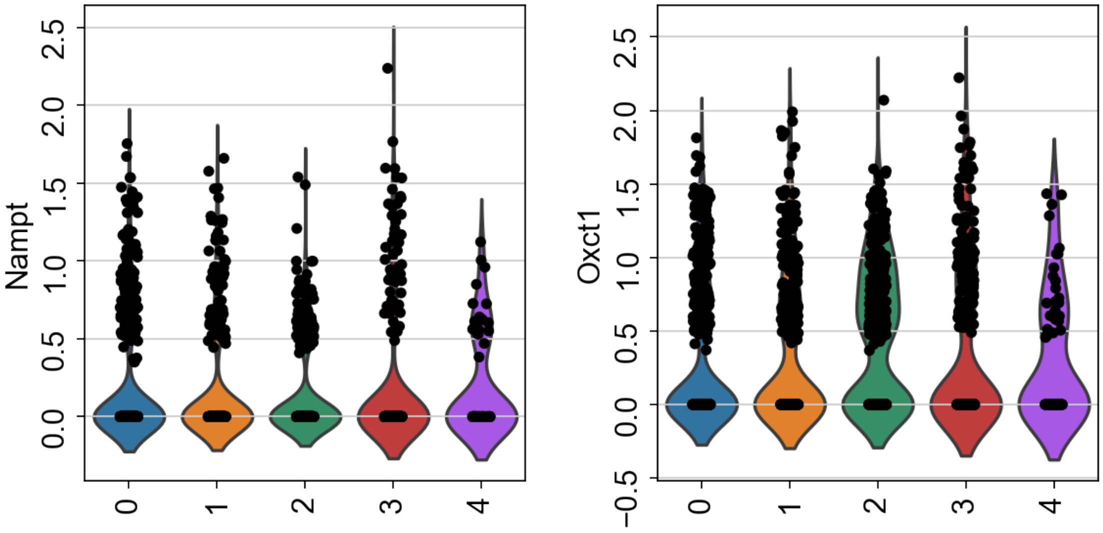

WARNING: saving figure to file
figures/violin_preadipYoung_markers_GO_adipocyte_dev_9.pdf

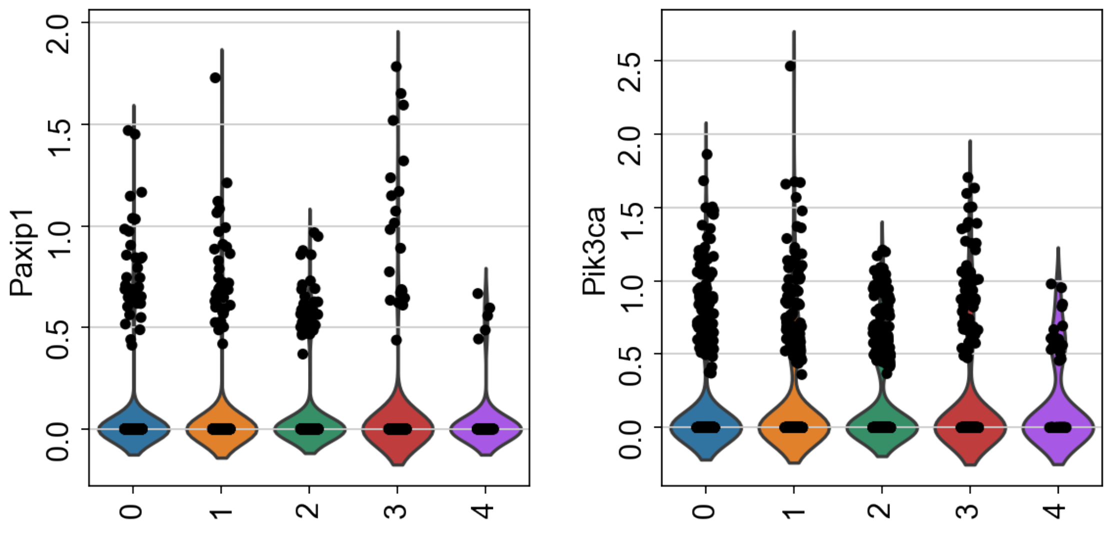

WARNING: saving figure to file
figures/violin_preadipYoung_markers_GO_adipocyte_dev_10.pdf

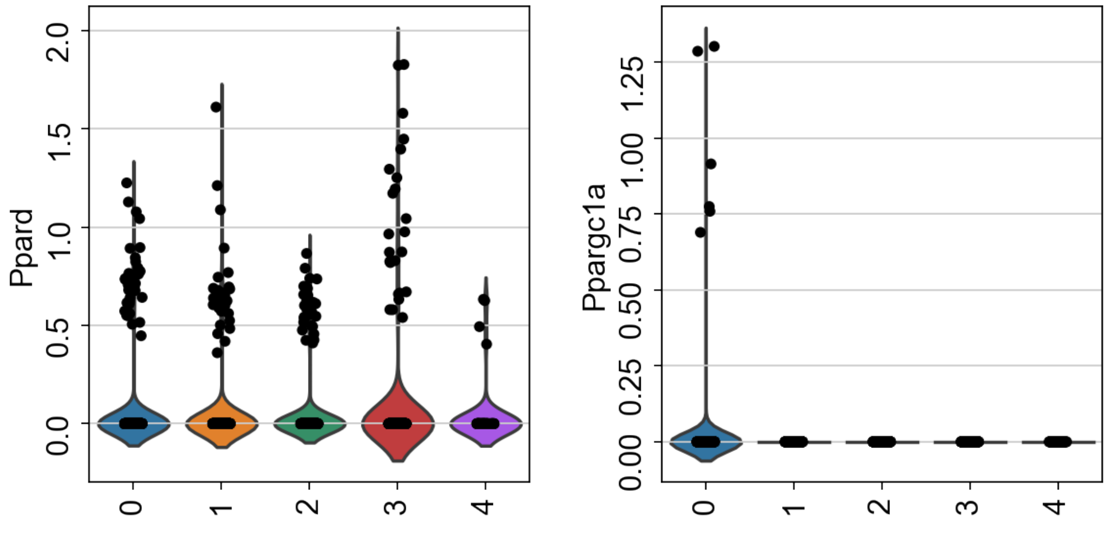

WARNING: saving figure to file
figures/violin_preadipYoung_markers_GO_adipocyte_dev_11.pdf

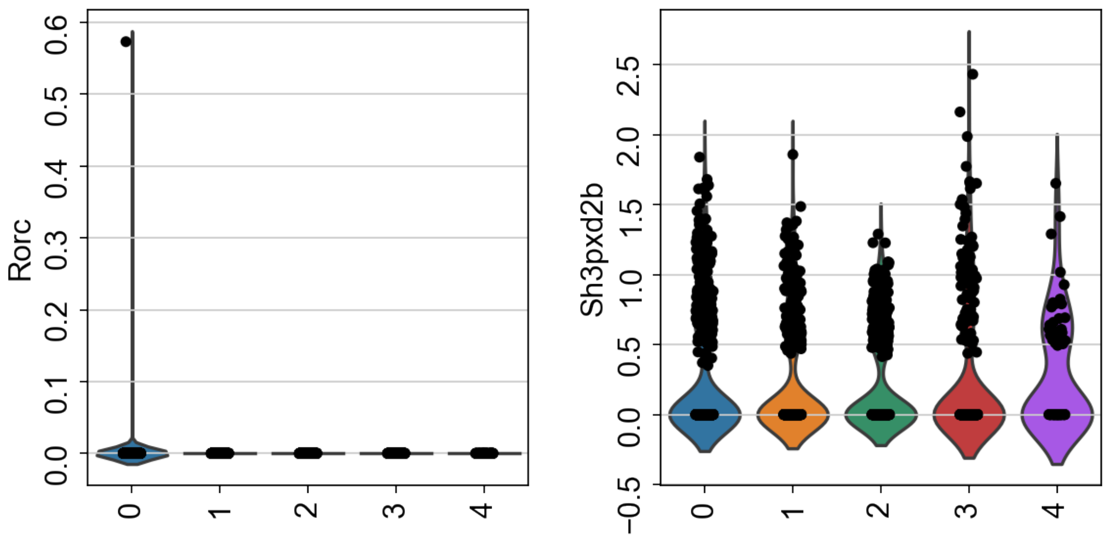


```
WARNING: saving figure to file
figures/violin_preadipYoung_markers_GO_adipocyte_dev_12.pdf
```

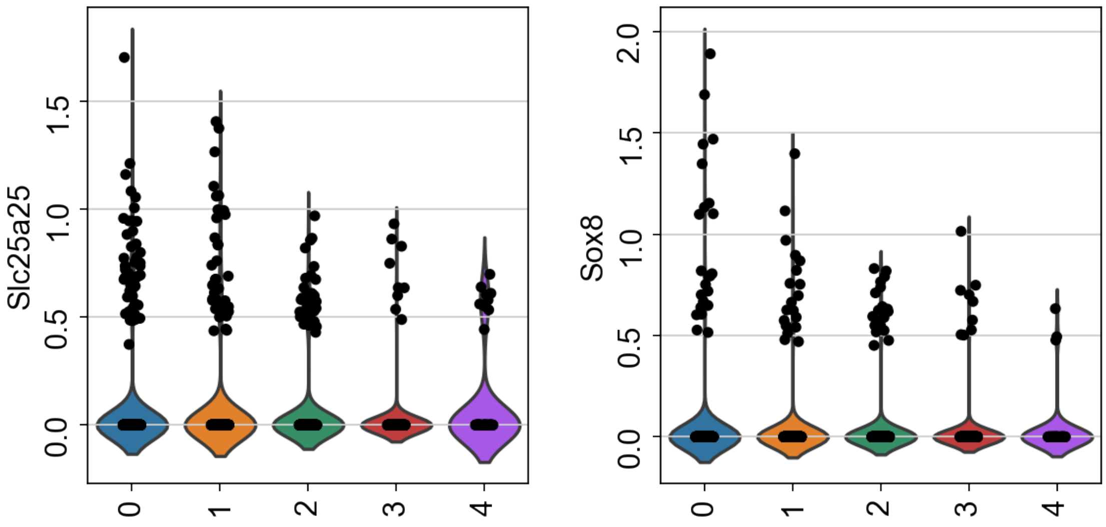


```
WARNING: saving figure to file
figures/violin_preadipYoung_markers_GO_adipocyte_dev_13.pdf
```

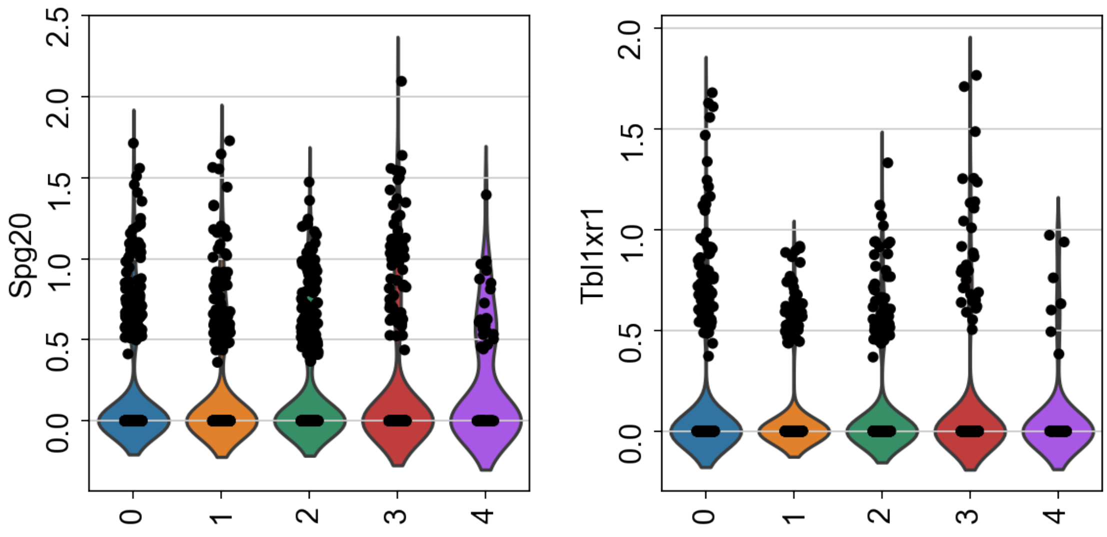


```
WARNING: saving figure to file
figures/violin_preadipYoung_markers_GO_adipocyte_dev_14.pdf
```

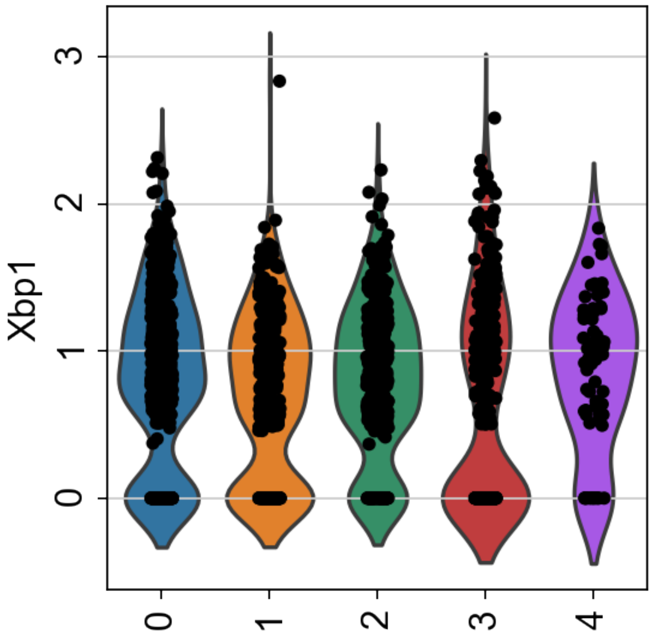

[47]:
```
if bool_plot==True:
    plot_tsne_marker(adata_adip_young, adipocyte_markers, size=20,␣
↪save="_preadipYoung_markers_preadipocytes", use_raw=False)
```

```
WARNING: saving figure to file
```

figures/tsne_preadipYoung_markers_preadipocytes_0.pdf

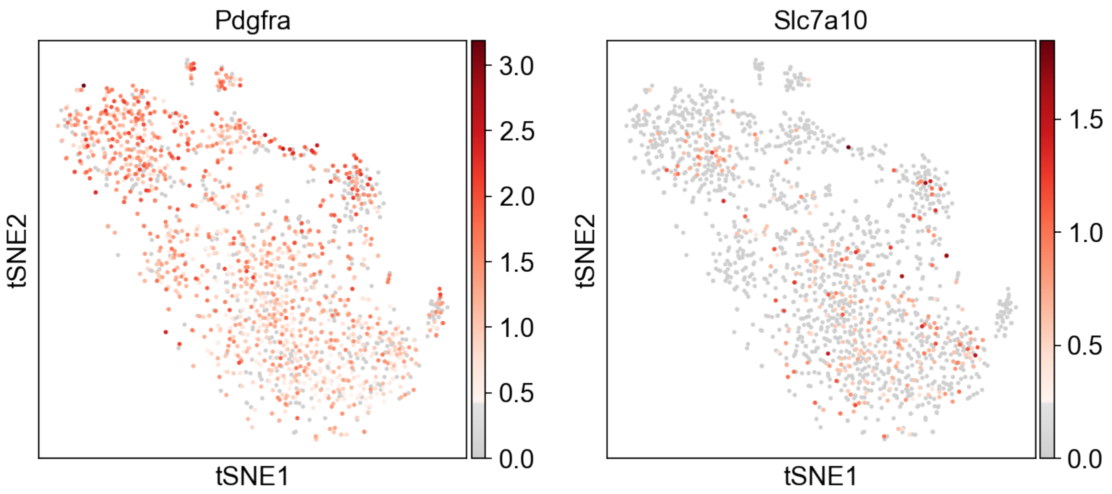

WARNING: saving figure to file
figures/tsne_preadipYoung_markers_preadipocytes_1.pdf

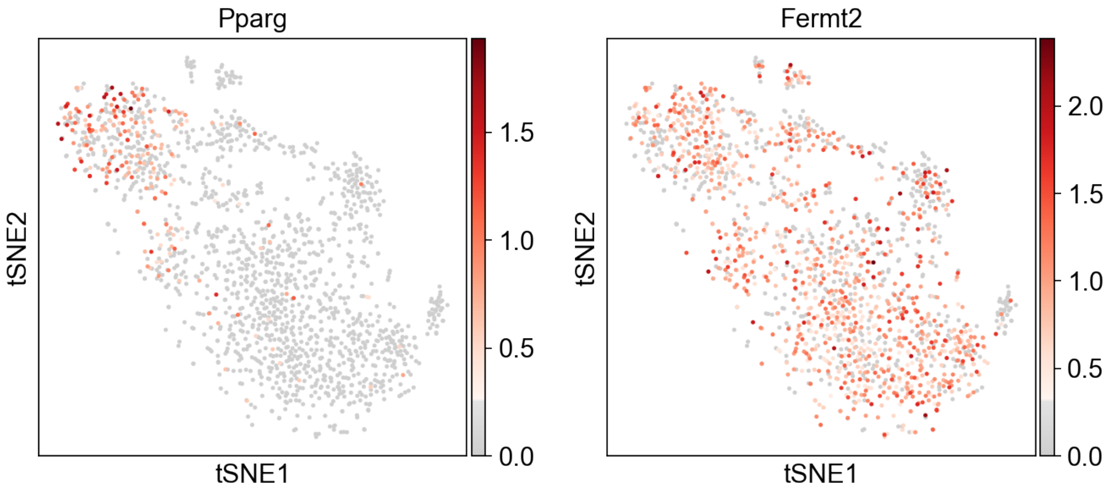

WARNING: saving figure to file
figures/tsne_preadipYoung_markers_preadipocytes_2.pdf

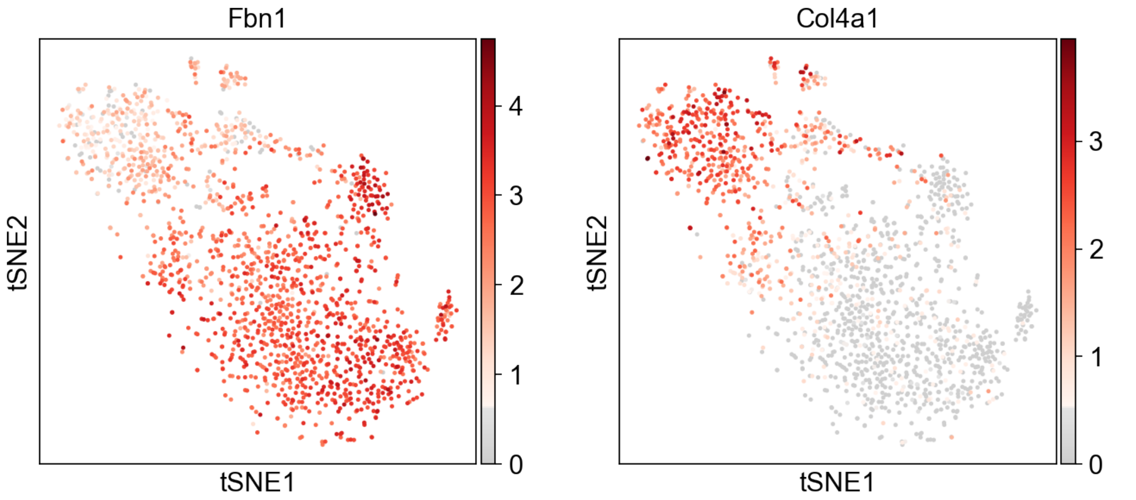


```
WARNING: saving figure to file
figures/tsne_preadipYoung_markers_preadipocytes_3.pdf
```

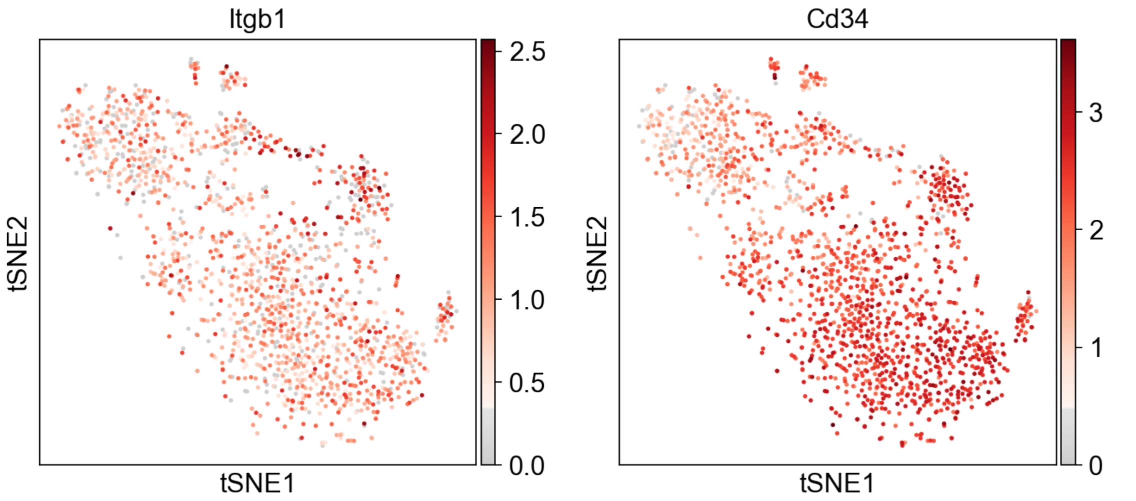


```
WARNING: saving figure to file
figures/tsne_preadipYoung_markers_preadipocytes_4.pdf
```

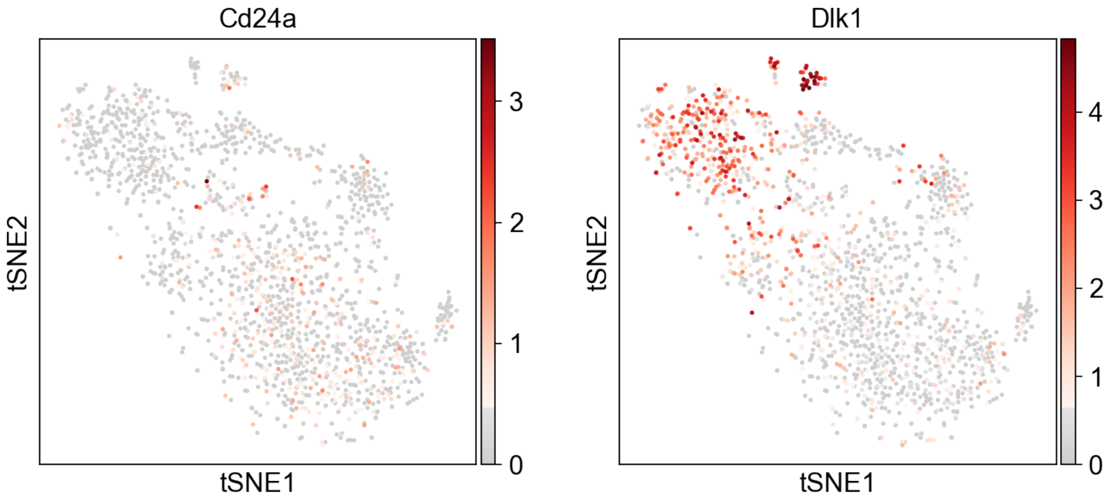

WARNING: saving figure to file
figures/tsne_preadipYoung_markers_preadipocytes_5.pdf

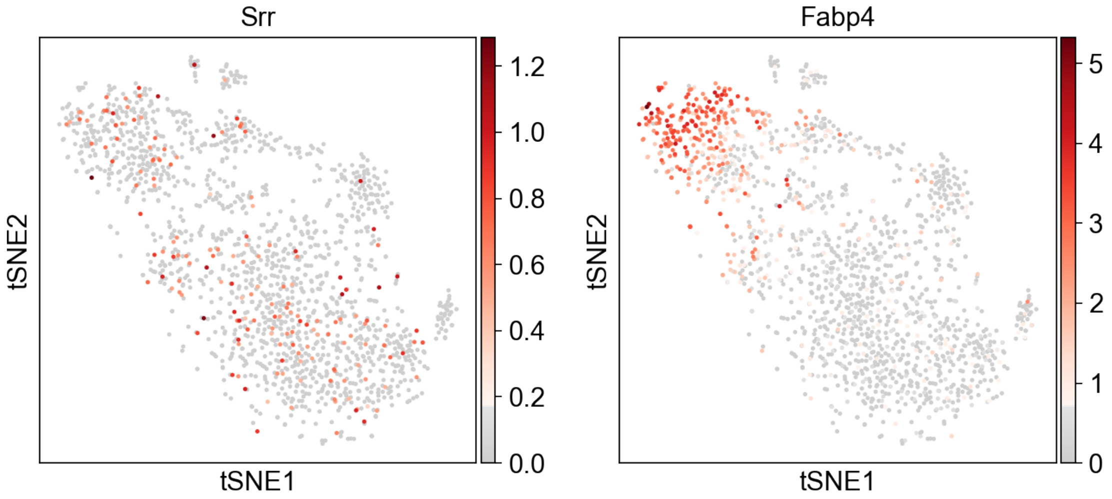

WARNING: saving figure to file
figures/tsne_preadipYoung_markers_preadipocytes_6.pdf

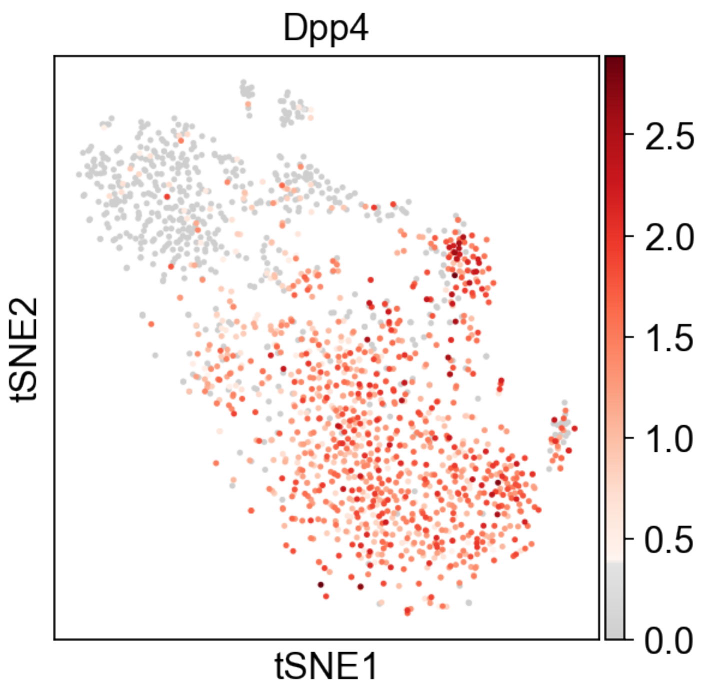

[48]:
```
if bool_plot==True:
    plot_tsne_marker(adata_adip_young, go_adip_dev, size=20,␣
 ↪save="_preadipYoung_markers_GO_adipocyte_dev", use_raw=False)
```

```
WARNING: saving figure to file
figures/tsne_preadipYoung_markers_GO_adipocyte_dev_0.pdf
```

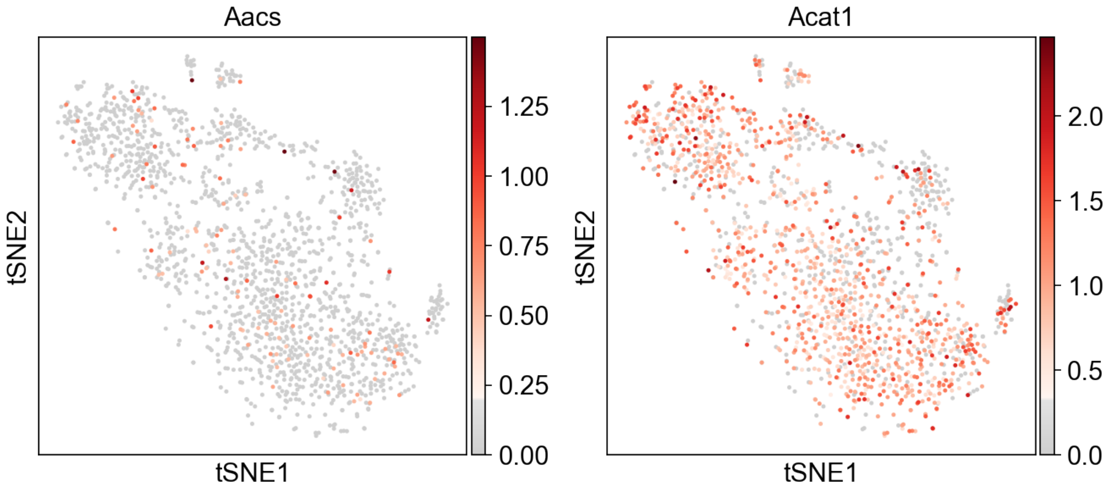


```
WARNING: saving figure to file
```

figures/tsne_preadipYoung_markers_GO_adipocyte_dev_1.pdf

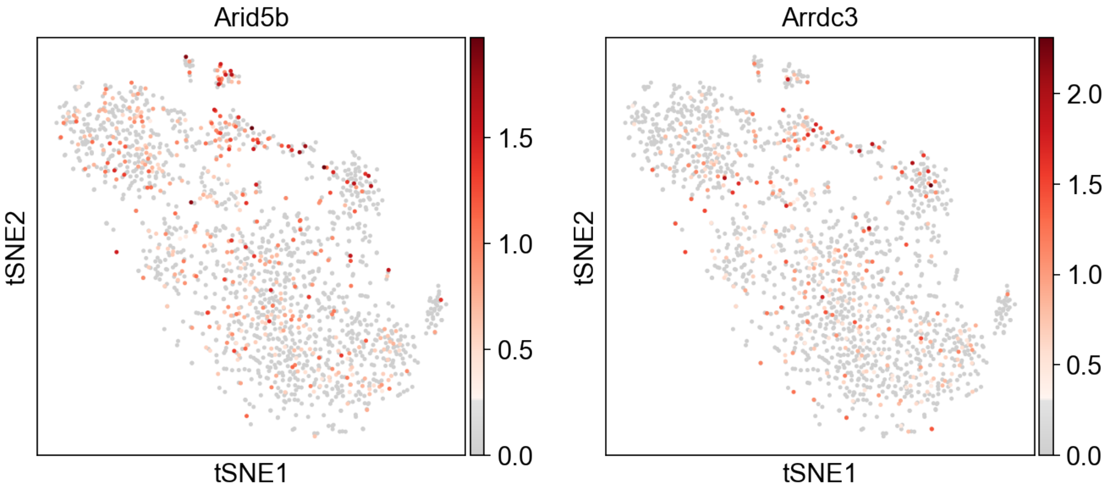

WARNING: saving figure to file
figures/tsne_preadipYoung_markers_GO_adipocyte_dev_2.pdf

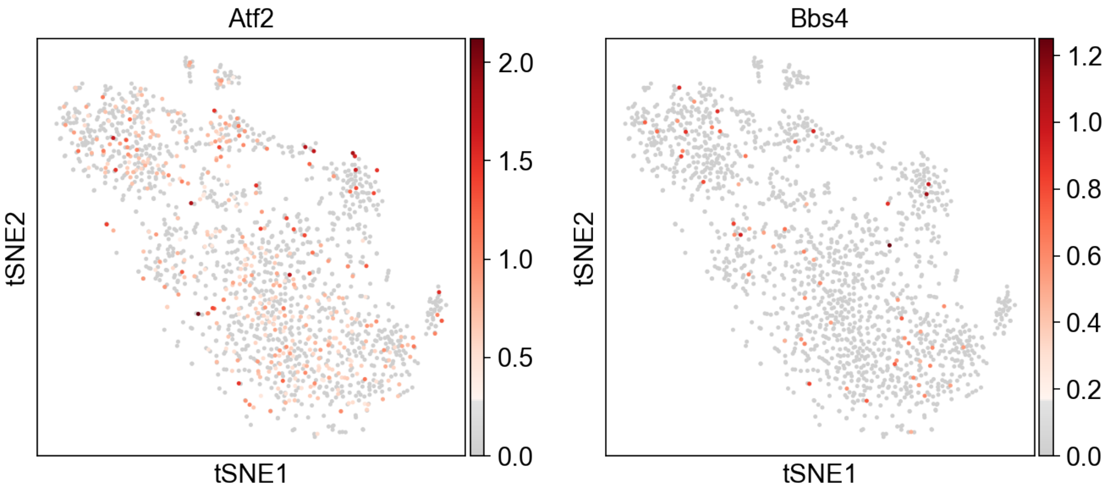

WARNING: saving figure to file
figures/tsne_preadipYoung_markers_GO_adipocyte_dev_3.pdf

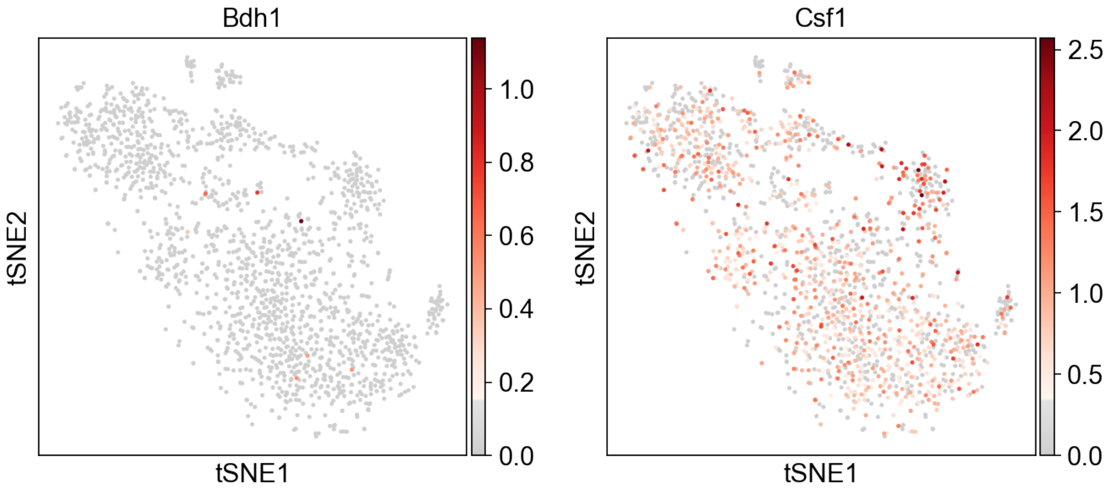

WARNING: saving figure to file
figures/tsne_preadipYoung_markers_GO_adipocyte_dev_4.pdf

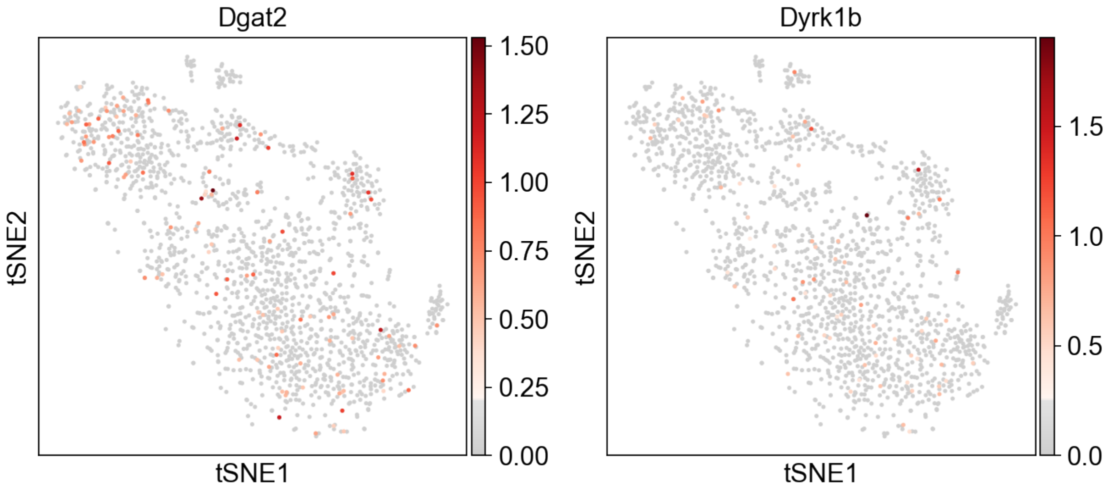

WARNING: saving figure to file
figures/tsne_preadipYoung_markers_GO_adipocyte_dev_5.pdf

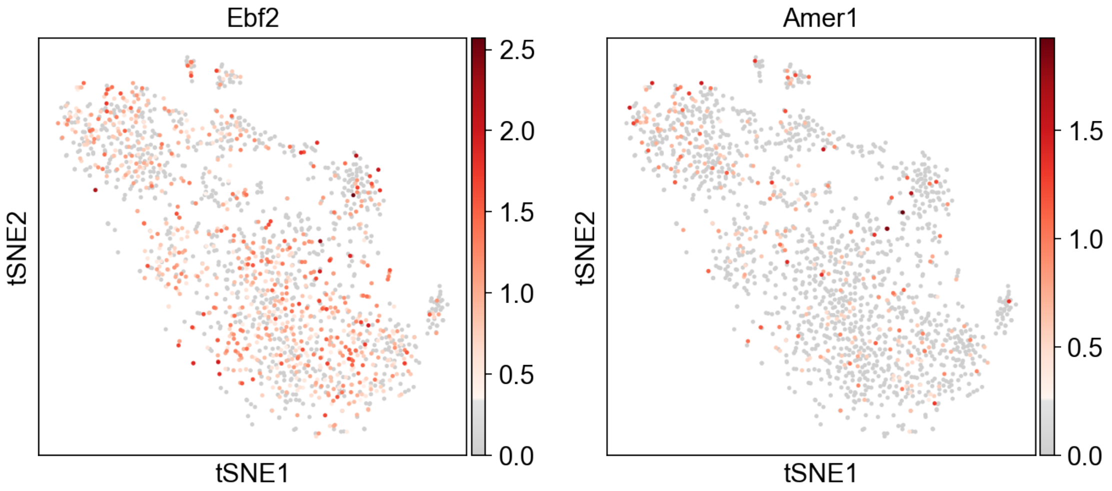

WARNING: saving figure to file
figures/tsne_preadipYoung_markers_GO_adipocyte_dev_6.pdf

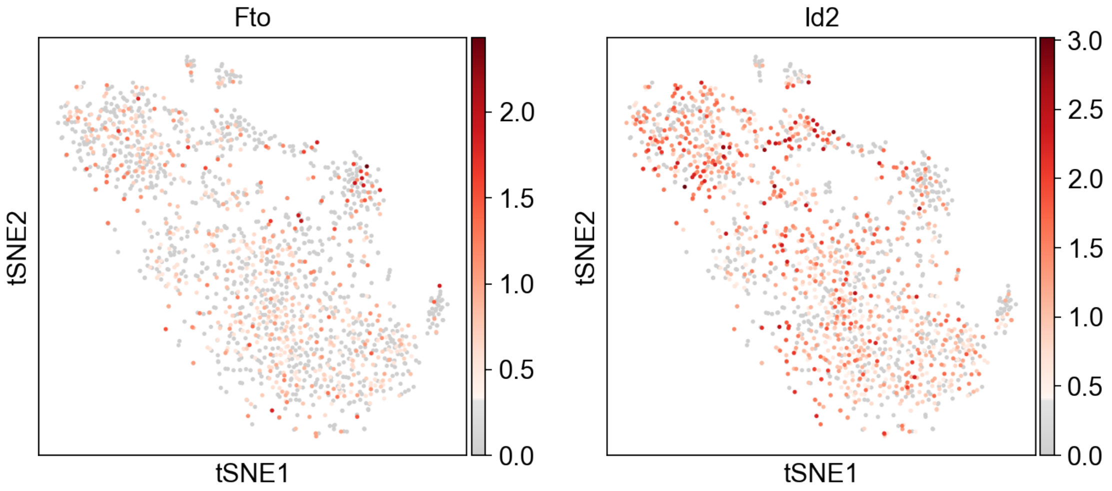

WARNING: saving figure to file
figures/tsne_preadipYoung_markers_GO_adipocyte_dev_7.pdf

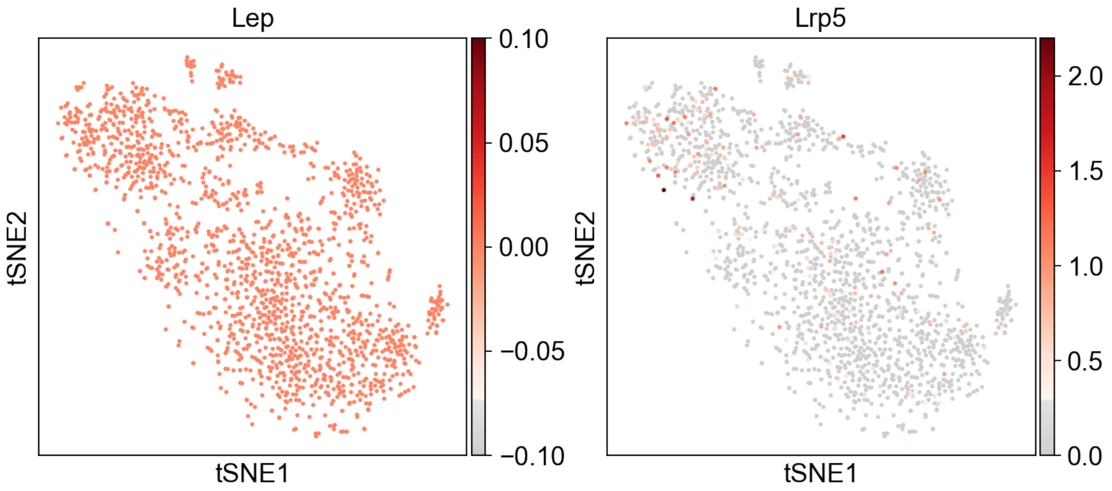

WARNING: saving figure to file
figures/tsne_preadipYoung_markers_GO_adipocyte_dev_8.pdf

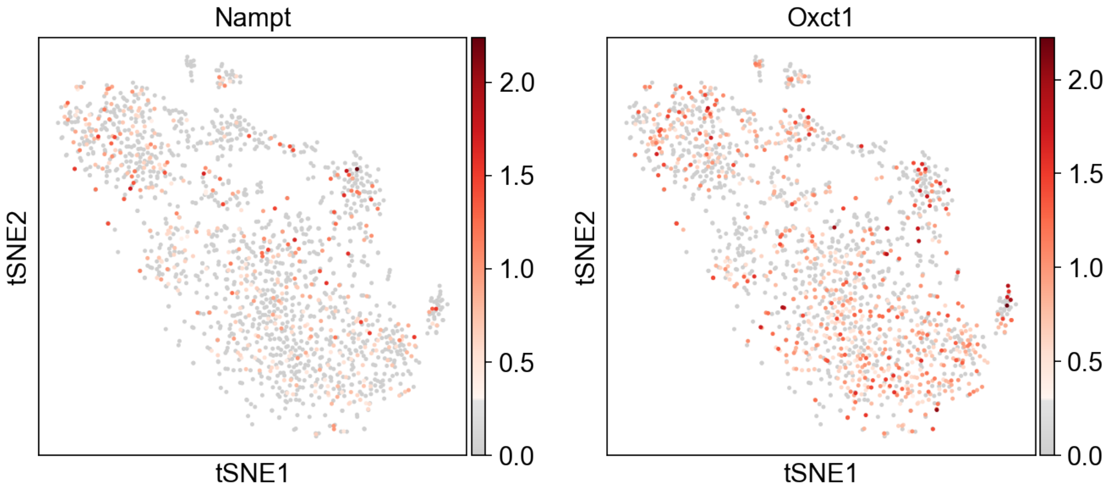

WARNING: saving figure to file
figures/tsne_preadipYoung_markers_GO_adipocyte_dev_9.pdf

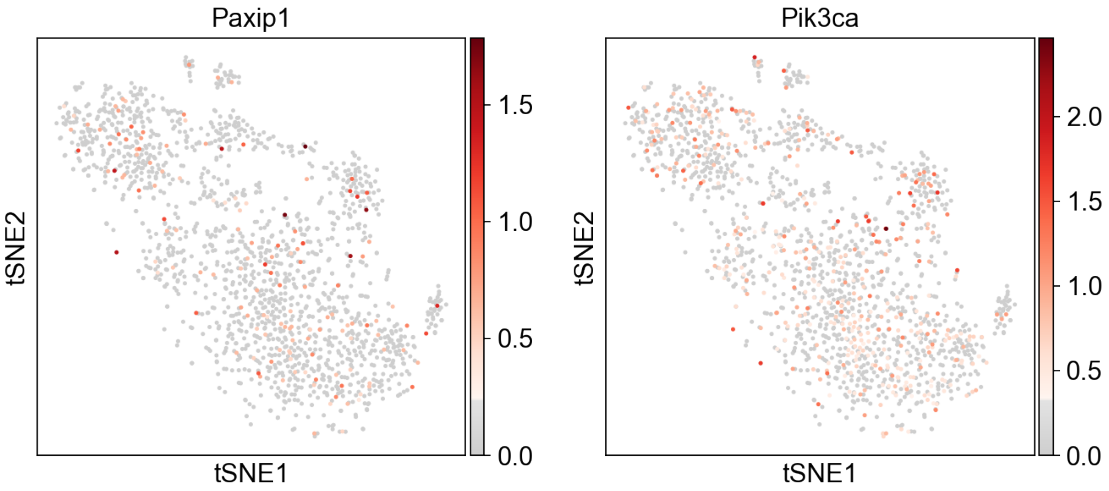


```
WARNING: saving figure to file
figures/tsne_preadipYoung_markers_GO_adipocyte_dev_10.pdf
```

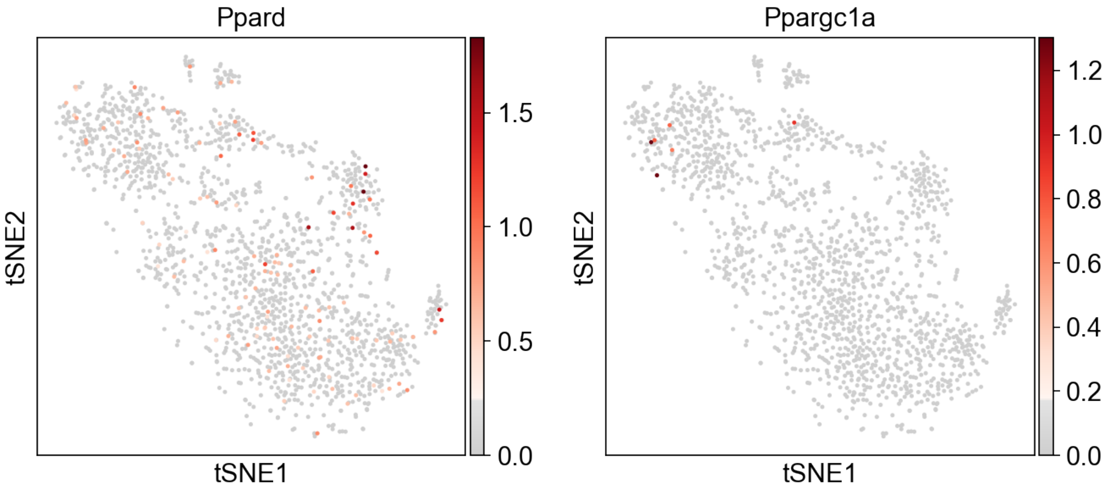


```
WARNING: saving figure to file
figures/tsne_preadipYoung_markers_GO_adipocyte_dev_11.pdf
```

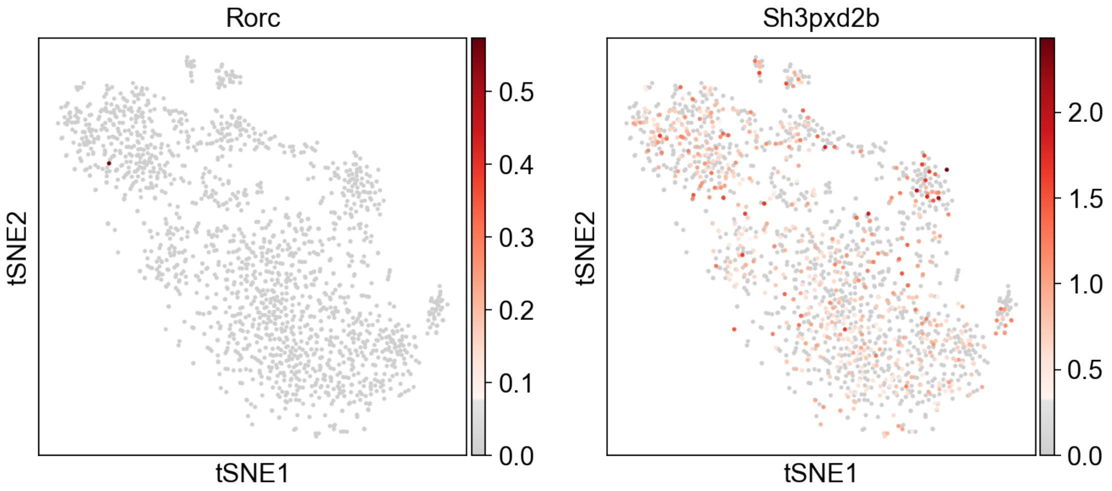


```
WARNING: saving figure to file
figures/tsne_preadipYoung_markers_GO_adipocyte_dev_12.pdf
```

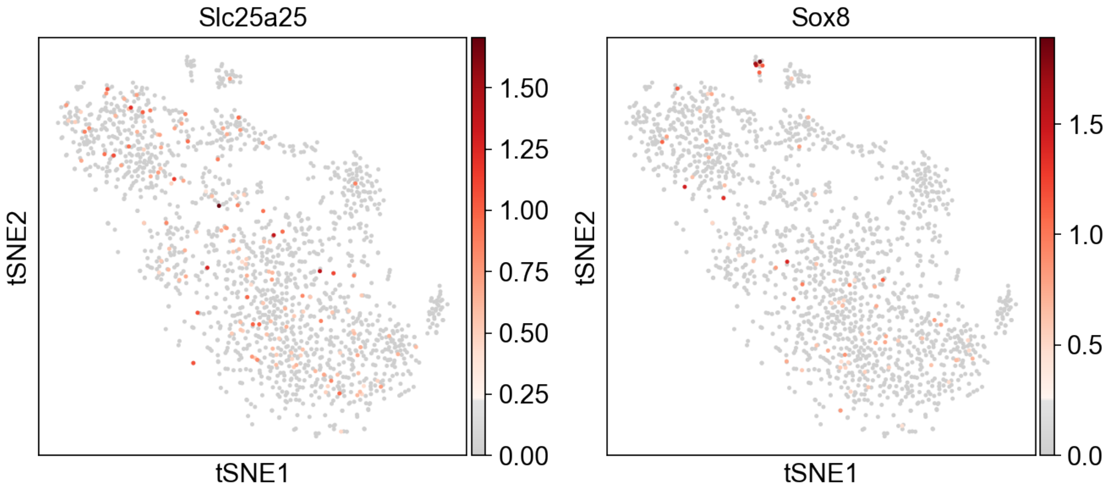


```
WARNING: saving figure to file
figures/tsne_preadipYoung_markers_GO_adipocyte_dev_13.pdf
```

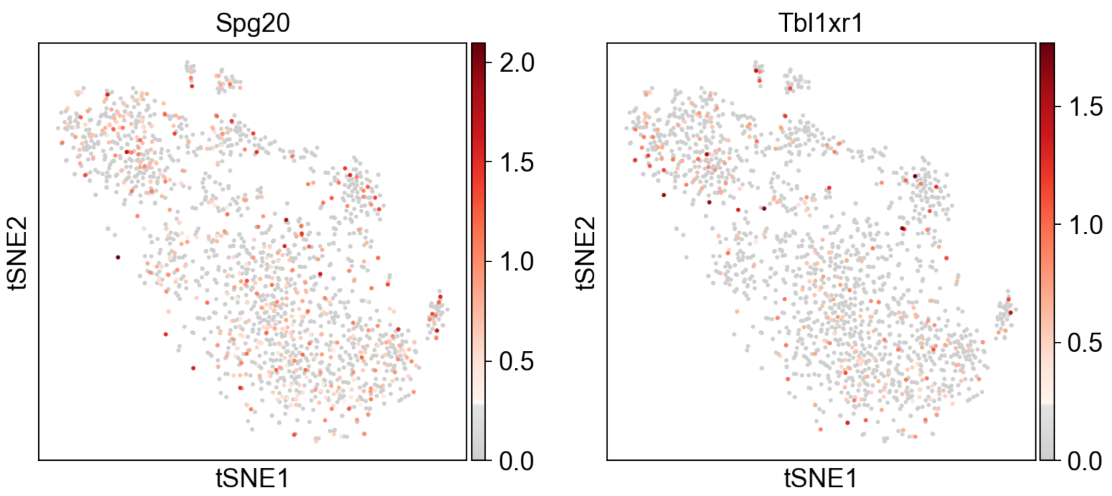


```
WARNING: saving figure to file
figures/tsne_preadipYoung_markers_GO_adipocyte_dev_14.pdf
```

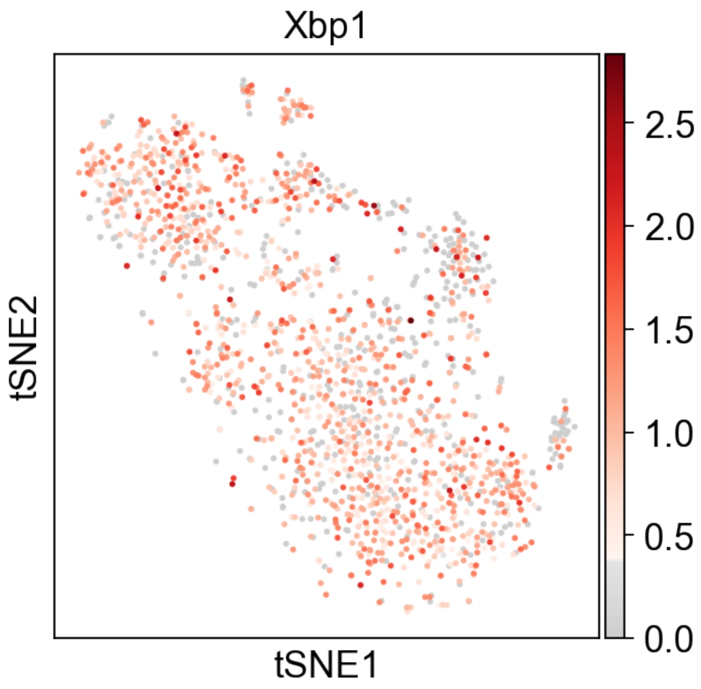

[49]:
```
if bool_plot==True:
    sc.pl.heatmap(
        adata=adata_adip_young,
        var_names=adipocyte_markers,
```

```
        groupby="louvain",
        use_raw=True,
        log=True,
        cmap="viridis",
        vmin=-1,
        vmax=4,
        dendrogram=False,
        var_group_rotation=90,
        show_gene_labels=True,
        show=True,
        save="_preadipYoung_markers_preadipocytes.pdf"
    )
```

```
WARNING: saving figure to file
figures/heatmap_preadipYoung_markers_preadipocytes.pdf
```

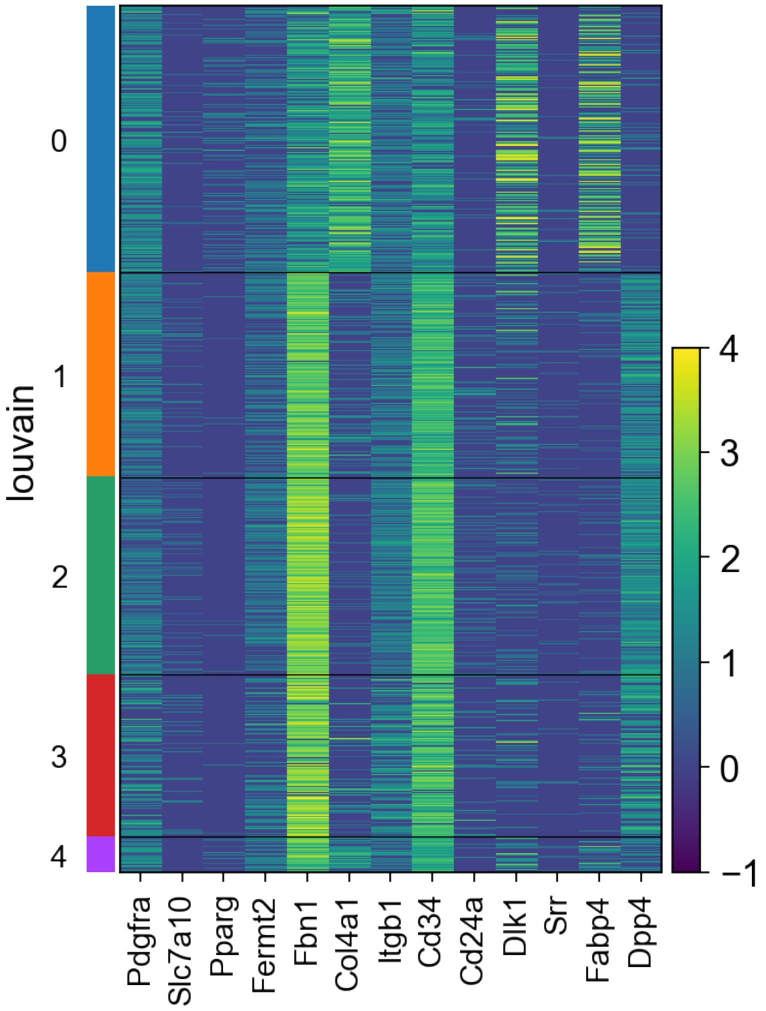

[50]:
```
if bool_plot==True:
    sc.pl.heatmap(
        adata=adata_adip_young,
        var_names=go_adip_dev,
        groupby="louvain",
        use_raw=True,
        log=True,
        cmap="viridis",
        vmin=-1,
        vmax=2,
        dendrogram=False,
        var_group_rotation=90,
        show_gene_labels=True,
        show=True,
        save="_preadipYoung_markers_GO_adipocyte_dev.pdf"
    )
```

```
WARNING: saving figure to file
figures/heatmap_preadipYoung_markers_GO_adipocyte_dev.pdf
```

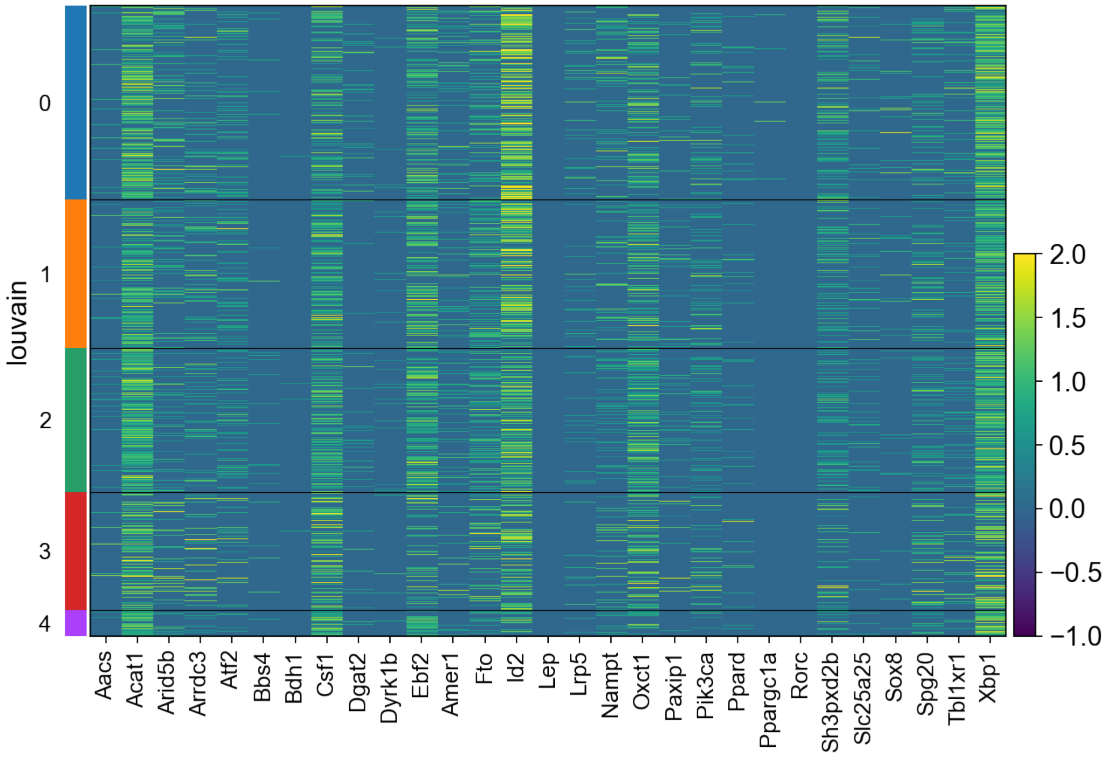

[51]:
```
[{'mean expression of Slc7a10 in louvain group '+x:
  np.mean(adata_adip_young[adata_adip_young.obs['louvain'].values==x,:][:
↪,'Slc7a10'].X)}
```

```
for x in adata_adip_young.obs['louvain'].cat.categories.values]
```

```
/Users/viktorian.miok/anaconda3/lib/python3.7/site-
packages/anndata/core/anndata.py:846: FutureWarning: In anndata v0.7+, arrays
contained within an AnnData object will maintain their dimensionality. For
example, prior to v0.7 `adata[0, 0].X` returned a scalar and `adata[0, :]`
returned a 1d array, post v0.7 they will return two dimensional arrays. If you
would like to get a one dimensional array from your AnnData object, consider
using the `adata.obs_vector`, `adata.var_vector` methods or accessing the array
directly.
  warn_flatten()
```

[51]:
```
[{'mean expression of Slc7a10 in louvain group 0': 0.07535988},
 {'mean expression of Slc7a10 in louvain group 1': 0.13056226},
 {'mean expression of Slc7a10 in louvain group 2': 0.1828374},
 {'mean expression of Slc7a10 in louvain group 3': 0.1417397},
 {'mean expression of Slc7a10 in louvain group 4': 0.00809834}]
```

This seems to be a continuum of cell types with two opposing gradients of gene expression: Pdgfra and Slc7a10. This could indicate a developmental lineage.

7.2.2 Young mouse - coarse clustering

[52]:
```
if bool_plot==True:
    plot_violin_marker(adata_adip_young_lowres, adipocyte_markers,␣
 ↪save="_preadipYoung_lowres_markers_preadipocytes")
```

```
WARNING: saving figure to file
figures/violin_preadipYoung_lowres_markers_preadipocytes_0.pdf
```

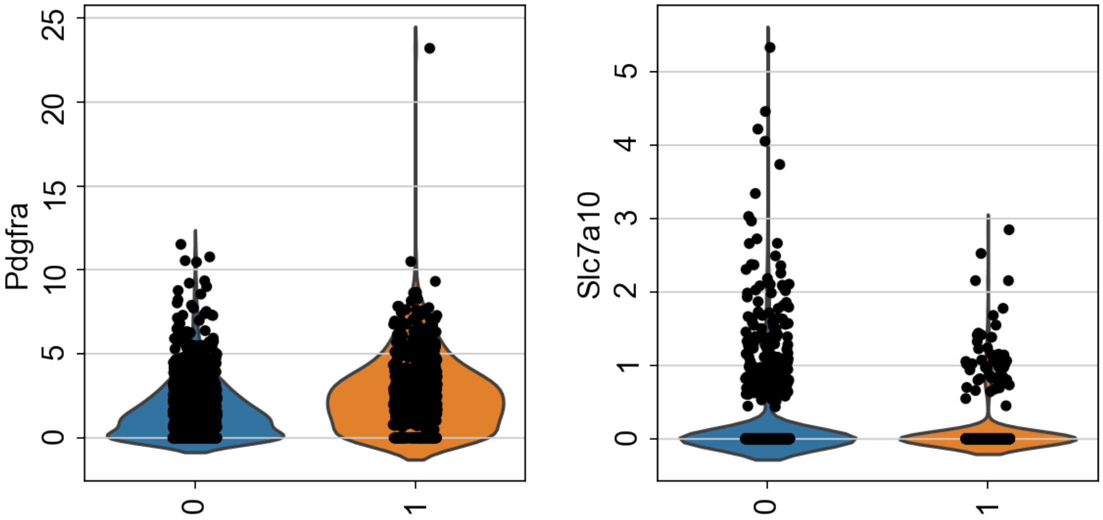

WARNING: saving figure to file
figures/violin_preadipYoung_lowres_markers_preadipocytes_1.pdf

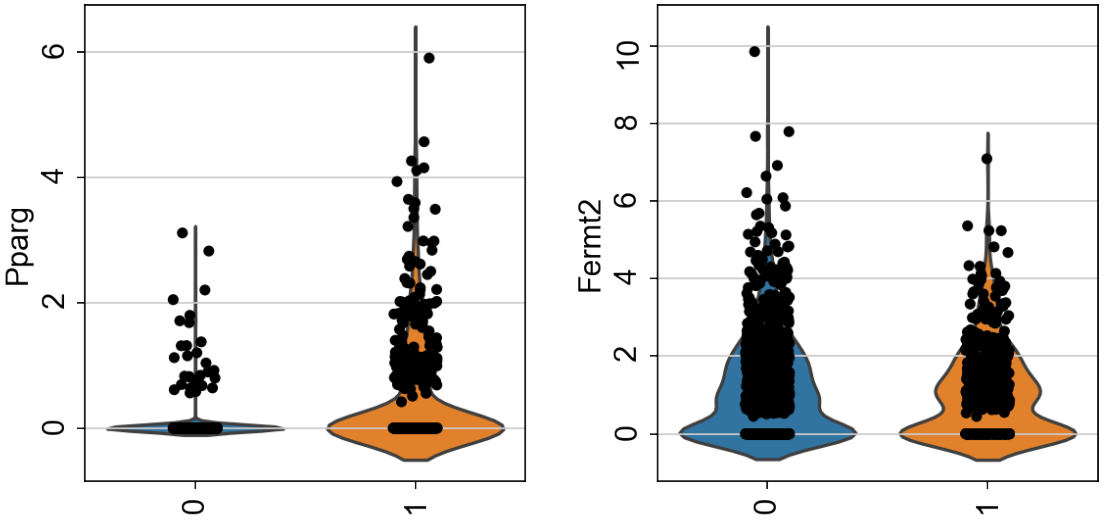

WARNING: saving figure to file
figures/violin_preadipYoung_lowres_markers_preadipocytes_2.pdf

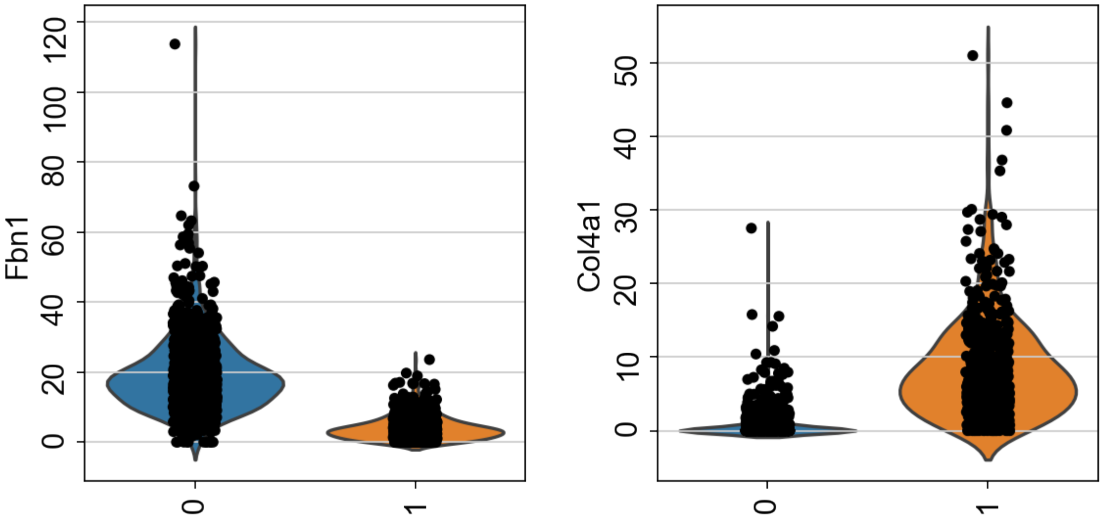

WARNING: saving figure to file
figures/violin_preadipYoung_lowres_markers_preadipocytes_3.pdf

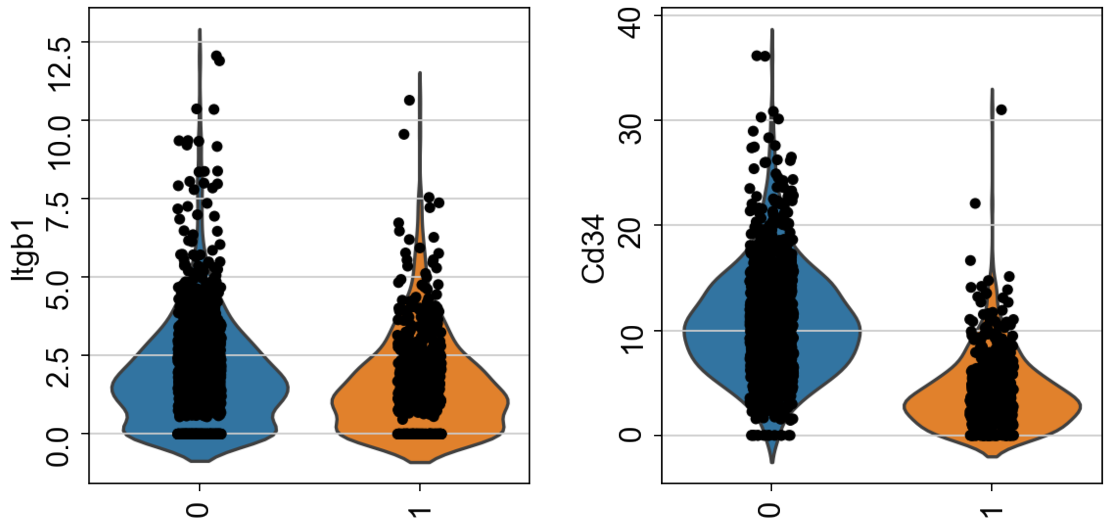


```
WARNING: saving figure to file
figures/violin_preadipYoung_lowres_markers_preadipocytes_4.pdf
```

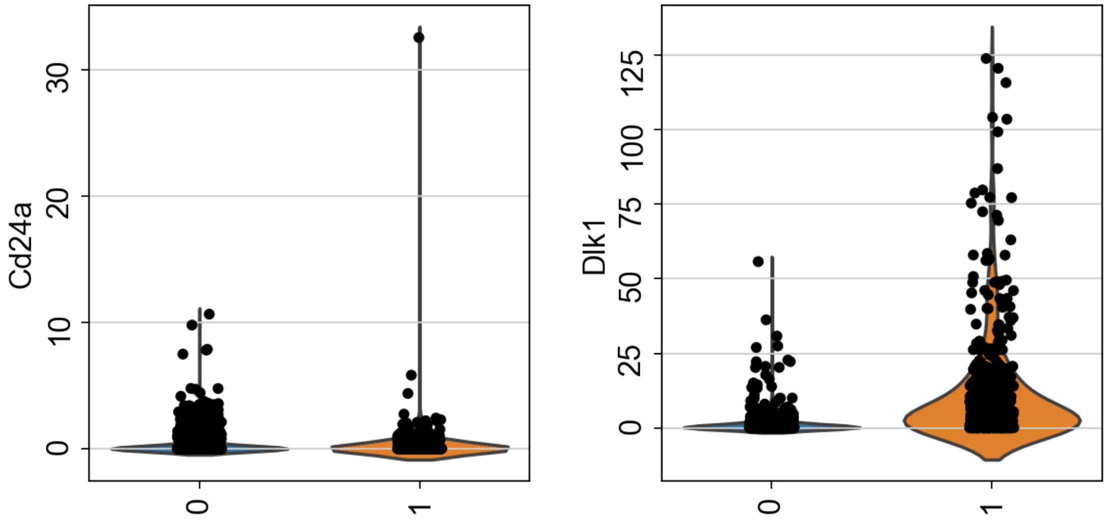


```
WARNING: saving figure to file
figures/violin_preadipYoung_lowres_markers_preadipocytes_5.pdf
```

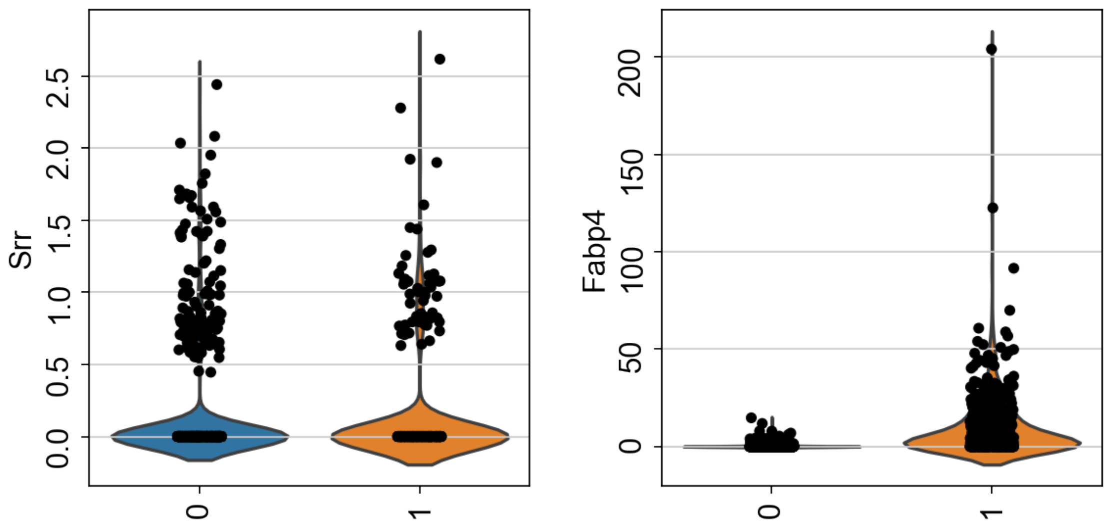


```
WARNING: saving figure to file
figures/violin_preadipYoung_lowres_markers_preadipocytes_6.pdf
```

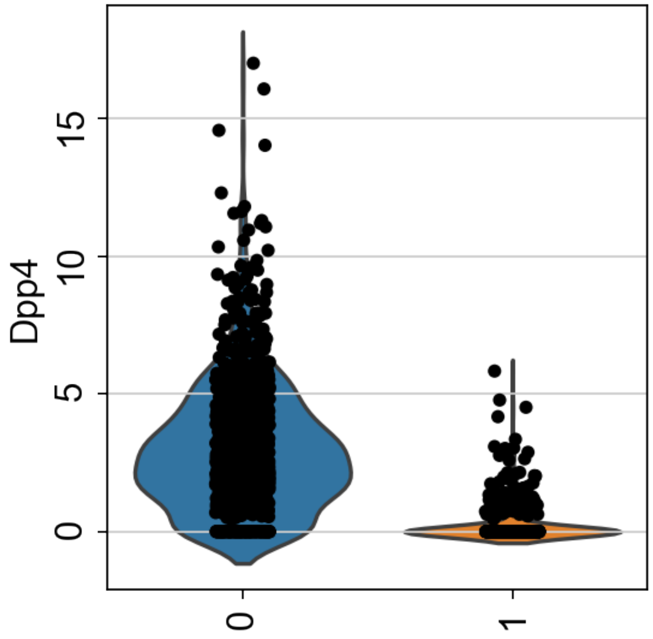

[53]:
```python
if bool_plot==True:
    plot_violin_marker(adata_adip_young_lowres, go_adip_dev,␣
 ↪save="_preadipYoung_lowres_markers_GO_adipocyte_dev")
```

```
WARNING: saving figure to file
```

figures/violin_preadipYoung_lowres_markers_GO_adipocyte_dev_0.pdf

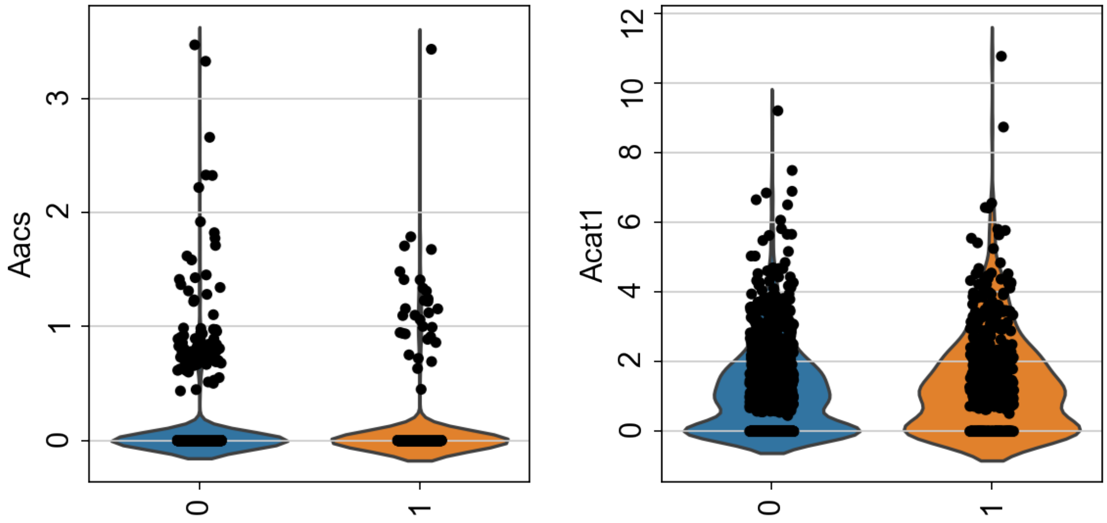

WARNING: saving figure to file
figures/violin_preadipYoung_lowres_markers_GO_adipocyte_dev_1.pdf

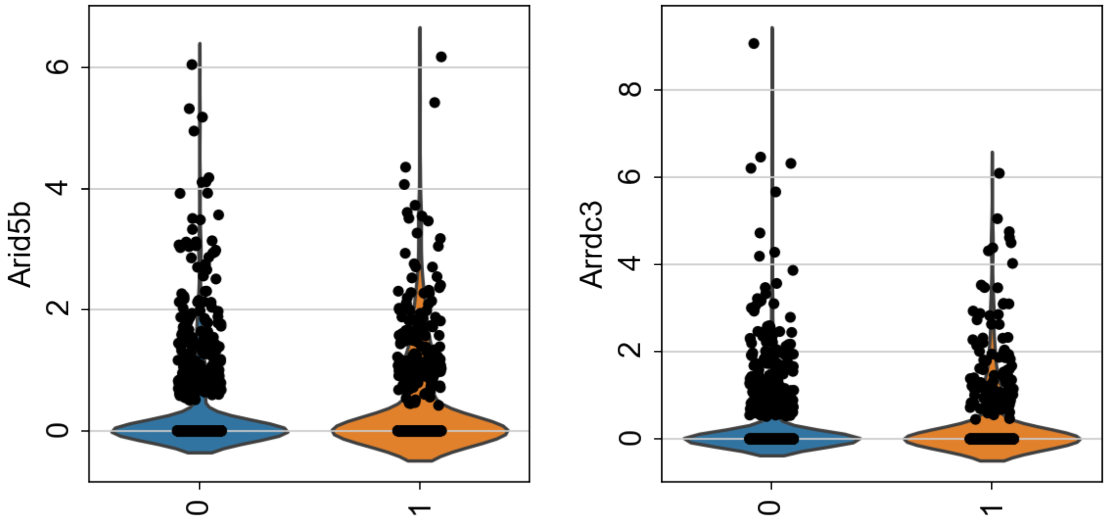

WARNING: saving figure to file
figures/violin_preadipYoung_lowres_markers_GO_adipocyte_dev_2.pdf

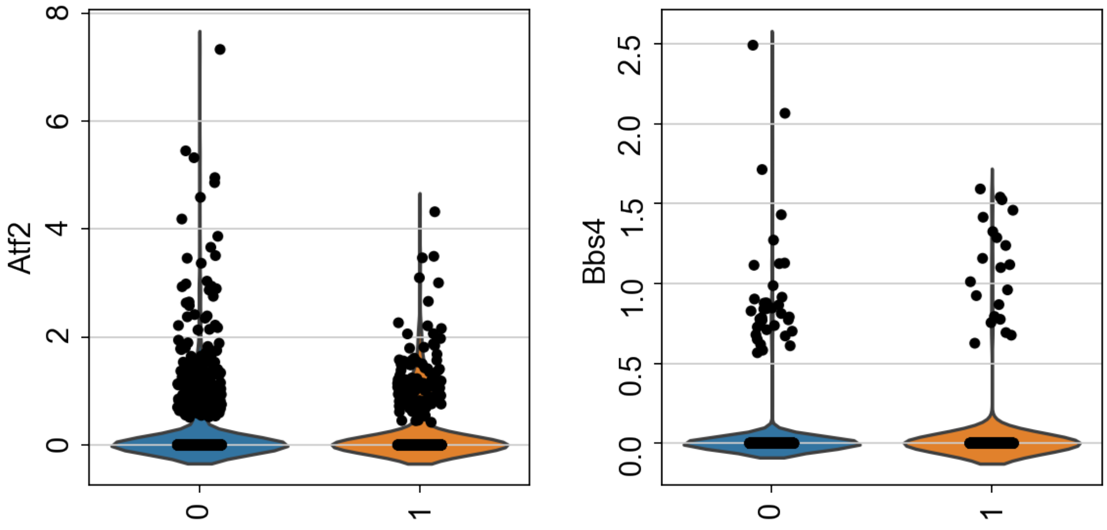


```
WARNING: saving figure to file
figures/violin_preadipYoung_lowres_markers_GO_adipocyte_dev_3.pdf
```

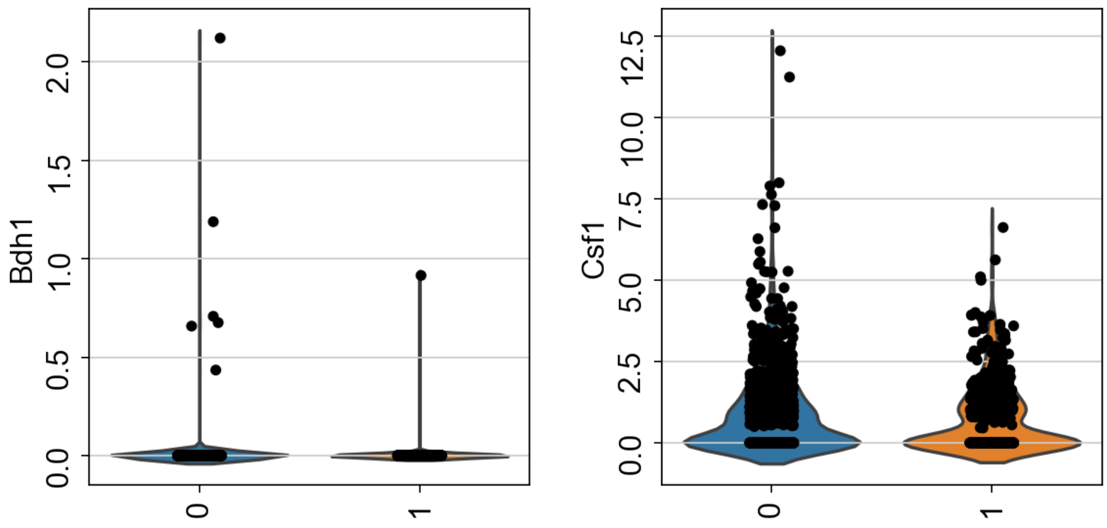


```
WARNING: saving figure to file
figures/violin_preadipYoung_lowres_markers_GO_adipocyte_dev_4.pdf
```

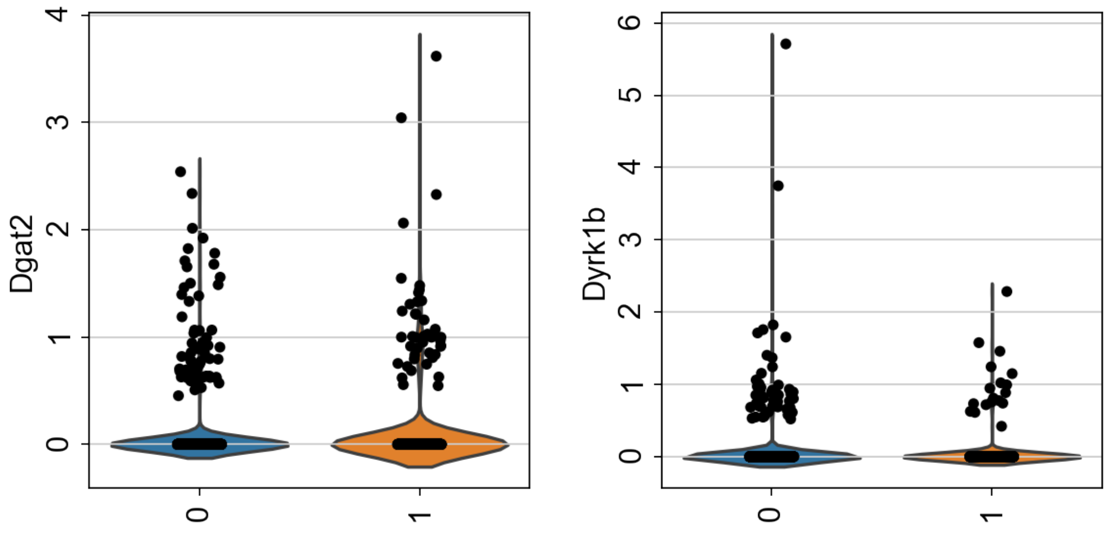

WARNING: saving figure to file
figures/violin_preadipYoung_lowres_markers_GO_adipocyte_dev_5.pdf

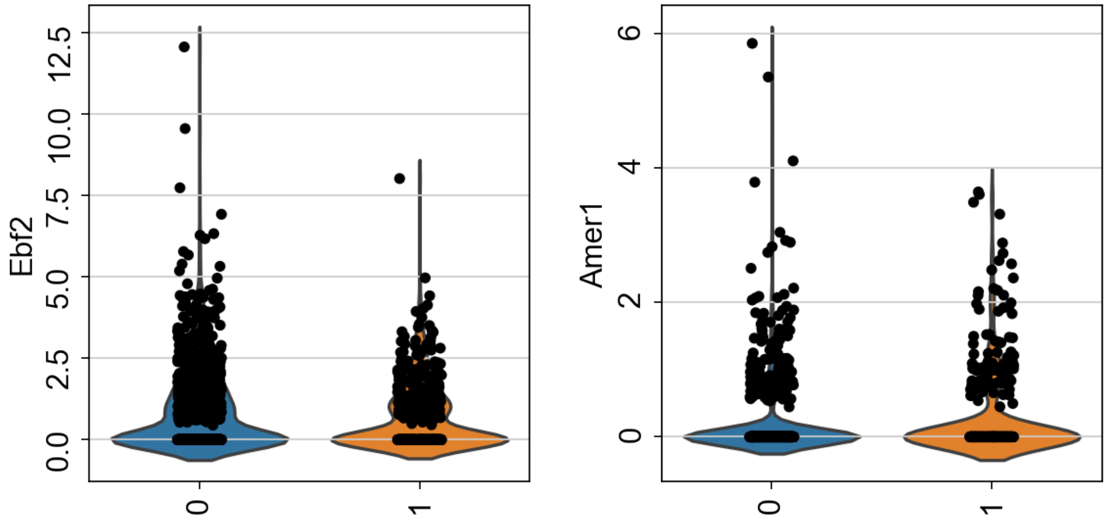

WARNING: saving figure to file
figures/violin_preadipYoung_lowres_markers_GO_adipocyte_dev_6.pdf

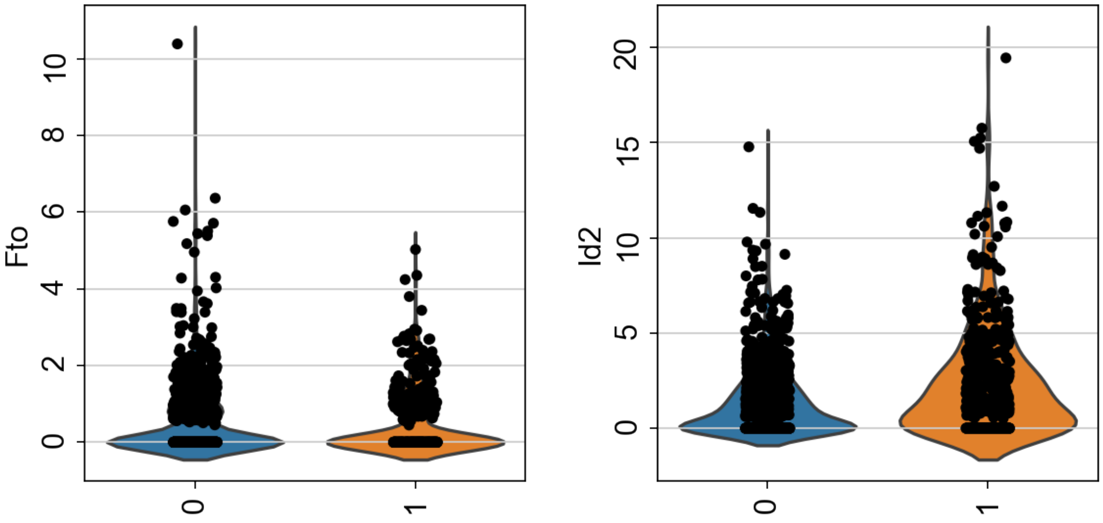

WARNING: saving figure to file
figures/violin_preadipYoung_lowres_markers_GO_adipocyte_dev_7.pdf

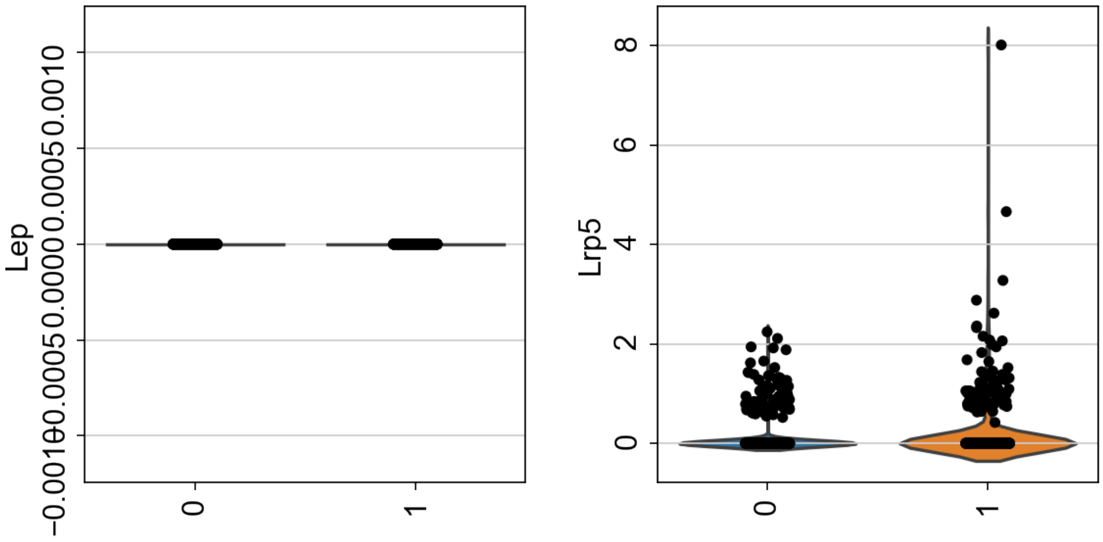

WARNING: saving figure to file
figures/violin_preadipYoung_lowres_markers_GO_adipocyte_dev_8.pdf

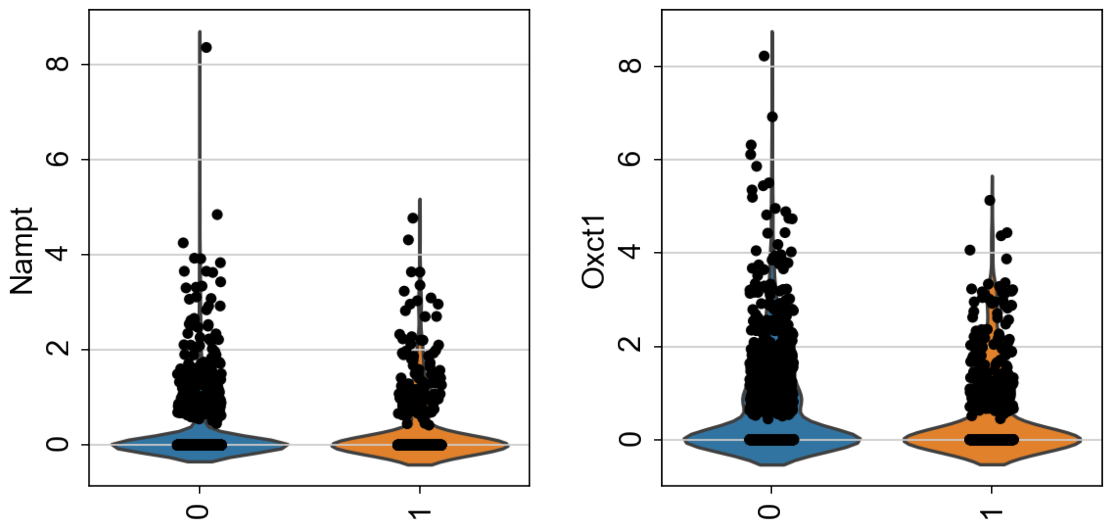

WARNING: saving figure to file
figures/violin_preadipYoung_lowres_markers_GO_adipocyte_dev_9.pdf

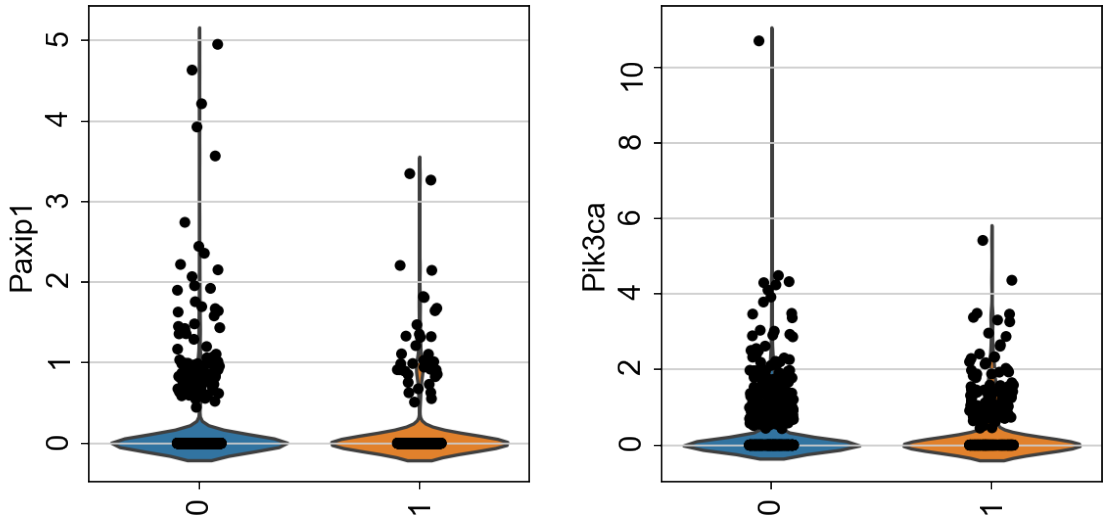

WARNING: saving figure to file
figures/violin_preadipYoung_lowres_markers_GO_adipocyte_dev_10.pdf

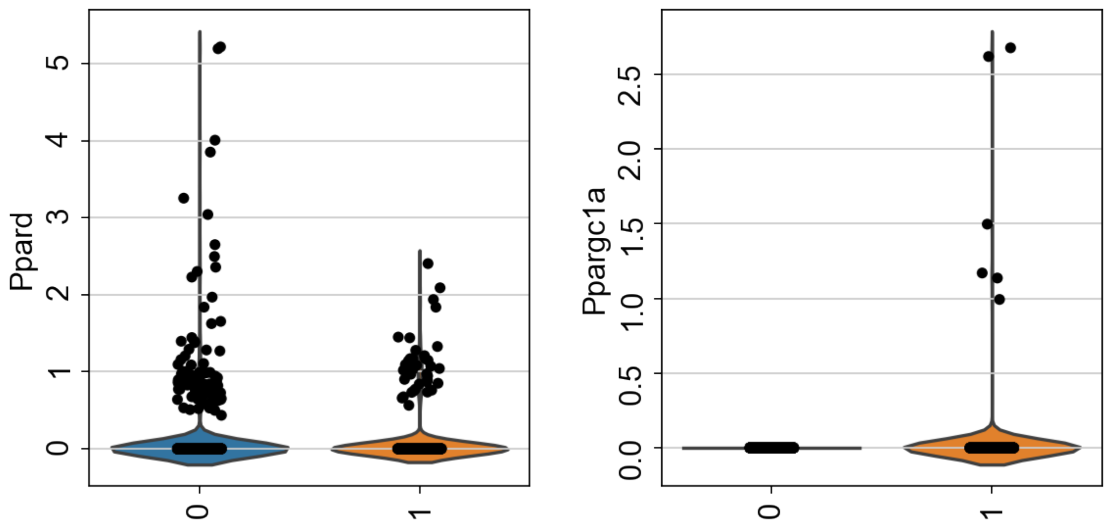


```
WARNING: saving figure to file
figures/violin_preadipYoung_lowres_markers_GO_adipocyte_dev_11.pdf
```

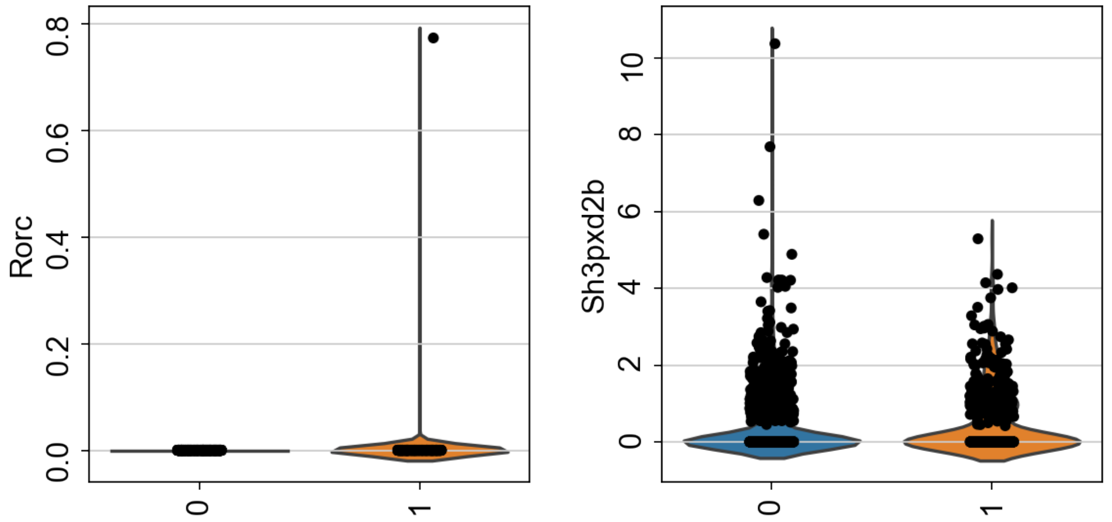


```
WARNING: saving figure to file
figures/violin_preadipYoung_lowres_markers_GO_adipocyte_dev_12.pdf
```

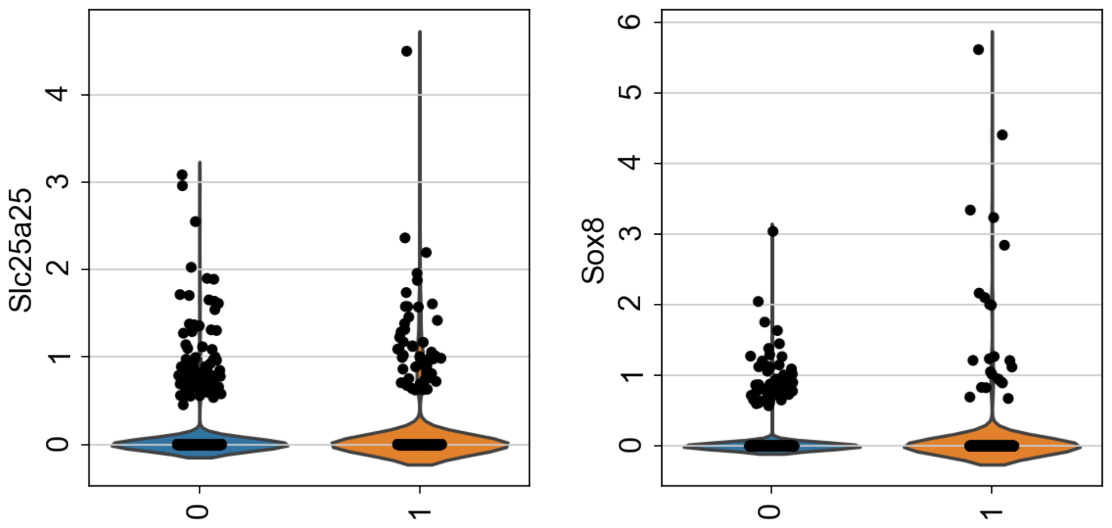

WARNING: saving figure to file
figures/violin_preadipYoung_lowres_markers_GO_adipocyte_dev_13.pdf

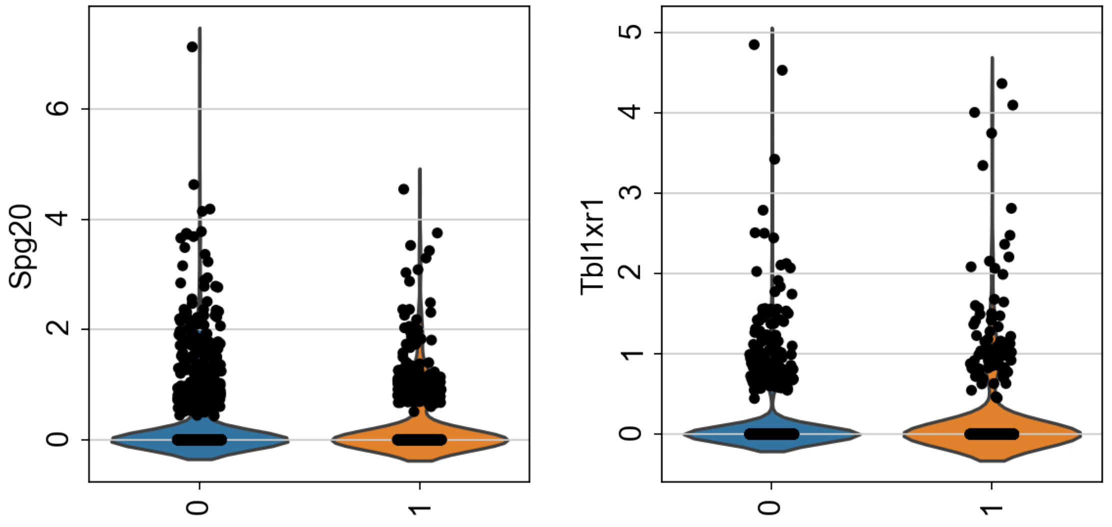

WARNING: saving figure to file
figures/violin_preadipYoung_lowres_markers_GO_adipocyte_dev_14.pdf

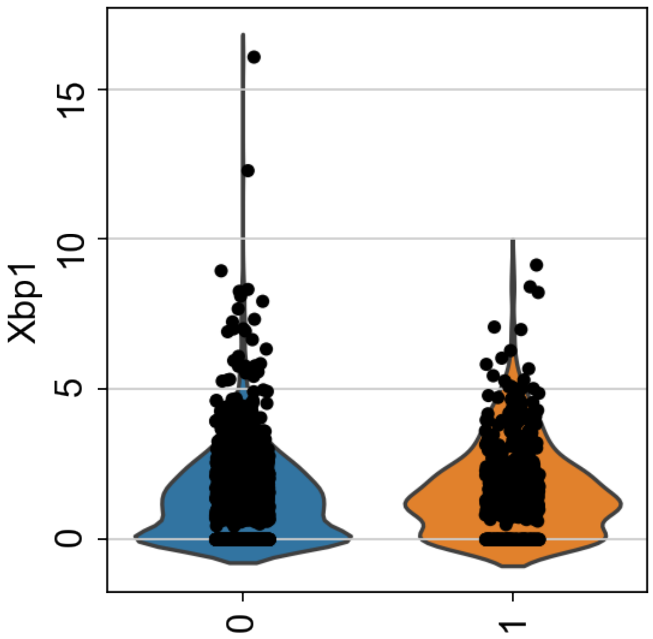

[54]:
```
if bool_plot==True:
    sc.pl.heatmap(
        adata=adata_adip_young_lowres,
        var_names=adipocyte_markers,
        groupby="louvain",
        use_raw=True,
        log=True,
        dendrogram=False,
        var_group_rotation=90,
        show_gene_labels=True,
        show=True,
        save="_preadipYoung_lowres_markers_preadipocytes.pdf"
    )
```

```
WARNING: saving figure to file
figures/heatmap_preadipYoung_lowres_markers_preadipocytes.pdf
```

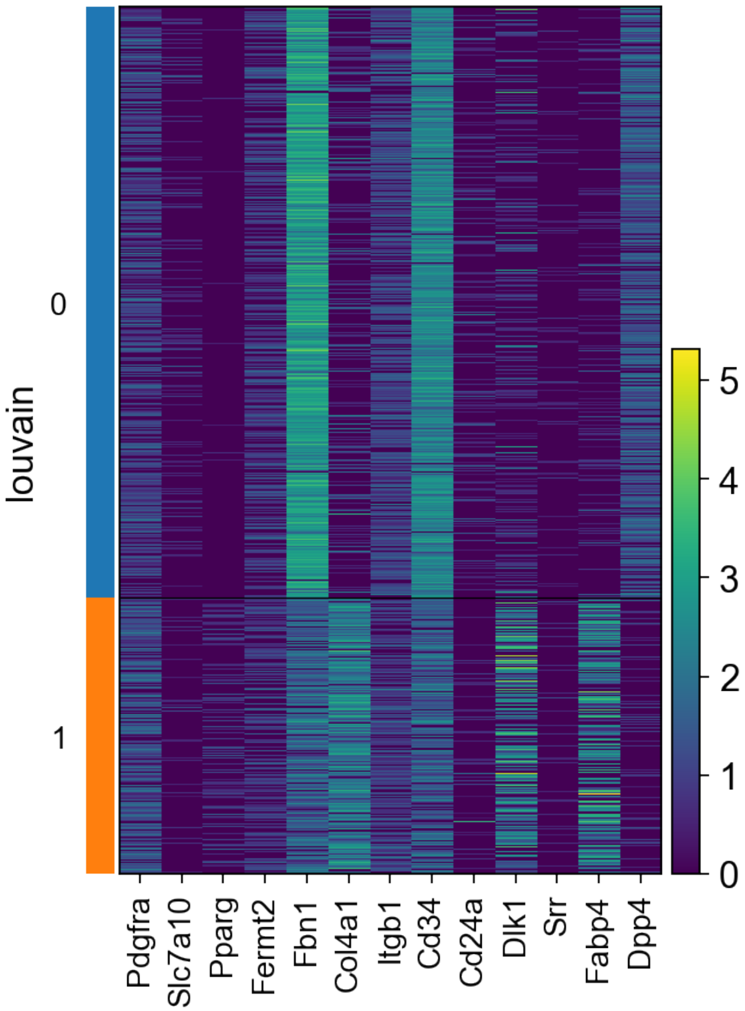

[55]:

```
if bool_plot==True:
    sc.pl.heatmap(
        adata=adata_adip_young_lowres,
        var_names=go_adip_dev,
        groupby="louvain",
        use_raw=True,
        log=True,
        dendrogram=False,
        var_group_rotation=90,
        show_gene_labels=True,
        show=True,
        save="_preadipYoung_lowres_markers_GO_adipocyte_dev.pdf"
    )
```

```
WARNING: saving figure to file
figures/heatmap_preadipYoung_lowres_markers_GO_adipocyte_dev.pdf
```

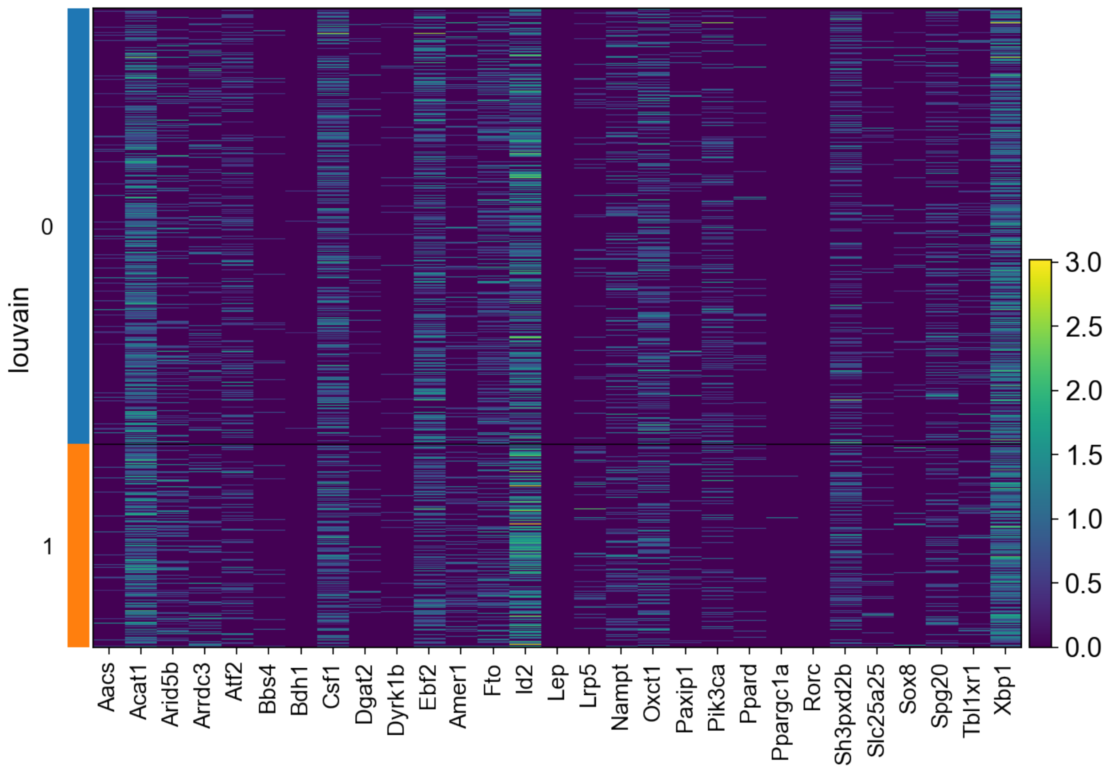

7.2.3 Old mouse

[56]:
```
if bool_plot==True:
    plot_violin_marker(adata_adip_old, adipocyte_markers,␣
↪save="_preadipOld_markers_preadipocytes")
```

```
WARNING: saving figure to file
figures/violin_preadipOld_markers_preadipocytes_0.pdf
```

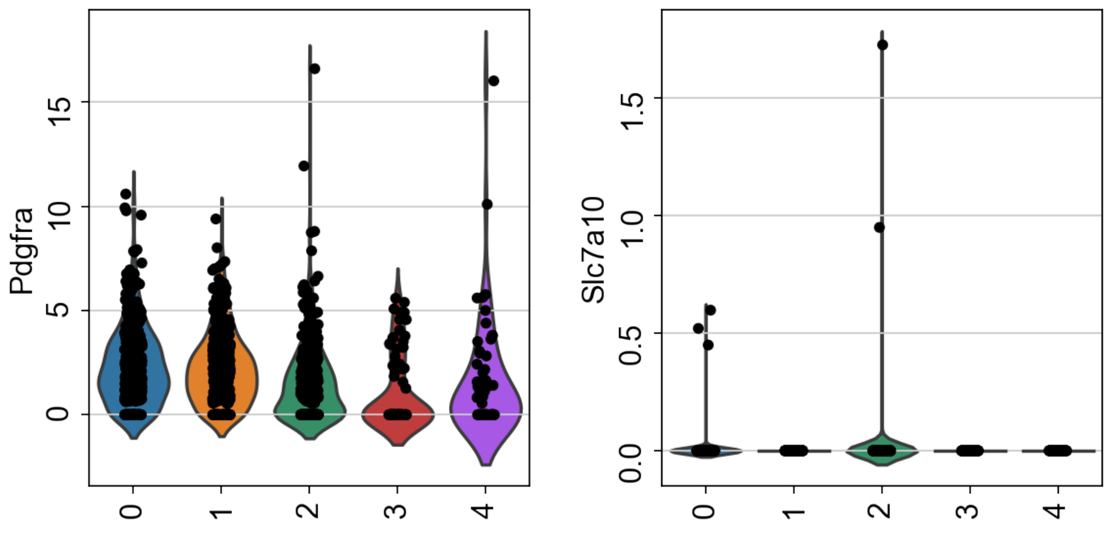

WARNING: saving figure to file
figures/violin_preadipOld_markers_preadipocytes_1.pdf

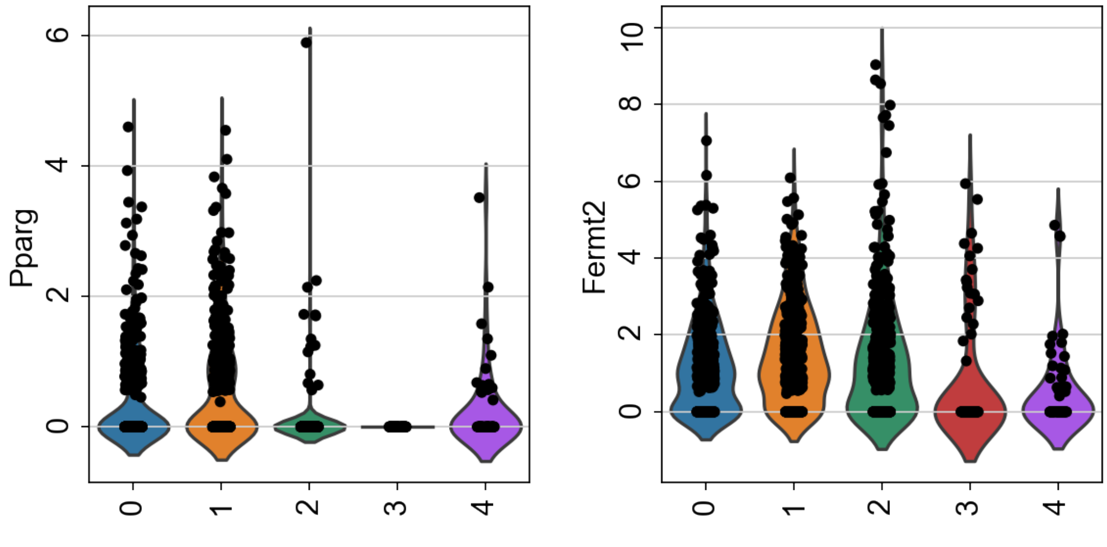

WARNING: saving figure to file
figures/violin_preadipOld_markers_preadipocytes_2.pdf

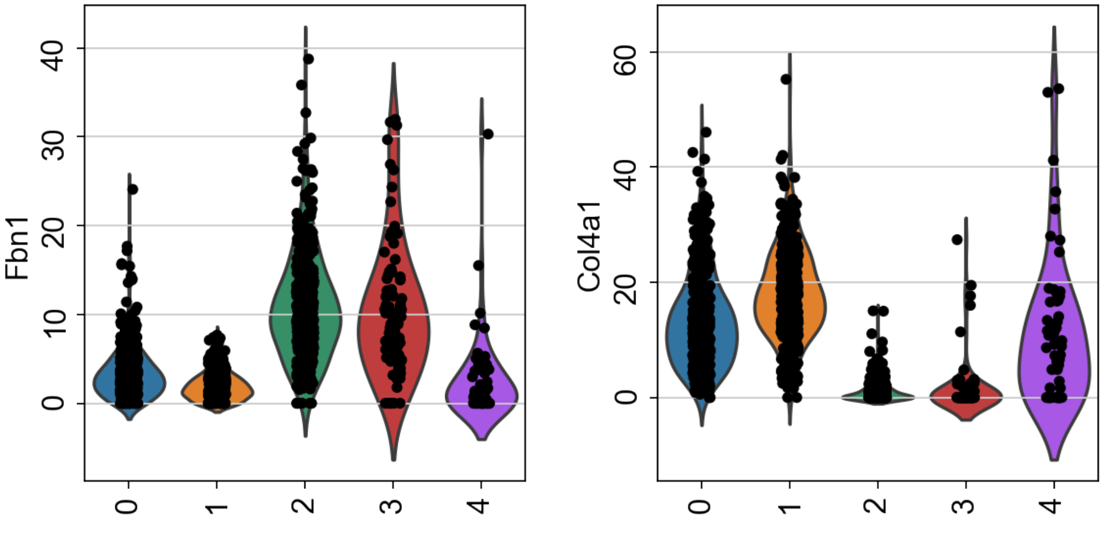

WARNING: saving figure to file
figures/violin_preadipOld_markers_preadipocytes_3.pdf

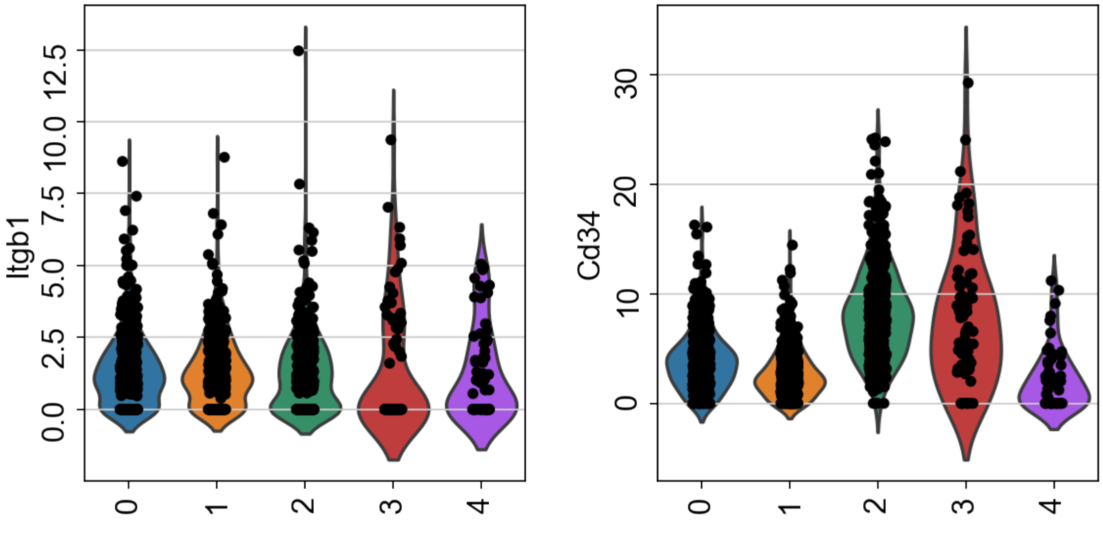

WARNING: saving figure to file
figures/violin_preadipOld_markers_preadipocytes_4.pdf

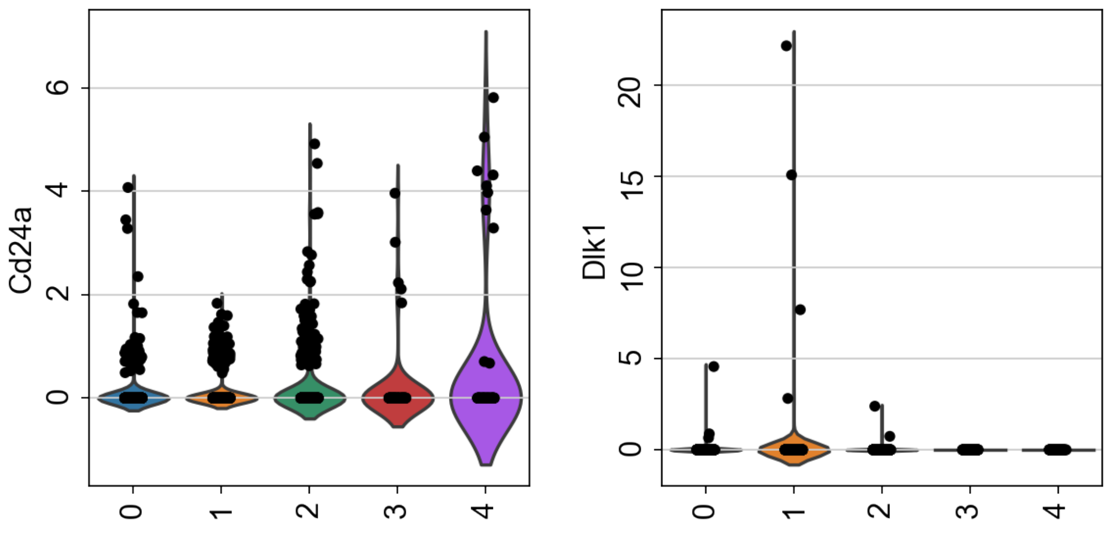

WARNING: saving figure to file
figures/violin_preadipOld_markers_preadipocytes_5.pdf

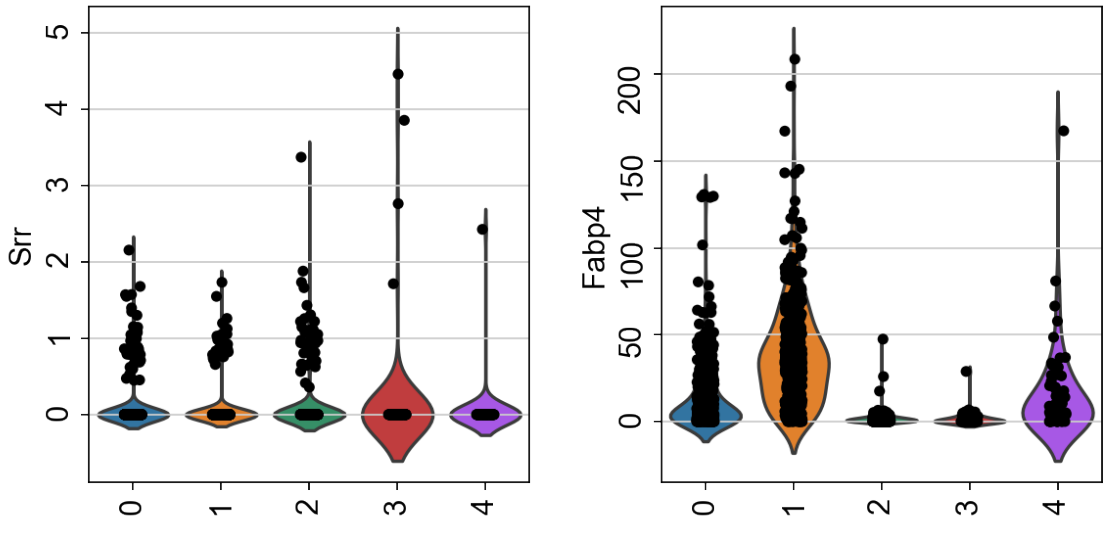

WARNING: saving figure to file
figures/violin_preadipOld_markers_preadipocytes_6.pdf

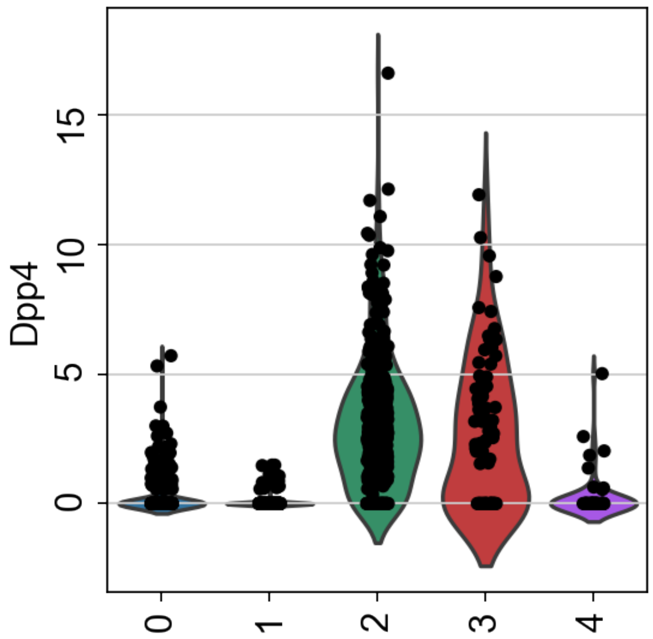

[57]:
```
if bool_plot==True:
    plot_violin_marker(adata_adip_old, go_adip_dev,␣
↪save="_preadipOld_markers_GO_adipocyte_dev")
```

```
WARNING: saving figure to file
figures/violin_preadipOld_markers_GO_adipocyte_dev_0.pdf
```

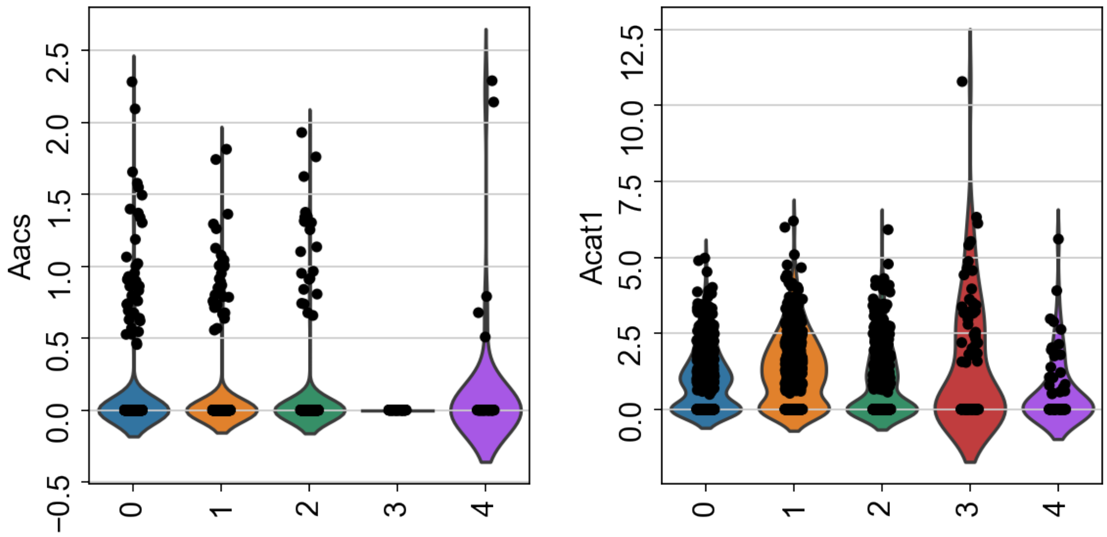


```
WARNING: saving figure to file
```

```
figures/violin_preadipOld_markers_GO_adipocyte_dev_1.pdf
```

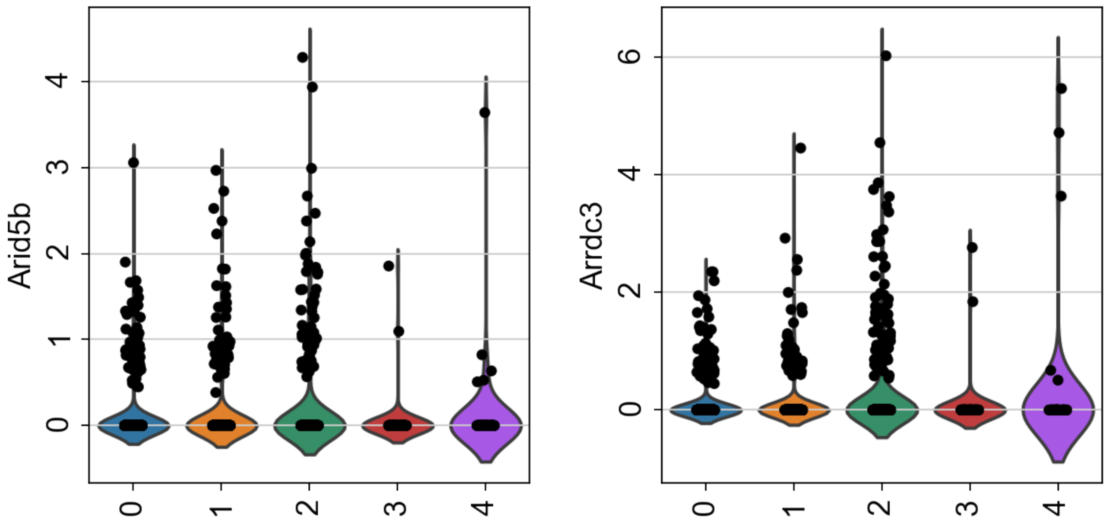


```
WARNING: saving figure to file
figures/violin_preadipOld_markers_GO_adipocyte_dev_2.pdf
```

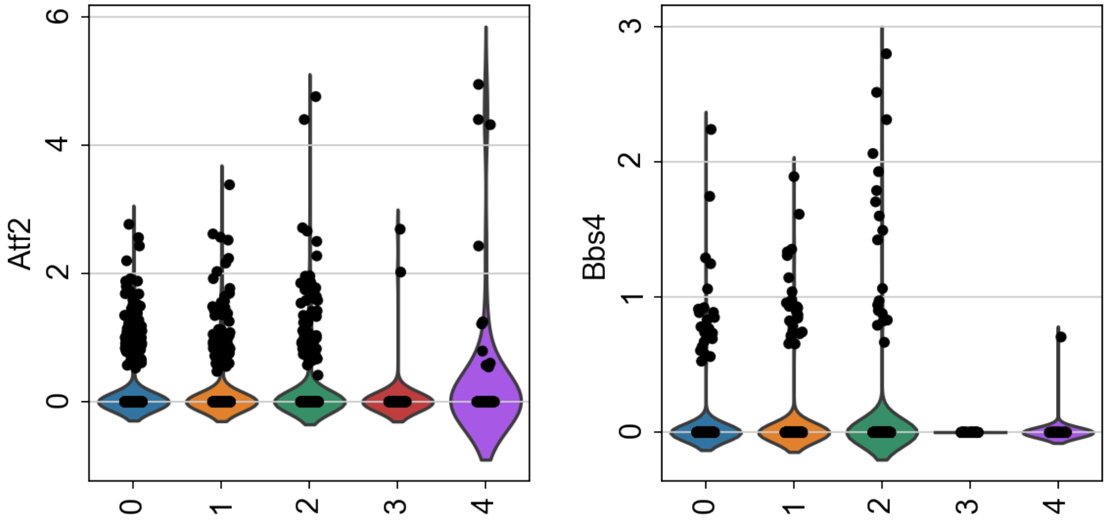


```
WARNING: saving figure to file
figures/violin_preadipOld_markers_GO_adipocyte_dev_3.pdf
```

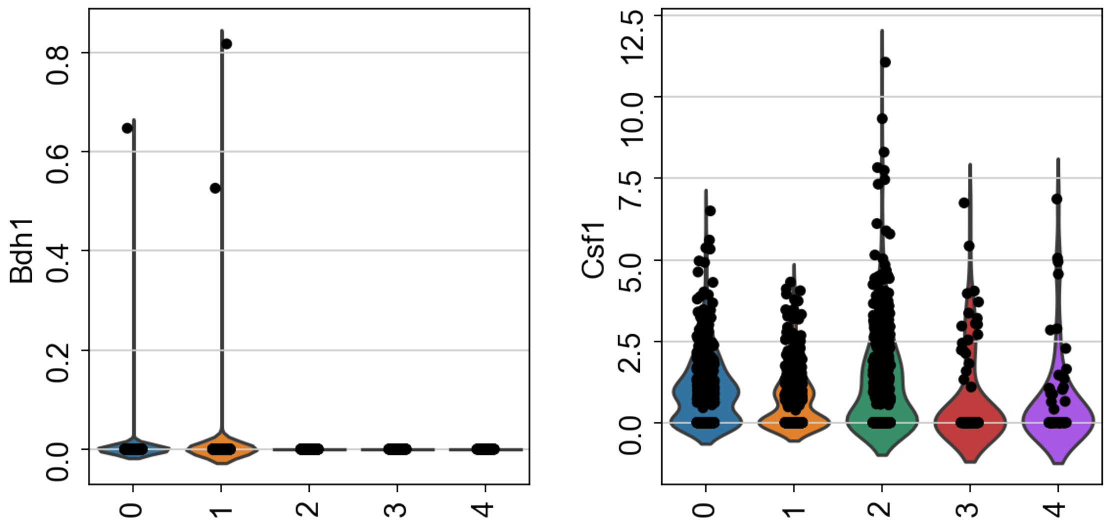

WARNING: saving figure to file
figures/violin_preadipOld_markers_GO_adipocyte_dev_4.pdf

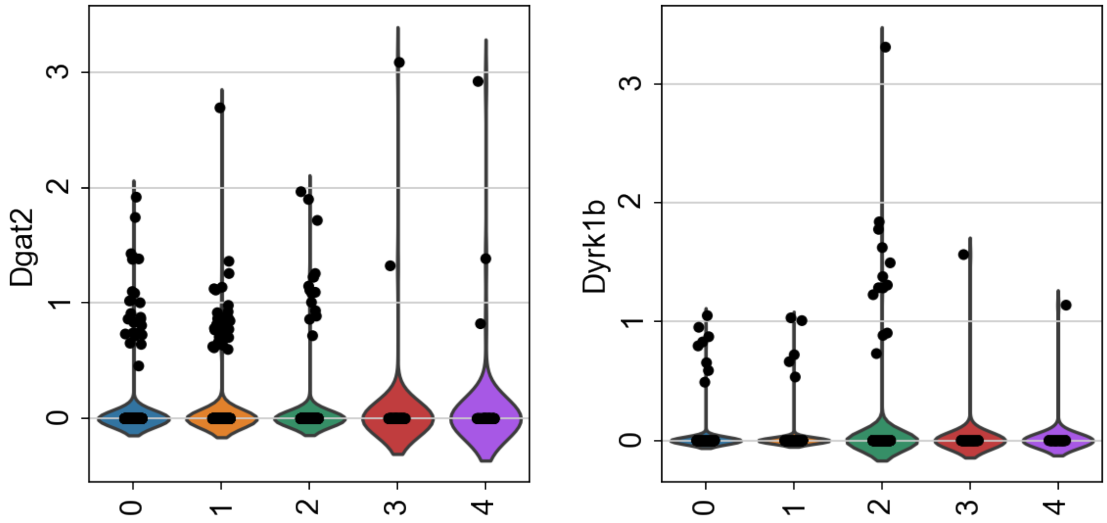

WARNING: saving figure to file
figures/violin_preadipOld_markers_GO_adipocyte_dev_5.pdf

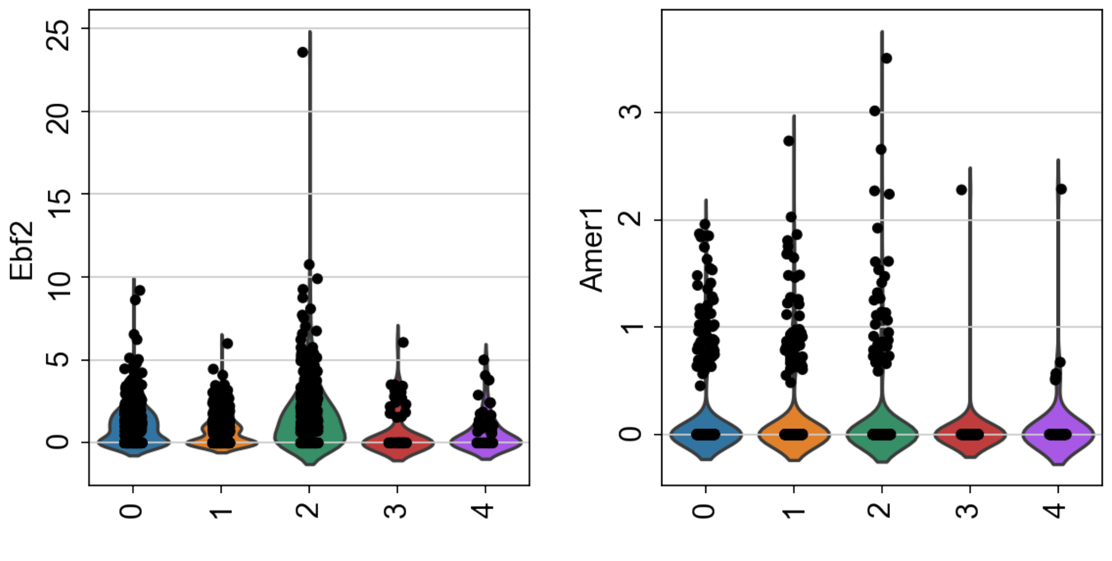

WARNING: saving figure to file
figures/violin_preadipOld_markers_GO_adipocyte_dev_6.pdf

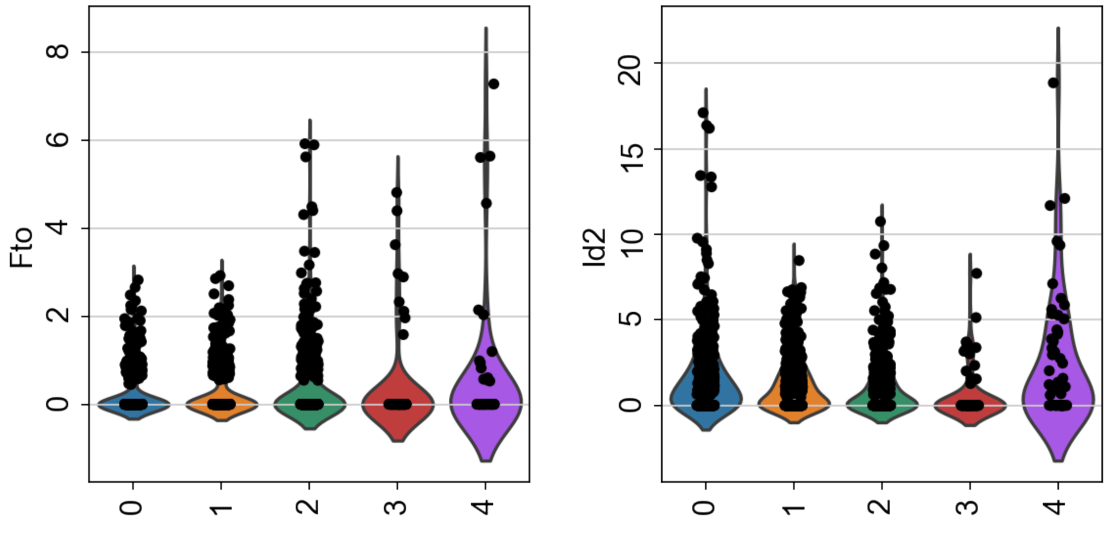

WARNING: saving figure to file
figures/violin_preadipOld_markers_GO_adipocyte_dev_7.pdf

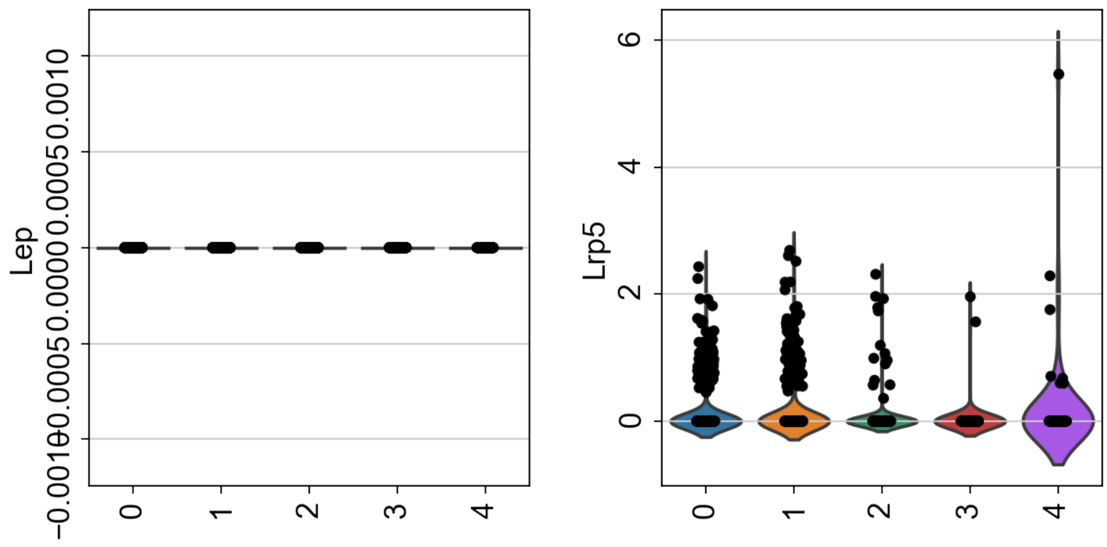

WARNING: saving figure to file
figures/violin_preadipOld_markers_GO_adipocyte_dev_8.pdf

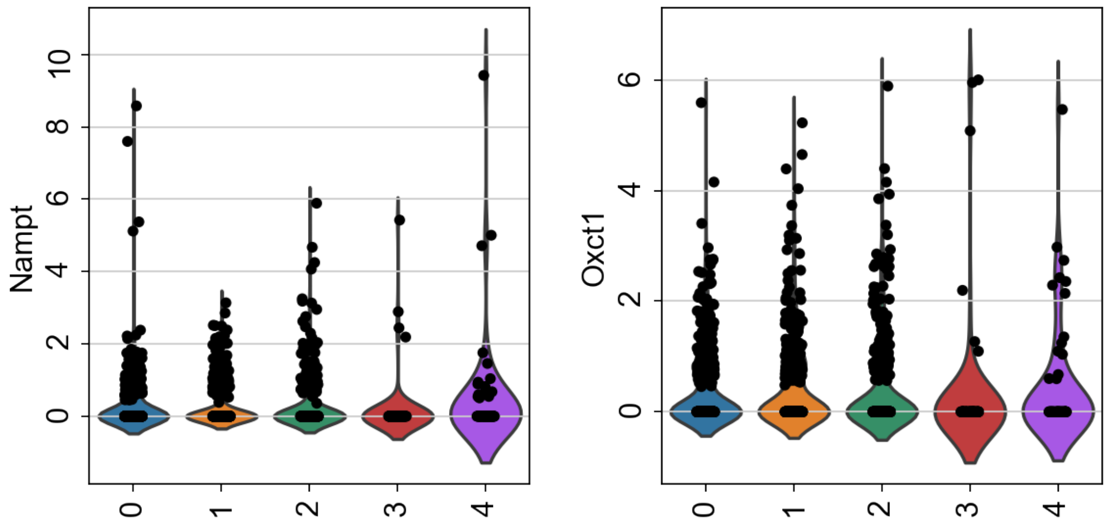

WARNING: saving figure to file
figures/violin_preadipOld_markers_GO_adipocyte_dev_9.pdf

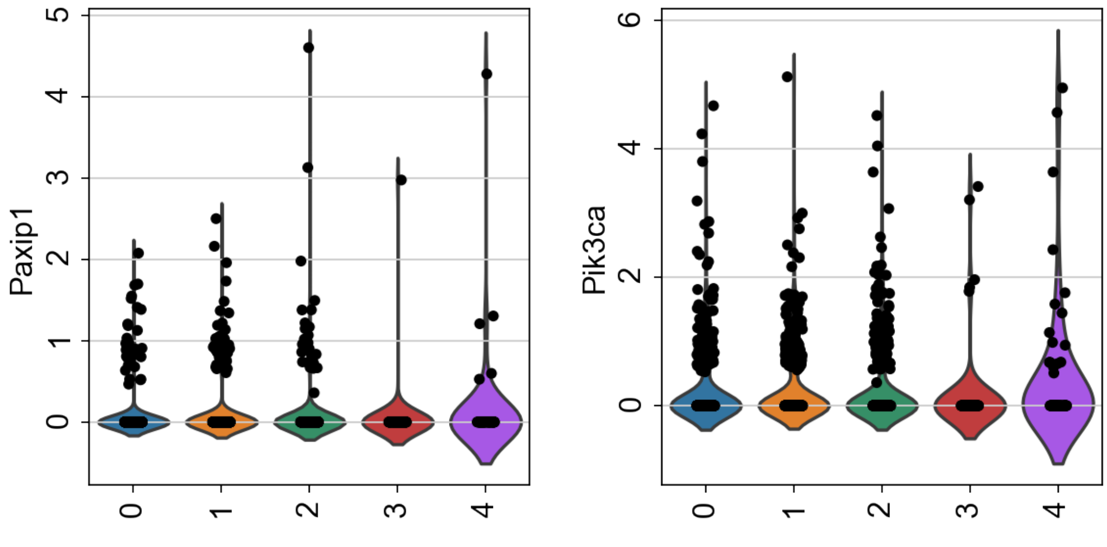

WARNING: saving figure to file
figures/violin_preadipOld_markers_GO_adipocyte_dev_10.pdf

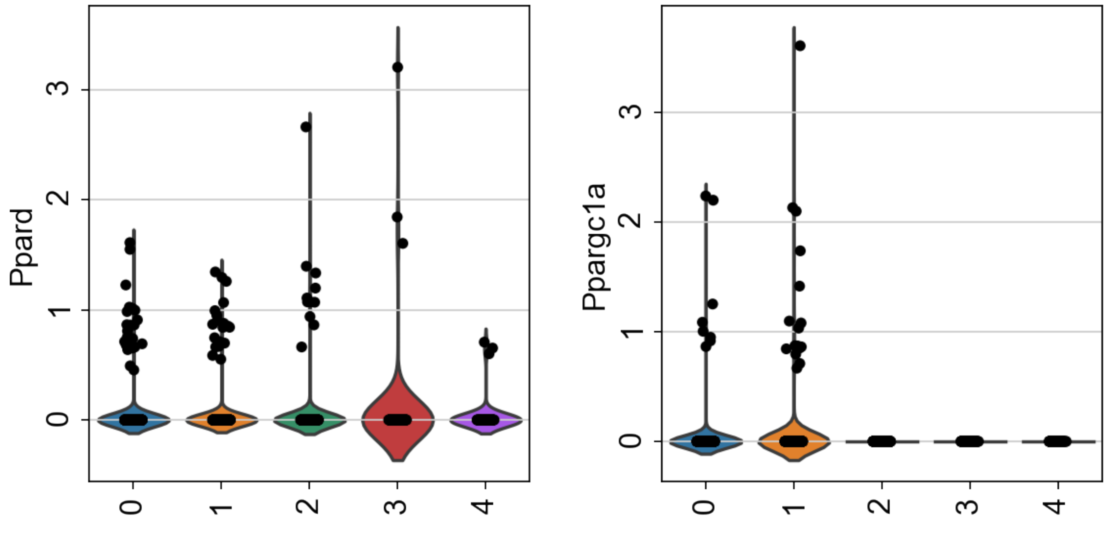

WARNING: saving figure to file
figures/violin_preadipOld_markers_GO_adipocyte_dev_11.pdf

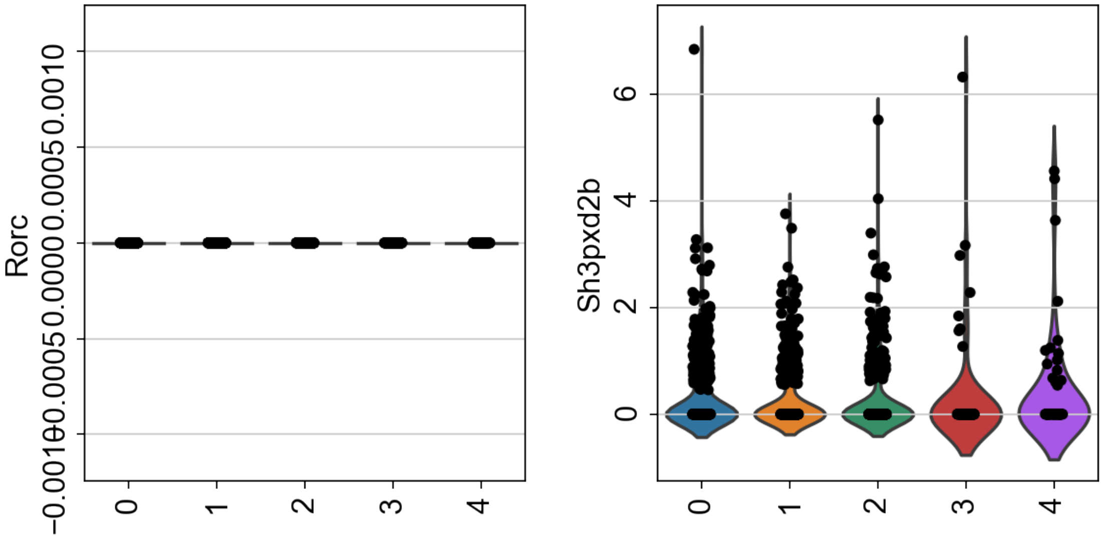

WARNING: saving figure to file
figures/violin_preadipOld_markers_GO_adipocyte_dev_12.pdf

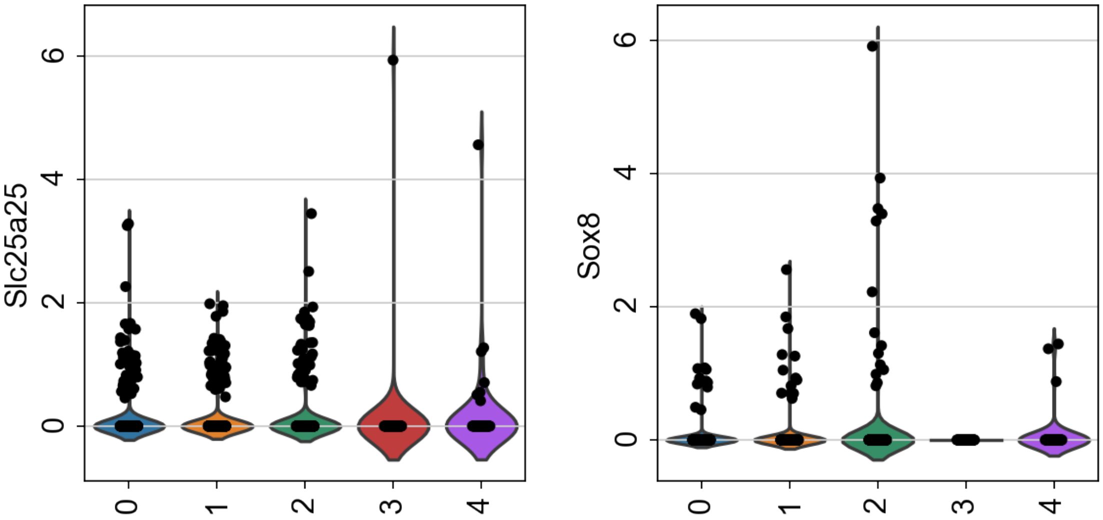

WARNING: saving figure to file
figures/violin_preadipOld_markers_GO_adipocyte_dev_13.pdf

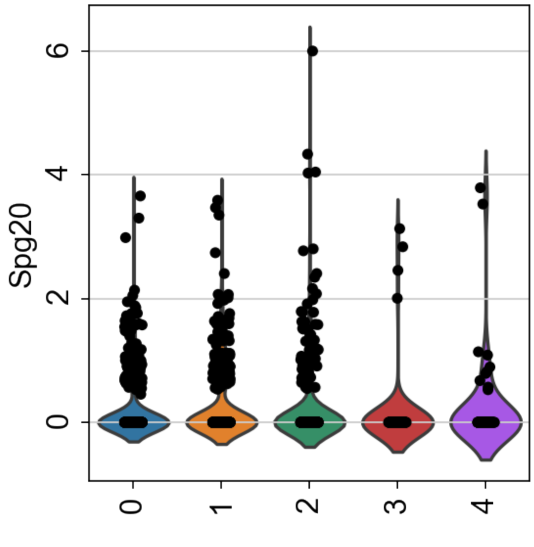

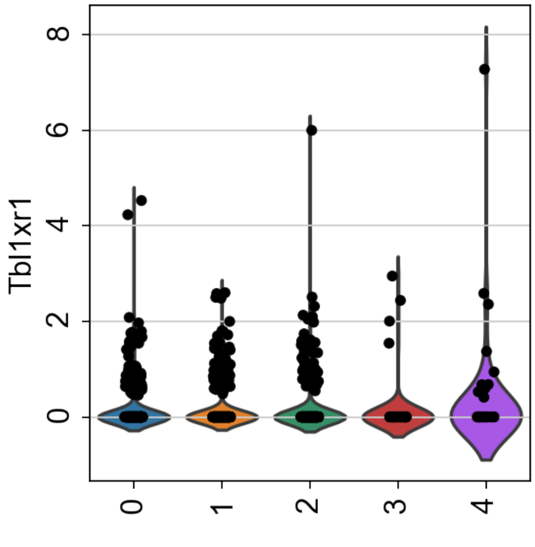


```
WARNING: saving figure to file
figures/violin_preadipOld_markers_GO_adipocyte_dev_14.pdf
```

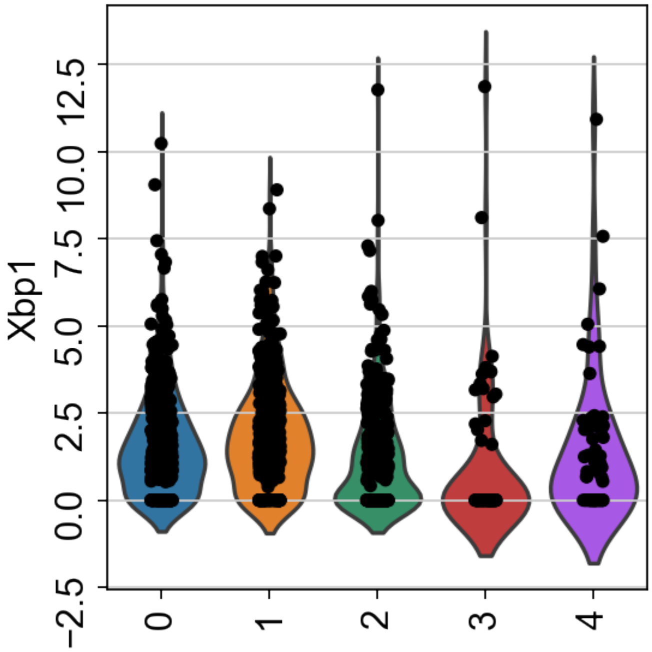

[58]:
```
if bool_plot==True:
    plot_tsne_marker(adata_adip_old, adipocyte_markers, size=10,␣
↪save="_preadipOld_markers_preadipocytes")
```

```
WARNING: saving figure to file
```

```
figures/tsne_preadipOld_markers_preadipocytes_0.pdf
```

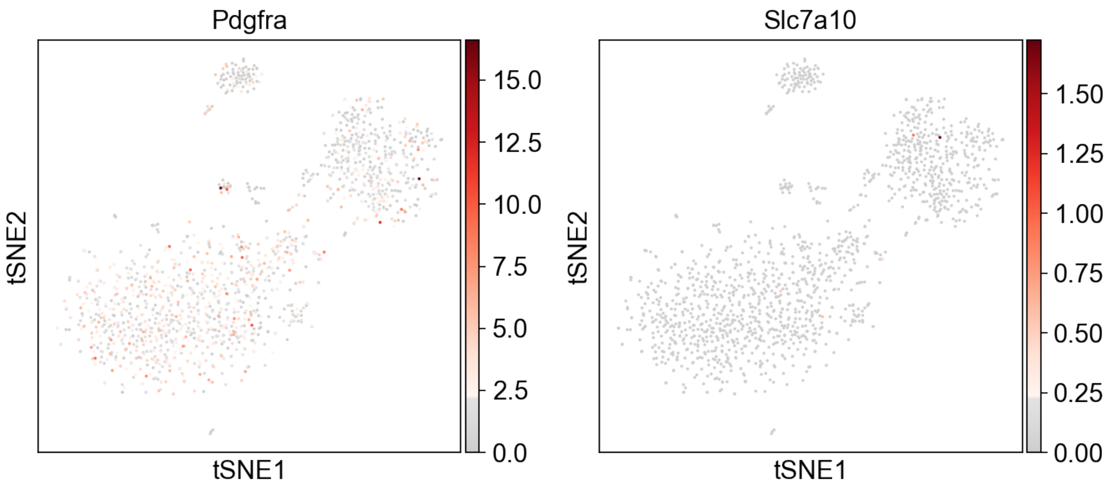


```
WARNING: saving figure to file
figures/tsne_preadipOld_markers_preadipocytes_1.pdf
```

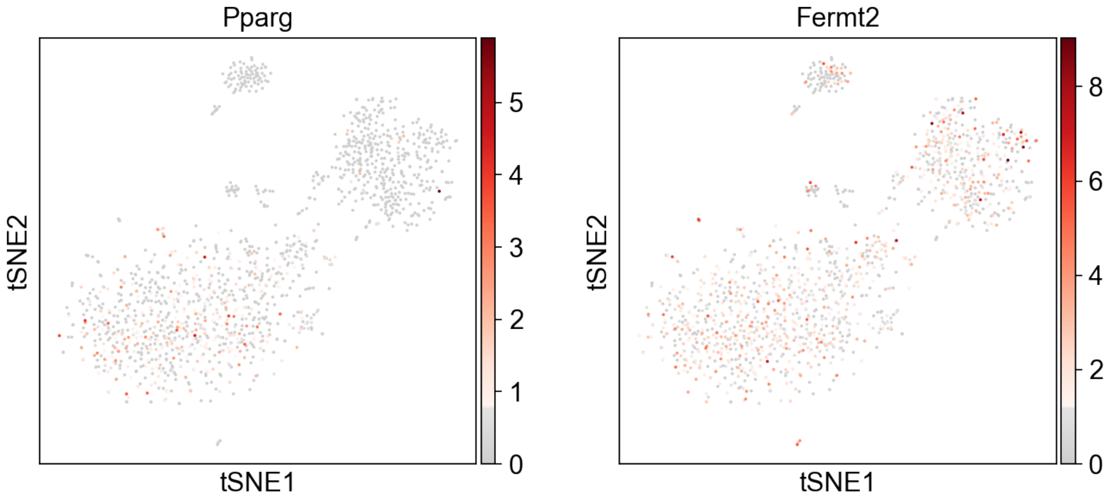


```
WARNING: saving figure to file
figures/tsne_preadipOld_markers_preadipocytes_2.pdf
```

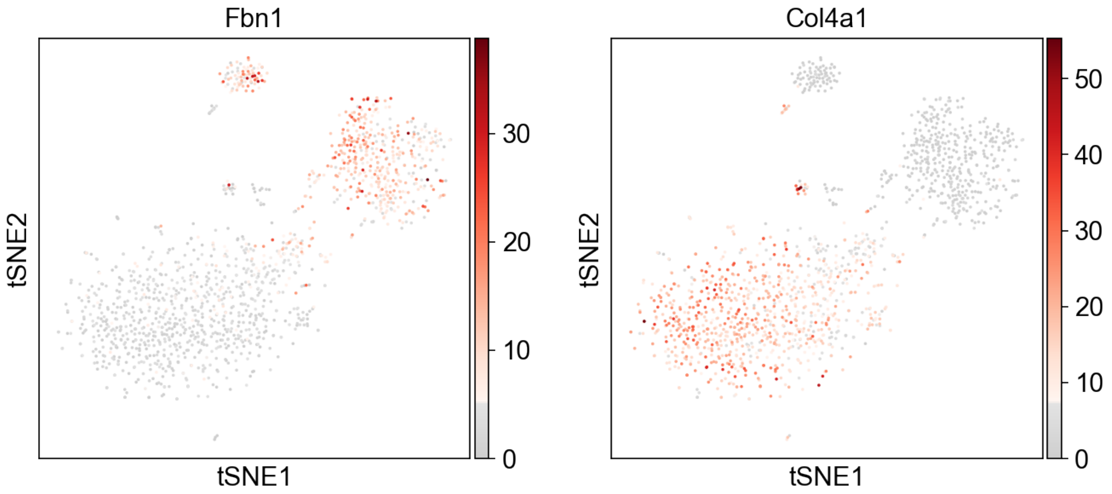

WARNING: saving figure to file
figures/tsne_preadipOld_markers_preadipocytes_3.pdf

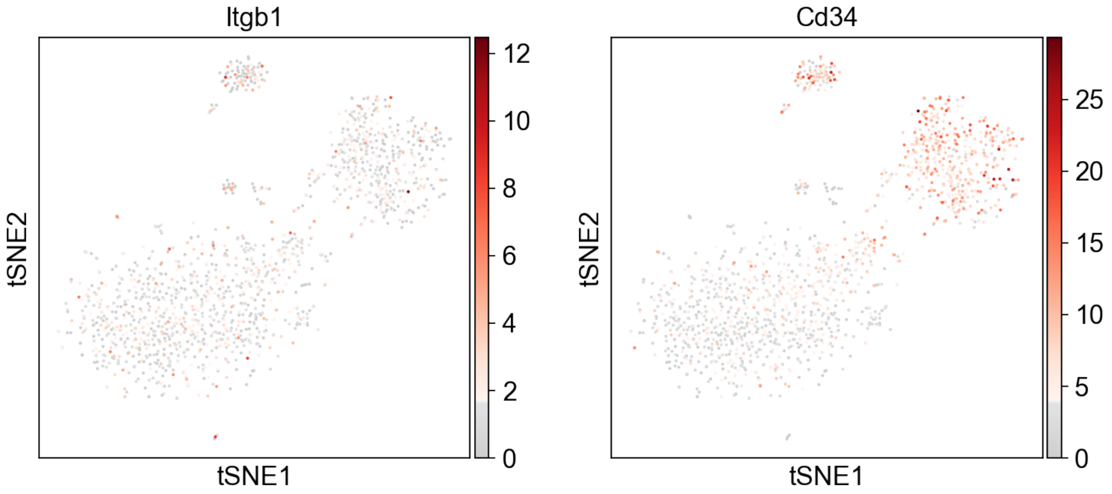

WARNING: saving figure to file
figures/tsne_preadipOld_markers_preadipocytes_4.pdf

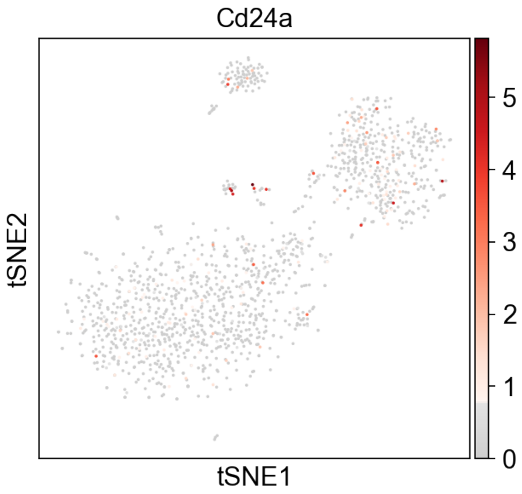

WARNING: saving figure to file
figures/tsne_preadipOld_markers_preadipocytes_5.pdf

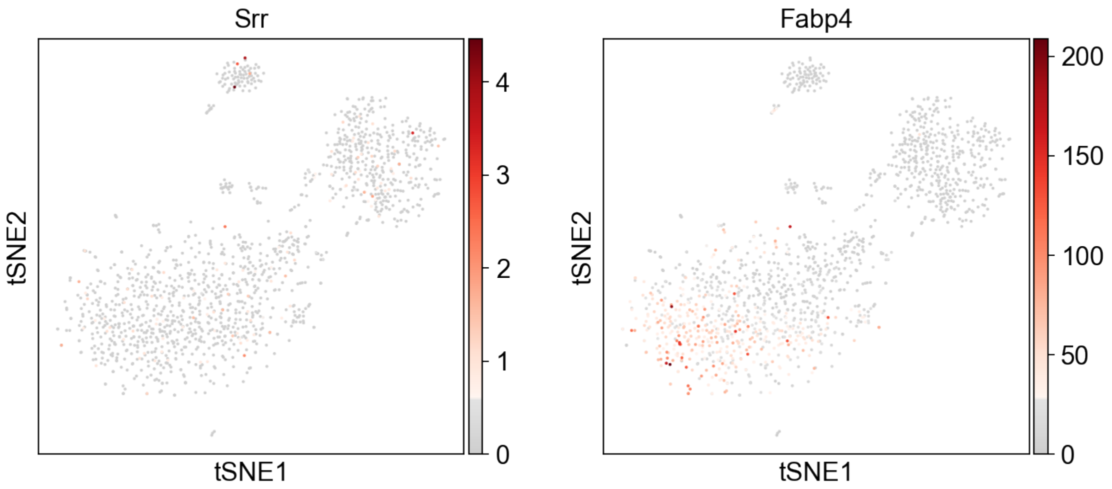

WARNING: saving figure to file
figures/tsne_preadipOld_markers_preadipocytes_6.pdf

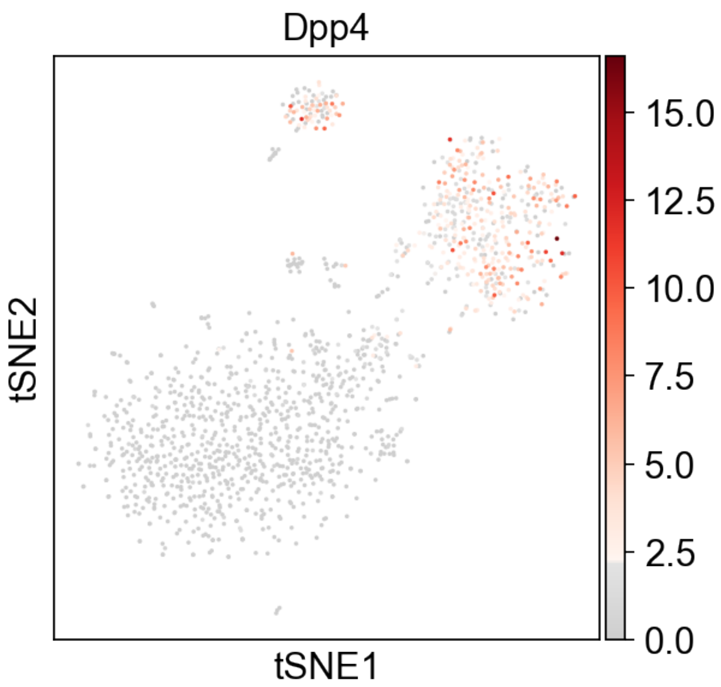

[59]:
```
if bool_plot==True:
    plot_tsne_marker(adata_adip_old, go_adip_dev, size=10,␣
↪save="_preadipOld_markers_GO_adipocyte_dev")
```

WARNING: saving figure to file
figures/tsne_preadipOld_markers_GO_adipocyte_dev_0.pdf

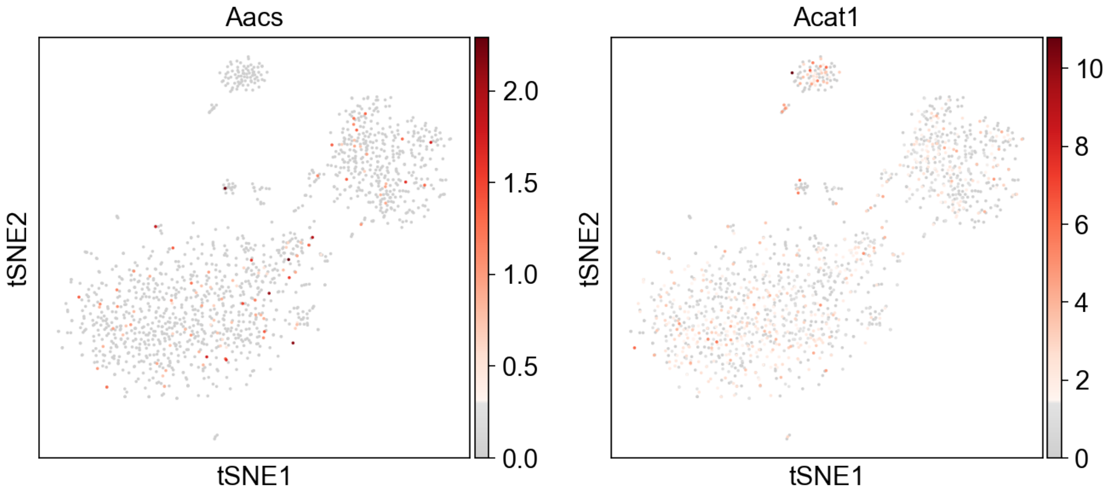

WARNING: saving figure to file

```
figures/tsne_preadipOld_markers_GO_adipocyte_dev_1.pdf
```

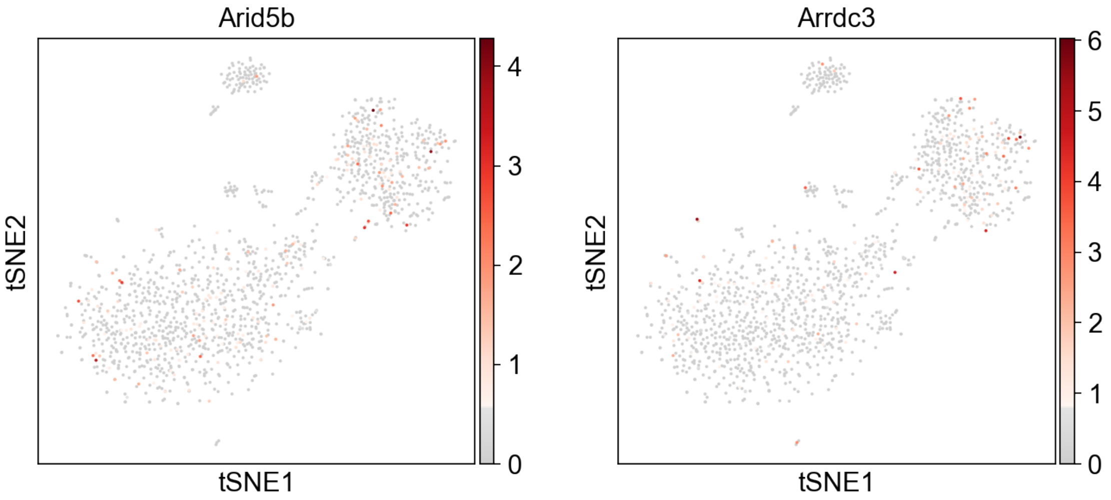


```
WARNING: saving figure to file
figures/tsne_preadipOld_markers_GO_adipocyte_dev_2.pdf
```

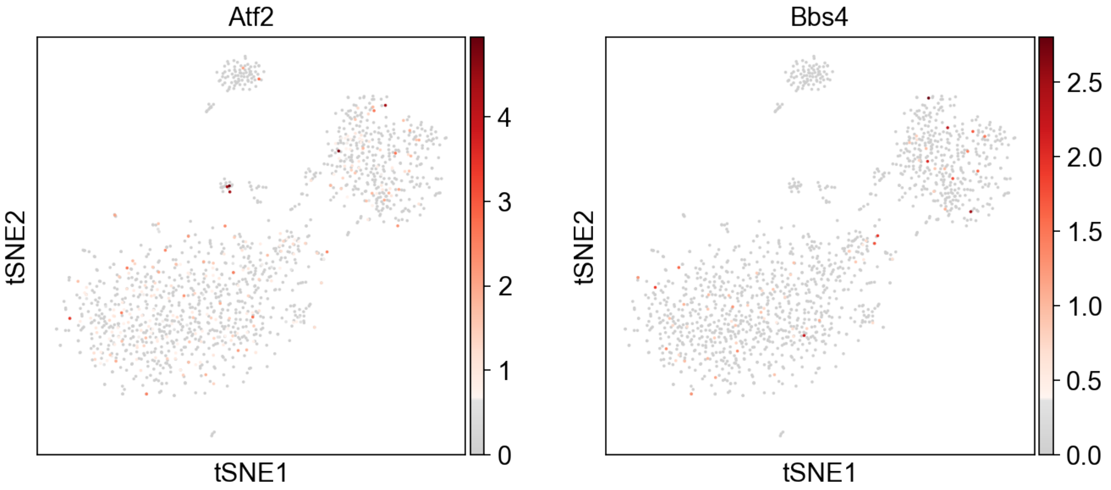


```
WARNING: saving figure to file
figures/tsne_preadipOld_markers_GO_adipocyte_dev_3.pdf
```

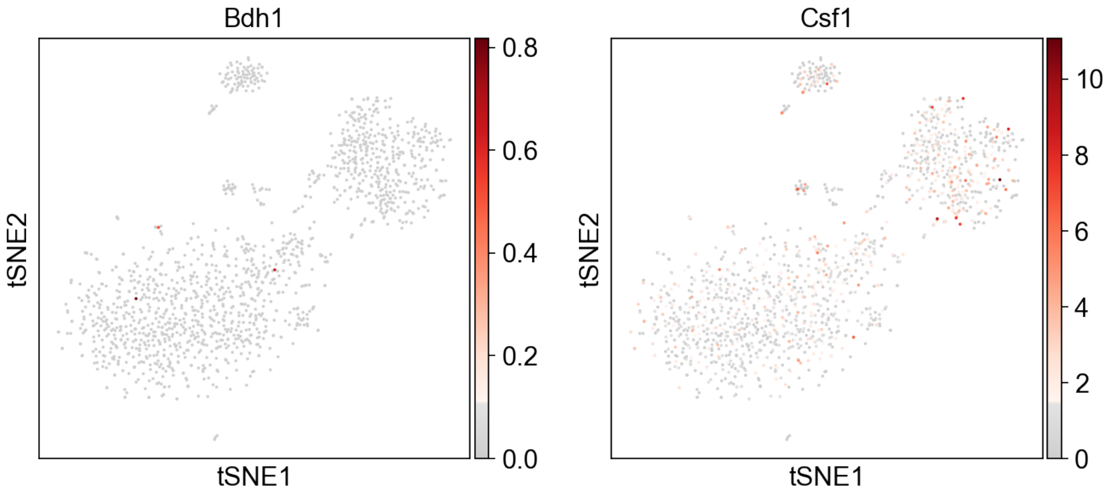


```
WARNING: saving figure to file
figures/tsne_preadipOld_markers_GO_adipocyte_dev_4.pdf
```

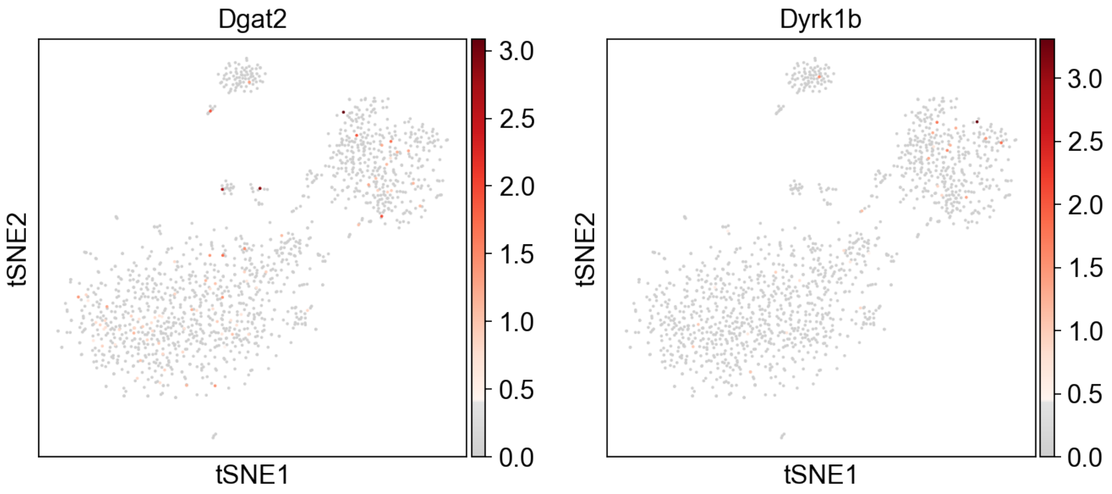


```
WARNING: saving figure to file
figures/tsne_preadipOld_markers_GO_adipocyte_dev_5.pdf
```

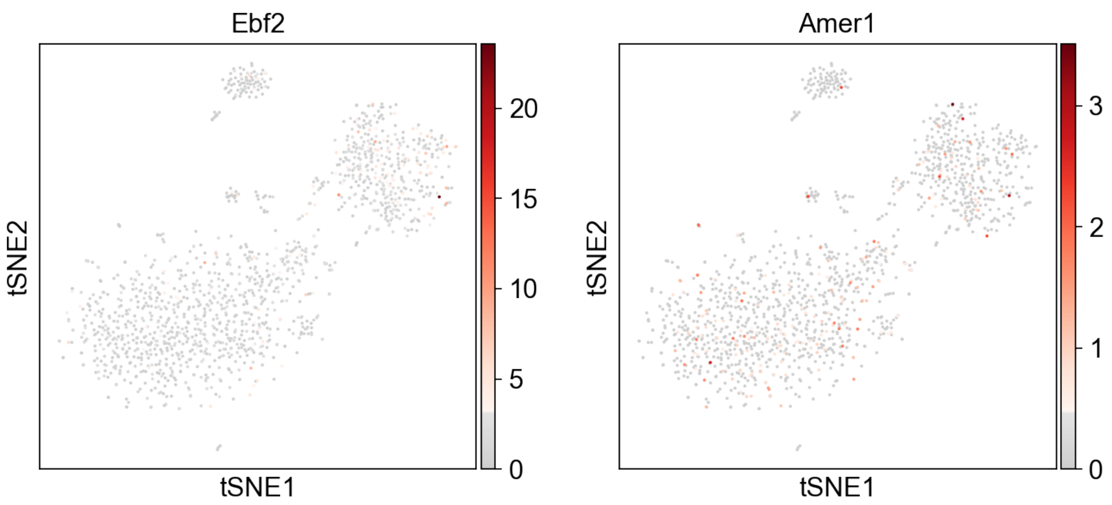


```
WARNING: saving figure to file
figures/tsne_preadipOld_markers_GO_adipocyte_dev_6.pdf
```

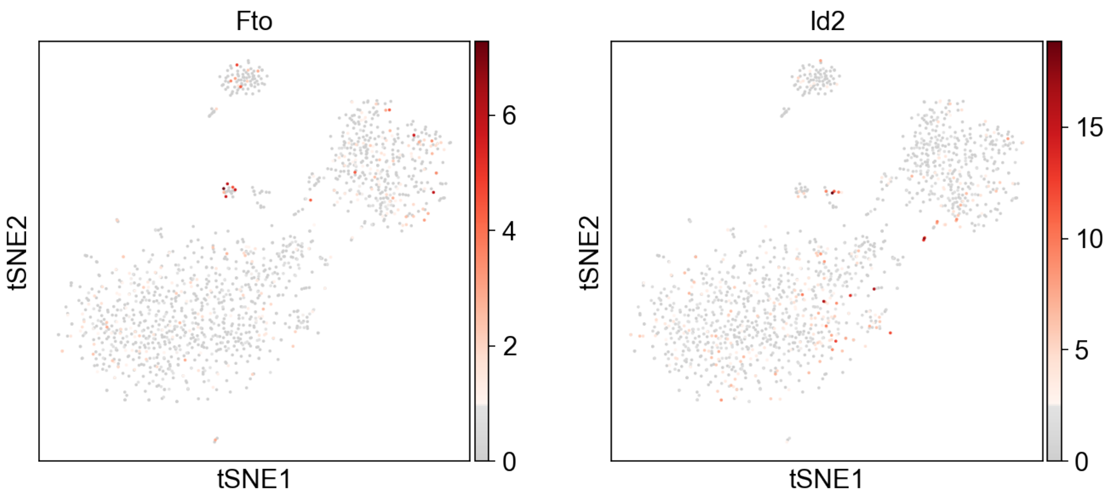


```
WARNING: saving figure to file
figures/tsne_preadipOld_markers_GO_adipocyte_dev_7.pdf
```

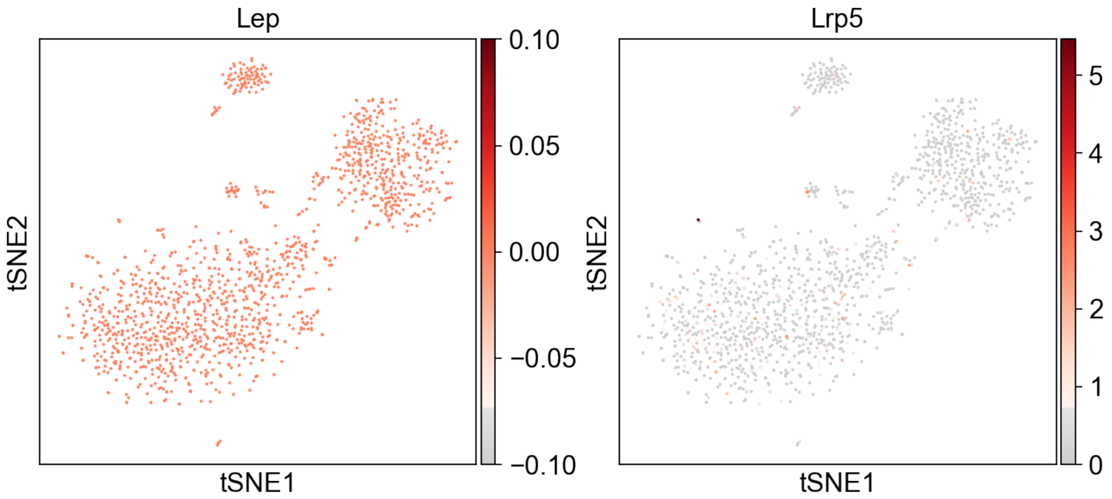

WARNING: saving figure to file
figures/tsne_preadipOld_markers_GO_adipocyte_dev_8.pdf

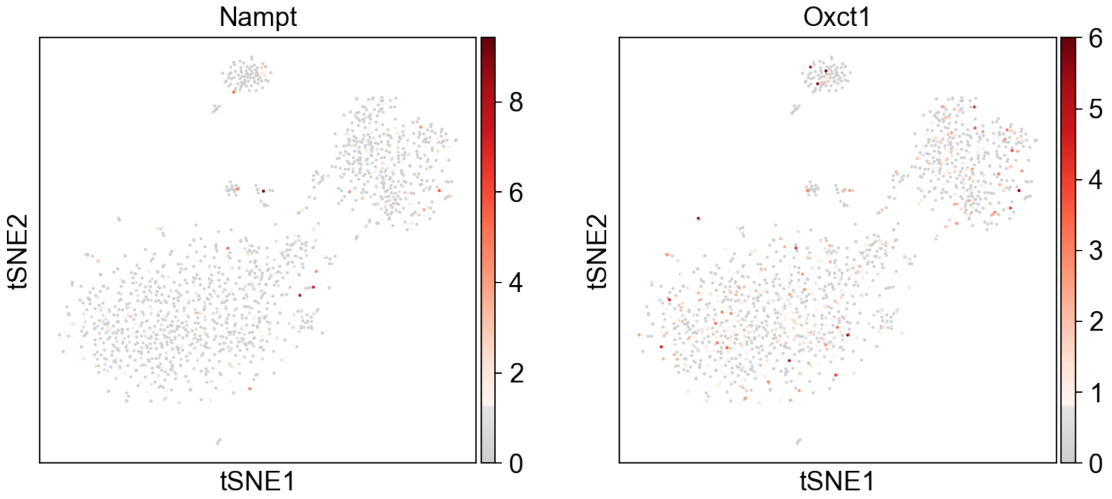

WARNING: saving figure to file
figures/tsne_preadipOld_markers_GO_adipocyte_dev_9.pdf

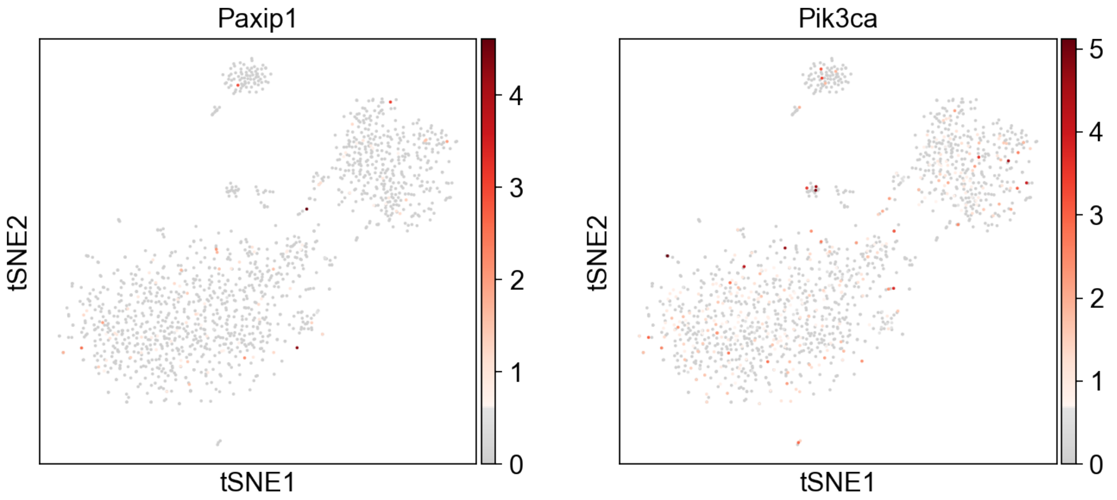

WARNING: saving figure to file
figures/tsne_preadipOld_markers_GO_adipocyte_dev_10.pdf

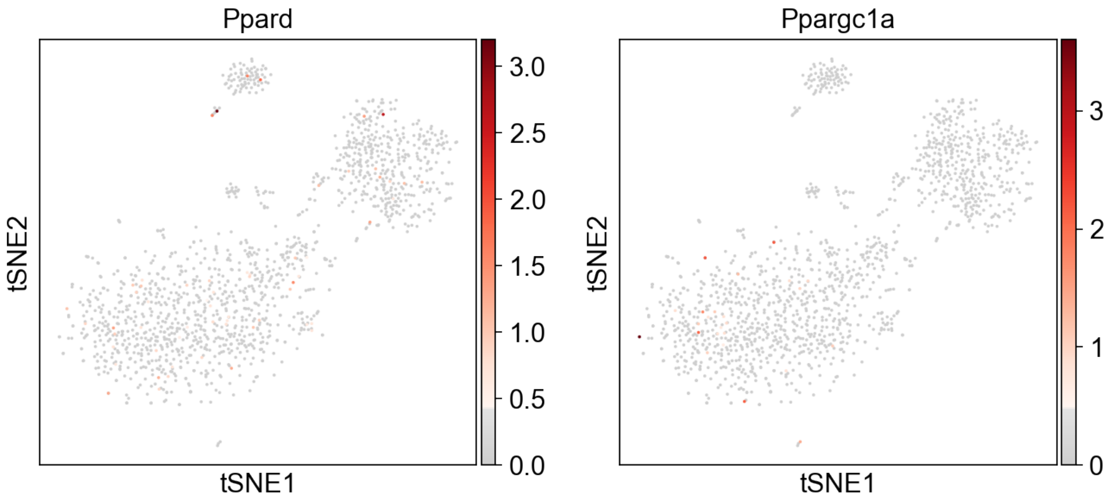

WARNING: saving figure to file
figures/tsne_preadipOld_markers_GO_adipocyte_dev_11.pdf

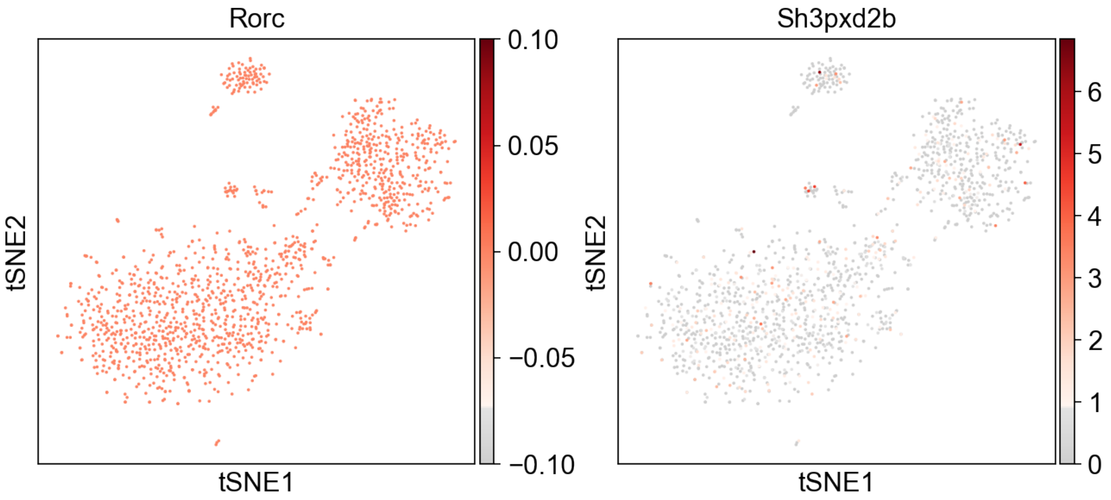

WARNING: saving figure to file
figures/tsne_preadipOld_markers_GO_adipocyte_dev_12.pdf

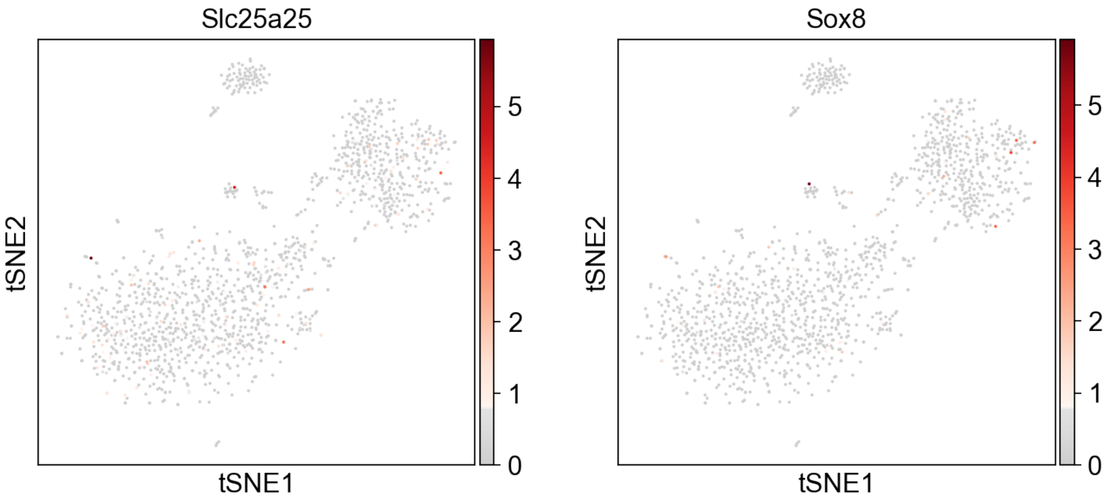

WARNING: saving figure to file
figures/tsne_preadipOld_markers_GO_adipocyte_dev_13.pdf

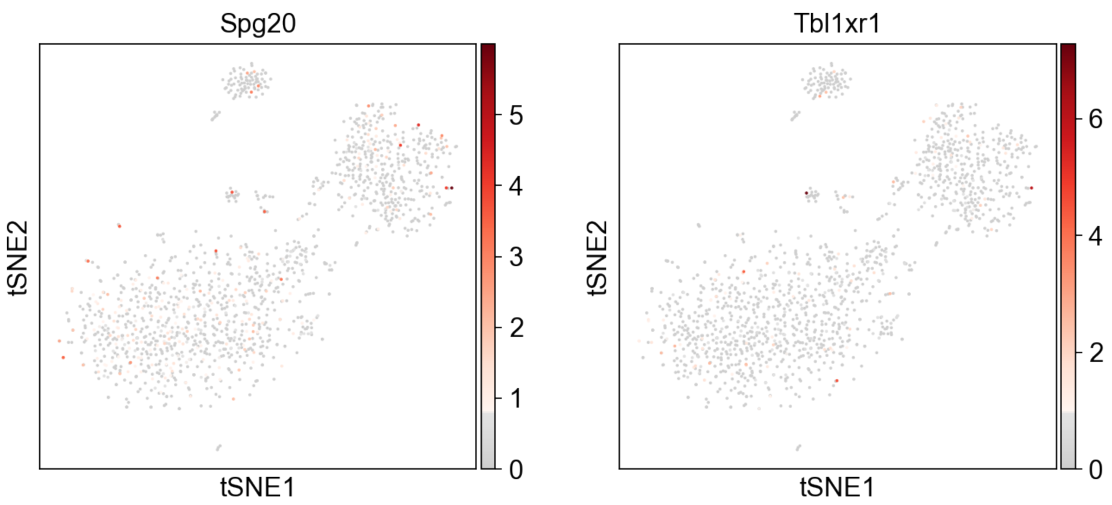

WARNING: saving figure to file
figures/tsne_preadipOld_markers_GO_adipocyte_dev_14.pdf

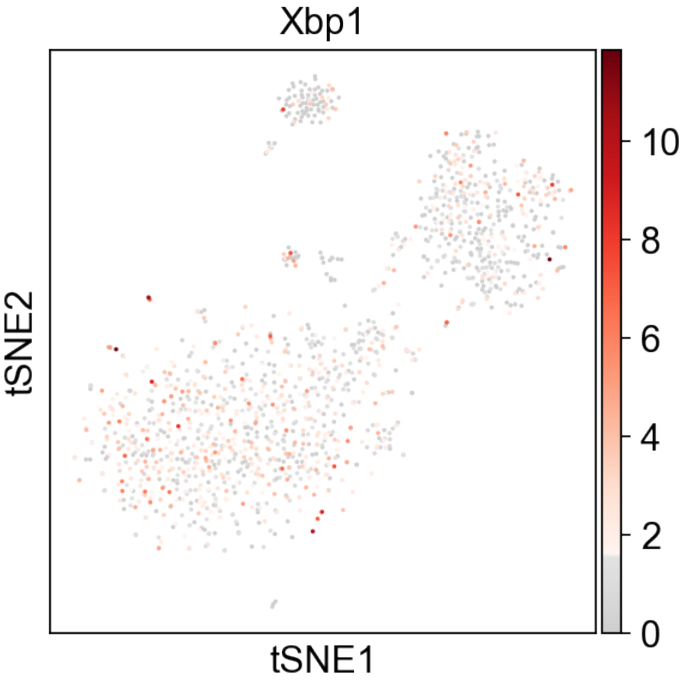

[60]:
```
if bool_plot==True:
    sc.pl.heatmap(
        adata=adata_adip_old,
        var_names=adipocyte_markers,
```

```
        groupby="louvain",
        use_raw=True,
        log=True,
        dendrogram=False,
        var_group_rotation=90,
        show_gene_labels=True,
        show=True,
        save="_preadipOld_markers_preadipocytes.pdf"
    )
```

```
WARNING: saving figure to file
figures/heatmap_preadipOld_markers_preadipocytes.pdf
```

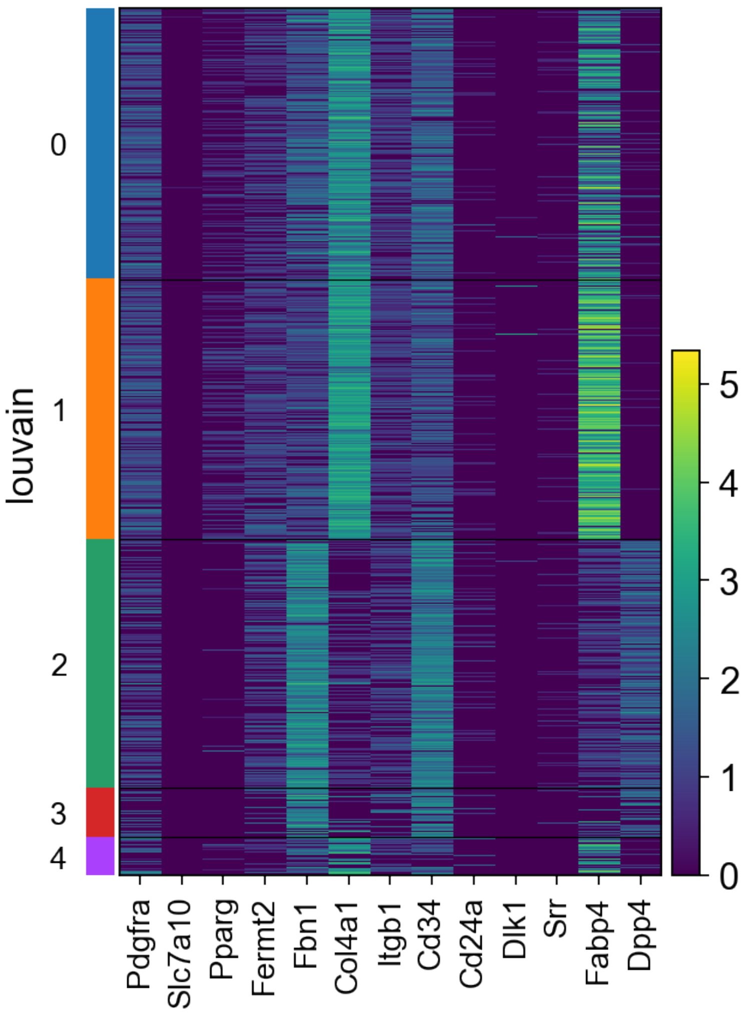

[61]:
```
if bool_plot==True:
    sc.pl.heatmap(
```

```
        adata=adata_adip_old,
        var_names=go_adip_dev,
        groupby="louvain",
        use_raw=True,
        log=True,
        dendrogram=False,
        var_group_rotation=90,
        show_gene_labels=True,
        show=True,
        save="_preadipOld_markers_GO_adipocyte_dev.pdf"
    )
```

```
WARNING: saving figure to file
figures/heatmap_preadipOld_markers_GO_adipocyte_dev.pdf
```

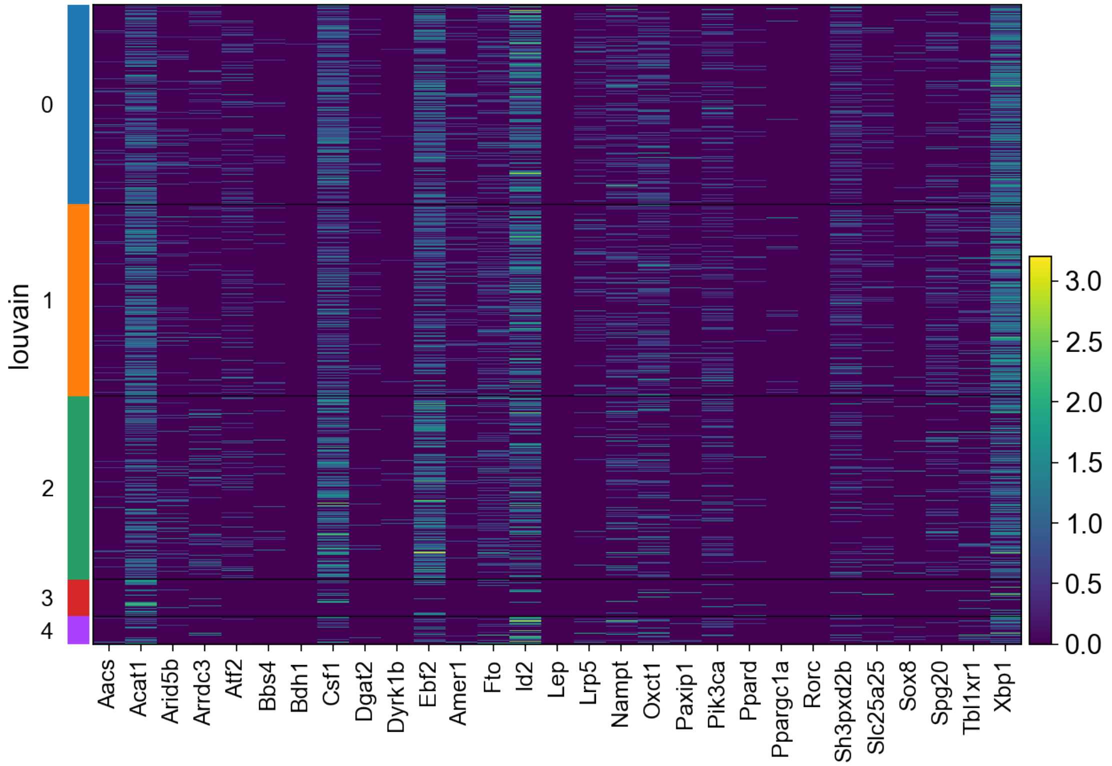

This looks like there are two clusters, one consisting of louvain group 0,4 (Pdgfra-Slc7a10-) and one of group 1,2,3,5 (Pdgfra+Slc7a10-).

8 Differential expression analysis

8.1 By age

[62]:
```
dets_age = de.test.t_test(
    data=adata_adip.raw,
    sample_description=adata_adip.obs,
    grouping="age",
    is_logged=False
)
```

The differentially expressed genes with some fold-change and mean expression thresholding are:

[63]:
```
dets_age_summary = dets_age.summary(
    mean_thres=np.log(0.01), qval_thres=0.05,
    fc_lower_thres=0.5, fc_upper_thres=2
)
dets_age_summary.to_csv(path_or_buf=dir_tables+"DE_preadip_by_age.tab",␣
↪sep="\t")
dets_age_summary
```

[63]:
```
                gene          pval          qval    log2fc      mean  zero_mean  \
31            Mcmdc2  7.304947e-04  4.593177e-03 -1.768519  0.013358      False
40             Prex2  2.570578e-05  2.197343e-04 -1.089182  0.093344      False
44             Sulf1  4.526136e-60  3.844565e-58  1.583435  0.653168      False
54            Gm9947  5.757802e-04  3.713231e-03 -1.209250  0.032041      False
55               Msc  5.399946e-12  9.914974e-11 -1.315166  0.147970      False
…                ...           ...           ...       ...       ...        ...
31004        Hspa12a  4.379431e-08  5.549928e-07 -1.551823  0.063218      False
31006         Shtn1  8.864250e-03  3.920102e-02 -2.354673  0.012819      False
31024        Nanos1  1.162409e-02  4.903703e-02  2.189834  0.009470      False
31029          Grk5  1.745639e-05  1.542527e-04 -1.076947  0.064772      False
31113     AC125149.3  7.552487e-03  3.419382e-02 -2.116613  0.013095      False

       zero_variance
31             False
40             False
44             False
54             False
55             False
…                ...
31004          False
31006          False
31024          False
31029          False
31113          False

[1500 rows x 7 columns]
```

[64]:
```
dets_age.plot_volcano(
    alpha=0.01, min_fc=1.5, size=15, log10_p_threshold=-50, log2_fc_threshold=7,
    highlight_ids=['Slc7a10'], highlight_size=30, highlight_col="red",
    save=sc_settings_figdir+"preadip_DE_age", suffix="_volcano.pdf"
)
```

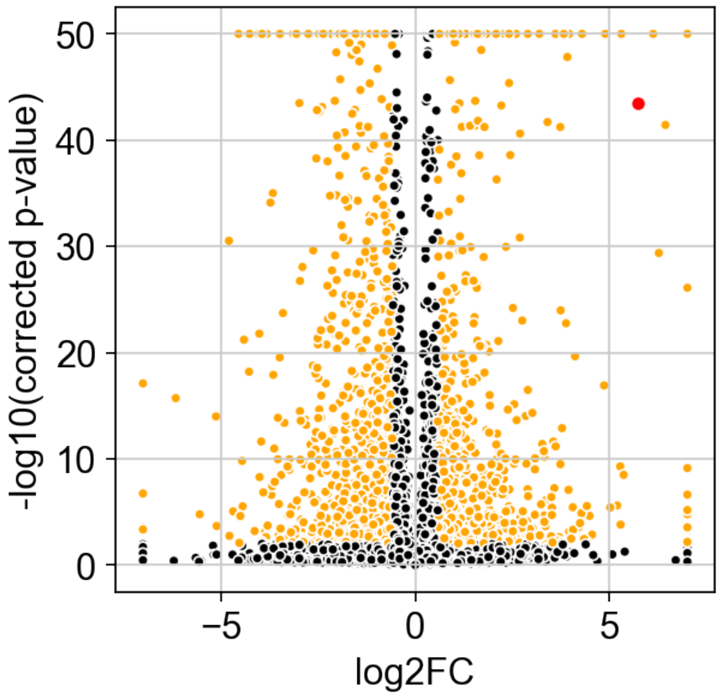

[65]:
```
dets_age.plot_ma(
    alpha=0.01, log2_fc_threshold=6,
    highlight_ids=['Slc7a10'], highlight_size=30, highlight_col="red",
    save=sc_settings_figdir+"preadip_DE_age", suffix="_ma_plot.pdf"
)
```

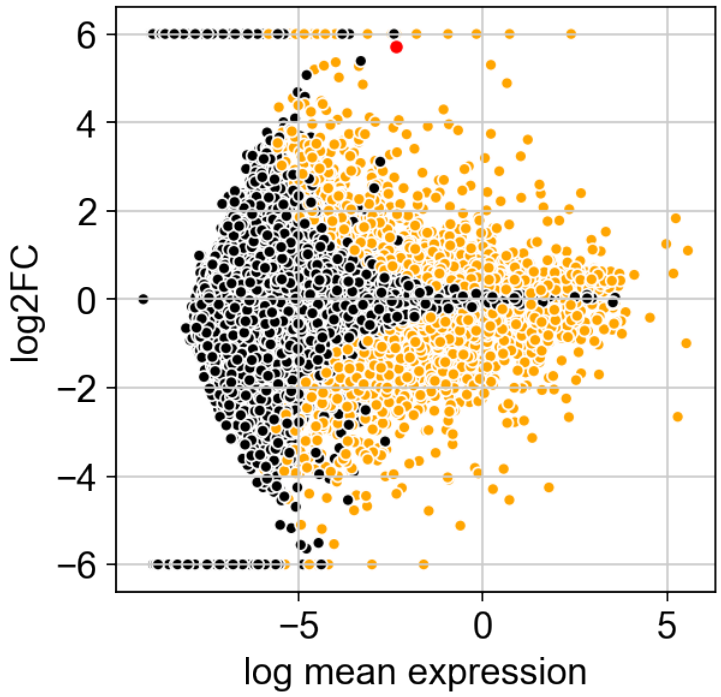

Count DE genes:

```
[66]: np.sum(np.array([x < 0.01 if x is not np.nan else False for x in dets_age.
       ↪qval]))
```

```
[66]: 3359
```

8.2 By louvain group

8.2.1 Coarse clustering

Test Here, we perform a differential expression test across the coarse-grained louvain clustering which yielded two groups.

```
[67]: dets_young_lowres_louvain = de.test.t_test(
          data=adata_adip_young_lowres.raw,
          sample_description=adata_adip_young_lowres.obs,
          grouping="louvain",
          is_logged=False
      )
```

The differentially expressed genes with some fold-change and mean expression thresholding are:

```
[68]: dets_young_lowres_louvain_summary = dets_young_lowres_louvain.summary(
          mean_thres=np.log(0.01), qval_thres=0.05,
```

```
    fc_lower_thres=0.5, fc_upper_thres=2
)
dets_young_lowres_louvain_summary.
 ↪to_csv(path_or_buf=dir_tables+"DE_preadipYoung_by_louvain_lowres.csv",␣
 ↪sep="\t")
dets_young_lowres_louvain_summary
```

[68]:

```
                gene          pval          qval       log2fc      mean  \
22             Sntg1  9.307578e-05  9.000487e-04    -2.938886  0.020287
24            Adhfe1  1.195094e-10  2.535832e-09     4.923560  0.045822
44             Sulf1  7.783329e-81  1.888088e-78    -2.226169  1.056395
54            Gm9947  2.906599e-06  3.674391e-05     4.928583  0.022184
55               Msc  1.458330e-13  3.861133e-12     4.531582  0.097486
…                  …             …             …            …         …
30997          Gfra1  2.004776e-03  1.404891e-02  1069.601729  0.015102
31014         Emx2os  7.788998e-13  1.972078e-11    -2.070294  0.118866
31015           Emx2  1.216663e-15  3.652226e-14    -1.965351  0.199940
31019  E330013P04Rik  1.345002e-04  1.250991e-03    -2.148886  0.043125
31081     CR974586.5  3.477534e-03  2.267534e-02     3.360395  0.010408

       zero_mean  zero_variance
22         False          False
24         False          False
44         False          False
54         False          False
55         False          False
…            …            …
30997      False          False
31014      False          False
31015      False          False
31019      False          False
31081      False          False

[1439 rows x 7 columns]
```

[69]:

```
dets_young_lowres_louvain.plot_volcano(
    alpha=0.01, min_fc=1.5, size=15, log10_p_threshold=-15, log2_fc_threshold=5,
    highlight_ids=['Slc7a10'], highlight_size=30, highlight_col="red",
    save=sc_settings_figdir+"preadipYoung_DE_louvain_lowres", suffix="_volcano.
 ↪pdf"
)
```

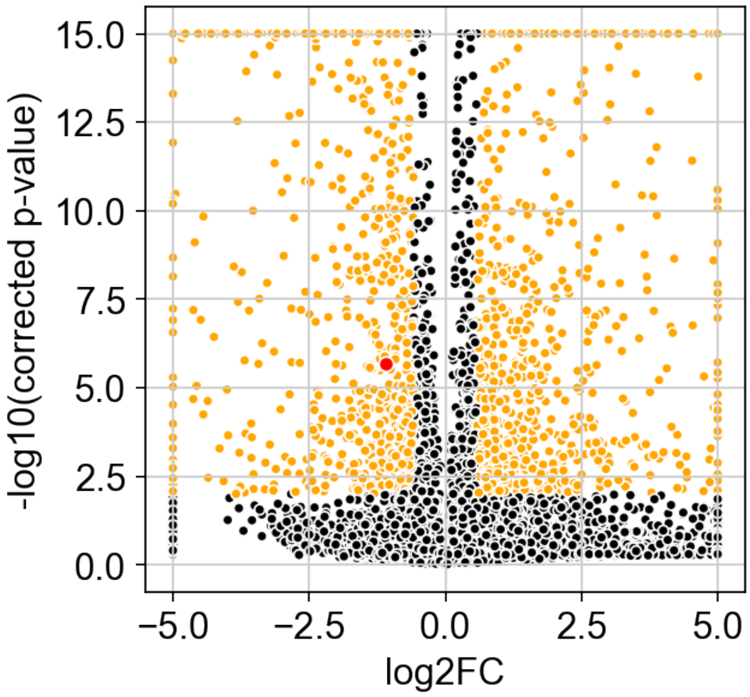

[70]:
```
dets_young_lowres_louvain.plot_ma(
    alpha=0.01, log2_fc_threshold=5,
    highlight_ids=['Slc7a10'], highlight_size=30, highlight_col="red",
    save=sc_settings_figdir+"preadipYoung_DE_louvain_lowres", suffix="_ma_plot.
↪pdf"
)
```

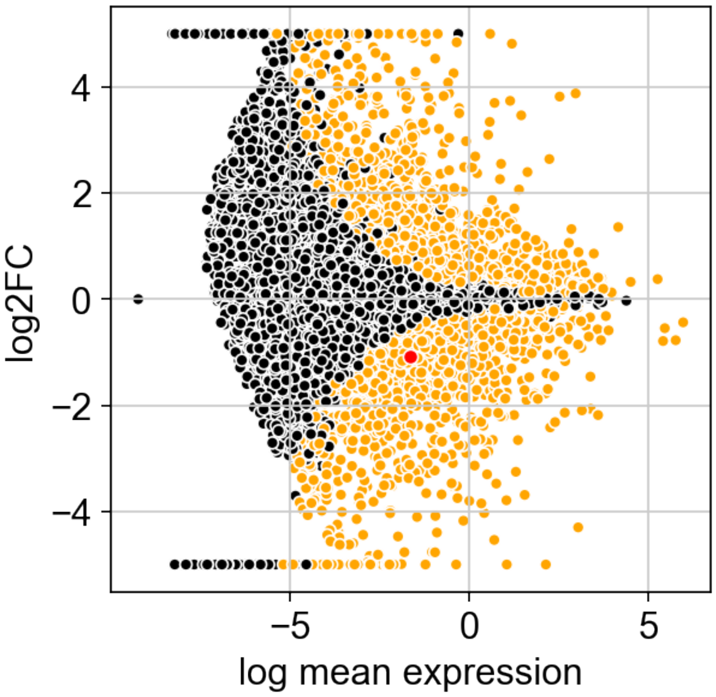

Count DE genes:

```
[71]: np.sum(np.array([x < 0.01 if x is not np.nan else False for x in␣
      ↪dets_young_lowres_louvain.qval]))
```

```
[71]: 2442
```

Save DE genes to file for enrichment:

```
[72]: dets_young_lowres_louvain.summary(qval_thres=0.01)["gene"].to_csv(
          dir_out+"enrichment/de_genes_young_preadip.csv", index=False)
```

```
/Users/viktorian.miok/anaconda3/lib/python3.7/site-
packages/ipykernel_launcher.py:2: FutureWarning: The signature of
`Series.to_csv` was aligned to that of `DataFrame.to_csv`, and argument 'header'
will change its default value from False to True: please pass an explicit value
to suppress this warning.
```

Heatmaps Select all differentially expressed genes at a corrected p-value threshold of 0.01 and a minimal or maximal log2 fold change of 2 or 0.5 and a minimal mean expression of 0.5.

```
[73]: dets_young_lowres_louvain_summary_forheatmap = dets_young_lowres_louvain.
      ↪summary(
          mean_thres=0.5, qval_thres=0.01,
```

```
    fc_lower_thres=0.5, fc_upper_thres=2
)
```

[74]:
```
all_de_genes_young_coarse =␣
↪dets_young_lowres_louvain_summary_forheatmap['gene'].values[
    np.argsort(dets_young_lowres_louvain_summary_forheatmap['log2fc'].values)
]
```

[75]:
```
print(len(all_de_genes_young_coarse))
```

247

[76]:
```
if bool_plot==True:
    sc.pl.heatmap(
        adata=adata_adip_young_lowres,
        var_names=all_de_genes_young_coarse,
        groupby="louvain",
        use_raw=False,
        log=False,
        cmap="viridis",
        vmin=-1,
        vmax=5,
        dendrogram=False,
        var_group_rotation=90,
        show_gene_labels=True,
        show=True,
        save="_preadipYoung_lowres_all_de_genes.pdf"
    )
```

WARNING: saving figure to file
figures/heatmap_preadipYoung_lowres_all_de_genes.pdf

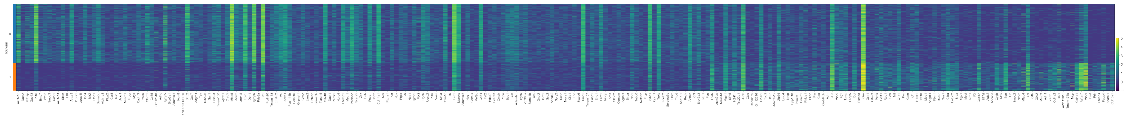

Supplement: Supplementary file 5 — Supplementary Data 3 [file 41467_2021_21826_MOESM5_ESM.pdf]
